# Supplementary material for: Development of Fluorescently Labeled SSEA-3, SSEA-4, and Globo-H Glycosphingolipids for Elucidating Molecular Interactions in the Cell Membrane
Source: Int J Mol Sci. 2019 Dec 7;20(24):6187. doi: 10.3390/ijms20246187 (PMC6941013; doi:10.3390/ijms20246187)
Supplement: Supplementary file 1 [file ijms-20-06187-s001.pdf]

Supporting Information

Development of fluorescently labeled SSEA-3, SSEA-4, and Globo-H glycosphingolipids for elucidating molecular interactions in the cell membrane

Sachi Asano [2,3], Rita Pal [3], Hide-Nori Tanaka [1,2,3], Akihiro Imamura [2,3], Hideharu Ishida [1,2,3], Kenichi G. N. Suzuki [1,2,3,*], and Hiromune Ando [1,2,3,*]

[1] *Center for Highly Advanced Integration of Nano and Life Sciences (G-CHAIN), Gifu University, 1-1 Yanagido, Gifu 501-1193, Japan*

[2] *The United Graduate School of Agricultural Science, Gifu University, 1-1 Yanagido, Gifu 501-1193, Japan*

[3] *Department of Applied Bioorganic Chemistry, Gifu University, 1-1 Yanagido, Gifu 501-1193, Japan*

** Correspondence: kgsuzuki@gifu-u.ac.jp (K. G. N. S.); hando@gifu-u.ac.jp (H. A.)*

Table of contents

1. General information
2. Synthetic procedures
3. ^{1}H and ^{13}C NMR spectral data for new compounds
4. References

1. General information

All reactions were performed under an argon atmosphere. All chemicals were purchased from commercial suppliers and used without further purification, if not stated otherwise. Molecular sieves were purchased from FUJIFILM Wako Pure Chemical Co. (Osaka, Japan) and pre-dried at 300 °C for 2 h in a muffle furnace, and dried in a flask at 300 °C for 2 h in vacuo prior to use. Dry solvents for reaction media (CH_2Cl_2, toluene, THF, MeCN, DMF, and pyridine) were purchased from Kanto Chemical Co., Inc. (Tokyo, Japan) and used without purification. Other solvents for reaction media were dried over molecular sieves and used without purification. TLC analysis was performed on Merck TLC plates (silica gel $60F_{254}$ on glass plates), and visualized either by exposure to UV light (253.6 nm) or by soaking in a solution of 10% H_2SO_4 in ethanol followed by heating. Purification by flash column chromatography was performed on a silica gel (80 and 300 mesh; Fuji Silysia Chemical, Ltd., Aichi, Japan). The quantity of silica gel was usually estimated as 50- to 100-fold weight of crude sample to be charged. Solvent systems for chromatography were specified as *v*/*v* ratios. Evaporation, concentration, and drying-up were performed *in vacuo*. ^{1}H and ^{13}C NMR spectra were recorded on 500 and 800 MHz spectrometers (Biospin AVANCE III; Bruker, Billerica, MA, USA). Chemical shifts (δ) were measured in ppm relative to TMS δ = 0.00 ppm as an internal standard. Data are presented as follows: chemical shift, multiplicity (s = singlet, d = doublet, t = triplet, q = quartet, quin = quintet, sext = sextet, dd = double doublet, dt = double triplet, and m = multiplet and/or multiple resonances), integration, coupling constant in Hz, and position of the corresponding proton. ^{1}H-^{1}H COSY, ^{1}H-^{13}C HMBC and ^{1}H-^{13}C HMQC methods were used to confirm the NMR signal assignments. High-resolution mass (ESI-TOF MS) spectra were taken with a Bruker micrOTOF. Optical rotations were measured with a high-sensitivity polarimeter (SEPA-300; Horiba, Kyoto, Japan). All analogs labeled with ATTO594-NHS ester (ATTO-TEC; AD 594-35) were stored at −80 °C in glass vials after freeze-drying.

2. Synthetic procedures

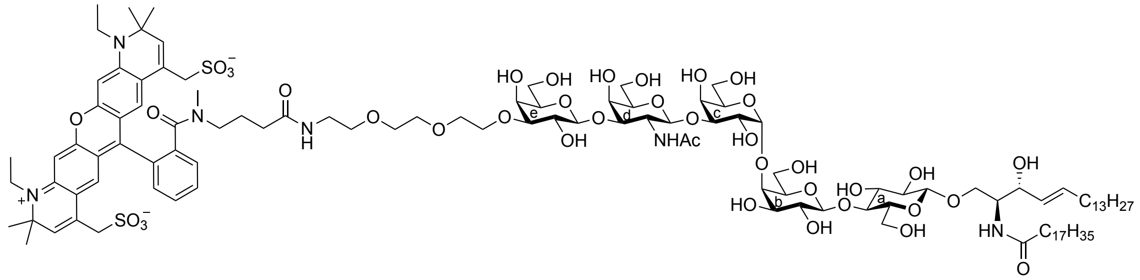

ATTO594 SSEA-3 (1)

To a solution of compound **20** (3.4 mg, 2.50 µmol) in DMF/H_2O = 11/1 (0.22 mL) were added ATTO594 NHS ester (4.6 mg, 3.29 µmol) and Et_3N (6.10 µL, 43.9 µmol). After stirring for 4 h at ambient temperature as the progress of the reaction was monitored by TLC ($CHCl_3$/MeOH/H_2O/AcOH = 5/4/1/0.2), the reaction mixture was purified by gel filtration (LH20; $CHCl_3$/MeOH = 1/1) and PTLC ($CHCl_3$/MeOH/H_2O = 5/4/1) to give compound **1** (5.1 mg, 100%) as a blue syrup; ^{1}H NMR (500 MHz, CD_3OD) δ 7.75–5.89 (m, 9 H, $Ar^{ATTO594}$), 5.68 (near quin, 1 H, $J_{5,6a}$ = $J_{5,6b}$ = 6.5 Hz, $J_{4,5}$ = 15.3 Hz, H-5^{Cer}), 5.44 (dd, 1 H, $J_{3,4}$ = 7.5 Hz, H-4^{Cer}), 4.94 (d, 1 H, $J_{1,2}$ = 4.0 Hz, H-1^c), 4.73–3.20 (m, 56 H, H-1^a, H-2^a, H-3^a, H-4^a, H-5^a, H-$6a^a$, H-$6b^a$, H-1^b, H-2^b, H-3^b, H-4^b, H-5^b, H-$6a^b$, H-$6b^b$, H-2^c, H-3^c, H-4^c, H-5^c, H-$6a^c$, H-$6b^c$, H-1^d, H-2^d, H-3^d, H-4^d, H-5^d, H-$6a^d$, H-$6b^d$, H-1^e, H-2^e, H-3^e, H-4^e, H-5^e, H-$6a^e$, H-$6b^e$, H-$1a^{Cer}$, H-$1b^{Cer}$, H-2^{Cer}, H-3^{Cer}, 5 OCH_2, $NHCH_2$, 3 $NCH_2^{ATTO594}$, 2 $CH_2SO_3^{ATTO594}$), 2.72 and 2.64 (2 s, 3 H, $NCH_3^{ATTO594}$), 2.18–2.15 (m, 2 H, NHC(=O)CH_2^{Cer}), 2.03–2.00 (m, 2 H, H-$6a^{Cer}$, H-$6b^{Cer}$), 1.97 (m, 3 H, Ac), 1.78–1.29 (m, 74 H, 26 CH_2^{Cer}, $NCH_2CH_2^{ATTO594}$, $NCH_2CH_2CH_2^{ATTO594}$, 2 $NCH_2CH_3^{ATTO594}$, 4 $Me^{ATTO594}$), 0.91–0.88 (m, 6 H, 2 Me^{Cer}); ^{13}C NMR (200 MHz, CD_3OD) δ 180.3, 176.0, 175.2, 175.1, 175.0, 173.0, 171.5, 171.3, 159.2, 155.1, 154.2, 138.4, 137.6, 137.4, 135.1, 132.2, 131.9, 131.8, 131.4, 131.2, 130.7, 130.5, 129.3, 129.0, 126.1, 124.3, 124.3, 124.2, 122.9, 115.0, 106.3, 105.6, 104.4, 104.3, 102.8, 97.0, 96.9, 83.3, 83.2, 81.3, 81.2, 80.8, 80.0, 80.0, 79.5, 79.0, 76.5, 76.4, 76.4, 76.3, 75.0, 74.7, 73.0, 72.6, 72.5, 71.7, 71.6, 71.5, 71.5, 71.1, 70.6, 70.5, 70.0, 69.7, 69.7, 69.5, 69.5, 66.8, 64.3, 62.7, 61.8, 61.7, 61.6, 61.5, 58.4, 54.7, 54.1, 54.1, 53.9, 53.9, 53.4, 53.0, 50.4, 47.5, 46.8, 41.4, 40.4, 40.3, 38.8, 37.4, 33.7, 33.5, 33.4, 33.2, 33.1, 32.9, 32.8, 32.7, 30.9, 30.9, 30.9, 30.9, 30.8, 30.8, 30.7, 30.6, 30.5, 30.5, 30.5, 29.5, 29.4, 29.3, 27.2, 25.1, 24.2, 23.8, 23.7, 23.7, 23.5, 23.0, 20.9, 18.4, 14.5, 14.5, 14.4, 13.8, 13.7; HRMS (ESI) *m/z*: found [(M–H)/2]$^-$ 1166.5867, $C_{115}H_{181}N_6O_{39}S_2^-$ calcd for [(M–H)/2]$^-$ 1166.5869.

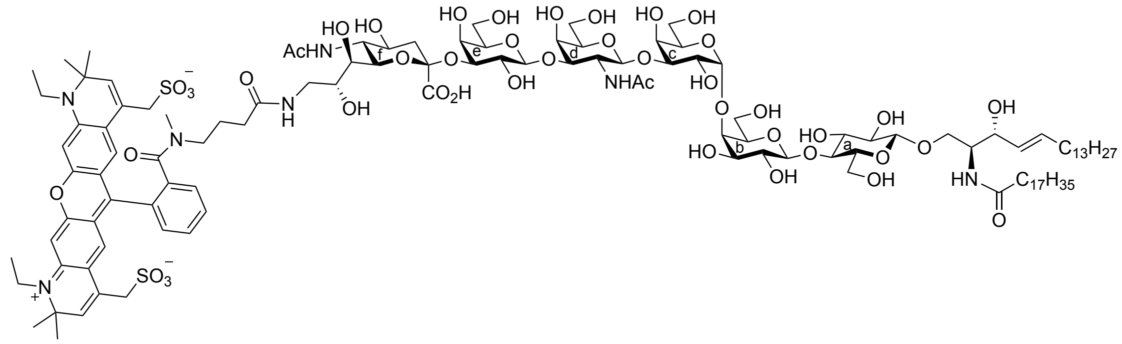

ATTO594 SSEA-4 (2)

To a solution of compound **38** (3.5 mg, 2.05 μmol) in DMF/H_2O = 11/1 (0.21 mL) were added ATTO594 NHS ester (4.3 mg, 3.07 μmol) and Et_3N (5.70 μL, 41.0 μmol). After stirring for 22 h at ambient temperature as the progress of the reaction was monitored by TLC ($CHCl_3$/MeOH/5% aq. $CaCl_2$ = 5/4/1), the reaction mixture was purified by gel filtration (LH20; $CHCl_3$/MeOH = 1/1) and PTLC (1st: $CHCl_3$/MeOH/H_2O = 5/4/1, 2nd: $CHCl_3$/MeOH/H_2O = 5/4/1, 3rd: $CHCl_3$/MeOH/H_2O = 5/5/0.5) to give compound **2** (4.2 mg, 82%) as a blue syrup; ^{1}H NMR (500 MHz, CD_3OD) δ 7.75–5.88 (m, 9 H, Ar^{ATTO594}), 5.68 (near quin, 1 H, $J_{5,6a}$ = $J_{5,6b}$ = 6.5 Hz, $J_{4,5}$ = 15.4 Hz, H-5^{Cer}), 5.44 (dd, 1 H, $J_{3,4}$ = 7.8 Hz, H-4^{Cer}), 4.94–3.16 (m, 58 H, H-1^a, H-2^a, H-3^a, H-4^a, H-5^a, H-6a^a, H-6b^a, H-1^b, H-2^b, H-3^b, H-4^b, H-5^b, H-6a^b, H-6b^b, H-1^c, H-2^c, H-3^c, H-4^c, H-5^c, H-6a^c, H-6b^c, H-1^d, H-2^d, H-3^d, H-4^d, H-5^d, H-6a^d, H-6b^d, H-1^e, H-2^e, H-3^e, H-4^e, H-5^e, H-6a^e, H-6b^e, H-4^f, H-5^f, H-6^f, H-7^f, H-8^f, H-9a^f, H-9b^f, H-1a^{Cer}, H-1b^{Cer}, H-2^{Cer}, H-3^{Cer}, NHCH_2, 3 NC$H_2^{ATTO594}$, 2 CH_2S$O_3^{ATTO594}$), 2.85 (m, 1 H, H-3*eq*^{f}), 2.71 and 2.66 (2 s, 3 H, NC$H_3^{ATTO594}$), 2.18–2.15 (m, 2 H, NHC(=O)CH_2^{Cer}), 2.04–1.96 (m, 8 H, H-6a^{Cer}, H-6b^{Cer}, 2 Ac), 1.83–1.29 (m, 75 H, H-3*ax*^{f}, 26 CH_2^{Cer}, NCH_2C$H_2^{ATTO594}$, NCH_2CH_2C$H_2^{ATTO594}$, 2 NCH_2C$H_3^{ATTO594}$, 4 Me^{ATTO594}), 0.91–0.88 (m, 6 H, 2 Me^{Cer}); ^{13}C NMR (200 MHz, CD_3OD) δ 176.0, 175.9, 175.3, 175.3, 175.0, 175.0, 175.0, 171.2, 159.2, 159.1, 154.9, 154.2, 138.4, 137.7, 135.1, 132.2, 131.8, 131.4, 131.2, 130.7, 128.9, 126.2, 126.1, 124.2, 124.2, 122.9, 122.9, 115.0, 106.1, 106.1, 105.5, 104.4, 104.4, 102.8, 101.3, 97.2, 97.1, 81.3, 81.2, 81.1, 80.5, 80.0, 77.6, 76.8, 76.5, 76.4, 76.4, 76.2, 75.0, 74.8, 74.7, 73.0, 72.6, 72.5, 71.7, 70.8, 70.8, 70.7, 70.6, 69.9, 69.5, 69.5, 69.4, 69.4, 69.1, 63.0, 62.9, 62.8, 62.7, 61.8, 61.6, 54.7, 54.1, 54.0, 53.9, 53.9, 53.8, 53.2, 47.7, 43.7, 42.0, 41.4, 38.8, 37.4, 33.8, 33.6, 33.2, 33.1, 32.7, 31.0, 30.9, 30.9, 30.9, 30.8, 30.8, 30.7, 30.6, 30.6, 30.5, 29.5, 29.5, 29.4, 27.3, 23.8, 23.8, 23.6, 23.6, 22.8, 22.7, 14.5, 14.5, 13.9, 13.8; HRMS (ESI) *m/z*: found [(M–H)/2]$^-$ 1246.0952, $C_{120}H_{186}N_7O_{44}S_2^-$ calcd for [(M–H)/2]$^-$ 1246.0953.

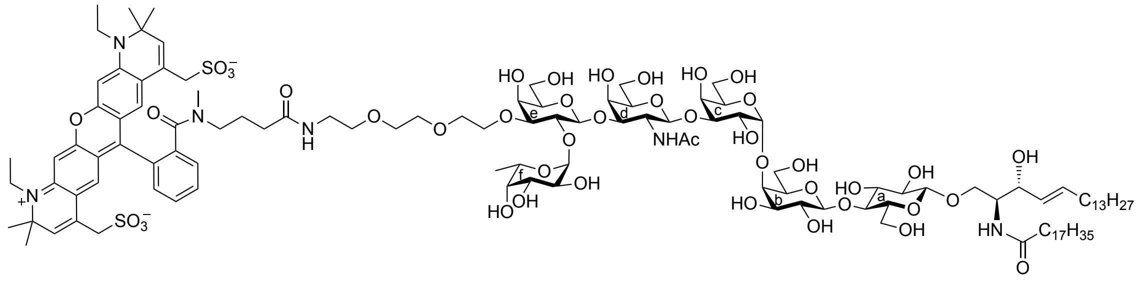

ATTO594 Globo-H (3)

To a solution of compound **50** (2.3 mg, 1.36 µmol) in DMF/H_2O = 11/1 (0.19 mL) were added ATTO594 NHS ester (3.9 mg, 2.83 µmol) and Et_3N (5.20 µL, 37.8 µmol). After stirring for 1.5 h at ambient temperature as the progress of the reaction was monitored by TLC ($CHCl_3$/MeOH/H_2O/AcOH = 5/4/1/0.2), the reaction mixture was purified by gel filtration (LH20; $CHCl_3$/MeOH = 1/1) and PTLC ($CHCl_3$/MeOH/H_2O = 5/4/1) to give compound **3** (2.3 mg, 68%) as a blue syrup; ^{1}H NMR (500 MHz, CD_3OD) δ 7.74–5.86 (m, 9 H, $Ar^{ATTO594}$), 5.68 (near quin, 1 H, $J_{5,6a}$ = $J_{5,6b}$ = 7.0 Hz, $J_{4,5}$ = 15.2 Hz, H-5^{Cer}), 5.44 (dd, 1 H, $J_{3,4}$ = 7.8 Hz, H-4^{Cer}), 5.31–3.14 (m, 66 H, H-1^a, H-2^a, H-3^a, H-4^a, H-5^a, H-$6a^a$, H-$6b^a$, H-1^b, H-2^b, H-3^b, H-4^b, H-5^b, H-$6a^b$, H-$6b^b$, H-1^c, H-2^c, H-3^c, H-4^c, H-5^c, H-$6a^c$, H-$6b^c$, H-1^d, H-2^d, H-3^d, H-4^d, H-5^d, H-$6a^d$, H-$6b^d$, H-1^e, H-2^e, H-3^e, H-4^e, H-5^e, H-$6a^e$, H-$6b^e$, H-1^f, H-2^f, H-3^f, H-4^f, H-5^f, H-$1a^{Cer}$, H-$1b^{Cer}$, H-2^{Cer}, H-3^{Cer}, 5 OCH_2, NHCH_2, 3 $NCH_2^{ATTO594}$, 2 $CH_2SO_3^{ATTO594}$), 2.72 and 2.64 (2 s, 3 H, $NCH_3^{ATTO594}$), 2.18–2.13 (m, 2 H, NHC(=O)CH_2^{Cer}), 2.03–1.16 (m, 82 H, H-6^f, H-$6a^{Cer}$, H-$6b^{Cer}$, Ac, 26 CH_2^{Cer}, $NCH_2CH_2^{ATTO594}$, $NCH_2CH_2CH_2^{ATTO594}$, 2 $NCH_2CH_3^{ATTO594}$, 4 $Me^{ATTO594}$), 0.91–0.88 (m, 6 H, 2 Me^{Cer}); ^{13}C NMR (200 MHz, CD_3OD) δ 176.0, 175.1, 174.4, 171.2, 159.3, 154.2, 154.2, 138.4, 138.4, 137.6, 135.1, 131.4, 131.2, 130.8, 128.9, 126.1, 124.3, 124.2, 123.0, 123.0, 115.1, 115.1, 105.6, 105.5, 104.5, 104.1, 102.9, 100.7, 97.1, 97.0, 84.6, 81.3, 80.6, 80.2, 79.1, 76.6, 76.5, 76.3, 75.2, 75.0, 74.8, 73.9, 73.6, 73.1, 72.7, 72.6, 71.7, 71.4, 71.1, 70.7, 70.5, 70.2, 70.0, 69.9, 69.7, 69.7, 67.8, 66.7, 64.5, 62.7, 62.7, 61.9, 61.8, 61.7, 61.7, 61.6, 54.8, 53.9, 53.2, 48.3, 47.7, 41.4, 40.5, 39.3, 38.8, 37.4, 33.9, 33.5, 33.1, 33.1, 33.1, 30.9, 30.9, 30.9, 30.9, 30.8, 30.8, 30.8, 30.7, 30.7, 30.7, 30.5, 30.5, 30.5, 30.5, 29.5, 29.5, 29.3, 29.3, 27.9, 27.2, 24.2, 23.8, 23.6, 21.6, 16.7, 14.5, 14.4, 13.8, 13.8; HRMS (ESI) *m/z*: found [(M–H)/2]$^-$ 1239.6158, $C_{121}H_{191}N_6O_{43}S_2^-$ calcd for [(M–H)/2]$^-$ 1239.6159.

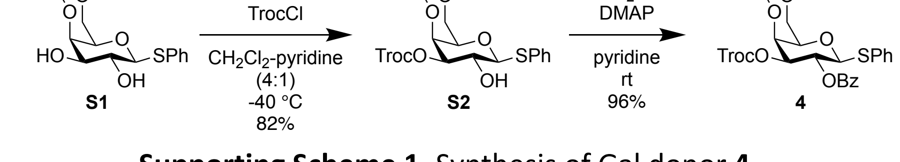

Supporting Scheme 1. Synthesis of Gal donor **4**.

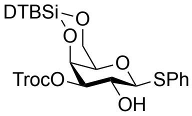

Compound S2

To a solution of compound **S1**[S01] (1.45 g, 3.51 mmol) in CH_2Cl_2/pyridine = 4/1 (35 mL) was added TrocCl (566 µL, 4.22 mmol) at –40 °C. After stirring for 30 min at –40 °C as the progress of the reaction was monitored by TLC (*n*-hexane/EtOAc = 2/1), the reaction mixture was co-evaporated with toluene and diluted with $CHCl_3$. The organic layer was washed with 2 M HCl, H_2O, satd. aq. $NaHCO_3$ and brine, dried over Na_2SO_4, and concentrated. The residue was purified by silica gel column chromatography (*n*-hexane/EtOAc = 12/1 → 5/1) to give compound **S2** (1.68 g, 82%) as a white foam; $[\alpha]_D$ –1.8° (c 1.0, $CHCl_3$); ^{1}H NMR (500 MHz, $CDCl_3$) δ 7.57–7.29 (m, 5 H, Ph), 4.93 (d, 1 H, J_{gem} = 11.8 Hz, C(=O)OCH_2CCl_3), 4.79 (d, 1 H, $J_{3,4}$ = 3.0 Hz, H-4), 4.71 (d, 1 H, C(=O)OCH_2CCl_3), 4.63–4.58 (m, 2 H, H-1, H-3), 4.30–4.23 (m, 2 H, H-6a, H-6b), 4.13 (td, 1 H, $J_{2,OH}$ = 2.6 Hz, $J_{1,2}$ = $J_{2,3}$ = 9.6 Hz, H-2), 3.50 (br s, 1 H, H-5), 2.54 (d, 1 H, OH-2), 1.04 (s, 9 H, ^{t}Bu), 1.00 (s, 9 H, ^{t}Bu); ^{13}C NMR (125 MHz, $CDCl_3$) δ 153.5, 132.8, 129.1, 128.2, 94.3, 89.9, 81.4, 76.9, 74.9, 69.7, 67.1, 67.1, 27.5, 27.4, 23.3, 20.6; HRMS (ESI) *m*/*z*: found $[M+Na]^+$ 609.0675, $C_{23}H_{33}Cl_3O_7SSi$ calcd for $[M+Na]^+$ 609.0674.

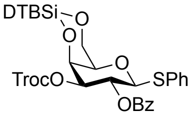

Compound 4

To a solution of compound **S2** (4.42 g, 7.52 mmol) in pyridine (75 mL) were added Bz_2O (2.56 g, 11.3 mmol) and DMAP (91.9 mg, 0.752 mmol) at 0 °C. After stirring for 20 h at ambient temperature as the progress of the reaction was monitored by TLC (*n*-hexane/EtOAc = 3/1), the reaction was quenched with MeOH, and the mixture was co-evaporated with toluene, and diluted with $CHCl_3$. The organic layer was washed with 2 M HCl, H_2O, satd. aq. $NaHCO_3$ and brine, dried over Na_2SO_4, and concentrated. The residue was purified by silica gel column chromatography (*n*-hexane/EtOAc = 8/1) to give compound **4** (5.01 g, 96%) as a white foam; $[\alpha]_D$ +62.5° (c 1.0, $CHCl_3$); ^{1}H NMR (500 MHz, $CDCl_3$) δ 8.05–7.22 (m, 10 H, Ph), 5.79 (t, 1 H, $J_{1,2}$ = $J_{2,3}$ = 9.9 Hz, H-2), 4.95 (dd, 1 H, $J_{3,4}$ = 3.0 Hz, H-3), 4.89 (d, 1 H, H-1), 4.80 (d, 1 H, H-4), 4.75 (d, 1 H, J_{gem} = 12.0 Hz, C(=O)OCH_2CCl_3), 4.65 (d, 1 H, C(=O)OCH_2CCl_3), 4.33–4.27 (m, 2 H, H-6a, H-6b), 3.57 (br s, 1 H, H-5), 1.15 (s, 9 H, ^{t}Bu), 1.03 (s, 9 H, ^{t}Bu); ^{13}C NMR (125 MHz, $CDCl_3$) δ 165.2, 153.7, 133.8, 133.4, 132.5, 129.9, 129.5, 128.9, 128.4, 127.9, 94.2, 87.6, 79.2, 76.8, 74.8, 70.3, 67.9, 67.0, 27.5, 27.5, 23.3, 20.7; HRMS (ESI) *m*/*z*: found $[M+Na]^+$

713.0938, $C_{30}H_{37}Cl_3O_8SSi$ calcd for $[M+Na]^+$ 713.0936.

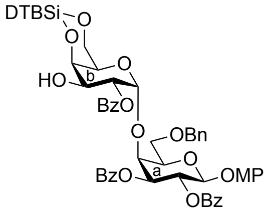

Compound 7

To a solution of donor **4** (2.74 g, 3.96 mmol) and acceptor **5**[S02] (1.34 g, 2.64 mmol) in CH_2Cl_2 (66 mL) were added NIS (1.78 g, 7.92 mmol) and 4 Å molecular sieves (6.50 g). After stirring for 1 h at ambient temperature, TfOH (210 µL, 2.38 mmol) was added at 0 °C. After stirring for 3 h at 0 °C as the progress of the reaction was monitored by TLC (toluene/acetone = 5/2), the reaction mixture was neutralized with satd. aq. $NaHCO_3$ and filtered through a pad of Celite. The pad was washed with $CHCl_3$. The combined filtrate and washings were diluted with $CHCl_3$ and washed with satd. aq. $Na_2S_2O_3$ and brine. The organic layer was dried over Na_2SO_4 and concentrated. The residue was purified by silica gel column chromatography (toluene/EtOAc = 90/1) to obtain crude mixture of **6**, which was dissolved in AcOH (30 mL) and zinc powder (15.4 g, 236 mmol) was added. After stirring for 2.5 h at ambient temperature as the progress of the reaction was monitored by TLC (toluene/EtOAc = 12/1), the reaction mixture was filtered through a pad of Celite. The pad was washed with $CHCl_3$. The combined filtrate and washings were co-evaporated with toluene and diluted with $CHCl_3$. The organic layer was washed with satd. aq. Na_2CO_3 and brine, dried over Na_2SO_4, and concentrated. The residue was purified by silica gel column chromatography (toluene/EtOAc = 20/1 → 6/1) to give compound **7** (2.04 g, 78%) as a white foam; $[\alpha]_D$ +80.5° (c 1.0, $CHCl_3$); ^1H NMR (500 MHz, $CDCl_3$) δ 8.20–6.76 (m, 24 H, 5 Ar), 5.98 (dd, 1 H, $J_{1,2}$ = 7.7 Hz, $J_{2,3}$ = 10.7 Hz, H-2^a), 5.44 (dd, 1 H, $J_{1,2}$ = 3.5 Hz, $J_{2,3}$ = 10.5 Hz, H-2^b), 5.31 (dd, 1 H, $J_{3,4}$ = 2.8 Hz, H-3^a), 5.16–5.14 (m, 2 H, H-1^a, H-1^b), 4.49 (d, 1 H, H-4^a), 4.46 (d, 1 H, $J_{3,4}$ = 3.0 Hz, H-4^b), 4.27 (td, 1 H, $J_{3,OH}$ = 11.5 Hz, H-3^b), 4.22 (d, 1 H, J_{gem} = 11.0 Hz, PhC*H*$_2$), 4.16 (d, 1 H, PhC*H*$_2$), 4.15 (br s, 1 H, H-5^b), 3.95 (near t, 1 H, H-5^a), 3.76 (s, 3 H, OMe), 3.69 (dd, 1 H, $J_{5,6a}$ = 7.5 Hz, J_{gem} = 9.4 Hz, H-6a^a), 3.54 (dd, 1 H, $J_{5,6b}$ = 6.3 Hz, H-6b^a), 3.37 (m, 2 H, H-6a^b, H-6b^b), 2.49 (d, 1 H, OH-3^b), 1.05 (s, 9 H, ^{t}Bu), 0.99 (s, 9 H, ^{t}Bu); ^{13}C NMR (125 MHz, $CDCl_3$) δ 166.6, 165.7, 165.5, 155.7, 151.1, 137.2, 133.7, 133.3, 133.2, 130.3, 129.8, 129.7, 129.4, 129.0, 129.0, 128.7, 128.4, 128.4, 128.2, 128.0, 127.9, 119.2, 114.5, 101.0, 98.0, 77.2, 73.8, 73.8, 73.7, 73.4, 73.3, 71.1, 69.5, 68.6, 67.7, 67.2, 66.2, 55.6, 27.5, 27.3, 23.3, 20.7; HRMS (ESI) *m/z*: found $[M+Na]^+$ 1013.3752, $C_{55}H_{62}O_{15}Si$ calcd for $[M+Na]^+$ 1013.3750.

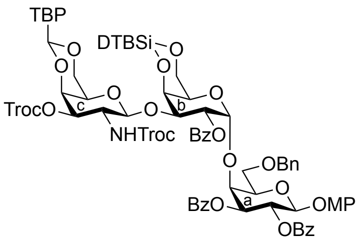

Compound 9

To a solution of donor **8**[S03] (2.98 g, 3.89 mmol) and acceptor **7** (3.86 g, 3.89 mmol) in CH_2Cl_2 (78 mL) were added NIS (1.05 g, 4.67 mmol) and 4 Å molecular sieves (7.80 g). After stirring for 1 h at ambient temperature, TfOH (3.4 µL, 38.9 µmol) was added at –40 °C. After stirring for 2.5 h at –40 °C as the progress of the reaction was monitored by TLC (toluene/acetone = 20/1), the reaction mixture was neutralized with satd. aq. $NaHCO_3$ and filtered through a pad of Celite. The pad was washed with $CHCl_3$. The combined filtrate and washings were diluted with $CHCl_3$ and washed with satd. aq. $Na_2S_2O_3$ and brine. The organic layer was dried over Na_2SO_4 and concentrated. The residue was purified by silica gel column chromatography (toluene/EtOAc = 100/1) to give compound **9** (6.04 g, 94%) as a white foam; $[\alpha]_D$ +107.3° (c 1.0, $CHCl_3$); ^{1}H NMR (500 MHz, acetone-d_6) δ 8.24–6.79 (m, 29 H, 6 Ar, NH-2^c), 5.97 (dd, 1 H, $J_{1,2}$ = 7.9 Hz, $J_{2,3}$ = 10.8 Hz, H-2^a), 5.80 (dd, 1 H, $J_{1,2}$ = 3.9 Hz, $J_{2,3}$ = 10.8 Hz, H-2^b), 5.64 (s, 1 H, ArC*H*<), 5.56 (dd, 1 H, $J_{3,4}$ = 2.8 Hz, H-3^a), 5.49 (d, 1 H, H-1^a), 5.35 (d, 1 H, H-1^b), 5.32 (d, 1 H, $J_{1,2}$ = 8.0 Hz, H-1^c), 5.21 (dd, 1 H, $J_{3,4}$ = 3.5 Hz, $J_{2,3}$ = 11.5 Hz, H-3^c), 5.10 (d, 1 H, $J_{3,4}$ = 2.4 Hz, H-4^b), 4.87 (d, 1 H, J_{gem} = 12.0 Hz, C(=O)OCH_2CCl_3), 4.80 (d, 1 H, C(=O)OCH_2CCl_3), 4.61 (d, 1 H, H-4^c), 4.54 (d, 1 H, H-4^a), 4.51 (dd, 1 H, H-3^b), 4.36 (d, 1 H, J_{gem} = 12.5 Hz, H-6a^c), 4.29 (t, 1 H, $J_{5,6a}$ = $J_{5,6b}$ = 6.8 Hz, H-5^a), 4.26–4.21 (m, 4 H, H-5^b, H-6b^c, PhCH_2, C(=O)OCH_2CCl_3), 4.15 (d, 1 H, J_{gem} = 11.5 Hz, PhCH_2), 4.06 (near q, 1 H, $J_{2,NH}$ = 8.5 Hz, H-2^c), 3.91 (s, 1 H, H-5^c), 3.74–3.72 (m, 4 H, OMe, C(=O)OCH_2CCl_3), 3.64–3.54 (m, 3 H, H-6a^a, H-6b^a, H-6a^b), 3.48 (dd, 1 H, $J_{5,6b}$ = 0.8 Hz, J_{gem} = 12.3 Hz, H-6b^b), 1.31 (s, 9 H, ^{t}Bu), 1.08 (s, 9 H, ^{t}Bu), 1.06 (s, 9 H, ^{t}Bu); ^{13}C NMR (125 MHz, acetone-d_6) δ 166.6, 166.2, 156.6, 154.7, 154.2, 152.4, 152.2, 139.1, 136.8, 134.6, 134.3, 134.1, 131.3, 130.9, 130.6, 130.3, 130.2, 129.6, 129.5, 129.5, 129.1, 128.5, 128.4, 127.3, 125.5, 119.5, 115.3, 103.2, 101.6, 101.3, 98.8, 97.0, 95.5, 79.2, 78.3, 77.4, 76.7, 74.9, 74.7, 74.6, 74.4, 73.8, 73.5, 73.4, 70.8, 70.0, 69.6, 69.0, 68.8, 67.5, 67.0, 55.8, 53.2, 35.1, 31.6, 28.1, 28.0, 24.0, 21.3; HRMS (ESI) *m/z*: found $[M+Na]^+$ 1666.3463, $C_{78}H_{87}Cl_6NO_{23}Si$ calcd for $[M+Na]^+$ 1666.3462.

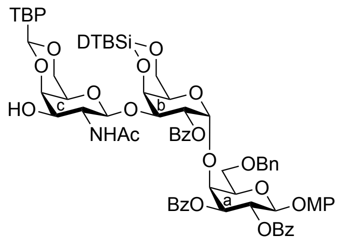

Compound 10

To a solution of compound **9** (100 mg, 60.7 μmol) in AcOH (2.5 mL) was added zinc powder (500 mg, 7.64 mmol). After stirring for 1 h at ambient temperature as the progress of the reaction was monitored by TLC ($CHCl_3$/MeOH = 20/1), the reaction mixture was filtered through a pad of Celite. The pad was washed with $CHCl_3$. The combined filtrate and washings were diluted with $CHCl_3$ and washed with satd. aq. $Na_2S_2O_3$ and brine. The organic layer was dried over Na_2SO_4, concentrated, and dried for 2 h. The residue was dissolved in CH_2Cl_2 (5.0 mL), followed by the addition of Ac_2O (8.60 μL, 91.1 μmol). After stirring for 1 h at ambient temperature as the progress of the reaction was monitored by TLC ($CHCl_3$/MeOH = 20/1), the reaction mixture was concentrated. The residue was purified by silica gel column chromatography ($CHCl_3$/EtOAc = 20/1) to give compound **10** (69.9 mg, 86%) as a white foam; $[\alpha]_D$ +101.8° (c 1.0, $CHCl_3$); ^{1}H NMR (500 MHz, $CDCl_3$) δ 8.21–6.75 (m, 28 H, 6 Ar), 6.03 (dd, 1 H, $J_{1,2}$ = 8.0 Hz, $J_{2,3}$ = 10.9 Hz, H-2^a), 5.74 (dd, 1 H, $J_{1,2}$ = 4.0 Hz, $J_{2,3}$ = 10.5 Hz, H-2^b), 5.59 (d, 1 H, $J_{2,NH}$ = 3.5 Hz, NH-2^c), 5.55 (s, 1 H, ArC*H*<), 5.35 (dd, 1 H, $J_{3,4}$ = 2.9 Hz, H-3^a), 5.20 (d, 1 H, H-1^b), 5.11 (d, 1 H, H-1^a), 4.81 (d, 1 H, $J_{3,OH}$ = 5.5 Hz, OH-3^c), 4.76 (d, 1 H, $J_{1,2}$ = 7.5 Hz, H-1^c), 4.74 (d, 1 H, $J_{3,4}$ = 2.5 Hz, H-4^b), 4.51 (d, 1 H, H-4^a), 4.41 (br d, 1 H, J_{gem} = 11.5 Hz, H-6a^c), 4.29 (dd, 1 H, H-3^b), 4.18–4.15 (m, 2 H, H-4^c, PhC*H*$_2$), 4.12 (d, 1 H, J_{gem} = 11.0 Hz, PhC*H*$_2$), 4.11 (br d, 1 H, H-6b^c), 4.04 (br s, 1 H, H-5^b), 3.94 (near t, 1 H, H-5^a), 3.88–3.80 (m, 2 H, H-2^c, H-3^c), 3.76 (s, 3 H, OMe), 3.63 (br s, 1 H, H-5^c), 3.57 (dd, 1 H, $J_{5,6a}$ = 7.3 Hz, J_{gem} = 9.3 Hz, H-6a^a), 3.48 (dd, 1 H, $J_{5,6b}$ = 6.8 Hz, H-6b^a), 3.34–3.28 (m, 2 H, H-6a^b, H-6b^b), 1.41 (s, 3 H, Ac), 1.31 (s, 9 H, ^{t}Bu), 1.06 (s, 9 H, ^{t}Bu), 0.99 (s, 9 H, ^{t}Bu); ^{13}C NMR (125 MHz, $CDCl_3$) δ 172.8, 166.1, 165.8, 165.7, 155.7, 152.0, 151.3, 137.2, 135.1, 133.8, 133.8, 133.4, 130.2, 129.7, 129.7, 129.3, 129.2, 128.9, 128.8, 128.5, 128.4, 128.0, 127.9, 126.4, 125.0, 119.1, 114.5, 101.9, 101.5, 101.5, 97.7, 76.3, 75.0, 73.8, 73.6, 73.5, 73.3, 73.0, 71.9, 69.7, 69.5, 69.1, 68.0, 67.2, 66.9, 66.4, 55.7, 55.6, 34.6, 31.3, 27.5, 23.4, 22.6, 20.6; HRMS (ESI) *m/z*: found $[M+Na]^+$ 1360.5485, $C_{74}H_{87}NO_{20}Si$ calcd for $[M+Na]^+$ 1360.5483.

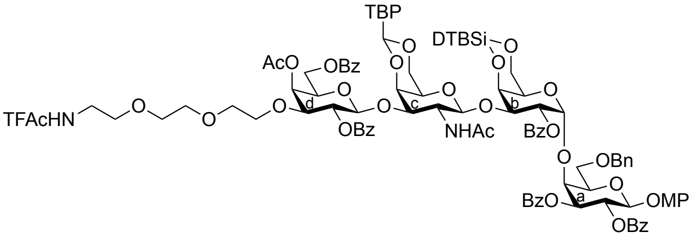

Compound 12

To a solution of donor **11** (208 mg, 0.259 mmol) and acceptor **10** (347 mg, 0.259 mmol) in CH_2Cl_2 (5.2 mL) was added 4 Å molecular sieves (AW-300, 700 mg). After stirring for 1 h at ambient temperature, TMSOTf (4.7 µL, 25.9 µmol) was added at 0 °C. After stirring for 4 h at 0 °C as the progress of the reaction was monitored by TLC (toluene/$CHCl_3$/EtOAc = 3/1/1), the reaction mixture was neutralized with satd. aq. $NaHCO_3$ and filtered through a pad of Celite. The pad was washed with $CHCl_3$. The combined filtrate and washings were diluted with $CHCl_3$ and washed with brine. The organic layer was dried over Na_2SO_4 and concentrated. The residue was purified by silica gel column chromatography (toluene/$CHCl_3$/EtOAc = 5/1/1) to give compound **12** (432 mg, 84%) as a white amorphous solid; $[\alpha]_D$ +61.5° (c 1.0, $CHCl_3$); ^{1}H NMR (500 MHz, $CDCl_3$) δ 8.08–6.72 (m, 39 H, 8 Ar, $CF_3C(=O)NH$), 5.92 (dd, 1 H, $J_{1,2}$ = 8.0 Hz, $J_{2,3}$ = 11.0 Hz, H-2^a), 5.58–5.55 (m, 2 H, H-2^b, H-4^d), 5.41 (s, 1 H, ArC*H*<), 5.34–5.28 (m, 3 H, H-3^a, H-2^d, NH-2^c), 5.23 (d, 1 H, $J_{1,2}$ = 8.5 Hz, H-1^c), 5.15 (d, 1 H, $J_{1,2}$ = 3.8 Hz, H-1^b), 5.08 (d, 1 H, H-1^a), 4.84 (dd, 1 H, $J_{3,4}$ = 3.3 Hz, $J_{2,3}$ = 11.3 Hz, H-3^c), 4.72 (d, 1 H, $J_{1,2}$ = 8.0 Hz, H-1^d), 4.68 (d, 1 H, $J_{3,4}$ = 2.8 Hz, H-4^b), 4.60 (dd, 1 H, $J_{5,6a}$ = 7.7 Hz, J_{gem} = 11.5 Hz, H-6a^d), 4.37 (d, 1 H, $J_{3,4}$ = 3.0 Hz, H-4^a), 4.30–4.27 (m, 2 H, H-4^c, H-6b^d), 4.22 (dd, 1 H, $J_{2,3}$ = 10.5 Hz, H-3^b), 4.18 (br d, 1 H, J_{gem} = 11.3 Hz, H-6a^c), 4.08–4.03 (m, 3 H, H-5^b, 2 PhCH_2), 4.01 (dd, 1 H, $J_{5,6b}$ = 4.9 Hz, H-5^d), 3.88 (t, 1 H, $J_{5,6a}$ = $J_{5,6b}$ = 6.5 Hz, H-5^a), 3.75 (s, 3 H, OMe), 3.71–3.67 (m, 2 H, H-3^d, OCH_2), 3.63 (br d, 1 H, H-6b^c), 3.51–3.25 (m, 17 H, H-6a^a, H-6b^a, H-6a^b, H-6b^b, H-2^c, H-5^c, 9 OCH_2, 2 NHCH_2), 2.20 (s, 3 H, Ac), 1.33 (s, 9 H, ^{t}Bu), 0.99 (s, 9 H, ^{t}Bu), 0.89 (s, 9 H, ^{t}Bu), 0.66 (s, 3 H, Ac); ^{13}C NMR (125 MHz, $CDCl_3$) δ 171.0, 170.7, 166.0, 166.0, 165.9, 165.5, 164.9, 157.4, 157.1, 155.6, 151.5, 151.3, 137.5, 135.5, 133.6, 133.6, 133.3, 133.3, 133.1, 130.3, 129.9, 129.8, 129.7, 129.6, 129.4, 129.4, 128.9, 128.7, 128.5, 128.4, 128.4, 128.3, 127.7, 126.3, 124.9, 118.9,117.1, 114.8, 114.4, 102.5, 101.2, 100.8, 99.6, 98.0, 78.9, 76.0, 74.9, 74.1, 74.0, 73.9, 73.5, 73.2, 71.3, 71.0, 70.8, 70.5, 70.2, 70.1, 69.6, 69.4, 69.0, 68.6, 68.1, 68.0, 66.7, 66.5, 66.2, 62.9, 55.6, 54.9, 39.6, 34.6, 31.3, 27.5, 27.3, 23.3, 22.4, 20.9, 20.6; HRMS (ESI) *m/z*: found $[M+Na]^+$ 1999.7412, $C_{104}H_{119}F_3N_2O_{31}Si$ calcd for $[M+Na]^+$ 1999.7410.

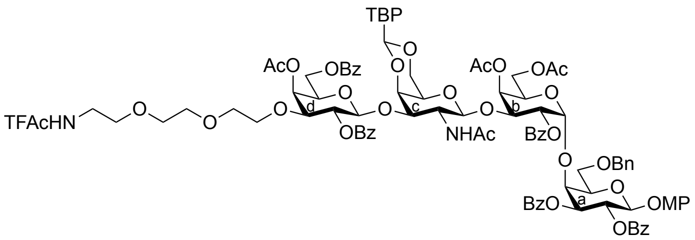

Compound 13

To a solution of compound **12** (893 mg, 0.451 mmol) in THF (4.5 mL) was added 1 M TBAHF in THF (4.51 mL, 4.51 mmol). After stirring for 14 h at ambient temperature as the progress of the reaction was monitored by TLC (*n*-hexane/acetone = 1/1), the reaction mixture was diluted with $CHCl_3$ and washed with 2 M HCl, H_2O, satd. aq. $NaHCO_3$ and brine. The organic layer was dried over Na_2SO_4, concentrated and dried for 2 h. The residue was dissolved in pyridine (4.5 mL), followed by the addition of Ac_2O (170 µL, 1.80 mmol) and DMAP (5.5 mg, 45.1 µmol) at 0 °C. After stirring for 20 h at ambient temperature as the progress of the reaction was monitored by TLC (*n*-hexane/acetone = 1/1), the reaction was quenched with MeOH at 0 °C and diluted with $CHCl_3$. The organic layer was washed with 2 M HCl, H_2O, satd. aq. $NaHCO_3$ and brine, dried over Na_2SO_4, and concentrated. The residue was purified by silica gel column chromatography (*n*-hexane/acetone = 4/3) to give compound **13** (736 mg, 85%) as a white amorphous solid; $[\alpha]_D$ +60.3° (c 1.0, $CHCl_3$); ^{1}H NMR (500 MHz, $CDCl_3$) δ 8.09–6.73 (m, 39 H, 8 Ar, $CF_3C(=O)NH$), 6.01 (dd, 1 H, $J_{1,2}$ = 7.7 Hz, $J_{2,3}$ = 10.8 Hz, H-2^a), 5.56 (d, 1 H, $J_{3,4}$ = 3.2 Hz, H-4^d), 5.49 (dd, 1 H, $J_{1,2}$ = 4.0 Hz, $J_{2,3}$ = 10.5 Hz, H-2^b), 5.44 (d, 1 H, $J_{3,4}$ = 2.5 Hz, H-4^b), 5.36–5.33 (m, 2 H, H-3^a, Ar*CH*<), 5.31 (dd, 1 H, $J_{1,2}$ = 8.3 Hz, $J_{2,3}$ = 9.8 Hz, H-2^d), 5.22 (d, 1 H, $J_{1,2}$ = 8.0 Hz, H-1^c), 5.19 (d, 1 H, H-1^b), 5.09 (d, 1 H, H-1^a), 5.01 (d, 1 H, $J_{2,NH}$ = 6.5 Hz, NH-2^c), 4.72–4.69 (m, 2 H, H-3^c, H-1^d), 4.58 (dd, 1 H, $J_{5,6a}$ = 7.4 Hz, J_{gem} = 11.4 Hz, H-6a^d), 4.50 (d, 1 H, $J_{3,4}$ = 3.0 Hz, H-4^a), 4.47–4.44 (m, 2 H, H-3^b, H-4^c), 4.29 (dd, 1 H, $J_{5,6b}$ = 5.3 Hz, H-6b^d), 4.25–4.22 (m, 2 H, H-5^b, PhCH_2), 4.19 (br d, 1 H, J_{gem} = 11.5 Hz, H-6a^c), 4.10 (d, 1 H, J_{gem-}= 12.0 Hz, PhCH_2), 4.04 (dd, 1 H, H-5^d), 3.90 (t, 1 H, $J_{5,6a}$ = $J_{5,6b}$ = 7.0 Hz, H-5^a), 3.76 (s, 3 H, OMe), 3.72-3.67 (m, 2 H, H-3^d, OCH_2), 3.58 (br d, 1 H, H-6b^c), 3.52–3.27 (m, 16 H, H-6a^a, H-6b^a, H-6a^b, H-6b^b, H-5^c, 9 OCH_2, 2 NHCH_2), 3.19 (m, 1 H, H-2^c), 2.19 (s, 3 H, Ac), 2.04 (s, 3 H, Ac), 1.79 (s, 3 H, Ac), 1.31 (s, 9 H, ^{t}Bu), 0.94 (s, 3 H, Ac); ^{13}C NMR (125 MHz, $CDCl_3$) δ 171.1, 170.7, 170.1, 169.9, 166.0, 165.9, 165.8, 165.4, 164.9, 157.4, 157.1, 155.6, 151.7, 151.3, 137.3, 135.5, 133.6, 133.6, 133.3, 133.1, 130.2, 129.9, 129.7, 129.7, 129.6, 129.5, 129.4, 128.8, 128.7, 128.4, 128.4, 127.8, 127.7, 126.4, 125.0, 119.0, 117.1, 114.5, 102.5, 101.1, 101.0, 98.4, 96.8, 78.8, 76.2, 75.8, 73.7, 73.5, 73.2, 72.8, 72.5, 71.3, 71.1, 70.9, 70.5, 70.2, 70.1, 69.7, 69.5, 68.8, 68.6, 67.2, 66.7, 66.5, 62.9, 61.5, 55.7, 54.7, 39.6, 34.6, 31.3, 29.3, 22.6, 20.9, 20.9, 20.6; HRMS (ESI) *m/z*: found $[M+Na]^+$ 1943.6601, $C_{100}H_{107}F_3N_2O_{33}$ calcd for $[M+Na]^+$ 1943.6600.

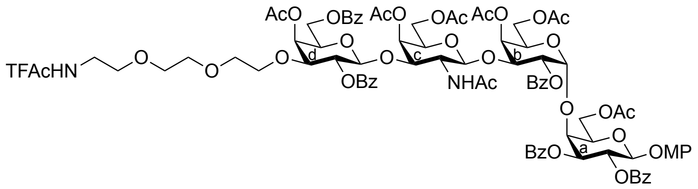

Compound 14

To a solution of compound **13** (736 mg, 0.383 mmol) in 1,4-dioxane/MeOH = 1/1 (19 mL) was added $Pd(OH)_2$-C (53.8 mg, 20% on carbon). After stirring for 16 h at ambient temperature under H_2 gas as the progress of the reaction was monitored by TLC ($CHCl_3$/MeOH =20/1), the reaction mixture was filtered through a pad of Celite, the pad was washed with $CHCl_3$, the combined filtrate and washings were concentrated, co-evaporated with toluene, and dried for 2 h. The residue was dissolved in pyridine (3.8 mL), followed by the addition of Ac_2O (145 µL, 1.53 mmol) and DMAP (4.7 mg, 38.3 µmol) at 0 °C. After stirring for 20 h at ambient temperature as the progress of the reaction was monitored by TLC ($CHCl_3$/MeOH =20/1), Ac_2O (145 µL, 1.53 mmol) was added to complete the reaction. After stirring for another 49 h, the reaction was quenched with MeOH at 0 °C, and the mixture was co-evaporated with toluene, and diluted with $CHCl_3$. The organic layer was washed with 2 M HCl, H_2O, satd. aq. $NaHCO_3$ and brine, dried over Na_2SO_4, and concentrated. The residue was purified by silica gel column chromatography (toluene/acetone = 3/1) to give compound **14** (607 mg, 87%) as a white amorphous solid; $[\alpha]_D$ +72.7° (c 1.0, $CHCl_3$); ^{1}H NMR (500 MHz, $CDCl_3$) δ 8.06–6.78 (m, 30 H, 6 Ar, CF_3C(=O)NH), 5.93 (dd, 1 H, $J_{1,2}$ = 7.8 Hz, $J_{2,3}$ = 10.5 Hz, H-2^a), 5.54 (m, 2 H, H-4^b, H-4^d), 5.48 (d, 1 H, $J_{3,4}$ = 3.5 Hz, H-4^c), 5.42 (dd, 1 H, $J_{1,2}$ = 3.5 Hz, $J_{2,3}$ = 10.5 Hz, H-2^b), 5.29 (dd, 1 H, $J_{3,4}$ = 3.0 Hz, H-3^a), 5.23 (dd, 1 H, $J_{1,2}$ = 7.9 Hz, $J_{2,3}$ = 9.9 Hz, H-2^d), 5.21 (d, 1 H, $J_{1,2}$ = 8.0 Hz, H-1^c), 5.17 (d, 1 H, H-1^b), 5.11 (d, 1 H, H-1^a), 5.08 (d, 1 H, $J_{2,NH}$ = 6.5 Hz, NH-2^c), 4.77 (dd, 1 H, $J_{2,3}$ = 11.0 Hz, H-3^c), 4.62 (d, 1 H, H-1^d), 4.50 (near t, 1 H, $J_{5,6b}$ = 6.0 Hz, $J_{5,6a}$ = 7.0 Hz, H-5^c), 4.47 (dd, 1 H, $J_{5,6a}$ = 6.8 Hz, J_{gem} = 11.4 Hz, H-6a^d), 4.43–4.40 (m, 2 H, H-3^b, H-6a^b), 4.35 (d, 1 H, H-4^a), 4.31 (dd, 1 H, $J_{5,6b}$ = 6.5 Hz, H-6b^d), 4.10 (dd, 1 H, $J_{5,6a}$ = 5.0 Hz, J_{gem} = 11.5 Hz, H-6a^a), 4.02–3.93 (m, 4 H, H-6b^a, H-5^b, H-6b^b, H-5^d), 3.87 (near t, 1 H, $J_{5,6b}$ = 7.0 Hz, H-5^a), 3.80–3.75 (m, 4 H, H-6a^c, OMe), 3.70 (m, 1 H, OCH_2), 3.65 (dd, 1 H, $J_{3,4}$ = 3.0 Hz, H-3^d), 3.53 (dd, 1 H, J_{gem} = 11.0 Hz, H-6b^c), 3.51–3.23 (m, 11 H, 9 OCH_2, 2 NHCH_2), 3.02 (m, 1 H, H-2^c), 2.16 (s, 3 H, Ac), 2.12 (s, 3 H, Ac), 2.07 (s, 3 H, Ac), 2.05 (s, 3 H, Ac), 1.88 (s, 3 H, Ac), 1.87 (s, 3 H, Ac), 0.73 (s, 3 H, Ac); ^{13}C NMR (125 MHz, $CDCl_3$) δ 170.8, 170.6, 170.4, 170.3, 170.0, 170.0, 169.1, 166.2, 166.1, 165.8, 165.2, 165.0, 157.4, 155.7, 151.1, 133.7, 133.6, 133.4, 133.3, 130.0, 129.9, 129.7, 129.7, 129.6, 129.3, 128.9, 128.7, 128.6, 128.6, 128.5, 128.5, 128.5, 118.8, 117.1, 114.5, 101.7, 101.0, 98.7, 98.4, 78.6, 77.6, 74.8, 74.6, 73.4, 72.4, 72.0, 71.5, 71.4, 70.8, 70.8, 70.4, 70.2, 70.2, 70.1, 70.0, 69.4, 69.2, 68.5, 67.7, 66.4, 62.6, 62.0, 61.3, 61.0, 55.7, 55.5, 39.6, 22.2, 20.8, 20.7, 20.6, 20.5;

HRMS (ESI) *m/z*: found $[M+Na]^+$ 1835.5513, $C_{88}H_{95}F_3N_2O_{36}$ calcd for $[M+Na]^+$ 1835.5509.

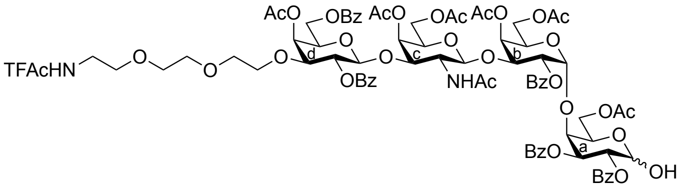

Compound 15

To a solution of compound **14** (595 mg, 0.328 mmol) in MeCN/toluene/H_2O = 6/5/3 (6.6 mL) was added cerium (IV) ammonium nitrate (1.80 g, 3.28 mmol) at 0 °C. After stirring for 4 h at 0 °C as the progress of the reaction was monitored by TLC ($CHCl_3$/MeOH = 30/1, developed twice), the reaction mixture was diluted with EtOAc. The organic layer was washed with H_2O, satd. aq. $NaHCO_3$ and brine, dried over Na_2SO_4, and concentrated. The residue was purified by silica gel column chromatography ($CHCl_3$/MeOH = 80/1) to give compound **15** (349 mg, 62%, α:β = 1:0.3) as a white amorphous solid; α isomer; ^1H NMR (500 MHz, $CDCl_3$) δ 8.06–7.33 (m, 25 H, 5 Ph), 7.17 (br s, 1 H, $CF_3C(=O)NH$), 5.75 (dd, 1 H, $J_{3,4}$ = 3.0 Hz, $J_{2,3}$ = 10.9 Hz, H-3^a), 5.71 (near t, 1 H, $J_{1,OH}$ = 3.0 Hz, $J_{1,2}$ = 3.4 Hz, H-1^a), 5.61 (dd, 1 H, H-2^a), 5.55 (m, 1 H, H-4^d), 5.51–5.49 (m, 2 H, H-4^b, H-4^c), 5.41 (dd, 1 H, $J_{1,2}$ = 3.4 Hz, $J_{2,3}$ = 10.3 Hz, H-2^b), 5.26–5.21 (m, 2 H, H-1^c, H-2^d), 5.19 (d, 1 H, H-1^b), 5.11 (d, 1 H, $J_{2,NH}$ = 6.5 Hz, NH-2^c), 4.76 (dd, 1 H, $J_{3,4}$ = 3.8 Hz, $J_{2,3}$ = 10.8 Hz, H-3^c), 4.62 (d, 1 H, $J_{1,2}$ = 7.5 Hz, H-1^d), 4.50–4.46 (m, 2 H, H-5^c, H-6a^d), 4.40–4.36 (m, 2 H, H-3^b, H-6a^b), 4.33–4.28 (m, 2 H, H-4^a, H-6b^d), 4.12 (dd, 1 H, $J_{5,6a}$ = 5.0 Hz, J_{gem} = 11.5 Hz, H-6a^a), 4.02–3.99 (m, 2 H, H-6b^a, H-6b^b), 3.96–3.94 (m, 2 H, H-5^b, H-5^d), 3.86 (near t, 1 H, H-5^a), 3.77–3.64 (m, 3 H, H-6a^c, H-3^d, OCH_2), 3.59 (dd, 1 H, $J_{5,6b}$ = 7.0 Hz, J_{gem} = 11.4 Hz, H-6b^c), 3.51–3.24 (m, 11 H, 9 OCH_2, 2 $NHCH_2$), 3.02 (m, 1 H, H-2^c), 2.80 (d, 1 H, OH-1^a), 2.17 (s, 3 H, Ac), 2.12 (s, 3 H, Ac), 2.06 (s, 3 H, Ac), 2.05 (s, 3 H, Ac), 1.87 (s, 3 H, Ac), 1.70 (s, 3 H, Ac), 0.72 (s, 3 H, Ac); HRMS (ESI) *m/z*: found $[M+Na]^+$ 1729.5090, $C_{81}H_{89}F_3N_2O_{35}$ calcd for $[M+Na]^+$ 1729.5090.

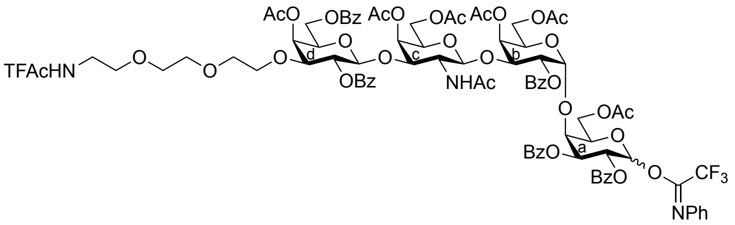

Compound 16

To a solution of compound **15** (32.7 mg, 19.1 µmol) in acetone (0.38 mL) were added $CF_3C(=NPh)Cl$ (6.2 µL, 38.3 µmol) and K_2CO_3 (13.2 mg, 95.7 µmol). After stirring for 6 h at ambient temperature as

the progress of the reaction was monitored by TLC ($CHCl_3$/MeOH = 20/1), the reaction mixture was concentrated. The residue was purified by silica gel column chromatography ($CHCl_3$/MeOH = 80/1) to give compound **16** (33.9 mg, 94%, α:β = 1:0.3) as a white amorphous solid; α isomer; ^{1}H NMR (500 MHz, $CDCl_3$) δ 8.06–6.48 (m, 31 H, 6 Ph, CF_3C(=O)NH), 6.05 (br s, 1 H, H-1^a), 5.87 (br dd, 1 H, H-2^a), 5.74 (br dd, 1 H, H-3^a), 5.55 (d, 1 H, $J_{3,4}$ = 3.5 Hz, H-4^d), 5.52 (d, 1 H, $J_{3,4}$ = 3.5 Hz, H-4^b), 5.49 (d, 1 H, $J_{3,4}$ = 3.3 Hz, H-4^c), 5.41 (dd, 1 H, $J_{1,2}$ = 4.0 Hz, $J_{2,3}$ = 10.5 Hz, H-2^b), 5.25–5.19 (m, 3 H, H-1^b, H-1^c, H-2^d), 5.13 (d, 1 H, $J_{2,NH}$ = 6.5 Hz, NH-2^c), 4.75 (dd, 1 H, $J_{2,3}$ = 11.0 Hz, H-3^c), 4.63 (d, 1 H, $J_{1,2}$ = 8.0 Hz, H-1^d), 4.50–4.46 (m, 2 H, H-5^c, H-6a^d), 4.42 (br s, 1 H, H-4^a), 4.39–4.36 (m, 2 H, H-3^b, H-5^b), 4.33–4.27 (m, 2 H, H-6a^b, H-6b^d), 4.12 (dd, 1 H, $J_{5,6a}$ = 5.5 Hz, J_{gem} = 11.5 Hz, H-6a^a), 4.04–3.97 (m, 2 H, H-6b^a, H-6b^b), 3.95 (near t, 1 H, H-5^d), 3.86 (near t, 1 H, H-5^a), 3.76 (dd, 1 H, $J_{5,6a}$ = 6.3 Hz, J_{gem} = 11.3 Hz, H-6a^c), 3.72–3.64 (m, 2 H, H-3^d, OCH_2), 3.61 (dd, 1 H, $J_{5,6b}$ = 6.5 Hz, H-6b^c), 3.50–3.24 (m, 11 H, 9 OCH_2, 2 NHC*H*$_2$), 3.03 (m, 1 H, H-2^c), 2.16 (s, 3 H, Ac), 2.12 (s, 3 H, Ac), 2.06 (s, 3 H, Ac), 2.05 (s, 3 H, Ac), 1.90 (s, 3 H, Ac), 1.75 (s, 3 H, Ac), 0.73 (s, 3 H, Ac); HRMS (ESI) *m/z*: found $[M+Na]^+$ 1900.5390, $C_{89}H_{93}F_6N_3O_{35}$ calcd for $[M+Na]^+$ 1900.5386.

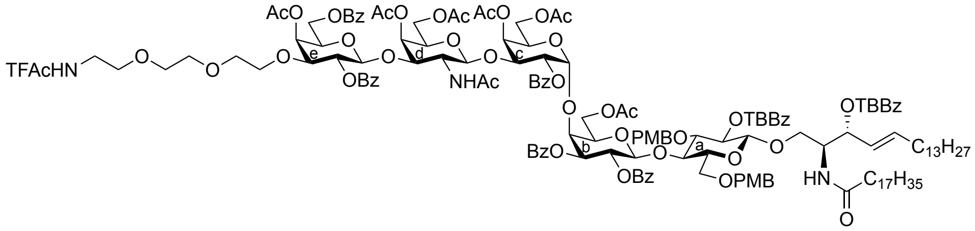

Compound 18

To a solution of donor **16** (50.0 mg, 26.6 μmol) and acceptor **17**[S03] (51.4 mg, 39.9 μmol) in CH_2Cl_2 (1.3 mL) was added 4 Å molecular sieves (AW-300, 130 mg). After stirring for 1 h at ambient temperature, TMSOTf (0.96 μL, 5.32 μmol) was added at 0 °C. After stirring for 3 h at 0 °C as the progress of the reaction was monitored by TLC (toluene/acetone = 3/1), the reaction mixture was neutralized with satd. aq. $NaHCO_3$ and filtered through a pad of Celite. The pad was washed with $CHCl_3$. The combined filtrate and washings were diluted with $CHCl_3$ and washed with brine. The organic layer was dried over Na_2SO_4 and concentrated. The residue was purified by silica gel column chromatography (toluene/acetone = 10/1 → 4/1) to give compound **18** (51.4 mg, 65%) as a white amorphous solid; $[α]_D$ +50.7° (c 1.9, $CHCl_3$); ^{1}H NMR (500 MHz, $CDCl_3$) δ 8.06–6.66 (m, 42 H, 9 Ar, CF_3C(=O)NH), 5.78 (near quin, 1 H, $J_{5,6a}$ = $J_{5,6b}$ = 6.5 Hz, $J_{4,5}$ = 15.5 Hz, H-5^{Cer}), 5.68–5.64 (m, 2 H, H-2^b, NH-2^{Cer}), 5.53 (d, 1 H, $J_{3,4}$ = 3.0 Hz, H-4^e), 5.46 (t, 1 H, $J_{2,3}$ = $J_{3,4}$ = 7.4 Hz, H-3^{Cer}), 5.44 (d, 1 H, $J_{3,4}$ = 2.0 Hz, H-4^c), 5.39 (dd, 1 H, H-4^{Cer}), 5.34–5.31 (m, 2 H, H-2^c, H-4^d), 5.19 (dd, 1 H, $J_{1,2}$ = 8.0 Hz, $J_{2,3}$ = 9.5 Hz, H-2^e), 5.17–5.13

(m, 2 H, H-2[a], H-1[c]), 5.09 (dd, 1 H, $J_{3,4}$ = 2.8 Hz, $J_{2,3}$ = 10.8 Hz, H-3[b]), 5.03 (d, 1 H, $J_{2,NH}$ = 7.0 Hz, NH-2[d]), 4.94 (d, 1 H, $J_{1,2}$ = 8.0 Hz, H-1[d]), 4.91 (d, 1 H, $J_{1,2}$ = 8.0 Hz, H-1[b]), 4.67 (d, 1 H, J_{gem} = 11.8 Hz, ArC*H*$_2$), 4.62 (d, 1 H, ArC*H*$_2$), 4.58 (d, 1 H, H-1[e]), 4.54 (dd, 1 H, $J_{3,4}$ = 3.5 Hz, $J_{2,3}$ = 11.0 Hz, H-3[d]), 4.48 (dd, 1 H, $J_{5,6a}$ = 6.5 Hz, J_{gem} = 11.0 Hz, H-6a[e]), 4.43–4.41 (m, 2 H, H-5[d], ArC*H*$_2$), 4.37–4.22 (m, 6 H, H-1[a], H-4[b], H-3[c], H-6b[e], H-1a[Cer], H-2[Cer]), 4.11–4.05 (m, 2 H, H-4[a], ArC*H*$_2$), 4.00–3.91 (m, 4 H, H-6a[b], H-6a[c], H-5[e], H-1b[Cer]), 3.83–3.67 (m, 10 H, H-3[a], H-6b[b], H-5[c], H-6a[d], 2 OMe), 3.62–3.22 (m, 19 H, H-5[a], H-6a[a], H-6b[a], H-5[b], H-6b[c], H-6b[d], H-3[e], 10 OCH$_2$, 2 NHC*H*$_2$), 2.97 (m, 1 H, H-2[d]), 2.15 (s, 3 H, Ac), 2.08 (s, 3 H, Ac), 2.07 (s, 3 H, Ac), 2.01 (s, 3 H, Ac), 1.96–1.92 (m, 2 H, H-6a[Cer], H-6b[Cer]), 1.89 (s, 3 H, Ac), 1.81 (s, 3 H, Ac), 1.71–1.66 (m, 2 H, NHC(=O)C*H*$_2$), 1.38–1.08 (m, 70 H, 26 CH$_2$[Cer], 2 ^{t}Bu), 0.89–0.85 (m, 6 H, 2 Me[Cer]), 0.60 (s, 3 H, Ac); ^{13}C NMR (125 MHz, CDCl$_3$) δ 172.6, 170.5, 170.4, 170.3, 169.9, 169.9, 169.9, 166.2, 166.0, 165.8, 165.2, 165.1, 165.0, 165.0, 159.3, 159.0, 157.4, 157.1, 157.0, 156.5, 137.1, 133.6, 133.6, 133.4, 133.3, 133.3, 130.5, 130.1, 129.9, 129.8, 129.7, 129.7, 129.7, 129.6, 129.6, 129.6, 129.5, 129.1, 128.9, 128.8, 128.8, 128.7, 128.5, 128.5, 127.6, 126.8, 125.4, 125.3, 124.9, 117.1, 114.8, 113.9, 113.7, 101.5, 101.1, 100.6, 99.2, 98.4, 79.0, 78.6, 77.6, 75.3, 74.8, 74.7, 74.1, 73.5, 73.4, 73.3, 73.2, 72.5, 72.1, 71.5, 70.9, 70.7, 70.4, 70.3, 70.1, 70.1, 69.1, 68.5, 67.7, 67.5, 67.4, 66.3, 62.3, 61.8, 61.1, 60.9, 55.2, 50.4, 39.6, 36.4, 35.2, 35.0, 32.3, 31.9, 31.1, 30.0, 29.7, 29.7, 29.7, 29.6, 29.6, 29.5, 29.5, 29.4, 29.3, 29.3, 29.0, 25.6, 22.7, 22.0, 21.6, 20.8, 20.7, 20.7, 20.6, 20.5, 14.1; HRMS (ESI) *m/z*: found [M/2+Na]$^+$ 1511.1882, $C_{161}H_{208}F_3N_3O_{46}$ calcd for [M/2+Na]$^+$ 1511.1883.

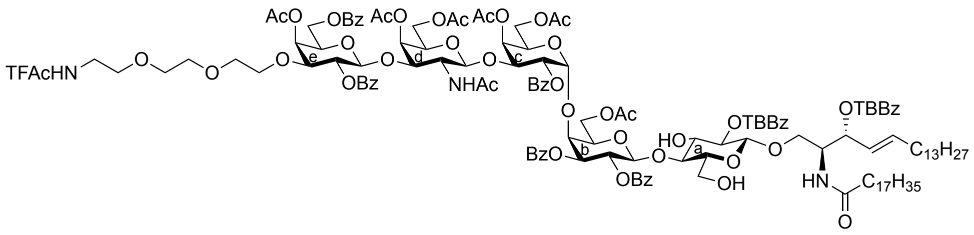

Compound 19

To a solution of compound **18** (55.5 mg, 18.6 μmol) in CH_2Cl_2 (1.2 mL) was added TFA (620 μL, 8.10 mmol) at 0 °C. After stirring for 30 min at 0 °C as the progress of the reaction was monitored by TLC (toluene/acetone = 3/1, developed twice), the reaction mixture was neutralized with satd. aq. $NaHCO_3$ and diluted with $CHCl_3$. The organic layer was washed with satd. aq. $NaHCO_3$ and brine, dried over Na_2SO_4, and concentrated. The residue was purified by silica gel column chromatography (toluene/acetone = 4/1) to give compound **19** (49.0 mg, 96%) as a white amorphous solid; $[\alpha]_D$ +58.7° (c 1.1, CHCl$_3$); ^{1}H NMR (500 MHz, CDCl$_3$) δ 8.05–7.03 (m, 34 H, 7 Ar, CF$_3$C(=O)NH), 5.90 (near quin, 1 H, $J_{5,6a}$ = $J_{5,6b}$ = 6.5 Hz, $J_{4,5}$ = 15.5 Hz, H-5[Cer]), 5.80 (d, 1 H, $J_{2,NH}$ = 10.0 Hz, NH-2[Cer]), 5.76 (dd, 1 H, $J_{1,2}$ =

8.0 Hz, $J_{2,3}$ = 10.8 Hz, H-2^{b}), 5.59 (t, 1 H, $J_{2,3}$ = $J_{3,4}$ = 8.8 Hz, H-3^{Cer}), 5.54 (d, 1 H, $J_{3,4}$ = 3.0 Hz, H-4^{e}), 5.50 (d, 1 H, $J_{3,4}$ = 3.2 Hz, H-4^{d}), 5.48–5.41 (m, 3 H, H-2^{c}, H-4^{c}, H-4^{Cer}), 5.25 (dd, 1 H, $J_{3,4}$ = 3.0 Hz, H-3^{b}), 5.23 (dd, 1 H, $J_{1,2}$ = 7.7 Hz, $J_{2,3}$ = 10.3 Hz, H-2^{e}), 5.17 (d, 1 H, $J_{1,2}$ = 8.0 Hz, H-1^{d}), 5.15–5.11 (m, 2 H, H-2^{a}, NH-2^{d}), 5.00 (d, 1 H, $J_{1,2}$ = 3.5 Hz, H-1^{c}), 4.89 (d, 1 H, H-1^{b}), 4.76 (dd, 1 H, $J_{2,3}$ = 11.0 Hz, H-3^{d}), 4.61 (d, 1 H, $J_{1,2}$ = 7.5 Hz, H-1^{e}), 4.50–4.41 (m, 4 H, H-1^{a}, H-5^{d}, H-6a^{e}, H-2^{Cer}), 4.33–4.27 (m, 3 H, H-4^{b}, H-3^{c}, H-6b^{e}), 4.20 (dd, 1 H, $J_{5,6a}$ = 7.8 Hz, J_{gem} = 10.3 Hz, H-6a^{b}), 4.14–4.11 (m, 2 H, H-6a^{c}, OH-3^{a}), 4.03–3.85 (m, 8 H, H-3^{a}, H-4^{a}, H-5^{b}, H-6b^{b}, H-5^{c}, H-6b^{c}, H-5^{e}, H-1a^{Cer}), 3.70 (m, 1 H, OCH$_2$), 3.67–3.63 (m, 2 H, H-6a^{d}, H-3^{e}), 3.55 (dd, 1 H, $J_{1b,2}$ = 3.5 Hz, J_{gem} = 9.5 Hz, H-1b^{Cer}), 3.50–3.24 (m, 13 H, H-6a^{a}, H-6b^{d}, 9 OCH$_2$, 2 NHCH_2), 3.16 (br d, 1 H, H-5^{a}), 3.04–2.97 (m, 2 H, H-6b^{a}, H-2^{d}), 2.90 (br s, 1 H, OH-6^{a}), 2.16 (s, 3 H, Ac), 2.11 (s, 3 H, Ac), 2.09 (s, 3 H, Ac), 2.03 (s, 3 H, Ac), 2.00–1.96 (m, 4 H, H-6a^{Cer}, H-6b^{Cer}, NHC(=O)CH_2), 1.84 (s, 3 H, Ac), 1.83 (s, 3 H, Ac), 1.49–1.22 (m, 70 H, 26 CH$_2^{Cer}$, 2 ^{t}Bu), 0.89–0.86 (m, 6 H, 2 Me^{Cer}), 0.65 (s, 3 H, Ac); ^{13}C NMR (125 MHz, CDCl$_3$) δ 172.7, 170.9, 170.5, 170.4, 170.1, 170.0, 169.9, 169.0, 166.0, 165.9, 165.6, 165.5, 165.2, 165.0, 157.4, 157.1, 157.0, 157.0, 138.5, 133.7, 133.5, 133.4, 133.3, 129.8, 129.8, 129.7, 129.7, 129.6, 129.5, 128.9, 128.8, 128.7, 128.6, 128.5, 128.5, 128.5, 127.3, 125.5, 125.4, 124.9, 117.1, 114.8, 102.2, 101.6, 99.7, 98.8, 98.3, 80.9, 78.6, 77.6, 77.3, 74.6, 74.2, 74.0, 74.0, 73.5, 73.4, 72.7, 72.6, 71.6, 71.2, 70.8, 70.8, 70.4, 70.2, 70.2, 70.1, 69.3, 69.2, 69.1, 68.5, 67.6, 66.4, 66.2, 62.5, 62.0, 61.2, 61.0, 59.6, 55.5, 50.4, 39.6, 36.8, 35.2, 35.1, 32.3, 31.9, 31.2, 31.1, 29.7, 29.7, 29.6, 29.6, 29.5, 29.4, 29.4, 29.3, 29.3, 28.9, 25.6, 22.7, 22.0, 20.8, 20.8, 20.7, 20.6, 20.2, 14.1; HRMS (ESI) *m/z*: found [M/2+Na]$^{+}$ 1391.1305, $C_{145}H_{192}F_3N_3O_{44}$ calcd for [M/2+Na]$^{+}$ 1391.1308.

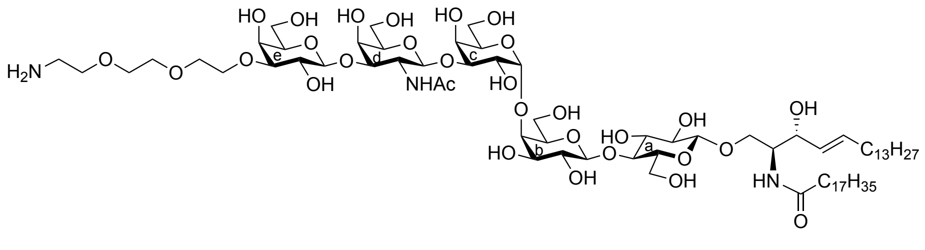

Compound 20

To a solution of compound **19** (37.5 mg, 13.7 µmol) in MeOH/THF = 1/1 (4.6 mL) was added 1 M NaOH aq. (274 µL, 0.274 mmol). After stirring for 43 h at ambient temperature as the progress of the reaction was monitored by TLC (CHCl$_3$/MeOH/H$_2$O/AcOH = 5/4/1/0.2), the reaction mixture was neutralized with Muromac (H^{+}), the resin was filtered through cotton, and washed with CHCl$_3$/MeOH = 1/1. The combined filtrate and washings were concentrated and co-evaporated with EtOH. The residue was purified by silica gel column chromatography (CHCl$_3$/MeOH/H$_2$O/28% NH$_3$ aq. =

5/1/0.05/0 → 5/4/1/0 → 3/3/1/0.1) to give compound **20** (22.3 mg, quant.) as a white amorphous solid; $[\alpha]_D$ +24.0° (c 1.0, $CHCl_3$/MeOH = 1/1); ^{1}H NMR (500 MHz, $CDCl_3$/CD_3OD = 1/1) δ 5.70 (near quin, 1 H, $J_{5,6a}$ = $J_{5,6b}$ = 6.5 Hz, $J_{4,5}$ = 15.4 Hz, H-5^{Cer}), 5.45 (dd, 1 H, $J_{3,4}$ = 7.8 Hz, H-4^{Cer}), 4.99 (d, 1 H, $J_{1,2}$ = 4.0 Hz, H-1^{c}), 4.75–4.29 (4 d, 4 H, H-1^{a}, H-1^{b}, H-1^{d}, H-1^{e}), 4.22–3.27 (m, 44 H, H-2^{a}, H-3^{a}, H-4^{a}, H-5^{a}, H-6a^{a}, H-6b^{a}, H-2^{b}, H-3^{b}, H-4^{b}, H-5^{b}, H-6a^{b}, H-6b^{b}, H-2^{c}, H-3^{c}, H-4^{c}, H-5^{c}, H-6a^{c}, H-6b^{c}, H-2^{d}, H-3^{d}, H-4^{d}, H-5^{d}, H-6a^{d}, H-6b^{d}, H-2^{e}, H-3^{e}, H-4^{e}, H-5^{e}, H-6a^{e}, H-6b^{e}, H-1a^{Cer}, H-1b^{Cer}, H-2^{Cer}, H-3^{Cer}, 5 OCH_2), 2.92–2.90 (m, 2 H, NH_2CH_2), 2.19–2.16 (m, 2 H, NHC(=O)CH_2), 2.05–2.02 (m, 2 H, H-6a^{Cer}, H-6b^{Cer}), 1.99 (s, 3 H, Ac), 1.60–1.58 (m, 2 H, NHC(=O)CH_2CH_2), 1.38-1.27 (m, 50 H, 25 CH_2^{Cer}), 0.90–0.88 (m, 6 H, 2 Me^{Cer}); ^{13}C NMR (125 MHz, $CDCl_3$/CD_3OD = 1/1) δ175.2, 174.4, 172.6, 134.9, 130.1, 129.4, 128.7, 125.7, 105.4, 104.6, 103.6, 103.2, 102.3, 82.6, 80.5, 80.5, 80.3, 80.2, 78.7, 75.7, 75.6, 75.4, 75.2, 74.0, 74.0, 72.5, 72.2, 71.7, 71.5, 71.0, 70.8, 70.3, 69.7, 69.3, 69.2, 68.8, 68.4, 66.3, 62.3, 62.0, 61.2, 61.0, 53.8, 52.8, 51.9, 41.1, 39.5, 37.0, 32.9, 32.4, 32.1, 30.9, 30.2, 30.2, 30.2, 30.1, 30.1, 30.0, 29.9, 29.9, 29.9, 29.8, 26.5, 23.2, 23.1, 21.5, 21.0, 14.3, 14.2; HRMS (ESI) *m/z*: found $[M+H]^+$ 1548.9364, $C_{74}H_{137}N_3O_{30}$ calcd for $[M+H]^+$ 1548.9360.

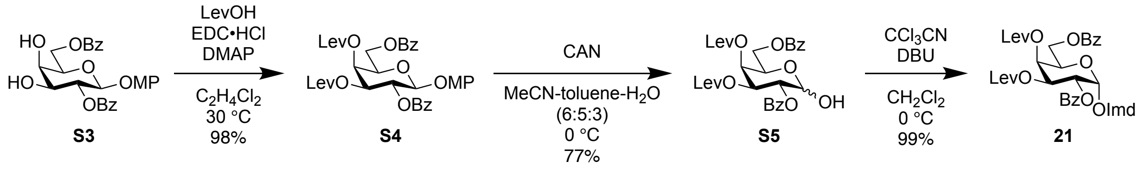

Supporting Scheme 2. Synthesis of Gal donor **21**.

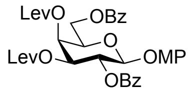

Compound S4

To a solution of compound **S3**[S04] (1.00 g, 2.02 mmol) in $C_2H_4Cl_2$ (40 mL) were added DMAP (247 mg, 2.02 mmol), LevOH (622 μL, 6.07 mmol) and EDC·HCl (1.16 g, 6.07 mmol). After stirring for 15 h at 30 °C as the progress of the reaction was monitored by TLC ($CHCl_3$/MeOH = 30/1), the reaction mixture was diluted with $CHCl_3$. The organic layer was washed with H_2O and brine, dried over Na_2SO_4, and concentrated. The residue was purified by silica gel column chromatography (*n*-hexane/EtOAc = 1/1 → 2/3) to give compound **S4** (1.37 g, 98%) as a white foam; $[\alpha]_D$ +20.0° (c 1.0, $CHCl_3$); ^{1}H NMR (500 MHz, $CDCl_3$) δ 8.05–6.64 (m, 14 H, 3 Ar), 5.74 (dd, 1 H, $J_{1,2}$ = 8.0 Hz, $J_{3,4}$ = 10.5 Hz, H-2), 5.62 (near d, 1 H, $J_{3,4}$ = 3.4 Hz, H-4), 5.27 (dd, 1 H, H-3), 5.06 (d, 1 H, H-1), 4.57 (dd, 1 H, $J_{5.6a}$ = 8.0 Hz, J_{gem} = 11.5 Hz, H-6a), 4.44 (dd, 1 H, $J_{5,6b}$ = 5.5 Hz, H-6b), 4.22 (near t, 1 H, H-5), 2.85–2.81 (m, 2 H, C(=O)CH_2),

2.79–2.71 (m, 2 H, C(=O)CH_2), 2.69–2.58 (m, 2 H, C(=O)CH_2), 2.52–2.41 (m, 2 H, C(=O)CH_2), 2.21 (s, 3 H, CH_2C(=O)*Me*), 2.03 (s, 3 H, CH_2C(=O)*Me*); ^{13}C NMR (125 MHz, $CDCl_3$) δ 206.0, 172.0, 171.9, 166.0, 165.2, 155.7, 151.1, 133.4, 133.3, 129.9, 129.8, 129.5, 129.3, 128.5, 128.5, 118.9, 114.4, 101.2, 71.4, 71.2, 69.3, 67.4, 62.0, 55.6, 37.9, 37.7, 29.8, 29.5, 27.9, 27.9; HRMS (ESI) *m/z*: found $[M+Na]^+$ 713.2205, $C_{37}H_{38}O_{13}$ calcd for $[M+Na]^+$ 713.2205.

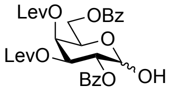

Compound S5

To a solution of compound **S4** (127 mg, 0.184 mmol) in MeCN/toluene/H_2O = 6/5/3 (3.7 mL) was added cerium (IV) ammonium nitrate (1.01 g, 1.84 mmol) at 0 °C. After stirring for 6 h at 0 °C as the progress of the reaction was monitored by TLC (toluene/acetone = 4/1), the reaction mixture was diluted with EtOAc. The organic layer was washed with H_2O, satd. aq. $NaHCO_3$ and brine, dried over Na_2SO_4, and concentrated. The residue was purified by silica gel column chromatography (toluene/acetone = 20/1 → 10/1 → 8/1 → 3/1) to give compound **S5** (83.5 mg, 77%, α:β = 1:0.3) as a white foam; ^{1}H NMR (500 MHz, $CDCl_3$) δ 8.06–7.42 (m, 20 H, 2 Phα,β), 5.71 (t. 1 H, $J_{1,2}$ = $J_{1,OH}$ = 2.8 Hz, H-1α), 5.68–5.64 (m, 2 H, H-3α, H-4α), 5.59 (br d, 1 H, H-4β), 5.40 (dd, 1 H, $J_{2,3}$ = 10.5 Hz, H-2α), 5.33–5.32 (m, 2 H, H-2β, H-3β), 4.87 (m, 1 H, H-1β), 4.67 (t, 1 H, $J_{5,6a}$ = $J_{5,6b}$ = 6.8 Hz, H-5α), 4.54 (dd, 1 H, $J_{5,6a}$ = 6.8 Hz, J_{gem} = 11.4 Hz, H-6aβ), 4.48 (dd, 1 H, J_{gem} = 11.5 Hz, H-6aα), 4.38 (dd, 1 H, $J_{5,6b}$ = 6.5 Hz, H-6bβ), 4.32 (dd, 1 H, H-6bα), 4.16 (dd, 1 H, H-5β), 3.96 (d, 1 H, $J_{1,OH}$ = 8.5 Hz, OH-1β), 3.03 (d, 1 H, OH-1α), 2.80–2.40 (m, 16 H, 2 C(=O)CH_2α,β), 2.18 (s, 3 H, CH_2C(=O)*Me*β), 2.17 (s, 3 H, CH_2C(=O)*Me*α), 2.05 (s, 3 H, CH_2C(=O)*Me*α), 2.03 (s, 3 H, CH_2C(=O)*Me*β); HRMS (ESI) *m/z*: found $[M+Na]^+$ 607.1786, $C_{30}H_{32}O_{12}$ calcd for $[M+Na]^+$ 607.1786.

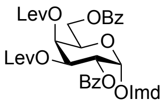

Compound 21

To a solution of compound **S5** (640 mg, 1.09 mmol) in CH_2Cl_2 (22 mL) were added CCl_3CN (2.20 mL, 21.9 mmol) and DBU (32.7 µL, 0.219 mmol) at 0 °C. After stirring for 3.5 h at 0 °C as the progress of the reaction was monitored by TLC ($CHCl_3$/MeOH = 30/1), the reaction mixture was concentrated. The residue was purified by silica gel column chromatography (toluene/acetone = 12/1) to give compound **21** (785 mg, 99%) as a colorless syrup; $[\alpha]_D$ +89.7° (c 1.0, $CHCl_3$); ^{1}H NMR (500 MHz, $CDCl_3$) δ 8.58 (s, 1 H, C(=NH)CCl_3), 8.02–7.43 (m, 10 H, 2 Ph), 6.76 (d, 1 H, $J_{1,2}$ = 3.0 Hz, H-1), 5.74 (m, 1 H, H-

4), 5.69–5.64 (m, 2 H, H-2, H-3), 4.65 (near t, 1 H, H-5), 4.48 (dd, 1 H, $J_{5,6a}$ = 7.0 Hz, J_{gem} = 11.5 Hz, H-6a), 4.37 (dd, 1 H, $J_{5,6b}$ = 6.0 Hz, H-6b), 2.85–2.60 (m, 6 H, 3 C(=O)CH_2), 2.56–2.43 (m, 2 H, C(=O)CH_2), 2.18 (s, 3 H, CH_2C(=O)*Me*), 2.04 (s, 3 H, CH_2C(=O)*Me*); ^{13}C NMR (125 MHz, $CDCl_3$) δ 206.0, 205.9, 171.9, 171.9, 165.9, 165.6, 160.5, 133.6, 133.2, 129.9, 129.7, 129.5, 128.8, 128.5, 93.6, 90.7, 69.4, 67.8, 67.8, 67.4, 62.0, 37.8, 37.7, 29.7, 29.5, 27.8, 27.8; HRMS (ESI) *m/z*: found [M+Na]$^+$ 750.0882, $C_{32}H_{32}Cl_3NO_{12}$ calcd for [M+Na]$^+$ 750.0882.

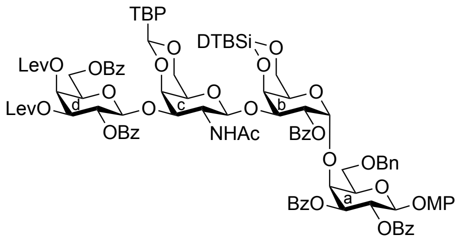

Compound 22

To a solution of donor **21** (291 mg, 0.399 mmol) and acceptor **10** (446 mg, 0.333 mmol) in CH_2Cl_2 (7.3 mL) was added 4 Å molecular sieves (AW-300, 1.00 g). After stirring for 1 h at ambient temperature, TMSOTf (6.0 µL, 33.3 µmol) was added at 0 °C. After stirring for 9 h at 0 °C as the progress of the reaction was monitored by TLC (toluene/acetone = 5/1), the reaction mixture was neutralized with satd. aq. $NaHCO_3$ and filtered through a pad of Celite. The pad was washed with $CHCl_3$. The combined filtrate and washings were diluted with $CHCl_3$ and washed with brine. The organic layer was dried over Na_2SO_4 and concentrated. The residue was purified by silica gel column chromatography (toluene/acetone = 8/1 → 7/1) to give compound **22** (551 mg, 87%) as a white amorphous solid; $[\alpha]_D$ +62.4° (c 0.9, $CHCl_3$); ^{1}H NMR (500 MHz, $CDCl_3$) δ 8.08–6.72 (m, 38 H, 8 Ar), 5.93 (dd, 1 H, $J_{1,2}$ = 7.7 Hz, $J_{2,3}$ = 10.9 Hz, H-2^a), 5.56 (dd, 1 H, $J_{1,2}$ = 4.0 Hz, $J_{2,3}$ = 10.5 Hz, H-2^b), 5.40 (d, 1 H, $J_{3,4}$ = 3.3 Hz, H-4^d), 5.43 (dd, 1 H, $J_{1,2}$ = 8.0 Hz, $J_{2,3}$ = 10.3 Hz, H-2^d, 5.41 (s, 1 H, ArC*H*<), 5.31 (dd, 1 H, $J_{3,4}$ = 2.5 Hz, H-3^a), 5.25–5.23 (m, 2 H, H-1^c, NH-2^c), 5.17 (d, 1 H, H-1^b), 5.14 (dd, 1 H, H-3^d), 5.09 (d, 1 H, H-1^a), 4.87 (dd, 1 H, $J_{2,3}$ = 11.0 Hz, $J_{3,4}$ = 3.3 Hz, H-3^c), 4.78 (d, 1 H, H-1^d), 4.70 (d, 1 H, $J_{3,4}$ = 2.5 Hz, H-4^b), 4.66 (dd, 1 H, $J_{5,6a}$ = 7.5 Hz, J_{gem} = 11.3 Hz, H-6a^d), 4.36 (d, 1 H, H-4^a), 4.27 (d, 1 H, H-4^c), 4.25–4.21 (m, 2 H, H-3^b, H-6b^d), 4.17 (d, 1 H, J_{gem} = 11.5 Hz, H-6a^c), 4.11 (dd, 1 H, $J_{5,6b}$ = 5.8 Hz, H-5^d), 4.07 (s, 3 H, H-5^b, PhC*H*$_2$), 3.89 (t, 1 H, $J_{5,6a}$ = $J_{5,6b}$ = 6.5 Hz, H-5^a), 3.74 (s, 3 H, OMe), 3.60 (d, 1 H, H-6b^c), 3.54–3.45 (m, 4 H, H-6a^a, H-6b^a, H-6a^b, H-6b^b), 3.37 (s, 1 H, H-5^c), 3.27 (m, 1 H, H-2^c), 2.75–2.72 (m, 4 H, 2 C(=O)CH_2), 2.56–2.52 (m, 2 H, C(=O)CH_2), 2.38–2.35 (m, 2 H, C(=O)CH_2), 2.11 (s, 3 H, CH_2C(=O)*Me*), 1.95 (s, 3 H, CH_2C(=O)*Me*), 1.33 (s, 9 H, ^{t}Bu), 1.00 (s, 9 H, ^{t}Bu), 0.91 (s, 9 H, ^{t}Bu), 0.62 (s, 3 H, Ac); ^{13}C NMR (125 MHz, $CDCl_3$) δ 206.2, 205.9, 171.8, 171.7, 170.9, 166.0, 165.9, 165.5, 164.8, 155.6, 151.6, 151.3, 137.5,

135.6, 133.6, 133.5, 133.4, 133.3, 130.3, 129.8, 129.7, 129.6, 129.6, 129.4, 129.4, 129.4, 129.0, 128.9, 128.7, 128.5, 128.4, 128.3, 128.2, 127.7, 127.7, 126.4, 125.3, 124.9, 118.9, 114.4, 102.4, 101.1, 100.8, 99.7, 98.0, 75.9, 75.1, 74.1, 73.9, 73.5, 73.2, 71.1, 71.0, 69.6, 69.4, 69.3, 69.0, 68.1, 68.0, 67.5, 66.6, 66.2, 62.4, 55.6, 54.8, 37.8, 37.7, 34.6, 31.4, 29.7, 29.4, 28.0, 27.8, 27.5, 27.4, 23.3, 22.3, 20.6; HRMS (ESI) *m/z*: found $[M+Na]^+$ 1926.7271, $C_{104}H_{117}NO_{31}Si$ calcd for $[M+Na]^+$ 1926.7271.

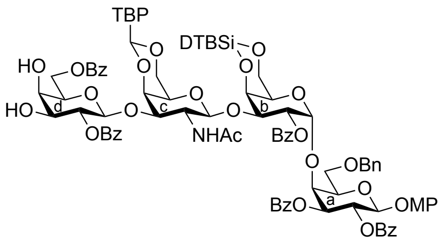

Compound 23

To a solution of compound **22** (118 mg, 61.9 µmol) in MeOH/THF = 1/5 (3.1 mL) was added N_2H_4·AcOH (57.0 mg, 0.619 mmol). After stirring for 1 h at ambient temperature as the progress of the reaction was monitored by TLC (toluene/acetone = 2/1), the reaction mixture was diluted with EtOAc. The organic layer was washed with satd. aq. $NaHCO_3$ and brine, dried over Na_2SO_4, and concentrated. The residue was purified by silica gel column chromatography (toluene/acetone = 11/3) to give compound **23** (101 mg, 95%) as a white amorphous solid; $[\alpha]_D$ +57.5° (c 1.0, $CHCl_3$); ^1H NMR (500 MHz, CD_3NO_2, 70 °C) δ 8.14–6.82 (m, 38 H, 8 Ar), 5.96 (dd, 1 H, $J_{1,2}$ = 8.0 Hz, $J_{2,3}$ = 10.7 Hz, H-2^a), 5.72 (dd, 1 H, $J_{1,2}$ = 3.5 Hz, $J_{2,3}$ = 10.5 Hz, H-2^b), 5.47 (dd, 1 H, $J_{3,4}$ = 2.8 Hz, H-3^a), 5.43 (s, 1 H, ArC*H*<), 5.38–5.36 (m, 2 H, H-1^a, NH-2^c), 5.27 (d, 1 H, H-1^b), 5.14 (near t, 1 H, H-2^d), 5.04 (br d, 1 H, H-1^c), 4.91 (br s, 1 H, H-4^c), 4.81–4.76 (m, 2 H, H-1^d, H-6a^d), 4.54–4.51 (m, 2 H, H-4^a, H-6b^d), 4.42 (d, 1 H, $J_{3,4}$ = 2.5 Hz, H-4^b), 4.36–4.09 (m, 9 H, H-5^a, H-3^b, H-5^b, H-3^c, H-6a^c, H-4^d, H-5^d, PhCH_2), 3.94 (m, 1 H, H-3^d), 3.77 (s, 3 H, OMe), 3.72–3.59 (m, 4 H, H-6a^a, H-6b^a, H-2^c, H-6b^c), 3.53 (d, 1 H, J_{gem} = 12.8 Hz, H-6a^b), 3.45 (d, 1 H, H-6b^b), 3.37 (s, 1 H, H-5^c), 3.06 (br s, 1 H, OH-3^d, OH-4^d), 1.32 (s, 9 H, ^{t}Bu), 1.04 (s, 9 H, ^{t}Bu), 1.02 (s, 9 H, ^{t}Bu), 0.84 (br s, 3 H, Ac); ^{13}C NMR (125 MHz, CD_3NO_2, 70 °C) δ 167.9, 167.4, 167.3, 167.2, 157.5, 153.8, 152.7, 139.8, 137.4, 135.1, 134.9, 134.7, 134.6, 131.9, 131.6, 131.3, 131.1, 131.0, 130.9, 130.8, 130.1, 130.1, 129.9, 129.8, 129.7, 129.1, 127.8, 126.4, 120.3, 116.2, 103.9, 103.3, 102.5, 102.4, 99.4, 79.4, 79.0, 77.1, 75.5, 75.4, 75.1, 75.0, 74.6, 74.4, 74.3, 73.7, 71.8, 70.9, 70.5, 70.2, 69.9, 69.7, 68.1, 68.0, 65.4, 56.6, 54.2, 35.6, 31.8, 28.4, 28.2, 24.3, 23.2, 21.6; HRMS (ESI) *m/z*: found $[M+Na]^+$ 1730.6535, $C_{94}H_{105}NO_{27}Si$ calcd for $[M+Na]^+$ 1730.6535.

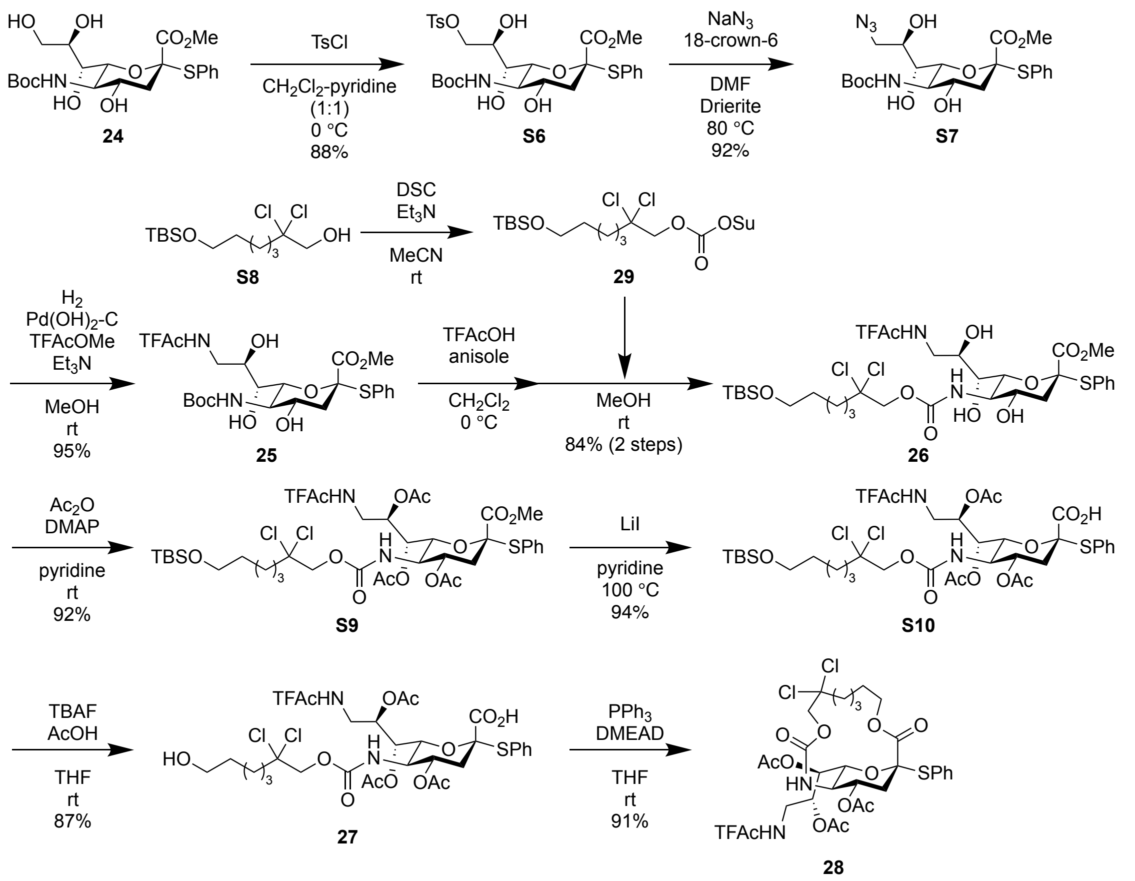

Supporting Scheme 3. Synthesis of Neu donor **28**.

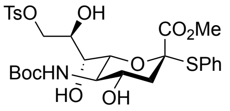

Compound S6

To a solution of compound **24**[S05] (47.1 mg, 99.5 µmol) in CH_2Cl_2/pyridine = 1/1 (2.0 mL) was added TsCl (28.4 mg, 0.149 mmol) at 0 °C. After stirring for 18.5 h at 0 °C as the progress of the reaction was monitored by TLC (*n*-hexane/acetone = 1/1), the reaction mixture was diluted with $CHCl_3$. The organic layer was washed with 2 M HCl, H_2O, satd. aq. $NaHCO_3$ and brine, dried over Na_2SO_4, and concentrated. The residue was purified by silica gel column chromatography (*n*-hexane/acetone = 2/1) to give compound **S6** (55.2 mg, 88%) as a white amorphous solid; $[\alpha]_D$ +65.7° (c 1.0, $CHCl_3$); ^1H NMR (500 MHz, acetone-d_6) δ 7.82–7.36 (m, 9 H, 2 Ar), 6.18 (d, 1 H, $J_{5,NH}$ = 8.5 Hz, NH-5), 4.56 (d, 1 H, $J_{4,OH}$ = 5.5 Hz, OH-4), 4.49 (d, 1 H, $J_{7,OH}$ = 4.0 Hz, OH-7), 4.28 (dd, 1 H, $J_{8,9a}$ = 1.5 Hz, J_{gem} = 9.9 Hz, H-9a), 4.12 (dd, 1 H, $J_{8,9b}$ = 5.3 Hz, H-9b), 3.97 (m, 1 H, H-8), 3.75 (m, 1 H, H-4), 3.64 (s, 3 H, C(=O)OMe), 3.54 (near td, 1 H, $J_{6,7}$ = 1.6 Hz, $J_{7,8}$ = 4.7 Hz, H-7), 3.53–3.47 (m, 2 H, H-5, OH-8), 3.28 (dd, 1 H, $J_{5,6}$ = 10.5 Hz, H-6),

2.84 (dd, 1 H, J_{gem} = 12.5 Hz, $J_{3eq,4}$ = 5.0 Hz, H-3*eq*), 2.46 (s, 3 H, Ar*Me*), 1.87 (dd, 1 H, $J_{3ax,4}$ = 11.5 Hz, H-3*ax*), 1.42 (s, 9 H, ^{t}Bu); ^{13}C NMR (125 MHz, acetone-d_6) δ 170.5, 159.0, 145.6, 137.5, 134.5, 130.9, 130.8, 129.8, 129.7, 128.7, 87.4, 80.6, 77.4, 73.0, 70.1, 69.5, 68.3, 54.4, 53.3, 41.7, 28.5, 21.5; HRMS (ESI) *m/z*: found [M+Na]$^+$ 650.1695, $C_{28}H_{37}NO_{11}S_2$ calcd for [M+Na]$^+$ 650.1695.

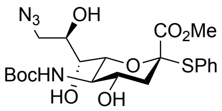

Compound S7

To a solution of compound **S6** (4.21 g, 6.71 mmol) in DMF (134 mL) were added Drierite (4.21 g), 18-crown-6 (1.05 g, 3.97 mmol) and NaN_3 (3.49 g, 53.7 mmol). After stirring for 2.5 h at 80 °C as the progress of the reaction was monitored by TLC (*n*-hexane/acetone = 3/2), the reaction mixture was filtered through a pad of Celite. The pad was washed with $CHCl_3$. The combined filtrate and washings were co-evaporated with toluene. The residue was purified by silica gel column chromatography (*n*-hexane/acetone = 2/1) to give compound **S7** (3.07 g, 92%) as a white foam; $[\alpha]_D$ +56.3° (c 1.0, $CHCl_3$); ^{1}H NMR (500 MHz, acetone-d_6) δ 7.57–7.37 (m, 5 H, Ph), 6.19 (d, 1 H, $J_{5,NH}$ = 8.5 Hz, NH-5), 4.57 (d, 1 H, $J_{4,OH}$ = 5.5 Hz, OH-4), 4.54 (d, 1 H, $J_{7,OH}$ = 4.0 Hz, OH-7), 3.98 (m, 1 H, H-8), 3.76 (m, 1 H, H-4), 3.68 (s, 3 H, C(=O)OMe), 3.56–3.47 (m, 4 H, H-5, H-7, H-9a, OH-8), 3.32 (dd, 1 H, $J_{6,7}$ = 1.5 Hz, $J_{5,6}$ = 10.8 Hz, H-6), 3.29 (dd, 1 H, $J_{8,9b}$ = 6.5 Hz, J_{gem} = 13.0 Hz, H-9b), 2.86 (dd, 1 H, $J_{3eq,4}$ = 4.8 Hz, J_{gem} = 12.8 Hz, H-3*eq*), 1.90 (dd, 1 H, $J_{3ax,4}$ = 11.8 Hz, H-3*ax*), 1.42 (s, 9 H, ^{t}Bu); ^{13}C NMR (125 MHz, acetone-d_6) δ 170.7, 159.0, 137.5, 131.0, 129.8, 129.7, 87.3, 80.6, 77.6, 72.0, 70.8, 68.3, 54.5, 54.4, 53.4, 41.7, 28.5; HRMS (ESI) *m/z*: found [M+Na]$^+$ 521.1677, $C_{21}H_{30}N_4O_8S$ calcd for [M+Na]$^+$ 521.1677.

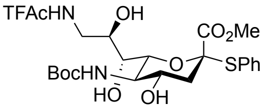

Compound 25

To a solution of compound **S7** (3.79 g, 5.60 mmol) in MeOH (280 mL) were added Et_3N (7.76 mL, 56.0 mmol), TFAcOMe (2.78 mL, 28.0 mmol) and $Pd(OH)_2$-C (393 mg, 20% on carbon). After stirring for 1.5 h at ambient temperature as the progress of the reaction was monitored by TLC (*n*-hexane/acetone = 3/2), the reaction mixture was filtered through a pad of Celite, the pad was washed with $CHCl_3$, and the combined filtrate and washings were concentrated. The residue was purified by silica gel column chromatography (*n*-hexane/acetone = 2/1) to give compound **25** (4.12 g, 95%) as a white foam; $[\alpha]_D$

+70.8° (c 1.0, $CHCl_3$); ^1H NMR (500 MHz, acetone-d_6) δ 8.07 (br s, 1 H, $CF_3C(=O)NH$), 7.56–7.36 (m, 5 H, Ph), 6.18 (d, 1 H, $J_{5,NH}$ = 8.0 Hz, NH-5), 4.63 (d, 1 H, $J_{7,OH}$ = 4.5 Hz, OH-7), 4.57 (d, 1 H, $J_{4,OH}$ = 5.5 Hz, OH-4), 3.93 (m, 1 H, H-8), 3.79–3.67 (m, 5 H, H-4, H-9a, C(=O)OMe), 3.53 (near q, 1 H, H-5), 3.46 (d, 1 H, $J_{8,OH}$ = 3.0 Hz, OH-8), 3.41–3.35 (m, 2 H, H-7, H-9b), 3.31 (dd, 1 H, $J_{6,7}$ = 1.5 Hz, $J_{5,6}$ = 10.5 Hz, H-6), 2.85 (dd, 1 H, $J_{3eq,4}$ = 4.5 Hz, J_{gem} = 12.5 Hz, H-3*eq*), 1.90 (dd, 1 H, $J_{3ax,4}$ = 11.5 Hz, H-3*ax*), 1.41 (s, 9 H, ^{t}Bu); ^{13}C NMR (125 MHz, acetone-d_6) δ 170.7, 158.9, 157.6, 157.3, 137.6, 130.9, 129.8, 129.6 118.4, 116.1, 87.2, 80.5, 77.6, 72.1, 70.1, 68.3, 54.4, 53.3, 43.9, 41.7, 28.5; HRMS (ESI) *m/z*: found $[M+Na]^+$ 591.1591, $C_{23}H_{31}F_3N_2O_9S$ calcd for $[M+Na]^+$ 591.1595.

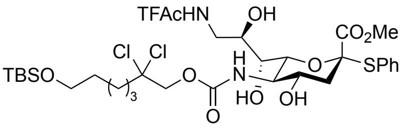

Compound 26

To a solution of compound **25** (3.12 g, 5.49 mmol) in CH_2Cl_2 (220 mL) were added anisole (1.19 mL, 11.0 mmol) and TFA (55 mL) at 0 °C. After stirring for 1 h at 0 °C as the progress of the reaction was monitored by TLC ($CHCl_3$/MeOH = 10/1), the reaction mixture was concentrated and the resulting amine was dried for 2 h. To a solution of compound **S8** (6.94 g, 22.0 mmol) in MeCN (110 mL) were added Et_3N (7.65 mL, 54.9 mmol) and DSC (5.75 g, 22.0 mmol). After stirring for 2 h at room temperature as the progress of the reaction was monitored by TLC (*n*-hexane/EtOAc = 5/1), the reaction mixture was added to a solution of amine in MeOH (110 mL) at 0 °C. After stirring for 5 h at ambient temperature as the progress of the reaction was monitored by TLC ($CHCl_3$/MeOH = 10/1), the reaction mixture was concentrated. The residue was purified by silica gel column chromatography (*n*-hexane/acetone = 4/1 → 3/1) to give compound **26** (3.75 g, 84%) as a white foam; $[\alpha]_D$ +57.8° (c 1.0, $CHCl_3$); ^1H NMR (500 MHz, acetone-d_6) δ 8.14 (br s, 1 H, $CF_3C(=O)NH$), 7.57–7.36 (m, 5 H, Ph), 6.74 (d, 1 H, $J_{5,NH}$ = 8.5 Hz, NH-5), 4.64–4.61 (m, 2 H, NHC(=O)OCH_2, OH-4), 4.43 (d, 1 H, J_{gem} = 12.0 Hz, NHC(=O)OCH_2), 4.25 (d, 1 H, $J_{7,OH}$ = 5.5 Hz, OH-7), 3.92 (m, 1 H, H-8), 3.84–3.74 (m, 2 H, H-4, H-9a), 3.67–3.63 (m, 6 H, H-5, 2 CH_2OSi(^{t}Bu)Me_2, C(=O)OMe), 3.47–3.43 (m, 3 H, H-6, H-7, OH-8), 3.25 (m, 1 H, H-9b), 2.85 (m, 1 H, H-3*eq*), 2.23 (m, 2 H, $CCl_2CH_2CH_2$), 1.92 (dd, 1 H, J_{gem} = 12.5 Hz, $J_{3ax,4}$ = 11.5 Hz, H-3*ax*), 1.68 (m, 2 H, $CCl_2CH_2CH_2$), 1.55 (m, 2 H, CH_2), 1.43 (m, 2 H, CH_2), 0.89 (s, 9 H, OSi(^{t}*Bu*)Me_2), 0.05 (s, 6 H, OSi(^{t}Bu)*Me_2*); ^{13}C NMR (125 MHz, acetone-d_6) δ 170.6, 157.8, 157.7, 157.4, 137.6, 130.9, 129.7, 129.6, 118.4, 116.1, 92.0, 87.2, 77.1, 72.3, 72.2, 70.3, 68.4, 63.4, 54.9, 53.3, 44.8, 44.1, 41.7, 33.3, 26.3, 26.0, 25.3, 18.8, –5.2; HRMS (ESI) *m/z*: found $[M+Na]^+$ 831.2099, $C_{32}H_{49}Cl_2F_3N_2O_{10}SSi$ calcd for $[M+Na]^+$ 831.2099.

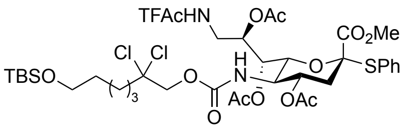

Compound S9

To a solution of compound **26** (2.10 g, 2.59 mmol) in pyridine (26 mL) were added Ac_2O (985 µL, 10.4 mmol) and DMAP (31.6 mg, 0.259 mmol) at 0 °C. After stirring for 4 h at ambient temperature as the progress of the reaction was monitored by TLC (*n*-hexane/acetone = 2/1), Ac_2O (491 µL, 5.18 mmol) was added to complete the reaction. After stirring for another 2 h at ambient temperature, the reaction was quenched with MeOH at 0 °C. The reaction mixture was co-evaporated with toluene and diluted with $CHCl_3$. . The organic layer was washed with 2 M HCl, H_2O, satd. aq. $NaHCO_3$ and brine, dried over Na_2SO_4, and concentrated. The residue was purified by silica gel column chromatography (*n*-hexane/acetone = 5/1) to give compound **S9** (2.22 g, 92%) as a white foam; $[\alpha]_D$ +16.3° (c 1.0, $CHCl_3$); ^{1}H NMR (500 MHz, $CDCl_3$) δ 7.49–7.29 (m, 6 H, Ph, $CF_3C(=O)NH$), 5.14 (dt, 1 H, $J_{8,9a} = J_{8,9b}$ = 2.6 Hz, $J_{7,8}$ = 9.5 Hz, H-8), 5.09 (dd, 1 H, $J_{6,7}$ = 1.5 Hz, H-7), 4.82 (td, 1 H, $J_{3eq,4}$ = 4.5 Hz, $J_{3ax,4} = J_{4,5}$ = 11.0 Hz, H-4), 4.70 (d, 1 H, $J_{5,NH}$ = 10.0 Hz, NH-5), 4.54 (d, 1 H, J_{gem} = 12.0 Hz, NHC(=O)OCH_2), 4.30 (d, 1 H, NHC(=O)OCH_2), 4.27 (ddd, 1 H, $J_{9a,NH}$ = 8.8 Hz, J_{gem} = 14.9 Hz, H-9a), 3.87 (dd, 1 H, $J_{5,6}$ = 10.5 Hz, H-6), 3.79 (near q, 1 H, H-5), 3.62 (t, 2 H, CH_2OSi(^{t}Bu)Me_2), 3.54 (s, 3 H, C(=O)OMe), 2.95 (dt, 1 H, $J_{9b,NH}$ = 3.3 Hz, H-9b), 2.88 (dd, 1 H, J_{gem} = 13.0 Hz, H-3*eq*), 2.25 (s, 3 H, Ac), 2.12 (m, 2 H, $CCl_2CH_2CH_2$), 2.05–2.00 (m, 4 H, H-3*ax*, Ac), 1.98 (s, 3 H, Ac), 1.66 (m, 2 H, $CCl_2CH_2CH_2$), 1.56 (m, 2 H, CH_2), 1.40 (m, 2 H, CH_2), 0.89 (s, 9 H, OSi(^{t}*Bu*)Me_2), 0.05 (s, 6 H, Si(^{t}Bu)*Me*$_2$); ^{13}C NMR (125 MHz, $CDCl_3$) δ 172.2, 170.7, 169.7, 167.5, 158.0, 157.7, 154.9, 136.1, 130.0, 129.0, 129.0, 128.5, 128.2, 125.3, 117.0, 114.8, 90.0, 87.5, 74.1, 71.8, 69.3, 68.5, 67.9, 62.9, 52.8, 51.5, 44.1, 38.4, 38.3, 32.5, 26.0, 25.3, 24.5, 21.1, 20.9, 20.7, 18.4, –5.3; HRMS (ESI) *m/z*: found $[M+Na]^+$ 957.2415, $C_{38}H_{55}Cl_2F_3N_2O_{13}SSi$ calcd for $[M+Na]^+$ 957.2415.

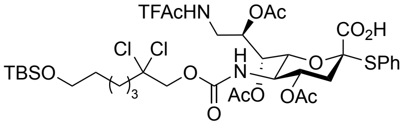

Compound S10

To a solution of compound **S9** (3.46 g, 3.70 mmol) in pyridine (74 mL) was added LiI (2.48 g, 18.5 mmol) at 0 °C. After stirring for 38 h at 100 °C as the progress of the reaction was monitored by TLC ($CHCl_3$/MeOH = 5/1), the reaction mixture was co-evaporated with toluene and diluted with $CHCl_3$. The organic layer was washed with 2 M HCl, H_2O, satd. aq. $NaHCO_3$ and brine, dried over Na_2SO_4, and

concentrated. The residue was purified by silica gel column chromatography ($CHCl_3$/MeOH = 60/1 → 20/1) to give compound **S10** (3.20 g, 94%) as a white foam; $[\alpha]_D$ +26.3° (c 1.0, $CHCl_3$); ^{1}H NMR (500 MHz, $CDCl_3/CD_3OD$ = 1/1) δ 7.64–7.34 (m, 5 H, Ph), 5.32 (br d, 1 H, $J_{7,8}$ = 4.0 Hz, H-7), 5.17 (m, 1 H, H-8), 4.95 (td, 1 H, $J_{3eq,4}$ = 4.5 Hz, $J_{3ax,4}$ = $J_{4,5}$ = 11.0 Hz, H-4), 4.54 (d, 1 H, J_{gem} = 12.0 Hz, NHC(=O)OC*H*$_2$), 4.27 (d, 1 H, NHC(=O)OC*H*$_2$), 4.07–4.02 (m, 2 H, H-6, H-9a), 3.64 (t, 2 H, C*H*$_2$OSi(^{t}Bu)Me_2), 3.57–3.51 (m, 2 H, H-5, H-9b), 2.84 (dd, 1 H, J_{gem} = 12.3 Hz, H-3*eq*), 2.20 (s, 3 H, Ac), 2.15 (m, 2 H, CCl_2C*H*$_2$$CH_2$), 2.00 (s, 3 H, Ac), 1.98 (s, 3 H, Ac), 1.75 (t, 1 H, H-3*ax*), 1.67 (m, 2 H, CCl_2CH_2C*H*$_2$), 1.57 (m, 2 H, CH_2), 1.41 (m, 2 H, CH_2), 0.90 (s, 9 H, OSi(^{t}*Bu*)Me_2), 0.07 (s, 6 H, Si(^{t}Bu)*Me*$_2$); ^{13}C NMR (125 MHz, $CDCl_3/CD_3OD$ = 1/1) δ 171.7, 171.5, 171.4, 158.7, 156.2, 136.7, 130.3, 129.9, 129.2, 117.7, 115.4, 90.9, 88.7, 74.8, 72.4, 71.9, 71.1, 69.2, 63.5, 51.5, 44.5, 39.7, 38.6, 32.9, 26.2, 25.7, 25.0, 21.1, 20.9, 20.8, 18.7, –5.2; HRMS (ESI) *m/z*: found [M–H]$^-$ 919.2294, $C_{37}H_{53}Cl_2F_3N_2O_{13}SSi$ calcd for [M–H]$^-$ 919.2294.

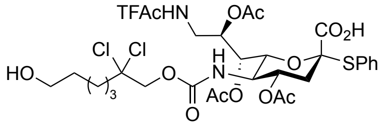

Compound 27

To a solution of compound **S10** (1.50 g, 1.63 mmol) in THF (33 mL) were added AcOH (290 µL,4.88 mmol) and 1 M TBAF in THF (4.88 mL, 4.88 mmol). After stirring for 36 h at ambient temperature as the progress of the reaction was monitored by TLC ($CHCl_3$/MeOH = 3/1), the reaction mixture was diluted with EtOAc. The organic layer was washed with 2 M HCl, H_2O, satd. aq. $NaHCO_3$ and brine, dried over Na_2SO_4, and concentrated. The residue was purified by silica gel column chromatography ($CHCl_3$/MeOH = 40/1 → 10/1) to give compound **27** (1.15 g, 87%) as a white foam; $[\alpha]_D$ +31.0° (c 1.0, $CHCl_3$); ^{1}H NMR (500 MHz, $CDCl_3/CD_3OD$ = 1/1) δ 7.59–7.33 (m, 5 H, Ph), 6.99 (d, 1 H, $J_{5,NH}$ = 10.0 Hz, NH-5), 5.26 (dd, 1 H, $J_{6,7}$ = 1.5 Hz, $J_{7,8}$ = 6.5 Hz, H-7), 5.17 (td, 1 H, $J_{8,9a}$ = 3.0 Hz, $J_{8,9b}$ = 6.5 Hz, H-8), 4.92 (td, 1 H, $J_{3eq,4}$ = 4.7 Hz, $J_{3ax,4}$ = $J_{4,5}$ = 10.9 Hz, H-4), 4.53 (d, 1 H, J_{gem} = 11.5 Hz, NHC(=O)OC*H*$_2$), 4.29 (d, 1 H, NHC(=O)OC*H*$_2$), 4.01 (br d, 1 H, $J_{5,6}$ = 10.5 Hz, H-6), 3.93 (dd, 1 H, J_{gem} = 14.8 Hz, H-9a), 3.63–3.57 (m, 3 H, H-5, C*H*$_2$OH), 3.40 (dd, 1 H, H-9b), 2.86 (dd, 1 H, J_{gem} = 12.5 Hz, H-3*eq*), 2.22 (s, 3 H, Ac), 2.18 (m, 2 H, CCl_2C*H*$_2$$CH_2$), 2.00 (s, 3 H, Ac), 1.97 (s, 3 H, Ac), 1.85 (t, 1 H, H-3*ax*), 1.68 (m, 2 H, CCl_2CH_2C*H*$_2$), 1.60 (m, 2 H, CH_2), 1.43 (m, 2 H, CH_2); ^{13}C NMR (125 MHz, $CDCl_3/CD_3OD$ = 1/1) δ 171.0, 170.1, 170.1, 168.8, 157.7, 157.4, 155.2, 155.2, 135.7, 129.1, 128.4, 128.2, 118.8, 116.6, 114.3, 89.7, 86.5, 73.4, 70.8, 69.5, 69.5, 67.8, 61.1, 50.4, 50.3, 43.5, 43.4, 38.4, 37.5, 31.5, 24.5, 23.9, 19.8, 19.5; HRMS (ESI) *m/z*: found [M–H]$^-$ 805.1429, $C_{31}H_{39}Cl_2F_3N_2O_{13}S$ calcd for [M–H]$^-$ 805.1429.

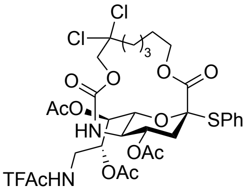

Compound 28

To a solution of PPh_3 (1.52 g, 5.79 mmol) in THF (659 mL), a solution of compound **27** (1.17 g, 1.45 mmol) and DMEAD (1.36 g, 5.79 mmol) in THF (181 mL) were added at ambiente temperature over a period of 2 h via cannula, followed by washing the flask containing compound **27** and DMTST with THF (66 mL) and transfer to the reaction mixture. After stirring for 1 h at ambient temperature as the progress of the reaction was monitored by TLC (*n*-hexane/acetone = 2/1, $CHCl_3$/MeOH = 3/1), the reaction was quenched with MeOH and AcOH and the mixture was concentrated. The residue was purified by silica gel column chromatography (*n*-hexane/acetone = 4/1 → 3/1) to give compound **28** (1.04 g, 91%) as a white foam; $[\alpha]_D$ +19.5° (c 1.0, $CHCl_3$); ^{1}H NMR (500 MHz, CD_3NO_2, 90 °C) δ 7.58–7.37 (m, 5 H, Ph), 7.09 (br s, 1 H, $CF_3C(=O)NH$), 5.49 (td, 1 H, $J_{3eq,4}$ = 5.0 Hz, $J_{3ax,4}$ = $J_{4,5}$ = 10.7 Hz, H-4), 5.41 (br s, 1 H, NH-5), 5.37 (br d, 1 H, $J_{7,8}$ = 5.9 Hz, H-7), 5.18 (td, 1 H, $J_{8,9a}$ = 3.2 Hz, $J_{8,9b}$ = 5.8 Hz, H-8), 4.62 (d, 1 H, J_{gem} = 12.0 Hz, NHC(=O)OCH_2), 4.49–4.44 (m, 3 H, H-6, NHC(=O)OCH_2, C(=O)OCH_2), 4.00 (m, 1 H, C(=O)OCH_2), 3.92 (m, 1 H, H-9a), 3.58 (near dt, 1 H, J_{gem} = 14.5 Hz, H-9b), 3.01 (dd, 1 H, J_{gem} = 13.0 Hz, H-3*eq*), 2.95 (td, 1 H, $J_{5,NH}$ = 6.8 Hz, $J_{5,6}$ = 10.4 Hz, H-5), 2.43 (m, 1 H, $CCl_2CH_2CH_2$), 2.26 (m, 1 H, $CCl_2CH_2CH_2$), 2.21 (s, 3 H, Ac), 2.00 (s, 6 H, 2 Ac), 1.89–1.83 (m, 3 H, H-3*ax*, CH_2), 1.65–1.56 (m, 4 H, 2 CH_2); ^{13}C NMR (125 MHz, CD_3NO_2, 90 °C) δ 172.7, 171.7, 171.6, 169.6, 158.9, 158.6, 155.6, 138.0, 131.4, 130.4, 130.3, 116.5, 91.4, 88.7, 74.7, 72.2, 71.6, 70.9, 69.2, 66.6, 54.6, 46.6, 41.1, 39.8, 28.3, 25.9, 24.1, 21.0, 20.8; HRMS (ESI) *m/z*: found $[M+Na]^+$ 811.1288, $C_{31}H_{37}Cl_2F_3N_2O_{12}S$ calcd for $[M+Na]^+$ 811.1289.

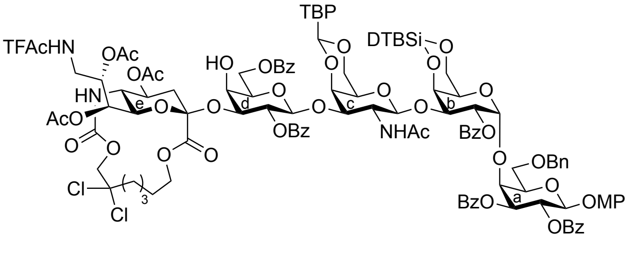

Compound 30

To a solution of donor **28** (297 mg, 0.376 mmol) and acceptor **23** (214 mg, 0.125 mmol) in CH_2Cl_2 (10 mL) were added NIS (101 mg, 0.451 mmol) and 3 Å molecular sieves (511 mg). After stirring for 1 h at ambient temperature, TfOH (2.2 µL, 25.0 µmol) was added at –50 °C. After stirring for 10 h at

–50 °C as the progress of the reaction was monitored by TLC (*n*-hexane/$CHCl_3$/acetone = 2/1/1, developed twice; $CHCl_3$/acetone = 7/1, developed twice), the reaction mixture was neutralized with satd. aq. $NaHCO_3$ and filtered through a pad of Celite. The pad was washed with $CHCl_3$. The combined filtrate and washings were diluted with $CHCl_3$ and washed with satd. aq. $Na_2S_2O_3$ and brine. The organic layer was dried over Na_2SO_4 and concentrated. The residue was purified by gel filtration (LH20; $CHCl_3$/MeOH = 1/1), then silica gel column chromatography (*n*-hexane/$CHCl_3$/acetone = 2/1/1) to give compound **30** (223 mg, 75%) as a white amorphous solid; $[\alpha]_D$ +60.7° (c 1.0, $CHCl_3$); ^{1}H NMR (500 MHz, CD_3NO_2, 80 °C) δ 8.14–6.81 (m, 39 H, 8 Ar, CF_3C(=O)NH), 5.96 (dd, 1 H, $J_{1,2}$ = 8.0 Hz, $J_{2,3}$ = 10.7 Hz, H-2^{a}), 5.71 (dd, 1 H, $J_{1,2}$ = 3.5 Hz, $J_{2,3}$ = 10.5 Hz, H-2^{b}), 5.47 (dd, 1 H, $J_{3,4}$ = 2.8 Hz, H-3^{a}), 5.43 (s, 1 H, ArC*H*<), 5.37–5.27 (m, 7 H, H-1^{a}, H-1^{b}, H-4^{e}, H-7^{e}, H-8^{e}, NH-2^{c}, NH-5^{e}), 5.23 (near t, 1 H, H-2^{d}), 5.03 (br s, 1 H, H-1^{c}), 4.89 (br s, 1 H, H-4^{c}), 4.87 (d, 1 H, $J_{1,2}$ = 8.0 Hz, H-1^{d}), 4.74 (dd, 1 H, $J_{5,6a}$ = 7.0 Hz, J_{gem} = 11.5 Hz, H-6a^{d}), 4.61–4.22 (m, 12 H, H-4^{a}, H-3^{b}, H-4^{b}, H-3^{c}, H-3^{d}, H-6b^{d}, H-6^{e}, 2 PhCH_2, 2 NHCO(=O)CH_2, C(=O)OCH_2), 4.18-4.10 (m, 4 H, H-5^{a}, H-5^{b}, H-6a^{c}, H-5^{d}), 4.06 (br s, 1 H, H-4^{d}), 3.96–3.88 (m, 2 H, H-9a^{e}, C(=O)OCH_2), 3.80–3.77 (m, 4 H, H-6b^{c}, OMe), 3.71–3.61 (m, 3 H, H-6a^{a}, H-6b^{a}, H-2^{c}), 3.54 (d, 1 H, J_{gem} = 12.8 Hz, H-6a^{b}), 3.48 (d, 1 H, H-6b^{b}), 3.41 (s, 1 H, H-5^{c}), 3.33 (dt, 1 H, $J_{8,9b}$ = $J_{9b,NH}$ = 5.9 Hz, J_{gem} = 14.5 Hz, H-9b^{e}), 2.97 (d, 1 H, $J_{4,OH}$ = 3.5 Hz, OH-4^{d}), 2.85 (td, 1 H, $J_{5,NH}$ = 6.8 Hz, $J_{4,5}$ = $J_{5,6}$ = 10.3 Hz, H-5^{e}), 2.65 (dd, 1 H, $J_{3eq,4}$ = 5.3 Hz, J_{gem} = 12.8 Hz, H-3*eq*^{e}), 2.36 (m, 1 H, $CCl_2CH_2CH_2$), 2.22 (m, 1 H, $CCl_2CH_2CH_2$), 2.08 (s, 3 H, Ac), 1.93 (s, 3 H, Ac), 1.91 (s, 3 H, Ac), 1.79–1.69 (m, 3 H, H-3*ax*^{e}, CH_2^{e}), 1.63–1.49 (m, 4 H, 2 CH_2^{e}), 1.32 (s, 9 H, ^{t}Bu), 1.04 (s, 9 H, ^{t}Bu), 1.01 (s, 9 H, ^{t}Bu), 0.91 (m, 3 H, Ac); ^{13}C NMR (125 MHz, CD_3NO_2, 80 °C) δ 172.8, 172.1, 171.4, 169.4, 167.8, 167.5, 167.3, 167.2, 166.7, 159.2, 158.9, 157.6, 155.7, 153.8, 152.8, 139.8, 137.4, 135.0, 134.8, 134.7, 134.6, 131.9, 131.8, 131.6, 131.4, 131.2, 131.1, 130.9, 130.8, 130.1, 130.1, 129.9, 129.7, 129.0, 129.0, 127.7, 126.3, 120.4, 116.4, 116.2, 103.7, 103.4, 102.5, 99.7, 99.3, 91.4, 79.1, 77.0, 75.7, 75.6, 75.4, 75.2, 75.0, 74.4, 73.8, 72.3, 71.9, 71.4, 70.6, 70.2, 69.9, 69.7, 69.0, 68.2, 68.0, 66.7, 65.2, 56.6, 54.2, 46.3, 41.1, 38.1, 35.6, 31.8, 29.9, 28.4, 28.2, 28.1, 25.5, 24.3, 23.9, 23.2, 21.6, 21.4, 20.9, 20.8; HRMS (ESI) *m/z*: found $[M+Na]^+$ 2408.7742, $C_{119}H_{136}Cl_2F_3N_3O_{39}Si$ calcd for $[M+Na]^+$ 2408.7742.

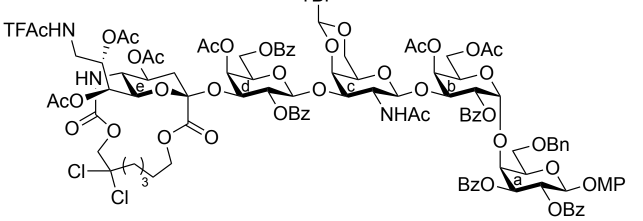

Compound 31

To a solution of compound **30** (315 mg, 0.132 mmol) in THF (1.3 mL) was added 1 M TBAHF in THF (1.32 mL, 1.32 mmol). After stirring for 9 h at ambient temperature as the progress of the reaction was monitored by TLC (*n*-hexane/acetone = 1/1), the reaction mixture was diluted with $CHCl_3$ and washed with 2 M HCl, H_2O, satd. aq. $NaHCO_3$ and brine. The organic layer was dried over Na_2SO_4, concentrated and dried for 2 h. The residue was dissolved in pyridine (1.3 mL), followed by the addition of Ac_2O (50.0 µL, 0.528 mmol) and DMAP (1.6 mg, 13.2 µmol) at 0 °C. After stirring for 3.5 h at ambient temperature as the progress of the reaction was monitored by TLC (*n*-hexane/acetone = 1/1), the reaction was quenched with MeOH at 0 °C and diluted with $CHCl_3$. The organic layer was washed with 2 M HCl, H_2O, satd. aq. $NaHCO_3$ and brine, dried over Na_2SO_4, and concentrated. The residue was purified by silica gel column chromatography (*n*-hexane/$CHCl_3$/acetone = 3/2/2) to give compound **31** (303 mg, 97%) as a white amorphous solid; $[\alpha]_D$ +42.3° (c 1.0, $CHCl_3$); ^{1}H NMR (500 MHz, CD_3NO_2, 80 °C) δ 8.17–6.78 (m, 39 H, 8 Ar, $CF_3C(=O)NH$), 6.01 (dd, 1 H, $J_{1,2}$ = 8.0 Hz, $J_{2,3}$ = 10.5 Hz, H-2^a), 5.58 (dd, 1 H, $J_{3,4}$ = 3.0 Hz, H-3^a), 5.54 (d, 1 H, $J_{3,4}$ = 3.0 Hz, H-4^b), 5.48–5.44 (m, 3 H, H-2^b, H-8^e, ArC*H*<), 5.39–5.34 (m, 5 H, H-1^a, H-1^b, H-4^d, H-4^e, NH-5^e), 5.24–5.20 (m, 2 H, H-2^d, H-7^e), 4.97 (d, 1 H, $J_{1,2}$ = 8.0 Hz, H-1^d), 4.90 (br d, 1 H, $J_{1,2}$ = 8.0 Hz, H-1^c), 4.83 (dd, 1 H, $J_{3,4}$ = 3.0 Hz, $J_{2,3}$ = 10.0 Hz, H-3^d), 4.70–4.04 (m, 17 H, H-4^a, H-5^a, H-3^b, H-5^b, H-3^c, H-4^c, H-6a^c, H-5^d, H-6a^d, H-6b^d, H-6^e, 2 $PhCH_2$, 2 $NHCO(=O)CH_2$, 2 $C(=O)OCH_2$), 3.91 (br d, 1 H, J_{gem} = 14.8 Hz, H-9a^e), 3.83 (d, 1 H, J_{gem} = 13.5 Hz, H-6b^c), 3.76 (s, 3 H, OMe), 3.71–3.62 (m, 5 H, H-6a^a, H-6b^a, H-6a^b, H-6b^b, H-2^c), 3.44 (s, 1 H, H-5^c), 3.25 (near d, 1 H, H-9b^e), 2.80 (t, 1 H, $J_{4,5}$ = $J_{5,6}$ = 10.3 Hz, H-5^e), 2.64 (dd, 1 H, $J_{3eq,4}$ = 4.0 Hz, J_{gem} = 12.0 Hz, H-3*eq*^{e}), 2.32 (m, 1 H, $CCl_2CH_2CH_2$), 2.26 (m, 1 H, $CCl_2CH_2CH_2$), 2.13 (s, 3 H, Ac), 2.11 (s, 3 H, Ac), 2.08 (s, 3 H, Ac), 1.94 (s, 3 H, Ac), 1.84–1.46 (m, 10 H, H-3*ax*^{e}, 3 CH_2^{e}, Ac), 1.32 (s, 9 H, ^{t}Bu), 1.00 (m, 3 H, Ac); ^{13}C NMR (125 MHz, CD_3NO_2, 80 °C) δ 173.1, 172.6, 172.2, 171.8, 169.5, 167.8, 167.7, 167.5, 167.0, 157.8, 154.1, 153.2, 140.1, 137.7, 135.3, 135.1, 135.0, 132.0, 131.9, 131.6, 131.3, 131.3, 131.1, 130.3, 130.2, 130.0, 129.3, 129.2, 127.9, 126.7, 120.5, 116.5, 104.0, 102.7, 98.4, 91.8, 79.9, 77.0, 75.9, 75.3, 74.7, 74.5, 73.6, 72.9, 72.5, 72.3, 72.2, 72.1, 70.5, 70.4, 70.1, 69.6, 68.6, 67.1, 64.4, 56.9, 41.1, 39.0, 35.9, 32.1, 28.5, 25.9, 24.4, 21.9, 21.3, 21.2, 21.1, 21.0; HRMS (ESI) *m/z*: found $[M+Na]^+$ 2394.7036, $C_{117}H_{126}Cl_2F_3N_3O_{42}$ calcd for $[M+Na]^+$ 2394.7037.

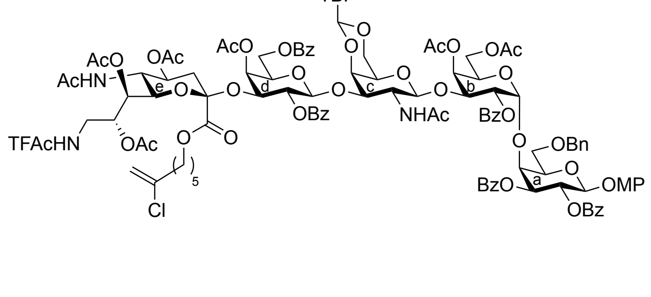

Compound 32

To a solution of compound **31** (282 mg, 0.119 mmol) in MeCN/AcOH = 4/1 (12 mL) were added Ac_2O (2.4 mL) and zinc powder (3.11 g, <50 nm particle size). After stirring for 2.5 h at ambient temperature as the progress of the reaction was monitored by TLC ($CHCl_3$/MeOH = 30/1, developed twice; toluene/acetone = 2/1), the reaction mixture was filtered through a pad of Celite. The pad was washed with $CHCl_3$. The combined filtrate and washings were diluted with $CHCl_3$, washed with satd. aq. $NaHCO_3$ and brine, dried over Na_2SO_4, and concentrated. The residue was purified by silica gel column chromatography (toluene/acetone = 4/1 → 3/1) to give compound **32** (249 mg, 90%) as a white amorphous solid; $[\alpha]_D$ +72.8° (c 1.0, $CHCl_3$); ^{1}H NMR (500 MHz, CD_3CN) δ 8.11–6.79 (m, 39 H, 8 Ar, CF_3C(=O)NH), 6.01 (d, 1 H, $J_{5,NH}$ = 9.5 Hz, NH-5^e), 5.94 (dd, 1 H, $J_{1,2}$ = 7.5 Hz, $J_{2,3}$ = 10.5 Hz, H-2^a), 5.82 (d, 1 H, $J_{2,NH}$ = 9.0 Hz, NH-2^c), 5.61 (dd, 1 H, $J_{3,4}$ = 3.0 Hz, H-3^a), 5.47 (d, 1 H, $J_{3,4}$ = 3.0 Hz, H-4^b), 5.42 (m, 1 H, H-8^e), 5.38–5.34 (m, 3 H, H-1^a, H-2^b, ArC*H*<), 5.27 (d, 1 H, $J_{3,4}$ = 3.0 Hz, H-4^d), 5.22–5.21 (m, 2 H, H-1^b, C(=CH_2)Cl), 5.16 (d, 1 H, J_{gem} = 1.0 Hz, C(=CH_2)Cl), 5.13–5.09 (m, 2 H, H-2^d, H-7^e), 4.85 (d, 1 H, $J_{1,2}$ = 8.0 Hz, H-1^d), 4.83–4.76 (m, 3 H, H-1^c, H-3^d, H-4^e), 4.57 (near t, 1 H, H-5^a), 4.53–4.49 (m, 2 H, H-3^b, H-6a^d), 4.46 (d, 1 H, H-4^a), 4.27–4.22 (m, 5 H, H-4^c, H-5^d, H-6b^d, PhCH_2, C(=O)OCH_2), 4.20 (d, 1 H, J_{gem} = 11.5 Hz, PhCH_2), 4.14–4.11 (m, 2 H, H-5^b, H-6a^c), 4.02 (dt, 1 H, C(=O)OCH_2), 3.96 (br d, 1 H, H-3^c), 3.86 (near q, 1 H, H-5^e), 3.78–3.70 (m, 6 H, H-6b^c, H-6^e, H-9a^e, OMe), 3.61–3.47 (m, 5 H, H-6a^a, H-6b^a, H-6a^b, H-6b^b, H-2^c), 3.33 (s, 1 H, H-5^c), 3.10 (dt, 1 H, $J_{8,9b}$ = $J_{9b,NH}$ = 6.3 Hz, J_{gem} = 15.0 Hz, H-9b^e), 2.42 (dd, 1 H, $J_{3eq,4}$ = 4.8 Hz, J_{gem} = 12.8 Hz, H-3*eq*^{e}), 3.47 (t, 2 H, C(=CH_2)ClCH_2), 2.12 (s, 3 H, Ac), 2.06 (s, 3 H, Ac), 2.06 (s, 3 H, Ac), 1.87 (s, 3 H, Ac), 1.81 (s, 3 H, Ac), 1.76 (s, 3 H, Ac), 1.72 (s, 3 H, Ac), 1.65 (m, 2 H, C(=O)OCH_2CH_2), 1.58–1.48 (m, 3 H, H-3*ax*^{e}, C(=CH_2)CH_2CH_2), 1.43–1.30 (m, 11 H, C(=O)OCH_2CH_2CH_2, ^{t}Bu), 0.81 (s, 3 H, Ac); ^{13}C NMR (125 MHz, CD_3CN) δ 172.0, 171.6, 171.2, 171.1, 170.8, 170.7, 170.7, 170.5, 168.3, 166.7, 166.6, 166.4, 166.4, 166.1, 158.6, 158.3, 158.1, 156.6, 153.0, 152.0, 143.9, 139.0, 136.8, 134.7, 134.6, 134.5, 134.3, 134.3, 131.1, 131.0, 131.0, 130.9, 130.6, 130.5, 130.5, 130.3, 130.3, 130.1, 129.7, 129.7, 129.6, 129.6, 129.5, 129.3, 128.6, 128.5, 127.1, 126.1, 119.4, 118.6, 115.9, 115.5, 113.0, 103.5, 102.0, 101.6, 101.0, 98.4, 97.3, 79.3, 79.1, 76.0, 74.6, 74.0, 73.7, 73.5, 72.8, 72.0, 71.9, 71.2, 70.9, 70.4, 70.2, 69.7, 69.4, 68.8, 68.2, 67.3, 67.2, 63.4, 62.9, 56.2, 54.6, 51.9, 49.1, 40.7, 39.3, 38.0, 35.3, 31.5, 28.5, 27.5, 25.4, 23.0, 22.5, 21.6, 21.4, 21.3, 21.2, 21.0, 20.9, 20.7; HRMS (ESI) *m/z*: found $[M+Na]^+$ 2358.7633, $C_{118}H_{129}ClF_3N_3O_{41}$ calcd for $[M+Na]^+$ 2358.7634.

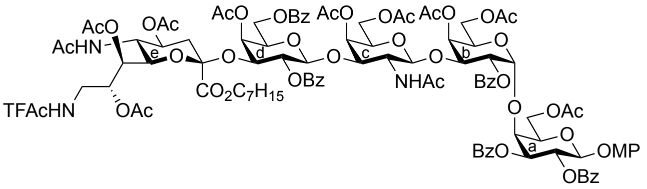

Compound 33

To a solution of compound **32** (37.9 mg, 16.2 µmol) in 1,4-dioxane/MeOH = 1/1 (0.80 mL) was added $Pd(OH)_2$-C (75.8 mg, 20% on carbon). After stirring for 3.5 h at ambient temperature under H_2 gas as the progress of the reaction was monitored by TLC ($CHCl_3$/MeOH =20/1), the reaction mixture was filtered through a pad of Celite, the pad was washed with $CHCl_3$, the combined filtrate and washings were concentrated, and dried for 2 h. The residue was dissolved in pyridine (0.32 mL), followed by the addition of Ac_2O (7.7 µL, 81.0 µmol) and DMAP (0.2 mg, 1.62 µmol) at 0 °C. After stirring for 55 h at ambient temperature, the reaction was quenched with MeOH at 0 °C, and the mixture was co-evaporated with toluene, and diluted with $CHCl_3$. The organic layer was washed with 2 M HCl, H_2O, satd. aq. $NaHCO_3$ and brine, dried over Na_2SO_4, and concentrated. The residue was purified by silica gel column chromatography ($CHCl_3$/MeOH = 100/1) to give compound **33** (29.1 mg, 82%) as a white amorphous solid; $[\alpha]_D$ +58.7° (c 1.0, $CHCl_3$); ^{1}H NMR (500 MHz, CD_3CN) δ 7.97–6.74 (m, 30 H, 6 Ar, $CF_3C(=O)NH$), 5.87 (d, 1 H, $J_{5,NH}$ = 9.5 Hz, NH-5^e), 5.75 (dd, 1 H, $J_{1,2}$ = 7.5 Hz, $J_{2,3}$ = 10.4 Hz, H-2^a), 5.58 (d, 1 H, $J_{2,NH}$ = 9.0 Hz, NH-2^c), 5.46 (dd, 1 H, $J_{3,4}$ = 2.9 Hz, H-3^a), 5.41 (d, 1 H, $J_{3,4}$ = 3.0 Hz, H-4^b), 5.27–5.21 (m, 4 H, H-1^a, H-2^b, H-4^c, H-8^e), 5.12 (d, 1 H, $J_{3,4}$ = 3.5 Hz, H-4^d), 5.10 (d, 1 H, $J_{1,2}$ = 3.5 Hz, H-1^b), 4.95–4.90 (m, 2 H, H-2^d, H-7^e), 4.74 (d, 1 H, $J_{1,2}$ = 8.0 Hz, H-1^d), 4.68–4.63 (m, 2 H, H-3^d, H-4^e), 4.60 (d, 1 H, $J_{1,2}$ = 8.5 Hz, H-1^c), 4.46 (near t, 1 H, H-5^c), 4.34 (dd, 1 H, $J_{2,3}$ = 10.5 Hz, H-3^b), 4.32 (d, 1 H, H-4^a), 4.29 (dd, 1 H, $J_{5,6a}$ = 5.5 Hz, J_{gem} = 10.0 Hz, H-6a^d), 4.23 (dd, 1 H, $J_{5,6a}$ = 7.5 Hz, J_{gem} = 11.0 Hz, H-6a^a), 4.09 (dt, 1 H, $C(=O)OCH_2$), 4.05–3.96 (m, 4 H, H-5^a, H-3^c, H-5^d, H-6b^d), 3.92–3.81 (m, 4 H, H-6b^a, H-6a^b, H-6^e, $C(=O)OCH_2$), 3.71–3.55 (m, 9 H, H-5^b, H-6b^b, H-2^c, H-6a^c, H-5^e, H-9a^e, OMe), 3.51 (dd, 1 H, $J_{5,6b}$ = 6.5 Hz, J_{gem} = 11.5 Hz, H-6b^c), 2.90 (dt, 1 H, $J_{8,9b}$ = $J_{9b,NH}$ = 5.8 Hz, J_{gem} = 15.0 Hz, H-9b^e), 2.30 (dd, 1 H, $J_{3eq,4}$ = 4.5 Hz, J_{gem} = 12.5 Hz, H-3*eq*^{e}), 1.99 (s, 3 H, Ac), 1.95 (s, 3 H, Ac), 1.94 (s, 3 H, Ac), 1.92 (s, 3 H, Ac), 1.92 (s, 3 H, Ac), 1.79 (s, 3 H, Ac), 1.76 (s, 3 H, Ac), 1.71 (s, 3 H, Ac), 1.58 (s, 3 H, Ac), 1.58 (s, 3 H, Ac), 1.49 (t, 2 H, $C(=O)OCH_2CH_2$), 1.35 (t, 1 H, $J_{3ax,4}$ = 12.3 Hz, H-3*ax*^{e}), 1.21–1.16 (m, 8 H, $C(=O)OCH_2CH_2CH_2$, $CH_2CH_2CH_2Me$, CH_2CH_2Me, CH_2Me), 0.79 (t, 3 H, CH_2Me), 0.60 (s, 3 H, Ac); ^{13}C NMR (125 MHz, CD_3CN) δ 172.1, 171.5, 171.4, 171.0, 171.0, 170.9, 170.9, 170.8, 170.6, 170.6, 170.2, 168.3, 166.5, 166.5, 166.4, 166.2, 165.9, 158.5, 158.2, 156.6, 151.9, 134.7, 134.6, 134.4, 134.3, 134.3, 131.0, 130.9, 130.9, 130.6, 130.4, 130.3, 130.1, 129.6, 129.6, 129.6, 119.1, 118.6, 115.9, 115.5, 102.5, 102.2, 100.9, 98.7, 98.2, 79.1, 78.0, 75.5, 73.9, 73.8, 73.5, 72.7, 72.2, 72.0, 71.5, 71.4, 71.0, 70.9, 70.2,

70.0, 69.1, 68.8, 68.7, 67.5, 63.6, 62.9, 62.7, 62.3, 56.2, 52.4, 49.0, 40.3, 38.0, 32.3, 29.6, 28.8, 26.4, 23.3, 23.0, 22.2, 21.5, 21.2, 21.1, 21.1, 20.9, 20.9, 20.9, 20.8, 20.8, 14.3; HRMS (ESI) *m/z*: found $[M+Na]^+$ 2218.7088, $C_{106}H_{120}F_3N_3O_{44}$ calcd for $[M+Na]^+$ 2218.7089.

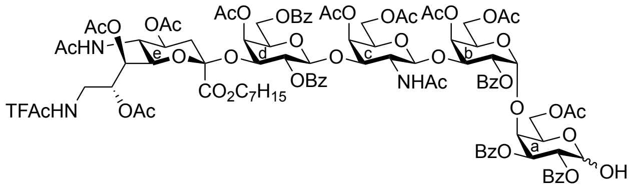

Compound 34

To a solution of compound **33** (28.0 mg, 12.7 μmol) in MeCN/toluene/H_2O = 6/5/3 (0.26 mL) was added cerium (IV) ammonium nitrate (69.6 mg, 0.127 mmol) at 0 °C. After stirring for 3 h at 0 °C as the progress of the reaction was monitored by TLC ($CHCl_3$/MeOH = 30/1, developed twice), the reaction mixture was diluted with EtOAc. The organic layer was washed with H_2O, satd. aq. $NaHCO_3$ and brine, dried over Na_2SO_4, and concentrated. The residue was purified by silica gel column chromatography ($CHCl_3$/MeOH = 60/1 → 50/1) to give compound **34** (17.4 mg, 65%, α:β = 1:0.3) as a white amorphous solid; α isomer; ^{1}H NMR (500 MHz, CD_3CN) δ 8.05–7.35 (m, 26 H, 5 Ph, $CF_3C(=O)NH$), 5.94 (d, 1 H, $J_{5,NH}$ = 9.5 Hz, NH-5^e), 5.78 (dd, 1 H, $J_{2,3}$ = 10.5 Hz, $J_{3,4}$ = 3.0 Hz, H-3^a), 5.64 (d, 1 H, $J_{2,NH}$ = 9.0 Hz, NH-2^c), 5.55 (t, 1 H, $J_{1,2}$ = $J_{1,OH}$ = 3.8 Hz, H-1^a), 5.49–5.45 (m, 2 H, H-2^a, H-4^b), 5.36–5.32 (m, 2 H, H-4^c, H-8^e), 5.28–5.21 (m, 3 H, H-1^b, H-2^b, H-4^d), 5.04–4.98 (m, 2 H, H-2^d, H-7^e), 4.82 (d, 1 H, $J_{1,2}$ = 8.0 Hz, H-1^d), 4.76–4.69 (m, 4 H, H-1^c, H-3^d, H-4^e, OH-1^a), 4.53 (near t, 1 H, H-5^c), 4.43–4.32 (m, 3 H, H-4^a, H-3^b, H-6a^d), 4.23 (dd, 1 H, $J_{5,6a}$ = 7.5 Hz, J_{gem} = 11.0 Hz, H-6a^a), 4.17 (dt, 1 H, $C(=O)OCH_2$), 4.12–3.90 (m, 7 H, H-5^a, H-6b^a, H-6a^b, H-3^c, H-5^d, H-6b^d, $C(=O)OCH_2$), 3.79–3.59 (m, 8 H, H-5^b, H-6b^b, H-2^c, H-6a^c, H-6b^c, H-5^e, H-6^e, H-9a^e), 2.99 (dt, 1 H, $J_{8,9b}$ = $J_{9b,NH}$ = 5.9 Hz, J_{gem} = 15.0 Hz, H-9b^e), 2.38 (dd, 1 H, $J_{3eq,4}$ = 4.5 Hz, J_{gem} = 12.4 Hz, H-3*eq*^{e}), 2.11–1.58 (m, 30 H, 10 Ac), 1.57 (m, 2 H, $C(=O)OCH_2CH_2$), 1.44 (t, 1 H, $J_{3ax,4}$ = 12.3 Hz, H-3*ax*^{e}), 1.27–1.24 (m, 8 H, $C(=O)OCH_2CH_2CH_2$, $CH_2CH_2CH_2Me$, CH_2CH_2Me, CH_2Me), 0.86 (t, 3 H, CH_2Me), 0.69 (s, 3 H, Ac); HRMS (ESI) *m/z*: found $[M+Na]^+$ 2112.6671, $C_{99}H_{114}F_3N_3O_{43}$ calcd for $[M+Na]^+$ 2112.6670.

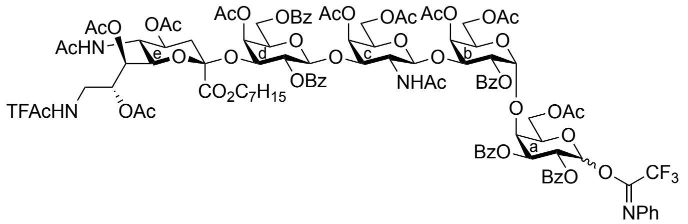

Compound 35

To a solution of compound **34** (72.5 mg, 34.7 µmol) in acetone (0.69 mL) were added $CF_3C(=NPh)Cl$ (11.2 µL, 69.3 µmol) and K_2CO_3 (23.9 mg, 0.173 mmol). After stirring for 10 h at ambient temperature as the progress of the reaction was monitored by TLC ($CHCl_3$/MeOH = 20/1), the reaction mixture was concentrated. The residue was purified by silica gel column chromatography ($CHCl_3$/MeOH = 80/1) to give compound **35** (73.6 mg, 94%, α:β = 1:0.3) as a white amorphous solid; α isomer; ^{1}H NMR (500 MHz, CD_3CN) δ 8.05–6.54 (m, 31 H, 6 Ph, $CF_3C(=O)NH$), 6.13 (br s, 1 H, H-1^a), 5.95 (d, 1 H, $J_{5,NH}$ = 9.5 Hz, NH-5^e), 5.88 (near d, 1 H, H-3^a), 5.73 (br d, 1 H, H-2^a), 5.67 (d, 1 H, $J_{2,NH}$ = 9.0 Hz, NH-2^c), 5.47 (d, 1 H, $J_{3,4}$ = 3.5 Hz, H-4^b), 5.35–5.32 (m, 2 H, H-4^c, H-8^e), 5.29–5.26 (m, 2 H, H-1^b, H-2^b), 5.19 (d, 1 H, $J_{3,4}$ = 2.5 Hz, H-4^d), 5.02–4.98 (m, 2 H, H-2^d, H-7^e), 4.81 (d, 1 H, $J_{1,2}$ = 8.0 Hz, H-1^d), 4.75–4.66 (m, 3 H, H-1^c, H-3^d, H-4^e), 4.55 (near t, 1 H, H-5^c), 4.45–4.28 (m, 5 H, H-4^a, H-6a^a, H-6b^a, H-3^b, H-6a^d), 4.16 (dt, 1 H, $C(=O)OCH_2$^{e}), 4.11–4.04 (m, 3 H, H-3^c, H-5^d, H-6b^d), 3.98–3.88 (m, 4 H, H-5^a, H-6a^b, H-6b^b, $C(=O)OCH_2$), 3.79–3.60 (m, 7 H, H-5^b, H-2^c, H-6a^c, H-6b^c, H-5^e, H-6^e, H-9a^e), 2.98 (dt, 1 H, $J_{8,9b}$ = $J_{9b,NH}$ = 6.0 Hz, J_{gem} = 14.5 Hz, H-9b^e), 2.38 (dd, 1 H, $J_{3eq,4}$ = 5.0 Hz, J_{gem} = 12.3 Hz, H-3*eq*^{e}), 2.14–1.55 (m, 32 H, 10 Ac, $C(=O)OCH_2CH_2$), 1.41 (t, 1 H, $J_{3ax,4}$ = 12.0 Hz, H-3*ax*^{e}), 1.26–1.20 (m, 8 CH_2^{e}), 0.86 (t, 3 H, CH_2Me^{Cer}), 0.63 (s, 3 H, Ac); HRMS (ESI) *m/z*: found $[M+Na]^+$ 2283.6966, $C_{107}H_{118}F_6N_4O_{43}$ calcd for $[M+Na]^+$ 2283.6966.

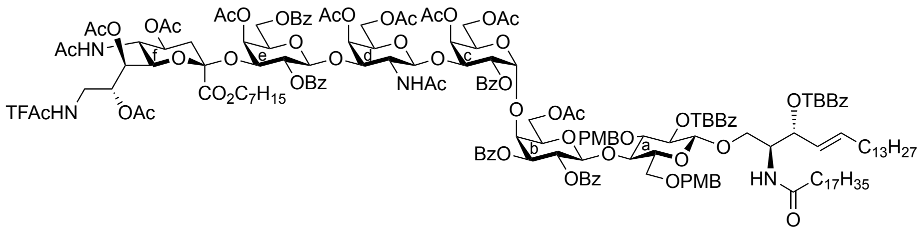

Compound 36

To a solution of donor **35** (127 mg, 56.1 µmol) and acceptor **17**[S03] (108 mg, 84.1 µmol) in CH_2Cl_2 (2.8 mL) was added 4 Å molecular sieves (AW-300, 280 mg). After stirring for 1 h at ambient temperature, TMSOTf (2.0 µL, 11.2 µmol) was added at 0 °C. After stirring for 8 h at 0 °C as the progress of the reaction was monitored by TLC (toluene/acetone = 5/2), the reaction mixture was neutralized with satd. aq. $NaHCO_3$ and filtered through a pad of Celite. The pad was washed with $CHCl_3$. The combined filtrate and washings were diluted with $CHCl_3$ and washed with brine. The organic layer was dried over Na_2SO_4 and concentrated. The residue was purified by silica gel column chromatography (toluene/acetone = 10/1 → 4/1) to give compound **36** (129 mg, 68%) as a white amorphous solid; $[\alpha]_D$ +65.7° (c 1.0, $CHCl_3$); ^{1}H NMR (500 MHz, $CDCl_3$) δ 8.05–6.67 (m, 42 H, 9 Ar, $CF_3C(=O)NH$), 5.78

(near quin, 1 H, $J_{5,6a}$ = $J_{5,6b}$ = 6.5 Hz, $J_{4,5}$ = 15.4 Hz, H-5^{Cer}), 5.68–5.64 (m, 2 H, H-2^{b}, NH-2^{Cer}), 5.46 (t, 1 H, $J_{2,3}$ = $J_{3,4}$ = 7.3 Hz, H-3^{Cer}), 5.42 (d, 1 H, $J_{3,4}$ = 2.5 Hz, H-4^{c}), 5.39 (dd, 1 H, H-4^{Cer}), 5.34–5.30 (m, 2 H, H-2^{c}, H-8^{f}), 5.28 (d, 1 H, $J_{3,4}$ = 3.0 Hz, H-4^{d}), 5.17–5.09 (m, 5 H, H-2^{a}, H-3^{b}, H-1^{c}, H-2^{e}, H-4^{e}), 5.02–5.00 (m, 2 H, NH-2^{d}, NH-5^{f}), 4.95–4.89 (m, 3 H, H-1^{b}, H-1^{d}, H-7^{f}), 4.80–4.75 (m, 2 H, H-1^{e}, H-4^{f}), 4.66 (d, 1 H, J_{gem} = 11.3 Hz, ArCH_2), 4.60 (d, 1 H, ArCH_2), 4.58 (dd, 1 H, $J_{3,4}$ = 3.5 Hz, $J_{2,3}$ = 10.0 Hz, H-3^{e}), 4.45–4.32 (m, 6 H, H-1^{a}, H-3^{d}, H-5^{d}, H-6a^{e}, H-2^{Cer}, ArCH_2), 4.30–4.27 (m, 2 H, H-3^{c}, H-5^{c}), 4.23 (d, 1 H, $J_{3,4}$ = 2.5 Hz, H-4^{b}), 4.20–3.90 (m, 11 H, H-4^{a}, H-6^{b}, H-5^{e}, H-6b^{e}, H-5^{f}, H-9a^{f}, H-1a^{Cer}, H-1b^{Cer}, ArCH_2, C(=O)OCH_2), 3.83–3.69 (m, 9 H, H-3^{a}, H-6b^{b}, H-6a^{d}, 2 OMe), 3.65–3.58 (m, 2 H, H-6a^{c}, H-6b^{d}), 3.52–3.37 (m, 5 H, H-6a^{a}, H-6b^{a}, H-5^{b}, H-6b^{c}, H-6^{f}), 3.26 (br d, 1 H, H-5^{a}), 3.11 (m, 1 H, H-2^{d}), 2.73 (near dt, 1 H, H-9b^{f}), 2.47 (dd, 1 H, $J_{3eq,4}$ = 4.5 Hz, J_{gem} = 12.5 Hz, H-3*eq*^{f}), 2.09 (s, 3 H, Ac), 2.06 (s, 3 H, Ac), 2.03 (s, 3 H, Ac), 1.99 (s, 3 H, Ac), 1.99 (s, 3 H, Ac), 1.95–1.92 (m, 5 H, H-6a^{Cer}, H-6b^{Cer}, Ac), 1.82 (s, 3 H, Ac), 1.81 (s, 3 H, Ac), 1.70–1.58 (m, 8 H, H-3*ax*^{f}, C(=O)OCH$_2$CH_2), NHC(=O)CH_2, Ac), 1.38–1.08 (m, 78 H, 4 CH$_2^{f}$, 26 CH$_2^{Cer}$, 2 ^{t}Bu), 0.89–0.86 (m, 9 H, CH$_2$*Me*^{f}, 2 Me^{Cer}), 0.73 (s, 3 H, Ac); ^{13}C NMR (125 MHz, CDCl$_3$) δ 172.6, 172.5, 170.7, 170.5, 170.4, 170.3, 170.3, 170.3, 169.9, 169.9, 169.1, 167.3, 166.2, 165.8, 165.6, 165.3, 165.1, 165.0, 164.8, 159.3, 159.1, 158.1, 157.8, 157.0, 156.4, 137.1, 133.6, 133.4, 133.2, 133.1, 130.5, 130.2, 130.1, 130.0, 130.0, 129.9, 129.8, 129.6, 129.5, 129.4, 129.1, 128.9, 128.9, 128.8, 128.7, 128.6, 128.5, 128.5, 128.4, 128.3, 127.6, 126.8, 125.4, 125.3, 124.9, 117.0, 114.8, 113.9, 113.7, 101.1, 100.9, 100.6, 99.4, 98.3, 96.9, 79.2, 79.1, 77.6, 75.3, 74.8, 74.1, 74.0, 73.5, 73.4, 73.2, 72.3, 72.1, 71.8, 71.3, 71.2, 70.8, 70.6, 70.5, 70.3, 70.2, 70.1, 69.3, 68.8, 67.7, 67.5, 67.4, 67.3, 66.9, 66.7, 62.3, 61.3, 61.2, 61.0, 55.2, 55.2, 54.8, 50.4, 48.9, 38.6, 37.4, 36.4, 35.1, 35.0, 32.3, 31.9, 31.7, 31.1, 29.7, 29.7, 29.6, 29.6, 29.5, 29.5, 29.4, 29.3, 29.3, 29.0, 28.8, 28.1, 25.7, 25.6, 23.1, 22.7, 22.5, 22.0, 21.1, 20.8, 20.8, 20.7, 20.6, 20.5, 14.1, 14.1; HRMS (ESI) *m/z*: found [M/2+Na]$^+$ 1702.7669, $C_{179}H_{233}F_3N_4O_{54}$ calcd for [M/2+Na]$^+$ 1702.7673.

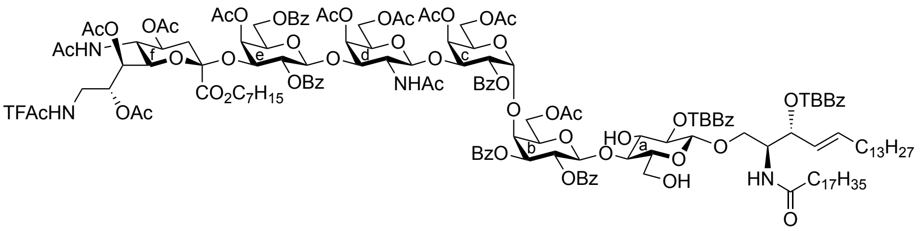

Compound 37

To a solution of compound **36** (167 mg, 49.7 μmol) in CH_2Cl_2 (3.3 mL) was added TFA (1.70 mL, 22.2 mmol) at 0 °C. After stirring for 30 min at 0 °C as the progress of the reaction was monitored by TLC (toluene/acetone = 3/1, developed twice), the reaction mixture was neutralized with satd. aq.

$NaHCO_3$ and diluted with $CHCl_3$. The organic layer was washed with satd. aq. $NaHCO_3$ and brine, dried over Na_2SO_4, and concentrated. The residue was purified by silica gel column chromatography (toluene/acetone = 4/1) to give compound **37** (149 mg, 96%) as a white amorphous solid; $[\alpha]_D$ +73.1° (c 1.2, $CHCl_3$); ^{1}H NMR (500 MHz, $CDCl_3$) δ 8.07–7.10 (m, 34 H, 7 Ar, CF_3C(=O)NH), 5.90 (near quin, 1 H, $J_{5,6a}$ = $J_{5,6b}$ = 7.0 Hz, $J_{4,5}$ = 15.0 Hz, H-5^{Cer}), 5.80 (d, 1 H, $J_{2,NH}$ = 10.0 Hz, NH-2^{Cer}), 5.77 (dd, 1 H, $J_{1,2}$ = 8.2 Hz, $J_{2,3}$ = 10.9 Hz, H-2^b), 5.59 (t, 1 H, $J_{2,3}$ = $J_{3,4}$ = 8.8 Hz, H-3^{Cer}), 5.49 (d, 1 H, $J_{3,4}$ = 2.5 Hz, H-4^c), 5.47–5.42 (m, 3 H, H-2^c, H-4^d, H-4^{Cer}), 5.34 (near dt, 1 H, $J_{7,8}$ = 10.0 Hz, H-8^f), 5.26 (dd, 1 H, $J_{3,4}$ = 2.5 Hz, H-3^b), 5.21–5.17 (m, 2 H, H-1^d, H-2^e), 5.15–5.07 (m, 4 H, H-2^a, H-4^e, NH-2^d, NH-5^f), 5.01 (d, 1 H, $J_{1,2}$ = 3.5 Hz, H-1^c), 4.92 (dd, 1 H, $J_{6,7}$ = 2.5 Hz, H-7^f), 4.89 (d, 1 H, H-1^b), 4.83 (d, 1 H, $J_{1,2}$ = 7.5 Hz, H-1^e), 4.79 (td, 1 H, $J_{3eq,4}$ = 4.2 Hz, $J_{3aq,4}$ = $J_{4,5}$ = 11.3 Hz, H-4^f), 4.68 (dd, 1 H, $J_{3,4}$ = 3.3 Hz, $J_{2,3}$ = 10.8 Hz, H-3^d), 4.60 (dd, 1 H, $J_{3,4}$ = 3.0 Hz, $J_{2,3}$ = 10.0 Hz, H-3^e), 4.49 (d, 1 H, $J_{1,2}$ = 8.0 Hz, H-1^a), 4.44–4.38 (m, 3 H, H-5^d, H-6a^e, H-2^{Cer}), 4.33–4.29 (m, 2 H, H-4^b, H-3^c), 4.23–4.11 (m, 5 H, H-6a^b, H-6a^c, H-6b^e, OH-3^a, C(=O)OCH_2^{f}), 4.03–3.84 (m, 11 H, H-3^a, H-4^a, H-5^b, H-6b^b, H-5^c, H-6b^c, H-5^e, H-5^f, H-9a^f, H-1a^{Cer}, C(=O)OCH_2), 3.66 (dd, 1 H, $J_{5,6a}$ = 7.5 Hz, J_{gem} = 11.3 Hz, H-6a^d), 3.56 (dd, 1 H, $J_{1b,2}$ = 3.0 Hz, J_{gem} = 9.0 Hz, H-1b^{Cer}), 3.49 (dd, 1 H, $J_{5,6}$ = 11.0 Hz, H-6^f), 3.36 (dd, 1 H, $J_{5,6b}$ = 6.0 Hz, H-6b^d), 3.29 (br t, 1 H, H-6a^a), 3.17 (br d, 1 H, H-5^a), 3.08 (m, 1 H, H-2^d), 3.02 (br d, 1 H, H-6b^a), 2.87 (m, 1 H, OH-6^a), 2.75 (near dt, 1 H, H-9b^f), 2.49 (dd, 1 H, J_{gem} = 12.5 Hz, H-3*eq*^{f}), 2.10 (s, 3 H, Ac), 2.09 (s, 3 H, Ac), 2.05 (s, 3 H, Ac), 2.03 (s, 3 H, Ac), 2.00–1.95 (m, 10 H, H-6a^{Cer}, H-6b^{Cer}, NHC(=O)CH_2, 2 Ac), 1.84 (s, 3 H, Ac), 1.83 (s, 3 H, Ac), 1.81 (s, 3 H, Ac), 1.69–1.64 (m, 4 H, H-3*ax*^{f}, Ac), 1.60–1.58 (m, 2 H, C(=O)OCH$_2$CH_2), 1.50–1.17 (m, 78 H, 4 CH$_2$^{f}, 26 CH$_2$^{Cer}, 2 ^{t}Bu), 0.89–0.86 (m, 9 H, CH$_2$*Me*^{f}, 2 Me^{Cer}), 0.77 (s, 3 H, Ac); ^{13}C NMR (125 MHz, $CDCl_3$) δ 172.7, 172.4, 170.6, 170.5, 170.5, 170.3, 170.2, 170.0, 169.9, 169.9, 169.1, 167.3, 166.0, 165.9, 165.6, 165.6, 165.5, 165.1, 164.8, 158.3, 158.0, 157.7, 157.0, 156.9, 138.5, 137.9, 133.7, 133.4, 133.1, 133.1, 130.3, 130.1, 129.9, 129.8, 129.7, 129.7, 129.4, 129.0, 128.9, 128.8, 128.7, 128.6, 128.5, 128.5, 128.4, 128.3, 128.2, 127.3, 127.2, 125.5, 125.4, 125.3, 125.0, 119.3, 117.0, 114.7, 112.5, 102.2, 101.0, 99.6, 99.0, 98.2, 97.0, 81.0, 79.0, 77.7, 74.2, 74.0, 74.0, 73.9, 73.5, 73.4, 72.7, 72.6, 71.8, 71.3, 71.2, 71.2, 70.4, 70.1, 69.5, 69.3, 69.2, 68.8, 67.6, 67.4, 67.3, 66.9, 66.8, 66.1, 62.5, 61.3, 61.3, 61.1, 59.6, 55.3, 50.4, 48.8, 38.6, 37.3, 36.8, 35.2, 35.1, 32.3, 31.9, 31.6, 31.1, 31.1, 29.7, 29.7, 29.7, 29.6, 29.6, 29.5, 29.4, 29.4, 29.3, 29.3, 28.9, 28.8, 28.1, 25.7, 25.6, 23.0, 22.7, 22.5, 22.0, 21.4, 21.1, 20.8, 20.7, 20.6, 20.2, 14.1, 14.1; HRMS (ESI) *m/z*: found $[M/2+Na]^+$ 1582.7097, $C_{163}H_{217}F_3N_4O_{52}$ calcd for $[M/2+Na]^+$ 1582.7098.

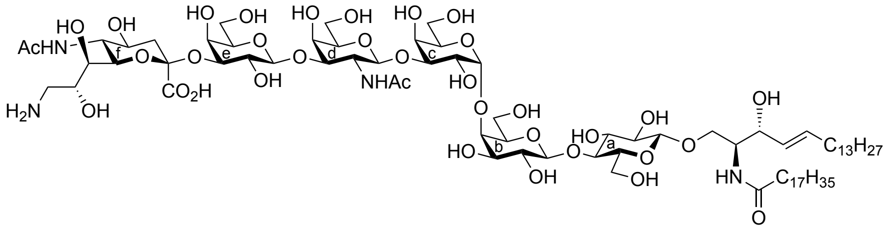

Compound 38

To a solution of compound **37** (138 mg, 44.2 μmol) in MeOH/THF = 1/1 (15 mL) was added 1 M NaOH aq. (884 μL, 0.884 mmol). After stirring for 48 h at ambient temperature as the progress of the reaction was monitored by TLC ($CHCl_3$/MeOH/5% aq. $CaCl_2$ = 5/4/1), 1 M NaOH aq. (442 μL, 0.442 mmol) was added to complete the reaction. After stirring for another 45 h, the reaction mixture was neutralized with Muromac (H^+), the resin was filtered through cotton, and washed with $CHCl_3$/MeOH = 1/1. The combined filtrate and washings were concentrated and co-evaporated with EtOH. The residue was purified by silica gel column chromatography ($CHCl_3$/MeOH/H_2O/28% NH_3 aq. = 5/2/0.2/0 → 5/5/0.5/0 → 3/3/1/0.1) to give compound **38** (69.3 mg, 92%) as a white amorphous solid; $[\alpha]_D$ +14.2° (c 0.7, $CHCl_3$/MeOH = 1/1); ^{1}H NMR (500 MHz, $CDCl_3$/CD_3OD = 1/1) δ 5.71 (near quin, 1 H, $J_{5,6a} = J_{5,6b}$ = 7.0 Hz, $J_{4,5}$ = 15.2 Hz, H-5^{Cer}), 5.46 (dd, 1 H, $J_{3,4}$ = 7.8 Hz, H-4^{Cer}), 4.99 (d, 1 H, $J_{1,2}$ = 3.5 Hz, H-1^c), 4.94–2.88 (m, 46 H, H-1^a, H-2^a, H-3^a, H-4^a, H-5^a, H-6a^a, H-6b^a, H-1^b, H-2^b, H-3^b, H-4^b, H-5^b, H-6a^b, H-6b^b, H-2^c, H-3^c, H-4^c, H-5^c, H-6a^c, H-6b^c, H-1^d, H-2^d, H-3^d, H-4^d, H-5^d, H-6a^d, H-6b^d, H-1^e, H-2^e, H-3^e, H-4^e, H-5^e, H-6a^e, H-6b^e, H-3*eq*^{f}, H-4^f, H-5^f, H-6^f, H-7^f, H-8^f, H-9a^f, H-9b^f, H-1a^{Cer}, H-1b^{Cer}, H-2^{Cer}, H-3^{Cer}), 2.19–2.16 (m, 2 H, NHC(=O)C*H*$_2$), 2.04–2.00 (m, 5 H, H-6a^{Cer}, H-6b^{Cer}, 2 Ac), 1.73 (t, 1 H, J_{gem} = 11.3 Hz, H-3*ax*^{f}), 1.59 (m, 2 H, NHC(=O)CH_2C*H*$_2$), 1.38–1.28 (m, 50 H, 25 CH_2^{Cer}), 0.90–0.88 (m, 6 H, 2 Me^{Cer}); ^{13}C NMR (200 MHz, $CDCl_3$/CD_3OD = 1/1) δ 175.3, 175.1, 174.6, 135.0, 130.0, 104.1, 103.6, 102.7, 77.1, 76.3, 75.7, 75.6, 75.1, 75.0, 74.0, 73.8, 72.5, 71.8, 71.4, 69.4, 69.2, 69.1, 68.8, 68.7, 68.5, 68.1, 67.9, 62.2, 61.0, 60.8, 54.2, 53.8, 53.1, 43.7, 41.5, 37.0, 32.9, 32.5, 30.2, 30.2, 30.2, 30.2, 30.1, 30.1, 29.9, 29.9, 29.9, 29.8, 26.6, 23.6, 23.2, 22.5, 14.3; HRMS (ESI) *m/z*: found [M–H]$^-$ 1705.9382, $C_{79}H_{142}N_4O_{35}$ calcd for [M–H]$^-$ 1705.9382.

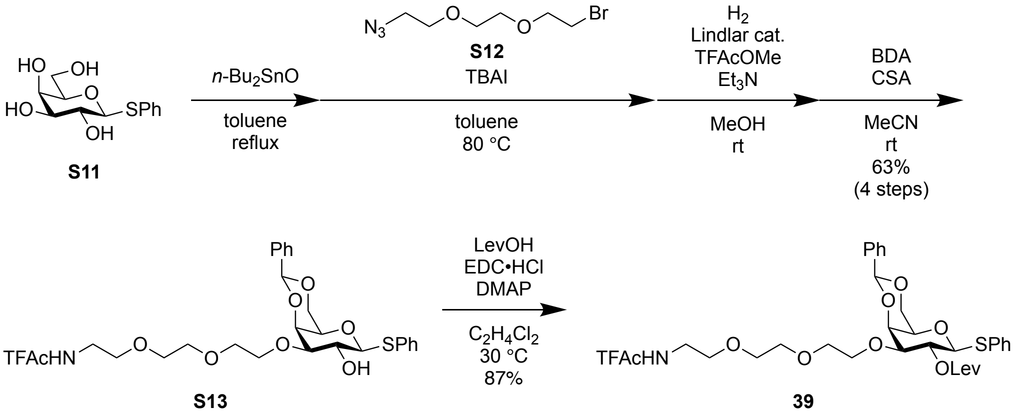

Supporting Scheme 4. Synthesis of Gal donor **39**.

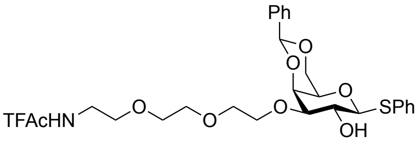

Compound S13

To a solution of compound **S11**[S06] (300 mg, 1.10 mmol) in MeOH (11 mL) were added *n*-Bu_2SnO (411 mg, 1.65 mmol) and Drierite (599 mg). After stirring for 7.5 h under reflux, the reaction mixture was cooled to ambient temperature and filtered through a pad of Celite. The pad was washed with MeOH. The combined filtrate and washings were concentrated, and dried for 12 h. The residue was dissolved in 1,4-dioxane (11 mL), followed by the addition of compound **S12**[S07] (786 mg, 3.30 mmol) and TBAI (1.22 g, 3.30 mmol). After stirring for 89 h at 80 °C as the progress of the reaction was monitored by TLC ($CHCl_3$/MeOH = 10/1), the reaction was quenched with MeOH and the mixture was concentrated. The residue was purified by silica gel column chromatography ($CHCl_3$/MeOH = 30/1). The resulting mixture was dried for 2 h, dissolved in MeOH (37 mL), followed by the addition of Et_3N (1.52 mL, 11.0 mL), TFAcOMe (546 µL, 5.50 mL) and $Pd(OH)_2$-C (77.2 mg, 20% on carbon). After stirring for 4 h at ambient temperature under H_2 gas as the progress of the reaction was monitored by TLC ($CHCl_3$/MeOH =10/1), the reaction mixture was filtered through a pad of Celite, the pad was washed with MeOH, the combined filtrate and washings were concentrated, and dried for 2 h. The residue was dissolved in MeCN (11 mL), followed by the addition of BDA (332 µL, 2.20 mmol) and CSA (25.6 mg, 0.110 mmol). After stirring for 50 min at ambient temperature as the progress of the reaction was monitored by TLC ($CHCl_3$/MeOH = 20/1), the reaction was quenched with Et_3N at 0 °C and the mixture was concentrated. The residue was purified by silica gel column chromatography ($CHCl_3$/MeOH = 100/1) to give compound **S13** (404 mg, 63%) as a white amorphous solid; $[\alpha]_D$ –39.7° (c 1.0, $CHCl_3$); ^1H NMR (500 MHz, $CDCl_3$) δ 8.10 (br s, 1 H, $CF_3C(=O)NH$), 7.67–7.23 (m, 10 H, 2 Ph),

5.50 (s, 1 H, PhC*H*<), 4.56 (d, 1 H, $J_{1,2}$ = 9.4 Hz, H-1), 4.47 (br s, 1 H, OH-2), 4.38 (dd, 1 H, $J_{5,6a}$ = 1.5 Hz, J_{gem} = 12.5 Hz, H-6a), 4.27 (d, 1 H, $J_{3,4}$ = 3.0 Hz, H-4), 4.04–4.00 (m, 2 H, H-6b, OCH_2), 3.96 (br t, 1 H, $J_{2,3}$ = 9.3 Hz, H-2), 3.74–3.66 (m, 2 H, 2 OCH_2), 3.65–3.45 (m, H-3, H-5, 7 OCH_2), 3.40–3.29 (m, 2 H, 2 $NHCH_2$); ^{13}C NMR (125 MHz, $CDCl_3$) δ 157.5, 157.2, 137.8, 133.5, 131.5, 129.2, 129.0, 128.8, 128.2, 128.2, 127.8, 126.6, 125.3, 117.2, 114.9, 101.3, 86.4, 83.7, 74.5, 70.7, 70.4, 70.1, 70.1, 70.0, 69.5, 68.9, 67.2, 39.8; HRMS (ESI) *m/z*: found $[M+Na]^+$ 610.1690, $C_{27}H_{32}F_3NO_8S$ calcd for $[M+Na]^+$ 610.1693.

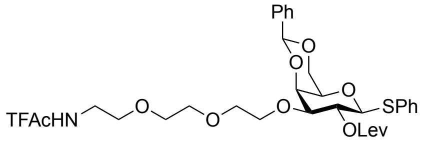

Compound 39

To a solution of compound **S13** (391 mg, 0.665 mmol) in $C_2H_4Cl_2$ (13 mL) were added DMAP (81.2 mg, 0.665 mmol), LevOH (102 µL, 0.998 mmol) and EDC·HCl (191 mg, 0.998 mmol). After stirring for 12.5 h at 30 °C as the progress of the reaction was monitored by TLC (toluene/acetone = 3/2, developed twice), the reaction mixture was diluted with EtOAc. The organic layer was washed with H_2O and brine, dried over Na_2SO_4, and concentrated. The residue was purified by silica gel column chromatography (toluene/acetone = 4/1) to give compound **39** (398 mg, 87%) as a white amorphous solid; $[\alpha]_D$ −3.8° (c 1.0, $CHCl_3$); ^{1}H NMR (500 MHz, $CDCl_3$) δ 7.61–7.14 (m, 11 H, 2 Ph, $CF_3C(=O)NH$), 5.49 (s, 1 H, PhC*H*<), 5.25 (t, 1 H, $J_{1,2}$ = $J_{2,3}$ = 9.9 Hz, H-2), 4.66 (d, 1 H, H-1), 4.38 (dd, 1 H, $J_{5,6a}$ = 1.3 Hz, J_{gem} = 12.2 Hz, H-6a), 4.32 (d, 1 H, $J_{3,4}$ = 3.0 Hz, H-4), 4.04 (dd, 1 H, $J_{5,6b}$ = 1.5 Hz, H-6b), 3.76 (m, 1 H, OCH_2), 3.65–3.56 (m, 6 H, H-3, 5 OCH_2), 3.53–3.43 (m, 5 H, H-5, 4 OCH_2), 3.38–3.25 (m, 2 H, 2 $NHCH_2$), 2.87–2.59 (m, 4 H, 4 $C(=O)CH_2$), 2.20 (s, 3 H, C(=O)Me); ^{13}C NMR (125 MHz, $CDCl_3$) δ 206.5, 171.2, 157.3, 157.0, 137.6, 133.5, 131.6, 129.2, 128.7, 128.1, 128.0, 126.6, 117.1, 114.8, 101.3, 85.2, 79.8, 73.3, 70.6, 70.4, 70.3, 70.0, 69.7, 69.3, 68.6, 68.4, 39.6, 37.9, 29.9, 28.1; HRMS (ESI) *m/z*: found $[M+Na]^+$ 708.2064, $C_{32}H_{38}F_3NO_{10}S$ calcd for $[M+Na]^+$ 708.2061.

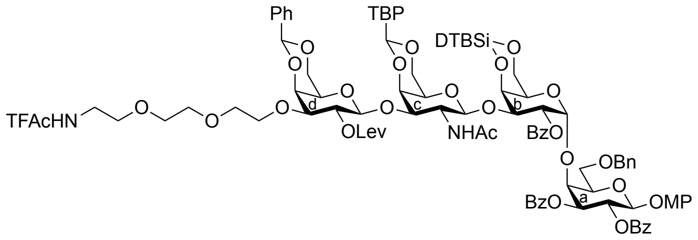

Compound 40

To a solution of donor **39** (79.2 g, 0.116 mmol) and acceptor **10** (51.5 g, 38.5 mmol) in CH_2Cl_2 (1.6 mL)

were added NIS (34.5 g, 0.154 mmol) and 4 Å molecular sieves (AW-300, 120 mg). After stirring for 1 h at ambient temperature, TfOH (1.0 µL, 11.6 mmol) was added at 0 °C. After stirring for 4 h at 0 °C as the progress of the reaction was monitored by TLC ($CHCl_3$/acetone = 4/1, developed twice), the reaction mixture was neutralized with satd. aq. $NaHCO_3$ and filtered through a pad of Celite. The pad was washed with $CHCl_3$. The combined filtrate and washings were diluted with $CHCl_3$ and washed with satd. aq. $Na_2S_2O_3$ and brine. The organic layer was dried over Na_2SO_4 and concentrated. The residue was purified by silica gel column chromatography (toluene/acetone = 5/1 → 4/1 → 2/1) to give compound **40** (53.2 g, 72%) as a white amorphous solid; $[\alpha]_D$ +89.7° (c 1.0, $CHCl_3$); ^1H NMR (500 MHz, $CDCl_3$) δ 8.20–6.72 (m, 34 H, 7 Ar, $CF_3C(=O)NH$), 6.55 (d, 1 H, $J_{2,NH}$ = 6.5 Hz, NH-2^c), 5.96 (dd, 1 H, $J_{1,2}$ = 7.8 Hz, $J_{2,3}$ = 10.7 Hz, H-2^a), 5.69 (dd, 1 H, $J_{1,2}$ = 3.8 Hz, $J_{2,3}$ = 10.7 Hz, H-2^b), 5.56 (s, 1 H, ArC*H*<), 5.48 (s, 1 H, PhC*H*<), 5.45 (d, 1 H, $J_{1,2}$ = 8.0 Hz, H-1^c), 5.33 (dd, 1 H, $J_{3,4}$ = 2.8 Hz, H-3^a), 5.24–5.20 (m, 2 H, H-1^b, H-2^d), 5.09 (d, 1 H, H-1^a), 4.82–4.79 (m, 2 H, H-4^b, H-3^c), 4.56 (d, 1 H, $J_{1,2}$ = 8.0 Hz, H-1^d), 4.40 (m, 2 H, H-4^a, H-4^c), 4.36 (br d, 1 H, J_{gem} = 11.5 Hz, H-6a^d), 4.31 (dd, 1 H, $J_{3,4}$ = 2.5 Hz, H-3^b), 4.27 (br d, 1 H, J_{gem} = 12.0 Hz, H-6a^c), 4.23 (d, 1 H, $J_{3,4}$ = 3.5 Hz, H-4^d), 4.11–4.04 (m, 5 H, H-5^b, H-6b^c, H-6b^d, 2 PhCH_2), 3.90 (t, 1 H, $J_{5,6a}$ = $J_{5,6b}$ = 6.5 Hz, H-5^a), 3.77–3.69 (m, 4 H, OMe, OCH_2), 3.64–3.31 (m, 18 H, H-6a^a, H-6b^a, H-6a^b, H-6b^b, H-2^c, H-5^c, H-3^d, H-5^d, 9 OCH_2, NHCH_2), 3.22 (m, 1 H, NHCH_2), 2.85 (m, 1 H, $C(=O)CH_2$), 2.54–2.45 (m, 2 H, 2 $C(=O)CH_2$), 2.38 (m, 1 H, $C(=O)CH_2$), 2.11 (s, 3 H, C(=O)Me), 1.32-1.31 (m, 12 H, Ac, ^{t}Bu), 1.03 (s, 9 H, ^{t}Bu), 0.95 (s, 9 H, ^{t}Bu); ^{13}C NMR (125 MHz, $CDCl_3$) δ 207.3, 171.2, 171.0, 166.2, 165.9, 165.5, 157.3, 157.0, 155.6, 151.3, 151.2, 137.7, 137.6, 135.6, 133.6, 133.2, 133.2, 130.5, 129.9, 129.8, 129.7, 129.4, 129.1, 128.9, 128.7, 128.5, 128.4, 128.4, 128.2, 127.8, 127.7, 126.5, 126.3, 124.7, 119.0, 117.1, 114.8, 114.4, 101.7, 101.3, 101.2, 100.7, 100.0, 98.1, 78.5, 76.3, 75.2, 74.3, 74.2, 74.0, 73.8, 73.6, 73.3, 73.2, 70.8, 70.6, 70.5, 70.4, 69.7, 69.6, 69.4, 69.3, 68.4, 68.2, 68.0, 66.7, 66.4, 66.2, 55.6, 54.9, 39.6, 37.6, 34.6, 31.4, 29.9, 29.7, 27.8, 27.5, 27.4, 27.2, 23.3, 23.0, 22.7, 20.6; HRMS (ESI) *m/z*: found $[M+Na]^+$ 1935.7461, $C_{100}H_{119}F_3N_2O_{30}Si$ calcd for $[M+Na]^+$ 1935.7461.

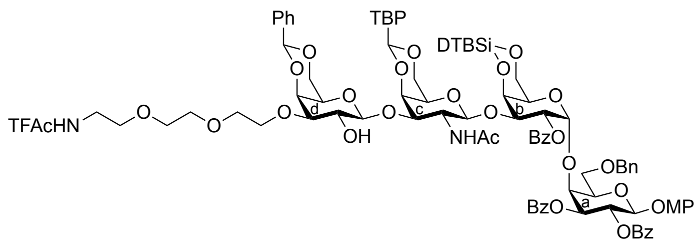

Compound 41

To a solution of compound **40** (66.3 mg, 34.6 µmol) in MeOH/THF = 1/5 (1.7 mL) was added N_2H_4·AcOH (31.9 mg, 0.346 mmol). After stirring for 40 min at ambient temperature as the progress of the reaction was monitored by TLC (toluene/acetone = 3/2), the reaction mixture was diluted with EtOAc. The organic layer was washed with satd. aq. $NaHCO_3$ and brine, dried over Na_2SO_4, and concentrated. The residue was purified by silica gel column chromatography (toluene/acetone = 2/1) to give compound **41** (58.9 mg, 94%) as a white amorphous solid; $[\alpha]_D$ +74.8° (c 1.0, $CHCl_3$); ^{1}H NMR (500 MHz, $CDCl_3$) δ 8.19–6.72 (m, 34 H, 7 Ar, $CF_3C(=O)NH$), 5.97 (dd, 1 H, $J_{1,2}$ = 7.7 Hz, $J_{2,3}$ = 10.9 Hz, H-2^a), 5.72–5.69 (m, 2 H, H-2^b, NH-2^c), 5.55 (s, 1 H, ArC*H*<), 5.48 (s, 1 H, PhC*H*<), 5.36 (d, 1 H, $J_{1,2}$ = 8.0 Hz, H-1^c), 5.33 (dd, 1 H, $J_{3,4}$ = 3.0 Hz, H-3^a), 5.20 (d, 1 H, $J_{1,2}$ = 3.5 Hz, H-1^b), 5.10 (d, 1 H, H-1^a), 4.81–4.79 (m, 2 H, H-4^b, H-3^c), 4.42–4.41 (m, 2 H, H-4^a, H-4^c), 4.36–4.34 (m, 2 H, H-1^d, H-6^d), 4.31–4.25 (m, 2 H, H-3^b, H-6a^c), 4.18 (d, 1 H, $J_{3,4}$ = 3.3 Hz, H-4^d), 4.12–4.03 (m, 5 H, H-5^b, H-6b^c, H-6b^d, 2 PhCH_2), 3.92–3.82 (m, 3 H, H-5^a, H-2^d, OCH_2), 3.74–3.41 (m, 18 H, H-6a^a, H-6b^a, H-6a^b, H-6b^b, H-2^c, H-5^d, OMe, 9 OCH_2), 3.36 (br s, 1 H, H-5^c), 3.32 (dd, 1 H, $J_{2,3}$ = 9.5 Hz, H-3^d), 3.26 (m, 1 H, NHCH_2), 3.10 (m, 1 H, NHCH_2), 2.43 (m, 1 H, OH-2^d), 1.30 (s, 9 H, ^{t}Bu), 1.19 (s, 3 H, Ac), 1.04 (s, 9 H, ^{t}Bu), 0.98 (s, 9 H, ^{t}Bu); ^{13}C NMR (125 MHz, $CDCl_3$) δ 171.4, 166.0, 165.7, 165.4, 157.5, 157.2, 156.9, 156.6, 155.5, 151.5, 151.1, 137.8, 137.7, 137.4, 135.3, 133.5, 133.2, 133.2, 130.3, 129.7, 129.5, 129.4, 129.2, 128.9, 128.8, 128.6, 128.5, 128.3, 128.2, 128.1, 128.1, 127.7, 126.4, 126.3, 125.2, 124.7, 124.4, 118.9, 117.0, 114.7, 114.3, 104.0, 101.0, 100.3, 98.0, 80.5, 77.3, 77.2, 77.0, 76.7, 76.1, 75.8, 74.2, 74.0, 73.9, 73.8, 73.7, 73.5, 73.1, 70.5, 70.2, 70.1, 69.9, 69.6, 69.5, 69.4, 69.2, 69.2, 68.1, 68.0, 67.8, 66.5, 66.3, 55.5, 54.9, 39.4, 34.4, 31.6, 31.2, 27.4, 27.3, 23.3, 22.7, 21.3, 20.5; HRMS (ESI) *m/z*: found $[M+Na]^+$ 1837.7094, $C_{95}H_{113}F_3N_2O_{28}Si$ calcd for $[M+Na]^+$ 1837.7093.

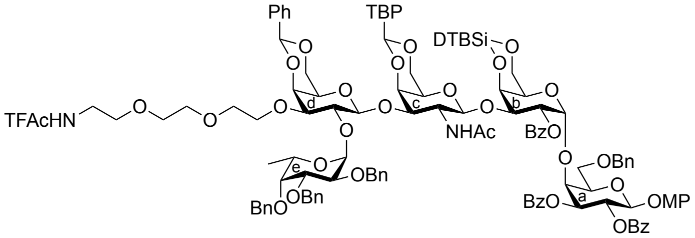

Compound 43

To a solution of donor **42**[S08] (186 mg, 0.354 mmol) and acceptor **41** (428 mg, 0.236 mmol) in CH_2Cl_2/CPME = 1/1 (5.9 mL) were added NIS (95.0 mg, 0.424 mmol) and 4 Å molecular sieves (600 mg). After stirring for 1 h at ambient temperature, TfOH (2.1 µL, 23.6 µmol) was added at 0 °C. After stirring for 29 h at 0 °C as the progress of the reaction was monitored by TLC (toluene/acetone = 3/1), the reaction mixture was neutralized with satd. aq. $NaHCO_3$ and filtered through a pad of Celite. The

pad was washed with $CHCl_3$. The combined filtrate and washings were diluted with $CHCl_3$ and washed with satd. aq. $Na_2S_2O_3$ and brine. The organic layer was dried over Na_2SO_4 and concentrated. The residue was purified by gel filtration (LH20; $CHCl_3$/MeOH = 1/1), then silica gel column chromatography (toluene/acetone = 7/1 → 4/1) to give compound **43** (430 mg, 82%) as a white amorphous solid; $[\alpha]_D$ +64.0° (c 1.0, $CHCl_3$); ^{1}H NMR (500 MHz, $CDCl_3$) δ 8.22–6.72 (m, 49 H, 10 Ar, CF_3C(=O)NH), 5.95 (dd, 1 H, $J_{1,2}$ = 7.7 Hz, $J_{2,3}$ = 10.7 Hz, H-2^a), 5.86 (dd, 1 H, $J_{1,2}$ = 3.7 Hz, $J_{2,3}$ = 10.8 Hz, H-2^b), 5.74 (d, 1 H, $J_{1,2}$ = 3.5 Hz, H-1^e), 5.54 (s, 1 H, ArC*H*<), 5.46 (s, 1 H, PhC*H*<), 5.37 (d, 1 H, $J_{1,2}$ = 8.0 Hz, H-1^c), 5.32 (dd, 1 H, $J_{3,4}$ = 2.8 Hz, H-3^a), 5.25 (d, 1 H, H-1^b), 5.09 (d, 1 H, H-1^a), 4.91 (d, 1 H, $J_{2,NH}$ = 7.0 Hz, NH-2^c), 4.89–4.86 (m, 2 H, H-4^b, PhCH_2), 4.83 (dd, 1 H, $J_{2,3}$ = 3.3 Hz, $J_{3,4}$ = 11.3 Hz, H-3^c), 4.78 (d, 1 H, J_{gem} = 13.0 Hz, PhCH_2), 4.68 (d, 1 H, J_{gem} = 12.0 Hz, PhCH_2), 4.60–4.55 (m, 2 H, 2 PhCH_2), 4.44 (d, 1 H, H-4^a), 4.33–4.31 (m, 4 H, H-3^b, H-4^c, 2 PhCH_2), 4.27 (br d, 1 H, J_{gem} = 12.0 Hz, H-6a^d), 4.21 (d, 1 H, $J_{1,2}$ = 8.0 Hz, H-1^d), 4.15–4.11 (m, 4 H, H-5^b, H-6a^c, H-4^d, H-5^e), 4.08–4.06 (m, 2 H, H-6b^d, PhCH_2), 4.00 (br d, 1 H, H-6b^c), 3.97-3.89 (m, 3 H, H-5^a, H-2^d, H-2^e), 3.75 (s, 3 H, OMe), 3.66–3.18 (m, 20 H, H-6a^a, H-6b^a, H-6a^b, H-6b^b, H-2^c, H-5^c, H-3^d, H-5^d, H-3^e, H-4^e, 10 OCH_2), 3.09–3.05 (m, 2 H, 2 NHCH_2), 1.30 (s, 9 H, ^{t}Bu), 1.04 (s, 9 H, ^{t}Bu), 1.00 (s, 9 H, ^{t}Bu), 0.85 (s, 3 H, Ac), 0.55 (d, 3 H, $J_{5,6}$ = 6.5 Hz, H-6^e); ^{13}C NMR (125 MHz, $CDCl_3$) δ 171.2, 166.2, 165.9, 165.5, 157.7, 157.4, 155.6, 152.0, 151.3, 139.6, 138.7, 138.6, 137.8, 137.5, 136.1, 133.7, 133.3, 133.3, 130.5, 129.8, 129.7, 129.6, 129.4, 129.0, 128.9, 128.8, 128.7, 128.5, 128.4, 128.4, 128.2, 128.1, 127.9, 127.8, 127.8, 127.7, 127.5, 127.1, 126.9, 126.7, 126.1, 124.8, 119.1, 117.1, 114.8, 114.4, 101.2, 101.1, 101.1, 100.6, 100.5, 97.9, 95.1, 82.2, 77.9, 76.1, 75.8, 75.7, 75.5, 74.4, 74.1, 73.7, 73.6, 73.2, 72.2, 71.0, 70.8, 70.5, 70.1, 70.0, 69.8, 69.7, 69.4, 69.2, 68.1, 67.9, 66.7, 66.5, 66.2, 55.7, 55.6, 39.6, 34.6, 31.4, 29.7, 27.6, 27.4, 23.5, 22.6, 20.6, 15.4; HRMS (ESI) *m/z*: found $[M+Na]^+$ 2253.9080, $C_{122}H_{141}F_3N_2O_{32}Si$ calcd for $[M+Na]^+$ 2253.9081.

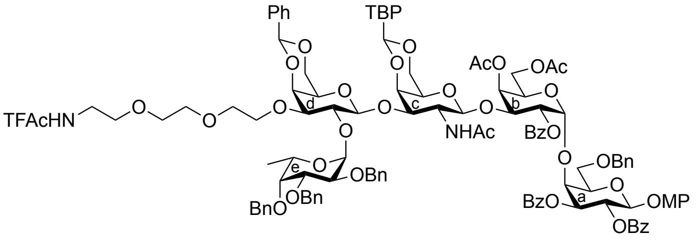

Compound 44

To a solution of compound **43** (426 mg, 0.191 mmol) in THF (3.8 mL) was added 1 M TBAHF in THF (1.91 mL, 1.91 mmol). After stirring for 13 h at ambient temperature as the progress of the reaction was monitored by TLC (*n*-hexane/acetone = 1/1), the reaction mixture was diluted with EtOAc and washed with 2 M HCl, H_2O, satd. aq. $NaHCO_3$ and brine. The organic layer was dried over Na_2SO_4,

concentrated and dried for 2 h. The residue was dissolved in pyridine (1.9 mL), followed by the addition of Ac_2O (72.4 µL, 0.764 mmol) and DMAP (2.3 mg, 19.1 µmol) at 0 °C. After stirring for 2 h at ambient temperature as the progress of the reaction was monitored by TLC (*n*-hexane/acetone = 1/1), Ac_2O (72.4 µL, 0.764 mmol) was added to complete the reaction. After stirring for another 2 h, the reaction was quenched with MeOH at 0 °C and diluted with EtOAc. The organic layer was washed with 2 M HCl, H_2O, satd. aq. $NaHCO_3$ and brine, dried over Na_2SO_4, and concentrated. The residue was purified by silica gel column chromatography (*n*-hexane/acetone = 2/1 → 3/2) to give compound **44** (386 mg, 93%) as a white amorphous solid; $[\alpha]_D$ +69.5° (c 1.0, $CHCl_3$); ^{1}H NMR (500 MHz, $CDCl_3$) δ 8.12–6.72 (m, 49 H, 10 Ar, $CF_3C(=O)NH$), 6.07 (dd, 1 H, $J_{1,2}$ = 7.5 Hz, $J_{2,3}$ = 10.7 Hz, H-2^{a}), 5.73 (d, 1 H, $J_{1,2}$ = 3.5 Hz, H-1^{e}), 5.65–5.61 (m, 2 H, H-2^{b}, NH-2^{c}), 5.53 (d, 1 H, $J_{3,4}$ = 3.0 Hz, H-4^{b}), 5.49 (s, 1 H, ArC*H*<), 5.47 (s, 1 H, PhC*H*<), 5.41 (d, 1 H, $J_{1,2}$ = 8.5 Hz, H-1^{c}), 5.38 (dd, 1 H, $J_{3,4}$ = 2.8 Hz, H-3^{a}), 5.25 (d, 1 H, $J_{1,2}$ = 3.5 Hz, H-1^{b}), 5.09 (d, 1 H, H-1^{a}), 4.81 (d, 1 H, J_{gem} = 10.5 Hz, PhCH_2), 4.96 (d, 1 H, J_{gem} = 11.5 Hz, PhCH_2), 4.64–4.56 (m, 3 H, H-3^{b}, 2 PhCH_2), 4.54 (t, 1 H, $J_{5,6a}$ = $J_{5,6b}$ = 6.8 Hz, H-5^{b}), 4.51–4.49 (m, 2 H, H-4^{a}, H-3^{c}), 4.41 (d, 1 H, $J_{1,2}$ = 8.0 Hz, H-1^{d}), 4.36–4.33 (m, 2 H, H-6a^{d}, PhCH_2), 4.30 (d, 1 H, $J_{3,4}$ = 3.0 Hz, H-4^{c}), 4.28–4.24 (m, 3 H, H-6a^{c}, 2 PhCH_2), 4.19 (d, 1 H, $J_{3,4}$ = 3.5 Hz, H-4^{d}), 4.11 (d, 1 H, J_{gem} = 11.5 Hz, PhCH_2), 4.06–3.90 (m, 6 H, H-5^{a}, H-6b^{c}, H-2^{d}, H-6b^{d}, H-2^{e}, H-5^{e}), 3.75 (s, 3 H, OMe), 3.67 (m, 1 H, OCH_2), 3.56–3.18 (m, 19 H, H-6a^{a}, H-6b^{a}, H-6a^{b}, H-6b^{b}, H-2^{c}, H-5^{c}, H-3^{d}, H-5^{d}, H-3^{e}, H-4^{e}, 9 OCH_2), 3.08–3.05 (m, 2 H, 2 NHCH_2), 2.09 (s, 3 H, Ac), 1.81 (s, 3 H, Ac), 1.42 (s, 3 H, Ac), 1.25 (s, 9 H, ^{t}Bu), 0.52 (d, 3 H, $J_{5,6}$ = 6.5 Hz, H-6^{e}); ^{13}C NMR (125 MHz, $CDCl_3$) δ 171.1, 170.1, 170.1, 165.9, 165.9, 165.5, 157.6, 157.4, 155.6, 151.8, 151.4, 139.3, 138.9, 138.8, 137.7, 137.4, 135.8, 133.7, 133.3, 130.3, 129.7, 129.7, 129.6, 129.4, 128.9, 128.8, 128.7, 128.5, 128.4, 128.4, 128.1, 128.1, 128.1, 128.0, 127.9, 127.8, 127.7, 127.5, 127.2, 126.5, 126.1, 124.8, 119.1, 117.0, 114.8, 114.4, 101.8, 101.3, 101.1, 100.7, 99.6, 96.7, 95.5, 82.1, 79.0, 78.8, 76.2, 75.6, 75.4, 73.8, 73.7, 73.5, 73.2, 72.8, 72.5, 72.2, 71.9, 70.2, 70.0, 69.9, 69.8, 69.6, 69.3, 68.8, 68.1, 68.0, 67.4, 67.3, 66.7, 66.4, 61.8, 55.6, 55.2, 39.6, 34.6, 31.3, 29.3, 23.1, 21.0, 20.6, 15.3; HRMS (ESI) *m/z*: found $[M+Na]^+$ 2197.8274, $C_{118}H_{129}F_3N_2O_{34}$ calcd for $[M+Na]^+$ 2197.8271.

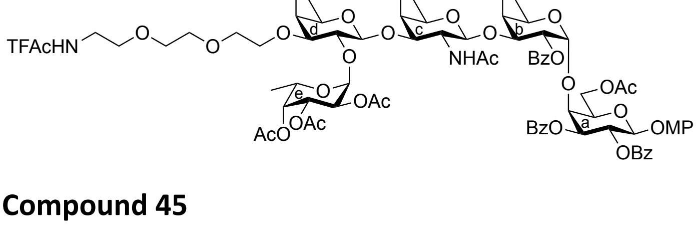

Compound 45

To a solution of compound **44** (57.5 mg, 26.4 µmol) in 1,4-dioxane/MeOH = 1/1 (1.3 mL) was added

Pd(OH)$_2$-C (115 mg, 20% on carbon). After stirring for 5 h at ambient temperature under H_2 gas as the progress of the reaction was monitored by TLC ($CHCl_3$/MeOH =5/1), the reaction mixture was filtered through a pad of Celite, the pad was washed with $CHCl_3$, the combined filtrate and washings were concentrated, co-evaporated with toluene, and dried for 2 h. The residue was dissolved in pyridine (0.26 mL), followed by the addition of Ac_2O (30.0 µL, 0.317 mmol) and DMAP (0.3 mg, 2.64 µmol) at 0 °C. After stirring for 7 h at ambient temperature as the progress of the reaction was monitored by TLC ($CHCl_3$/MeOH =20/1), the reaction was quenched with MeOH at 0 °C, and the mixture was co-evaporated with toluene, and diluted with $CHCl_3$. The organic layer was washed with 2 M HCl, H_2O, satd. aq. $NaHCO_3$ and brine, dried over Na_2SO_4, and concentrated. The residue was purified by silica gel column chromatography (*n*-hexane/acetone = 1/1) to give compound **45** (44.4 mg, 88%) as a white amorphous solid; $[\alpha]_D$ +46.8° (c 1.0, $CHCl_3$); ^{1}H NMR (500 MHz, $CDCl_3$) δ 8.12–6.75 (m, 20 H, 4 Ar, CF_3C(=O)NH), 6.50 (d, 1 H, $J_{2,NH}$ = 6.5 Hz, NH-2^c), 5.94 (dd, 1 H, $J_{1,2}$ = 7.8 Hz, $J_{2,3}$ = 10.5 Hz, H-2^a), 5.63 (d, 1 H, $J_{1,2}$ = 3.5 Hz, H-1^e), 5.59 (d, 1 H, $J_{3,4}$ = 2.0 Hz, H-4^b), 5.51 (dd, 1 H, $J_{1,2}$ = 3.5 Hz, $J_{2,3}$ = 10.5 Hz, H-2^b), 5.39 (d, 1 H, $J_{3,4}$ = 3.0 Hz, H-4^c), 5.36–5.32 (m, 2 H, H-3^a, H-4^d), 5.27 (d, 1 H, H-1^b), 5.22 (d, 1 H, $J_{1,2}$ = 8.0 Hz, H-1^c), 5.20–5.19 (m, 2 H, H-3^e, H-4^e), 5.13 (m, 1 H, H-2^e), 5.11 (d, 1 H, H-1^a), 5.01 (dd, 1 H, $J_{2,3}$ = 11.0 Hz, H-3^c), 4.60–4.56 (m, 2 H, H-3^b, H-5^c), 4.50–4.44 (m, 2 H, H-6a^b, H-5^e), 4.40 (d, 1 H, $J_{1,2}$ = 8.0 Hz, H-1^d), 4.34 (d, 1 H, $J_{3,4}$ = 2.8 Hz, H-4^a), 4.25 (dd, 1 H, $J_{5,6a}$ = 5.3 Hz, J_{gem} = 11.3 Hz, H-6a^a), 4.12–4.07 (m, 3 H, H-6b^a, H-6a^d, H-6b^d), 4.03–3.95 (m, 3 H, H-5^a, H-5^b, H-6b^b), 3.85 (dd, 1 H, $J_{5,6a}$ = 6.8 Hz, J_{gem} = 11.3 Hz, H-6a^c), 3.76–3.67 (m, 6 H, H-6b^c, H-5^d, OMe, OCH_2), 3.63–3.33 (m, 13 H, H-2^d, H-3^d, 9 OCH_2, 2 NHCH_2), 3.02 (m, 1 H, H-2^c), 2.14 (s, 6 H, 2 Ac), 2.12 (s, 3 H, Ac), 2.10 (s, 3 H, Ac), 2.09 (s, 3 H, Ac), 2.08 (s, 3 H, Ac), 1.94 (s, 3 H, Ac), 1.92 (s, 3 H, Ac), 1.90 (s, 3 H, Ac), 1.89 (s, 3 H, Ac), 1.38 (s, 3 H, Ac), 1.10 (d, 3 H, $J_{5,6}$ = 6.5 Hz, H-6^e); ^{13}C NMR (125 MHz, $CDCl_3$) δ 171.9, 171.3, 170.8, 170.6, 170.6, 170.5, 170.4, 170.1, 169.8, 169.6, 166.2, 166.0, 165.2, 157.5, 157.2, 155.7, 151.1, 133.7, 133.6, 133.4, 130.2, 129.9, 129.7, 129.2, 129.0, 128.7, 128.6, 128.6, 128.5, 118.7, 117.1, 114.8, 114.5, 101.8, 101.0, 99.0, 98.4, 95.1, 81.5, 74.8, 73.3, 73.2, 72.1, 71.6, 71.3, 71.1, 70.9, 70.8, 70.6, 70.4, 70.4, 70.2, 70.2, 70.1, 69.5, 69.4, 69.3, 68.7, 68.5, 67.9, 67.5, 66.1, 64.3, 62.5, 61.8, 61.6, 61.3, 56.1, 55.6, 53.8, 39.8, 31.7, 29.7, 29.3, 22.6, 20.8, 20.8, 20.7, 20.7, 20.7, 20.6, 20.6, 20.6, 20.5, 15.7; HRMS (ESI) *m/z*: found $[M+Na]^+$ 1941.5987, $C_{93}H_{109}F_3N_2O_{41}$ calcd for $[M+Na]^+$ 1941.5986.

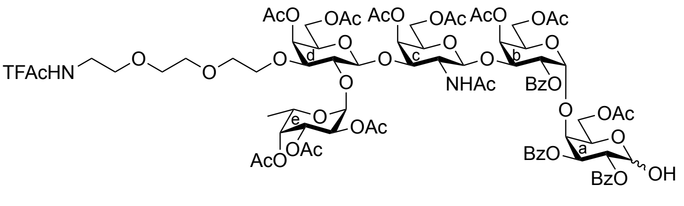

Compound 46

To a solution of compound **45** (44.4 mg, 23.1 µmol) in MeCN/toluene/H_2O = 6/5/3 (0.46 mL) was added cerium (IV) ammonium nitrate (127 g, 0.231 mmol) at 0 °C. After stirring for 2 h at 0 °C as the progress of the reaction was monitored by TLC ($CHCl_3$/MeOH = 20/1), the reaction mixture was diluted with EtOAc. The organic layer was washed with H_2O, satd. aq. $NaHCO_3$ and brine, dried over Na_2SO_4, and concentrated. The residue was purified by silica gel column chromatography ($CHCl_3$/MeOH = 60/1) to give compound **46** (27.4 mg, 65%, α:β = 1:0.3) as a white amorphous solid; α isomer; ^{1}H NMR (500 MHz, $CDCl_3$) δ 8.11–7.33 (m, 16 H, 3 Ph, CF_3C(=O)NH), 6.50 (d, 1 H, $J_{2,NH}$ = 6.0 Hz, NH-2^c), 5.80 (dd, 1 H, $J_{2,3}$ = 11.0 Hz, $J_{3,4}$ = 2.5 Hz, H-3^a), 5.70 (br t, 1 H, $J_{1,2}$ = $J_{1,OH}$ = 3.3 Hz, H-1^a), 5.63 (d, 1 H, $J_{1,2}$ = 3.4 Hz, H-1^e), 5.61 (dd, 1 H, H-2^a), 5.55 (d, 1 H, $J_{3,4}$ = 3.5 Hz, H-4^b), 5.49 (dd, 1 H, $J_{1,2}$ = 3.5 Hz, $J_{2,3}$ = 10.5 Hz, H-2^b), 5.39 (d, 1 H, $J_{3,4}$ = 3.5 Hz, H-4^c), 5.33 (d, 1 H, $J_{3,4}$ = 3.0 Hz, H-4^d), 5.30 (d, 1 H, H-1^b), 5.23 (d, 1 H, $J_{1,2}$ = 8.0 Hz, H-1^c), 5.22–5.18 (m, 2 H, H-3^e, H-4^e), 5.13 (dd, 1 H, $J_{2,3}$ = 10.8 Hz, H-2^e), 5.01 (dd, 1 H, $J_{2,3}$ = 11.0 Hz, H-3^c), 4.60–4.51 (m, 2 H, H-3^b, H-5^c), 4.49 (m, 1 H, H-5^e), 4.41–4.37 (m, 2 H, H-6a^b, H-1^d), 4.31 (d, 1 H, H-4^a), 4.24 (dd, 1 H, $J_{5,6a}$ = 5.3 Hz, J_{gem} = 11.3 Hz, H-6a^a), 4.12–3.99 (m, 6 H, H-5^a, H-6b^a, H-5^b, H-6b^b, H-6a^d, H-6b^d), 3.83 (m, 1 H, H-6a^c), 3.78–3.68 (m, 3 H, H-6b^c, H-5^d, OCH_2), 3.64–3.34 (m, 13 H, H-2^d, H-3^d, 9 OCH_2, 2 NHCH_2), 3.01 (m, 1 H, H-2^c), 2.90 (d, 1 H, OH-1^a), 2.15–2.08 (m, 18 H, 6 Ac), 1.94 (s, 3 H, Ac), 1.91 (s, 3 H, Ac), 1.89 (s, 3 H, Ac), 1.70 (s, 3 H, Ac), 1.38 (s, 3 H, Ac), 1.09 (d, 3 H, $J_{5,6}$ = 6.5 Hz, H-6^e); HRMS (ESI) *m/z*: found $[M+Na]^+$ 1835.5567, $C_{81}H_{99}F_3N_2O_{41}$ calcd for $[M+Na]^+$ 1835.3368.

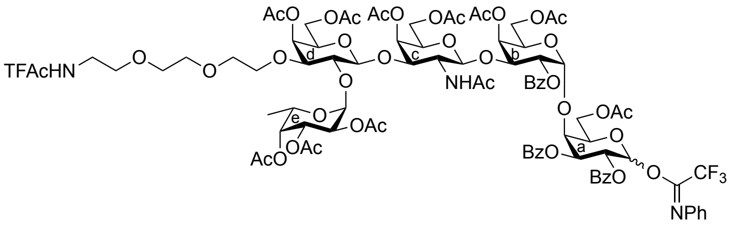

Compound 47

To a solution of compound **46** (12.7 mg, 7.00 µmol) in acetone (0.14 mL) were added CF_3C(=NPh)Cl (2.3 µL, 14.0 µmol) and K_2CO_3 (4.8 mg, 35.0 µmol). After stirring for 8 h at ambient temperature as the progress of the reaction was monitored by TLC ($CHCl_3$/MeOH = 20/1), the reaction mixture was concentrated. The residue was purified by silica gel column chromatography ($CHCl_3$/MeOH = 80/1) to give compound **47** (13.2 mg, 95%, α:β = 1:0.3) as a white amorphous solid; α isomer; ^{1}H NMR (500 MHz, $CDCl_3$) δ 8.11–6.47 (m, 22 H, 4 Ph, CF_3C(=O)NH, NH-2^c), 6.06 (br s, 1 H, H-1^a), 5.89 (m, 1 H, H-2^a), 5.78 (br dd, 1 H, $J_{3,4}$ = 2.3 Hz, $J_{2,3}$ = 10.8 Hz, H-3^a), 5.64 (d, 1 H, $J_{1,2}$ = 3.3 Hz, H-1^e), 5.56 (d, 1 H, $J_{3,4}$

= 2.5 Hz, H-4[b]), 5.49 (dd, 1 H, $J_{1,2}$ = 3.5 Hz, $J_{2,3}$ = 10.5 Hz, H-2[b]), 5.40 (d, 1 H, $J_{3,4}$ = 3.2 Hz, H-4[c]), 5.33–5.32 (m, 2 H, H-1[b], H-4[d]), 5.25 (d, 1 H, $J_{1,2}$ = 8.0 Hz, H-1[c]), 5.22–5.19 (m, 2 H, H-3[e], H-4[e]), 5.13 (dd, 1 H, $J_{2,3}$ = 10.5 Hz, H-2[e]), 5.03 (dd, 1 H, $J_{2,3}$ = 11.3 Hz, H-3[c]), 4.55–4.53 (m, 2 H, H-3[b], H-5[c]), 4.48 (m, 1 H, H-5[e]), 4.42–4.39 (m, 2 H, H-4[a], H-1[d]), 4.34 (m, 1 H, H-6[b]), 4.25 (dd, 1 H, $J_{5,6a}$ = 5.5 Hz, J_{gem} = 11.5 Hz, H-6a[a]), 4.13–4.00 (m, 6 H, H-5[a], H-6b[a], H-5[b], H-6b[b], H-6a[d], H-6b[d]), 3.84 (dd, 1 H, $J_{5,6a}$ = 5.5 Hz, J_{gem} = 11.5 Hz, H-6a[c]), 3.79–3.69 (m, 3 H, H-6b[c], H-5[d], OCH_2), 3.63–3.33 (m, 13 H, H-2[d], H-3[d], 9 OCH_2, 2 $NHCH_2$), 3.01 (m, 1 H, H-2[c]), 2.15–2.07 (m, 18 H, 6 Ac), 1.94 (s, 3 H, Ac), 1.93 (s, 3 H, Ac), 1.91 (s, 3 H, Ac), 1.75 (s, 3 H, Ac), 1.39 (s, 3 H, Ac), 1.10 (d, 3 H, $J_{5,6}$ = 6.5 Hz, H-6[e]); HRMS (ESI) *m/z*: found $[M+Na]^+$ 2006.5864, $C_{89}H_{103}F_6N_3O_{41}$ calcd for $[M+Na]^+$ 2006.5863.

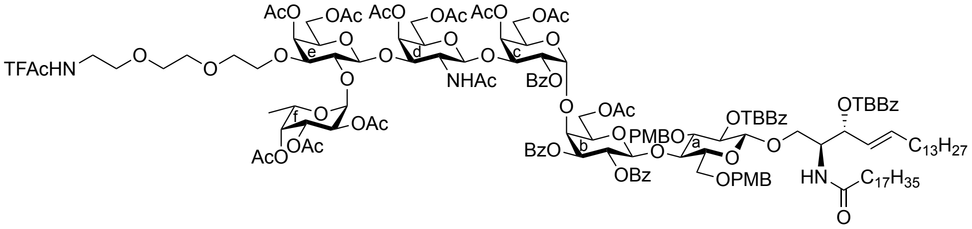

Compound 48

To a solution of donor **47** (50.0 mg, 25.2 µmol) and acceptor **17**[S03] (48.7 mg, 37.8 µmol) in CH_2Cl_2 (1.3 mL) was added 4 Å molecular sieves (AW-300, 130 mg). After stirring for 1 h at ambient temperature, TMSOTf (0.91 µL, 5.04 µmol) was added at 0 °C. After stirring for 16.5 h at 0 °C as the progress of the reaction was monitored by TLC (toluene/acetone = 3/1), the reaction mixture was neutralized with satd. aq. $NaHCO_3$ and filtered through a pad of Celite. The pad was washed with $CHCl_3$. The combined filtrate and washings were diluted with $CHCl_3$ and washed with brine. The organic layer was dried over Na_2SO_4 and concentrated. The residue was purified by silica gel column chromatography (toluene/acetone = 10/1 → 4/1) to give compound **48** (58.6 mg, 75%) as a white amorphous solid; $[\alpha]_D$ +31.1° (c 1.0, $CHCl_3$); ^1H NMR (500 MHz, $CDCl_3$) δ 8.05–6.67 (m, 32 H, 7 Ar, $CF_3C(=O)NH$), 6.40 (d, 1 H, $J_{2,NH}$ = 7.0 Hz, NH-2[d]), 5.77 (near quin, 1 H, $J_{5,6a}$ = $J_{5,6b}$ = 6.8 Hz, $J_{4,5}$ = 15.0 Hz, H-5[Cer]), 5.68 (dd, 1 H, $J_{1,2}$ = 8.0 Hz, $J_{2,3}$ = 10.5 Hz, H-2[b]), 5.63 (d, 1 H, $J_{2,NH}$ = 9.5 Hz, NH-2[Cer]), 5.60 (d, 1 H, $J_{1,2}$ = 3.5 Hz, H-1[f]), 5.48–5.36 (m, 4 H, H-2[c], H-4[c], H-3[Cer], H-4[Cer]), 5.31 (d, 1 H, $J_{3,4}$ = 2.5 Hz, H-4[e]), 5.26–5.24 (m, 2 H, H-1[c], H-4[d]), 5.16–5.09 (m, 5 H, H-2[a], H-3[b], H-2[f], H-3[f], H-4[f]), 5.01 (d, 1 H, $J_{1,2}$ = 8.0 Hz, H-1[d]), 4.91 (d, 1 H, H-1[b]), 4.84 (dd, 1 H, $J_{3,4}$= 3.3 Hz, $J_{2,3}$ = 11.3 Hz, H-3[d]), 4.68 (d, 1 H, J_{gem} = 11.5 Hz, $ArCH_2$), 4.61 (d, 1 H, $ArCH_2$), 4.50 (near t, 1 H, H-5[d]), 4.45–4.41 (m, 3 H, H-3[c], H-5[f], $ArCH_2$), 4.36–4.30 (m, 4 H, H-1[a], H-1[e], H-6a[e], H-2[Cer]), 4.23 (d, 1 H, $J_{3,4}$ = 2.5 Hz, H-4[b]), 4.14–4.05 (m, 5 H, H-4[a], H-6a[b], H-5[e], H-6b[e], $ArCH_2$), 4.02–3.97 (m, 2 H, H-6a[c], H-1a[Cer]), 3.85–3.80 (m, 2 H, H-6b[b], H-6a[d]), 3.76 (s, 3 H, OMe), 3.74–3.68 (m,

7 H, H-3[a], H-6b[d], 2 OCH_2, OMe), 3.65–3.30 (m, 18 H, H-6a[a], H-6b[a], H-5[b], H-5[c], H-6b[c], H-2[e], H-3[e], H-1b[Cer], 8 OCH_2, 2 $NHCH_2$), 3.25 (near dt, 1 H, H-5[a]), 2.93 (m, 1 H, H-2[d]), 2.11 (s, 3 H, Ac), 2.11 (s, 3 H, Ac), 2.08 (s, 3 H, Ac), 2.08 (s, 3 H, Ac), 2.05 (s, 3 H, Ac), 2.04 (s, 3 H, Ac), 1.95–1.89 (m, 11 H, H-6a[Cer], H-6b[Cer], 3 Ac), 1.83 (s, 3 H, Ac), 1.70–1.66 (m, 2 H, $NHC(=O)CH_2$), 1.35–1.05 (m, 76 H, H-6[f], 26 CH_2[Cer], 2 ^{t}Bu, Ac), 0.89–0.86 (m, 6 H, 2 Me[Cer]); ^{13}C NMR (125 MHz, $CDCl_3$) δ 172.6, 171.7, 171.3, 170.6, 170.5, 170.5, 170.4, 170.4, 169.9, 169.8, 169.6, 169.5, 166.1, 165.9, 165.2, 165.1, 165.0, 159.3, 159.1, 157.5, 157.2, 157.0, 156.4, 137.1, 133.6, 133.6, 133.4, 130.5, 130.1, 129.8, 129.7, 129.6, 129.5, 129.5, 129.1, 128.9, 128.8, 128.7, 128.5, 128.5, 127.6, 126.8, 125.4, 125.3, 124.9, 117.1, 114.8, 113.9, 113.7, 101.7, 101.1, 100.6, 99.2, 98.4, 95.1, 81.6, 79.1, 77.6, 75.4, 74.8, 74.1, 73.5, 73.4, 73.4, 73.2, 72.9, 72.2, 71.4, 71.1, 71.1, 70.7, 70.6, 70.4, 70.3, 70.2, 70.1, 70.1, 69.3, 68.7, 68.5, 67.8, 67.5, 67.4, 66.0, 64.3, 62.0, 61.6, 61.3, 61.1, 55.9, 55.2, 55.2, 50.3, 39.8, 36.4, 35.1, 35.0, 32.3, 31.9, 31.1, 29.7, 29.7, 29.6, 29.6, 29.5, 29.5, 29.4, 29.3, 29.3, 29.0, 25.6, 22.7, 22.5, 20.8, 20.8, 20.7, 20.6, 20.6, 20.5, 20.5, 15.7, 14.1; HRMS (ESI) *m/z*: found $[M/2+Na]^+$ 1564.2121, $C_{161}H_{218}F_3N_3O_{52}$ calcd for $[M/2+Na]^+$ 1564.2121.

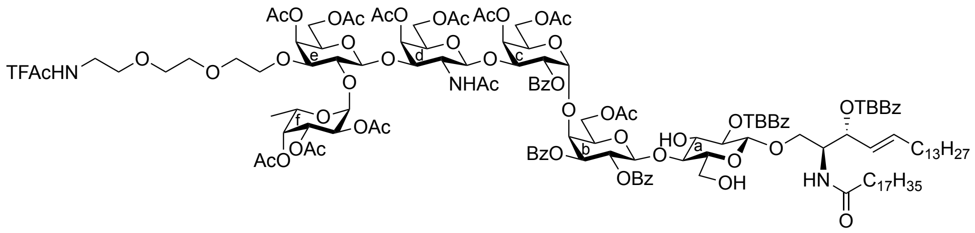

Compound 49

To a solution of compound **48** (154 mg, 49.9 μmol) in CH_2Cl_2 (3.3 mL) was added TFA (1.70 mL, 22.2 mmol) at 0 °C. After stirring for 30 min at 0 °C as the progress of the reaction was monitored by TLC (toluene/acetone = 3/1, developed twice), the reaction mixture was neutralized with satd. aq. $NaHCO_3$ and diluted with $CHCl_3$. The organic layer was washed with satd. aq. $NaHCO_3$ and brine, dried over Na_2SO_4, and concentrated. The residue was purified by silica gel column chromatography (toluene/acetone = 4/1) to give compound **49** (138 mg, 97%) as a white amorphous solid; $[\alpha]_D$ +38.2° (c 1.0, $CHCl_3$); ^{1}H NMR (500 MHz, $CDCl_3$) δ 8.06–7.16 (m, 24 H, 5 Ar, $CF_3C(=O)NH$), 6.51 (d, 1 H, $J_{2,NH}$ = 6.5 Hz, NH-2[d]), 5.89 (near quin, 1 H, $J_{5,6a}$ = $J_{5,6b}$ = 6.5 Hz, $J_{4,5}$ = 15.4 Hz, H-5[Cer]), 5.78–5.75 (m, 2 H, H-2[b], NH-2[Cer]), 5.62 (d, 1 H, $J_{1,2}$ = 3.0 Hz, H-1[f]), 5.58 (t, 1 H, $J_{2,3}$ = $J_{3,4}$ = 8.5 Hz, H-3[Cer]), 5.54–5.52 (m, 2 H, H-2[c], H-4[c]), 5.43 (dd, 1 H, H-4[Cer]), 5.39 (d, 1 H, $J_{3,4}$ = 3.0 Hz, H-4[d]), 5.32 (d, 1 H, $J_{3,4}$ = 3.0 Hz, H-4[e]), 5.28 (dd, 1 H, $J_{3,4}$ = 2.8 Hz, $J_{2,3}$ = 10.8 Hz, H-3[b]), 5.20–5.16 (m, 3 H, H-1[d], H-3[f], H-4[f]), 5.14–5.10 (m, 3 H, H-2[a], H-1[c], H-2[f]), 5.01 (dd, 1 H, $J_{2,3}$ = 11.0 Hz, H-3[d]), 4.87 (d, 1 H, $J_{1,2}$ = 8.0 Hz, H-1[b]), 4.51–4.37 (m, 6 H, H-1[a], H-3[c], H-5[d], H-1[e], H-5[f], H-2[Cer]), 4.29–4.24 (m, 3 H, H-4[b], H-6a[c], H-6a[e]), 4.14–4.07 (m, 4 H, H-6a[b],

H-6b^{c}, H-5^{e}, OH-3^{a}), 4.03–3.97 (m, 3 H, H-5^{b}, H-5^{c}, H-6b^{e}), 3.93–3.86 (m, 3 H, H-3^{a}, H-4^{a}, H-1a^{Cer}), 3.76–3.68 (m, 3 H, H-6b^{b}, H-6a^{d}, OCH_2), 3.62–3.26 (m, 16 H, H-6a^{a}, H-6b^{d}, H-2^{e}, H-3^{e}, H-1b^{Cer}, 9 OCH_2, 2 NHCH_2), 3.15 (br d, 1 H, H-5^{a}), 3.02–2.96 (m, 2 H, H-6b^{a}, H-2^{d}), 2.85 (br d, 1 H, OH-6^{a}), 2.14 (s, 3 H, Ac), 2.12–2.11 (m, 9 H, 3 Ac), 2.08 (s, 3 H, Ac), 2.07 (s, 3 H, Ac), 2.00–1.97 (m, 4 H, H-6a^{Cer}, H-6b^{Cer}, NHC(=O)CH_2), 1.94 (s, 3 H, Ac), 1.92 (s, 3 H, Ac), 1.87 (s, 3 H, Ac), 1.86 (s, 3 H, Ac), 1.50–1.22 (m, 73 H, 26 CH_2^{Cer}, 2 ^{t}Bu, Ac), 1.08 (d, 3 H, $J_{5,6}$ = 6.5 Hz, H-6^{f}), 0.89–0.85 (m, 6 H, 2 Me^{Cer}); ^{13}C NMR (125 MHz, $CDCl_3$) δ 172.7, 172.1, 171.2, 170.6, 170.4, 170.2, 170.1, 169.8, 169.6, 169.5, 166.0, 165.9, 165.7, 165.6, 165.2, 157.8, 157.5, 157.2, 157.0, 157.0, 138.5, 137.9, 133.7, 133.4, 130.0, 129.8, 129.8, 129.6, 129.4, 129.0, 128.9, 128.8, 128.8, 128.7, 128.6, 128.5, 128.4, 128.2, 127.3, 127.2, 125.5, 125.4, 125.3, 124.9, 119.4, 117.1, 114.8, 102.2, 101.8, 99.6, 99.2, 98.1, 95.1, 81.5, 81.0, 74.3, 74.0, 73.9, 73.5, 73.3, 73.0, 72.8, 72.5, 71.9, 71.5, 71.2, 70.8, 70.6, 70.4, 70.3, 70.3, 70.2, 70.1, 69.5, 69.3, 69.2, 68.7, 68.5, 67.8, 67.5, 66.1, 64.3, 62.3, 61.9, 61.5, 61.4, 59.6, 56.0, 53.8, 50.4, 39.8, 36.8, 35.2, 35.1, 32.3, 31.9, 31.1 31.1, 29.7, 29.7, 29.6, 29.6, 29.5, 29.4, 29.4, 29.3, 29.3, 28.9, 25.6, 22.7, 22.6, 20.8, 20.8, 20.7, 20.7, 20.7, 20.6, 20.6, 20.3, 15.7, 14.1; HRMS (ESI) *m/z*: found [M/2+Na]$^+$ 1444.1546, $C_{145}H_{202}F_3N_3O_{50}$ calcd for [M/2+Na]$^+$ 1444.1546.

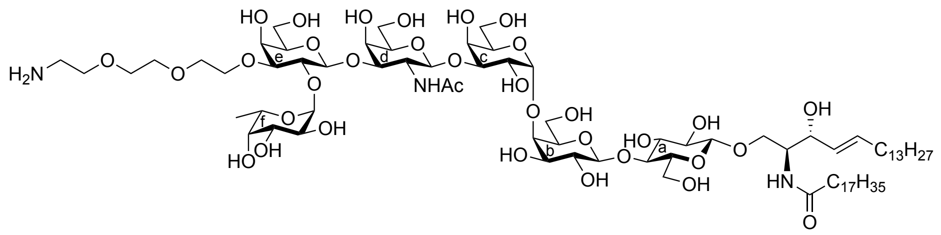

Compound 50

To a solution of compound **49** (129 mg, 45.4 µmol) in MeOH/THF = 1/1 (15 mL) was added 1 M NaOH aq. (908 µL, 0.908 mmol). After stirring for 18 h at ambient temperature as the progress of the reaction was monitored by TLC ($CHCl_3$/MeOH/H_2O/AcOH = 5/4/1/0.2), the reaction mixture was neutralized with Muromac (H^+), the resin was filtered through cotton, and washed with $CHCl_3$/MeOH = 1/1. The combined filtrate and washings were concentrated and co-evaporated with EtOH. The residue was purified by silica gel column chromatography ($CHCl_3$/MeOH/H_2O/28% NH_3 aq. = 5/1/0.05/0 → 5/4/1/0 → 3/3/1/0.1) to give compound **50** (75.3 mg, 98%) as a white amorphous solid; $[\alpha]_D$ +13.2° (c 1.0, $CHCl_3$/MeOH = 1/1); ^{1}H NMR (500 MHz, $CDCl_3$/CD_3OD = 1/1) δ 5.70 (near quin, 1 H, $J_{5,6a}$ = $J_{5,6b}$ = 6.8 Hz, $J_{4,5}$ = 15.2 Hz, H-5^{Cer}), 5.46 (dd, 1 H, $J_{3,4}$ = 7.8 Hz, H-4^{Cer}), 5.30 (d, 1 H, $J_{1,2}$ = 3.5 Hz, H-1^{f}), 4.98 (d, 1 H, $J_{1,2}$ = 4.0 Hz, H-1^{c}), 4.61–4.30 (4 d, 4 H, H-1^{a}, H-1^{b}, H-1^{d}, H-1^{e}), 4.24–3.31 (m, 48 H, H-2^{a}, H-3^{a}, H-4^{a}, H-5^{a}, H-6a^{a}, H-6b^{a}, H-2^{b}, H-3^{b}, H-4^{b}, H-5^{b}, H-6a^{b}, H-6b^{b}, H-2^{c}, H-3^{c}, H-4^{c}, H-5^{c},

H-6a^c, H-6b^c, H-2^d, H-3^d, H-4^d, H-5^d, H-6a^d, H-6b^d, H-2^e, H-3^e, H-4^e, H-5^e, H-6a^e, H-6b^e, H-2^f, H-3^f, H-4^f, H-5^f, H-1a^{Cer}, H-1b^{Cer}, H-2^{Cer}, H-3^{Cer}, 5 OCH_2), 3.18 (m, 2 H, NHCH_2), 2.19–2.16 (m, 2 H, NHC(=O)CH_2), 2.05–2.01 (m, 5 H, H-6a^{Cer}, H-6b^{Cer}, Ac), 1.60–1.58 (m, 2 H, NHC(=O)CH_2CH_2), 1.37–1.24 (m, 53 H, H-6^f, 25 CH_2^{Cer}), 0.91–0.88 (m, 6 H, 2 Me^{Cer}); ^{13}C NMR (125 MHz, $CDCl_3$/CD_3OD = 1/1) δ 175.3, 174.1, 134.9, 130.2, 104.7, 104.5, 103.6, 103.1, 102.3, 99.9, 83.3, 80.4, 80.3, 78.8, 75.6, 75.5, 75.4, 75.3, 75.1, 74.1, 72.7, 72.5, 72.3, 71.8, 71.1, 70.8, 70.5, 69.7, 69.5, 69.3, 69.0, 68.6, 67.7, 67.2, 66.0, 62.3, 62.0, 61.2, 61.1, 58.0, 53.9, 52.5, 50.1, 40.2, 37.0, 32.9, 32.5, 32.1, 30.9, 30.2, 30.2, 30.2, 30.2, 30.1, 30.1, 30.1, 30.1, 30.0, 29.9, 29.9, 29.9, 29.8, 26.6, 26.2, 23.4, 23.2, 18.1, 16.3, 14.3; HRMS (ESI) *m*/*z*: found $[M+H]^+$ 1694.9937, $C_{80}H_{147}N_3O_{34}$ calcd for $[M+H]^+$ 1694.9939.

2. ^{1}H and ^{13}C NMR spectral data for new compounds

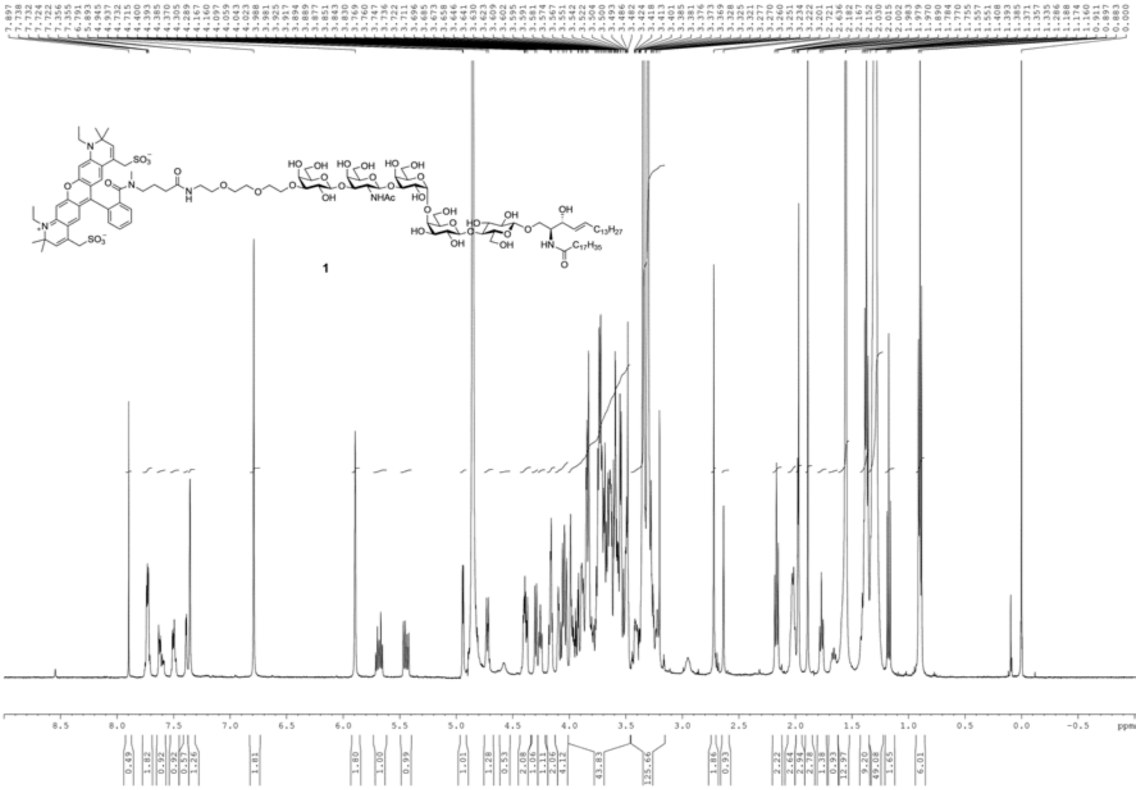

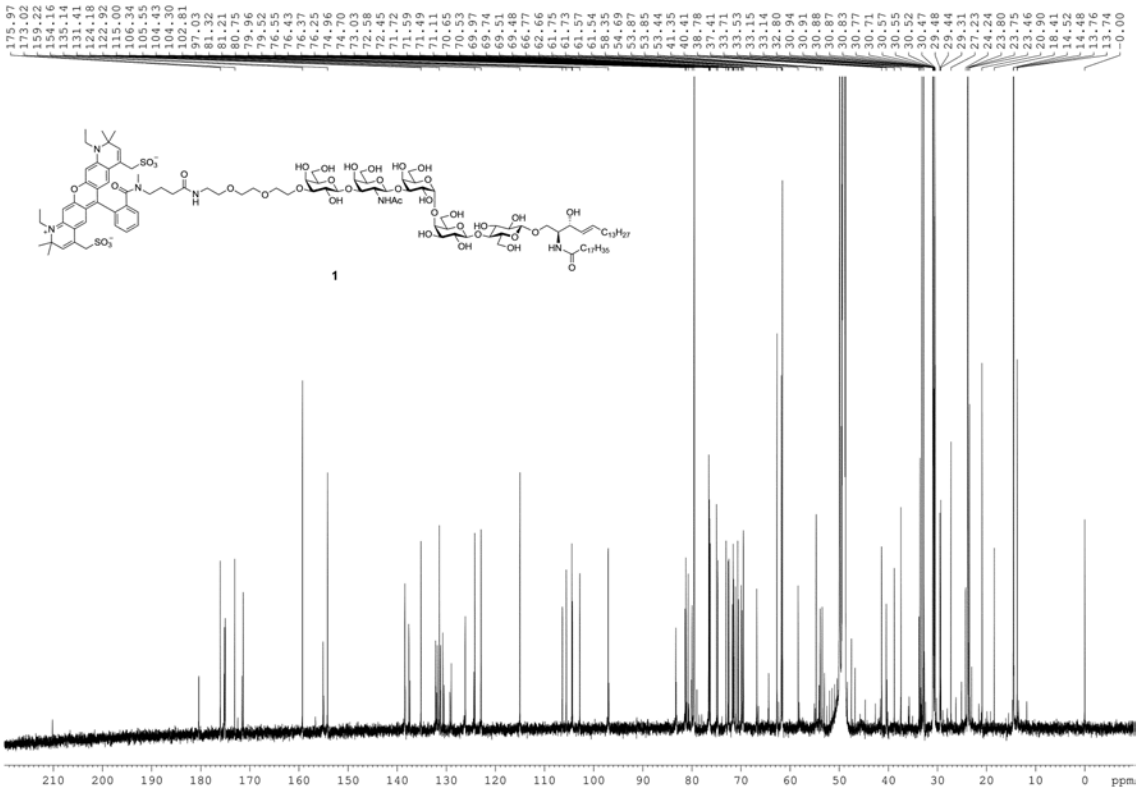

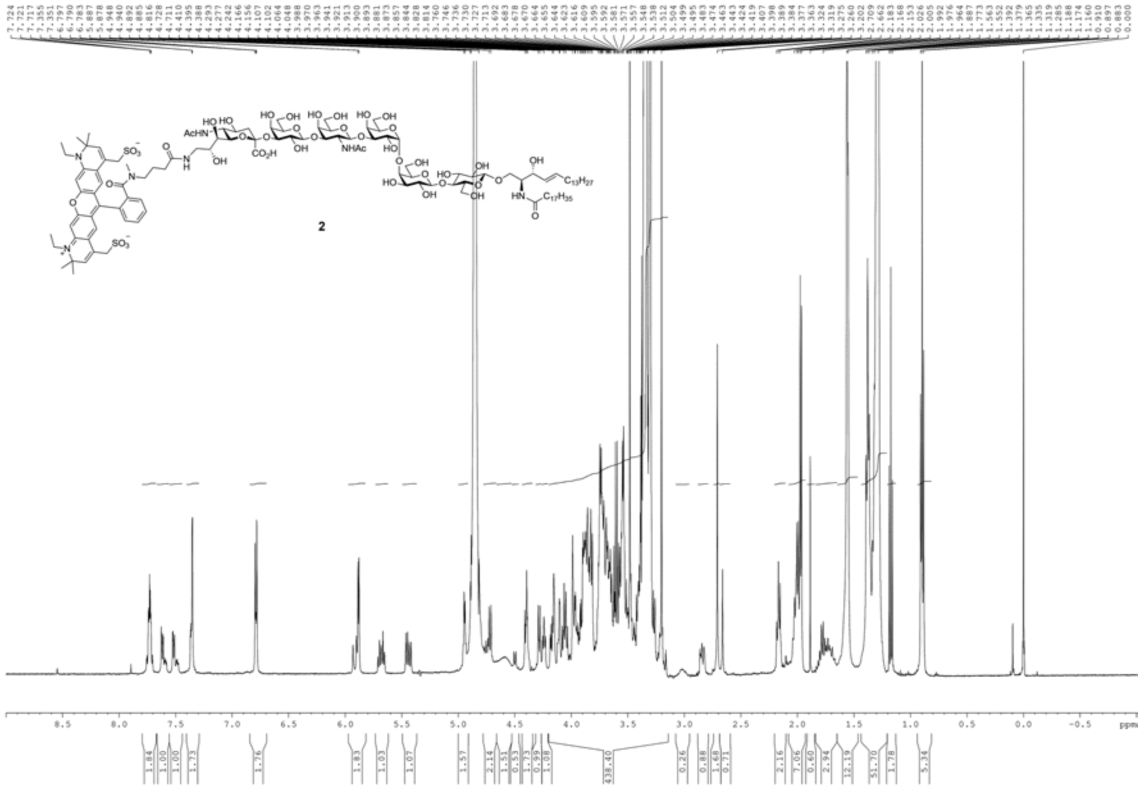
2
ppm

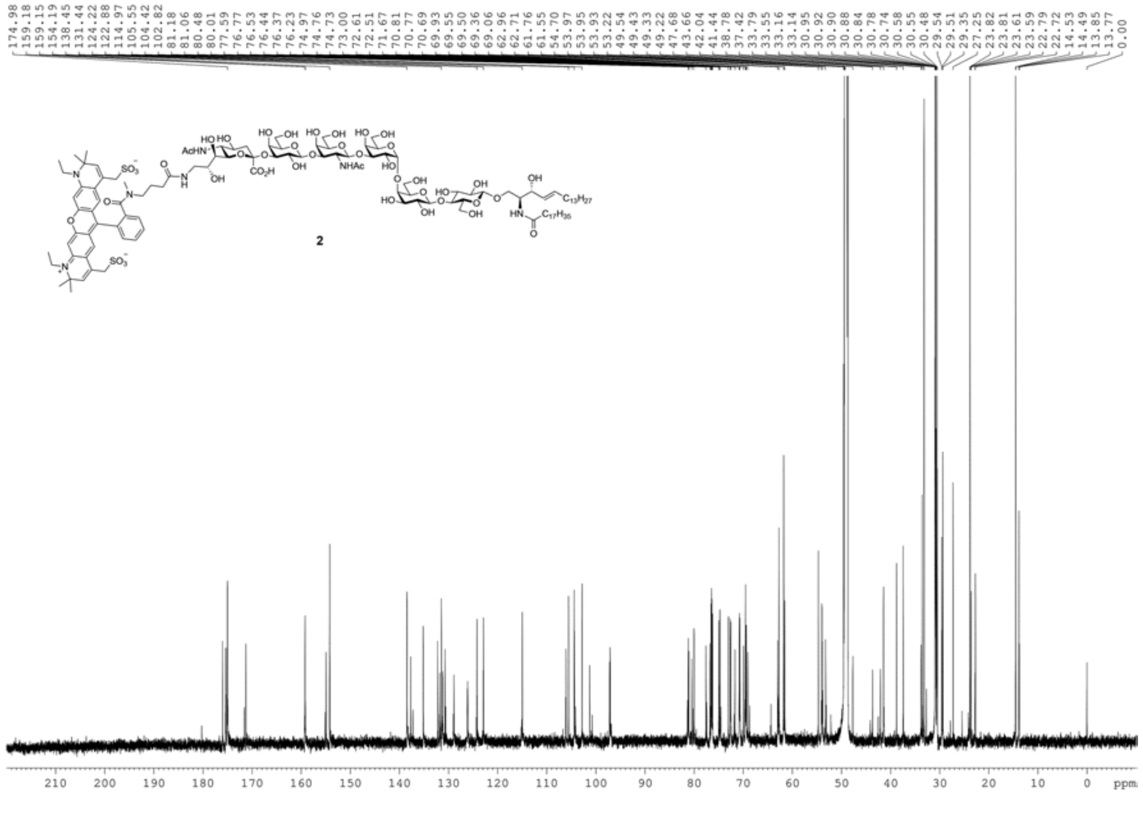
174.98
159.18
159.15
154.19
138.45
131.44
124.22
122.88
114.97
105.55
104.42
102.82
81.18
81.06
80.48
80.01
77.59
76.77
76.53
76.44
76.37
76.23
74.97
74.76
74.73
73.00
72.61
72.51
71.67
70.81
70.77
70.69
69.93
69.55
69.50
69.36
69.06
62.96
62.71
61.76
61.55
54.70
53.97
53.95
53.93
53.22
49.54
49.43
49.33
49.22
47.68
43.66
42.04
41.44
38.78
37.42
33.79
33.55
33.16
33.14
30.95
30.92
30.90
30.88
30.84
30.78
30.74
30.58
30.55
30.48
29.54
29.51
29.35
27.25
23.82
23.81
23.61
23.59
22.79
22.72
14.53
14.49
13.85
13.77
0.00
2
ppm

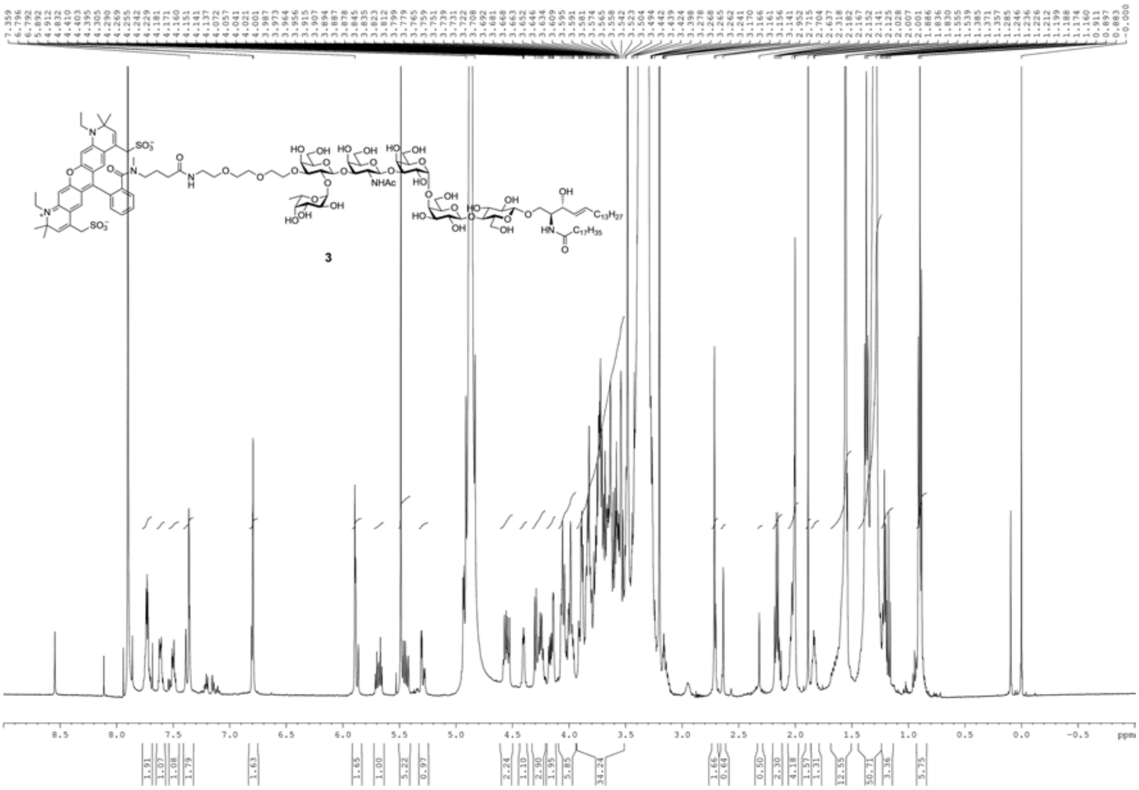
3
ppm

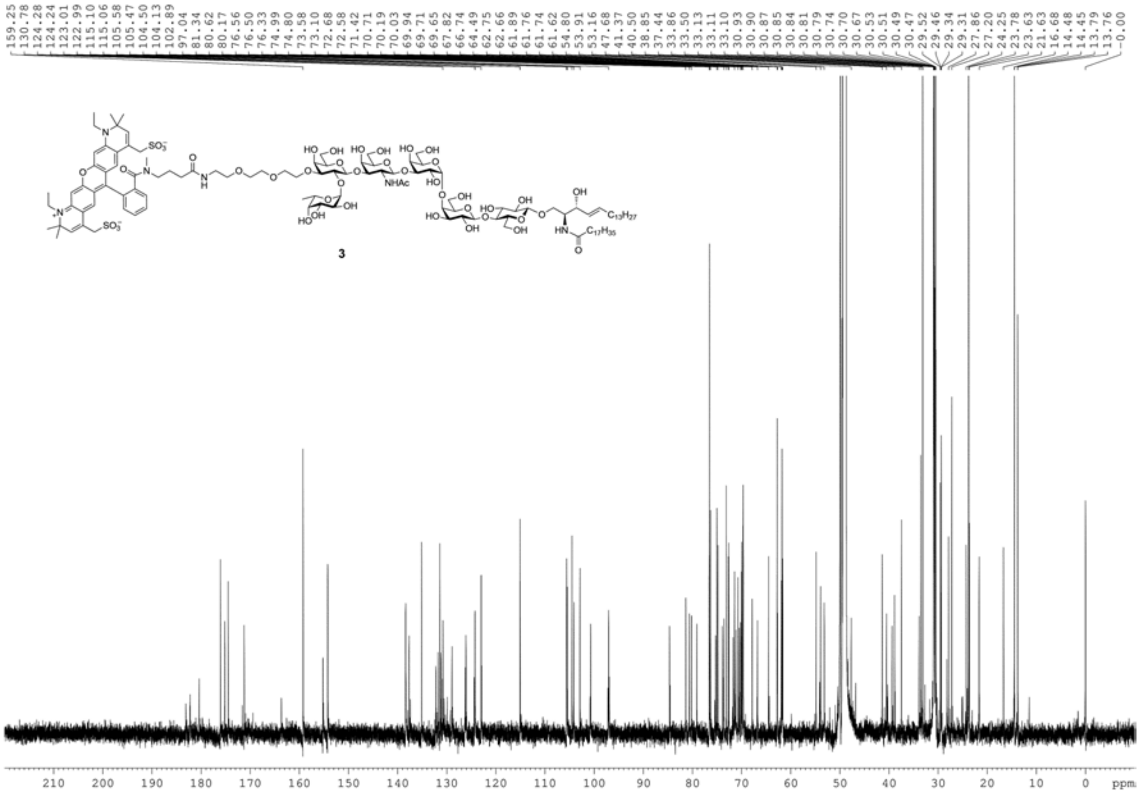
3
ppm

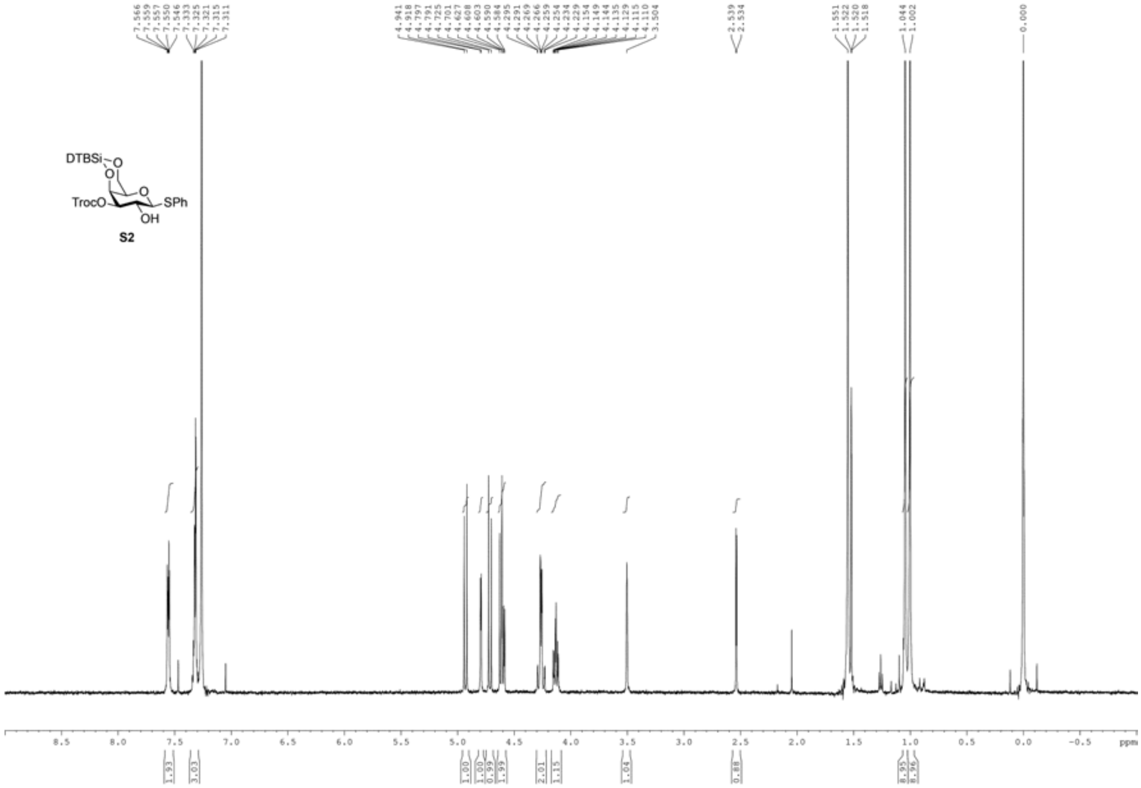
DTBSi
TrocO
SPh
OH
S2
ppm

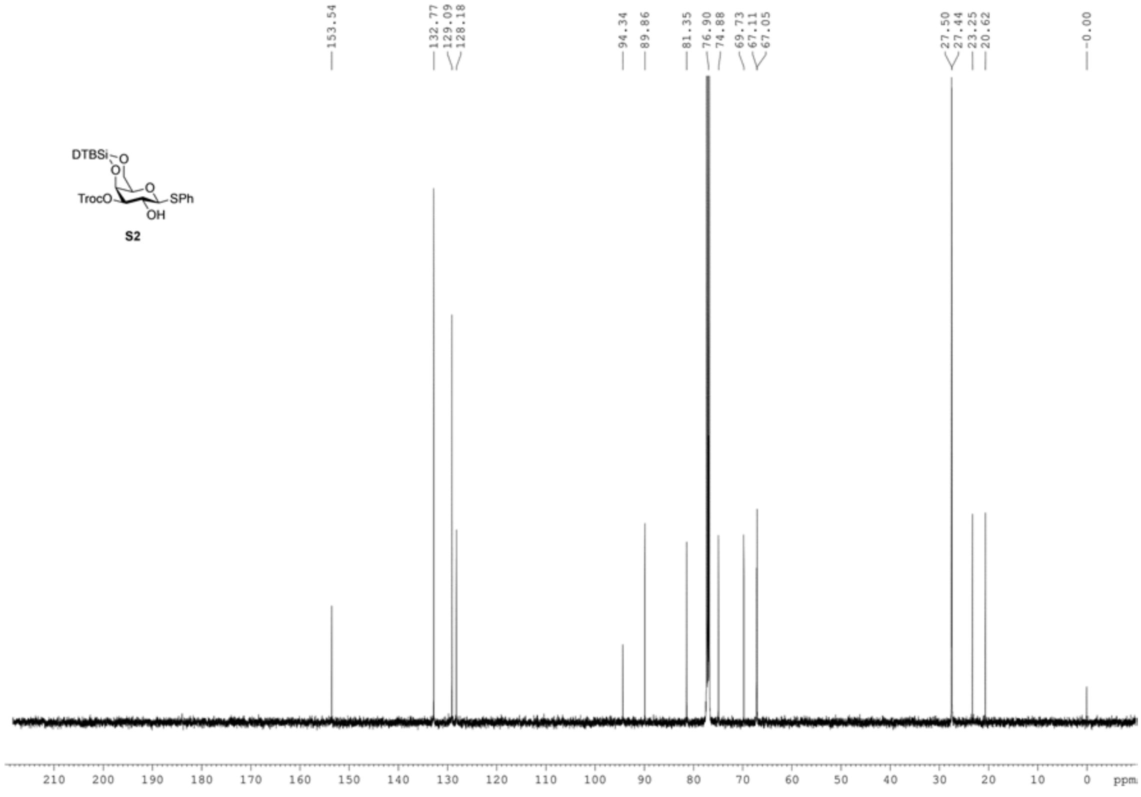
153.54
132.77
129.09
128.18
94.34
89.86
81.35
76.90
74.88
69.73
67.11
67.05
27.50
27.44
23.25
20.62
0.00
DTBSi
TrocO
SPh
OH
S2
ppm

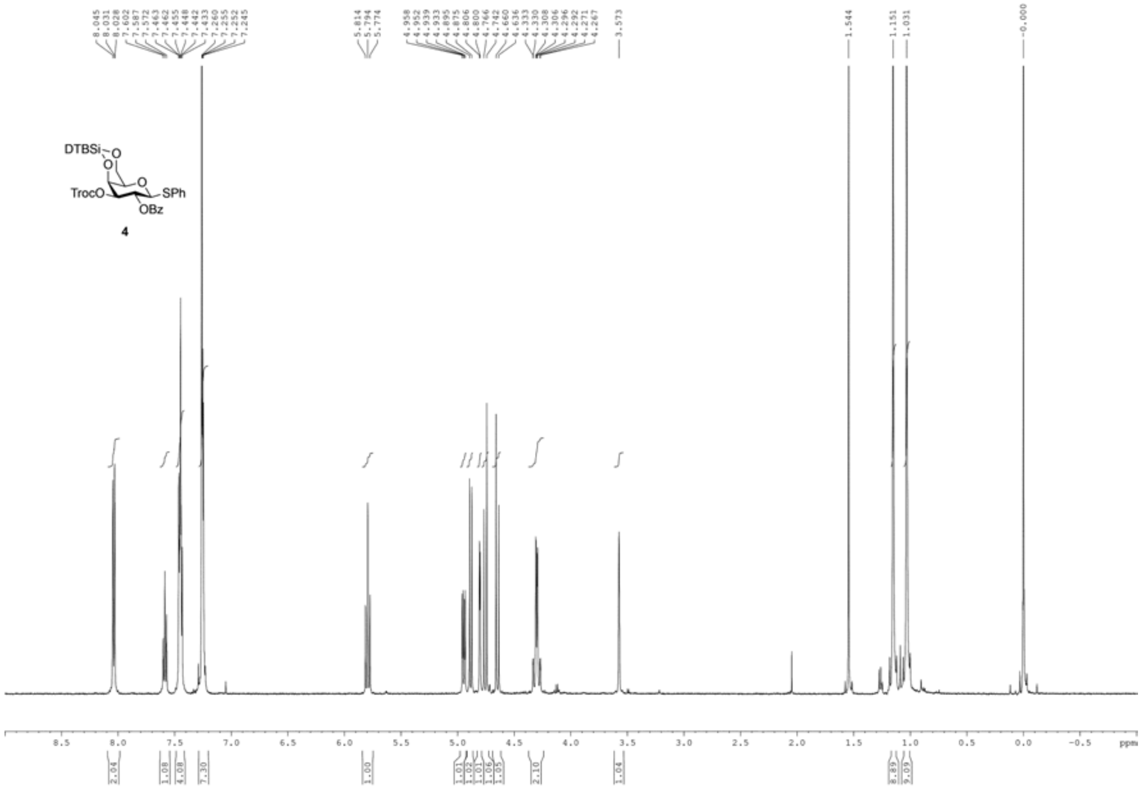

DTBSi
O
O
TrocO
O
SPh
OBz
4
8.045
8.031
8.028
7.602
7.587
7.572
7.463
7.462
7.455
7.448
7.442
7.433
7.260
7.255
7.252
7.245
5.814
5.794
5.774
4.958
4.952
4.939
4.933
4.895
4.875
4.806
4.800
4.766
4.742
4.660
4.636
4.333
4.330
4.308
4.306
4.296
4.292
4.271
4.267
3.573
1.544
1.151
1.031
0.000
2.04
1.08
4.08
7.30
1.00
1.01
1.02
1.01
1.06
1.05
2.10
1.04
8.89
9.09
ppm

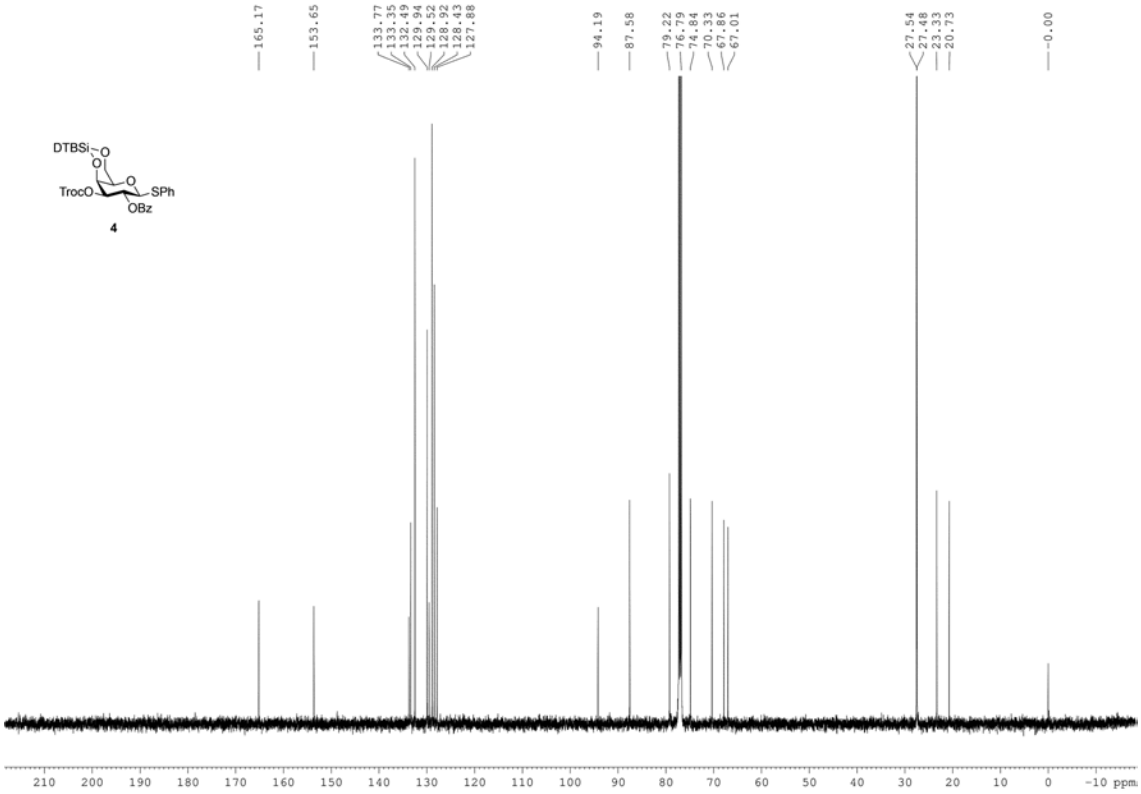

DTBSi
O
O
TrocO
O
SPh
OBz
4
165.17
153.65
133.77
133.35
132.49
129.94
129.52
128.92
128.43
127.88
94.19
87.58
79.22
76.79
74.84
70.33
67.86
67.01
27.54
27.48
23.33
20.73
0.00
ppm

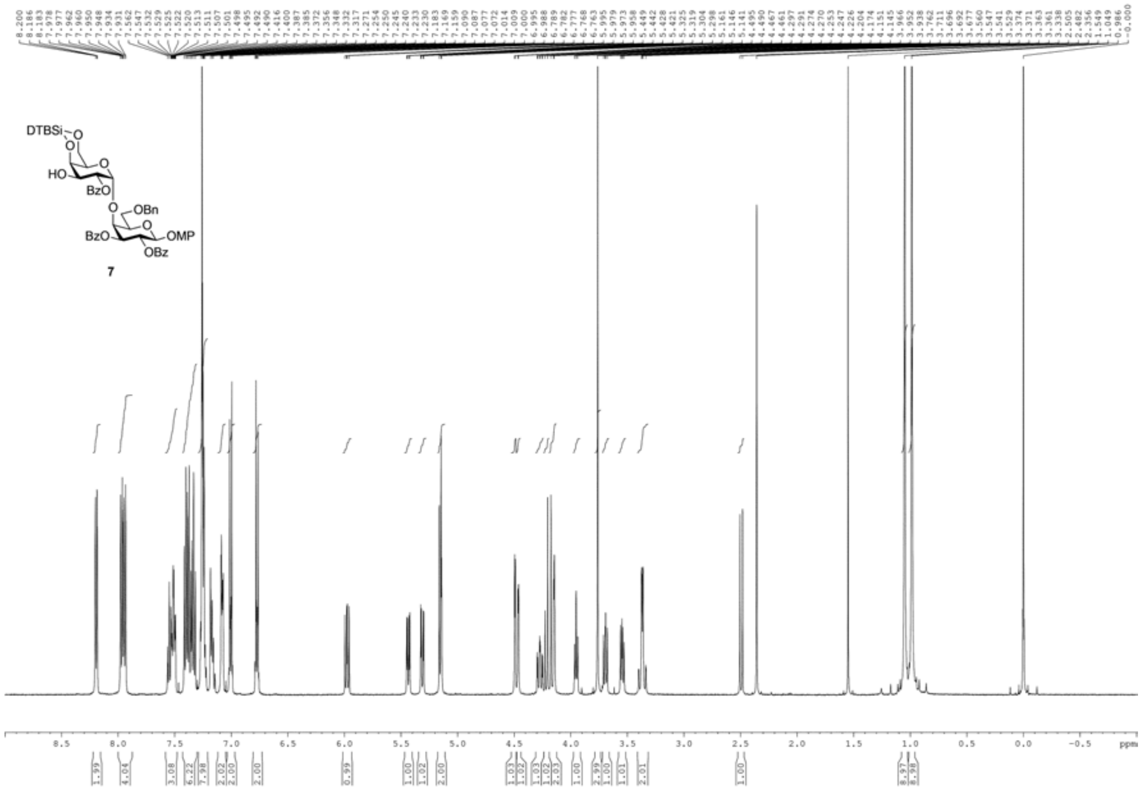
DTBSi
HO
BzO
OBn
BzO
OMP
OBz
7
ppm

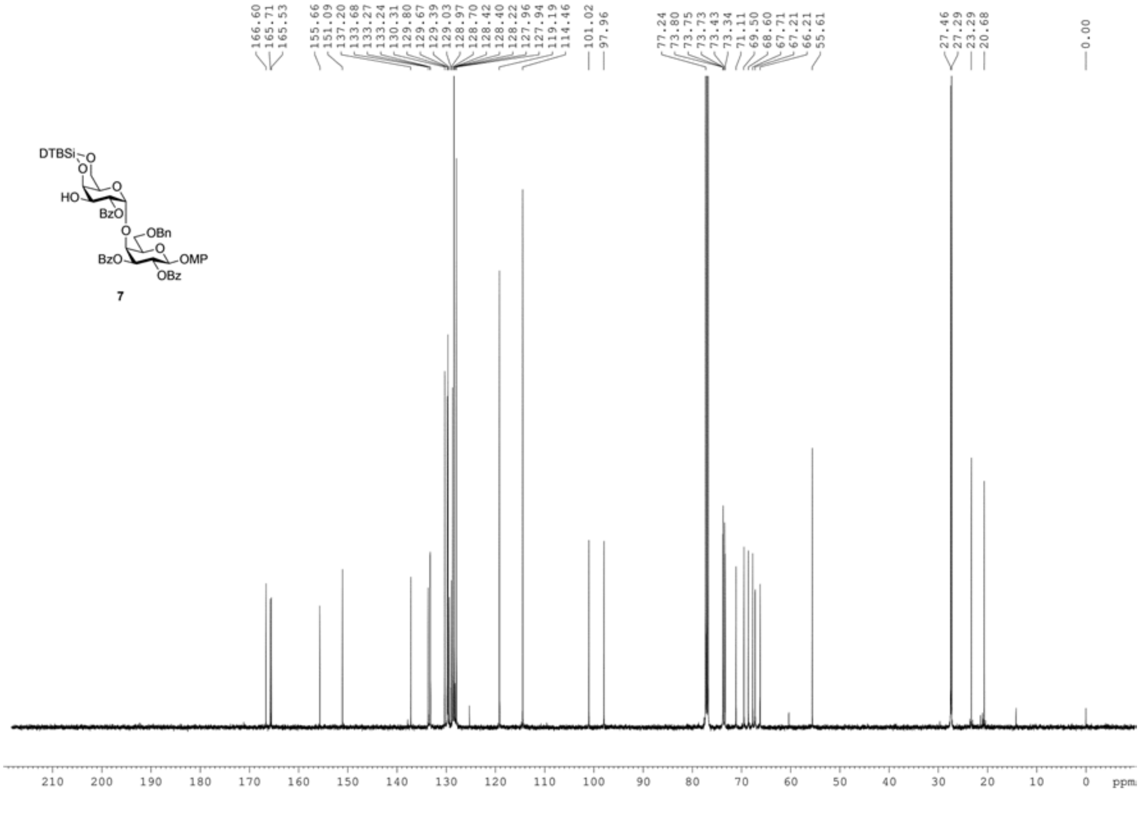
166.60
165.71
165.53
155.66
151.09
137.20
133.68
133.27
133.24
130.31
129.80
129.67
129.39
129.03
128.97
128.70
128.42
128.40
128.22
127.96
127.94
119.19
114.46
101.02
97.96
77.24
73.80
73.75
73.73
73.43
73.34
71.11
69.50
68.60
67.71
67.21
66.21
55.61
27.46
27.29
23.29
20.68
0.00
DTBSi
HO
BzO
OBn
BzO
OMP
OBz
7
ppm

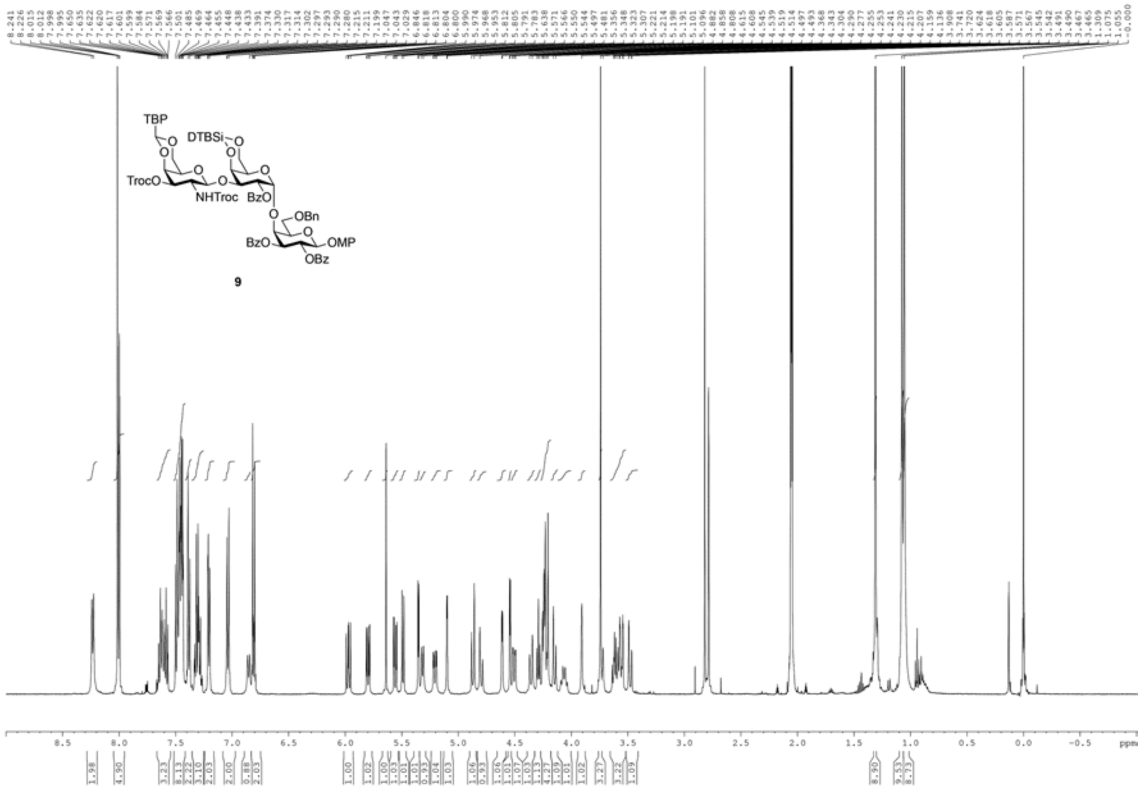
TBP
DTBSi
TrocO
NHTroc
BzO
OBn
BzO
OMP
OBz
9
ppm

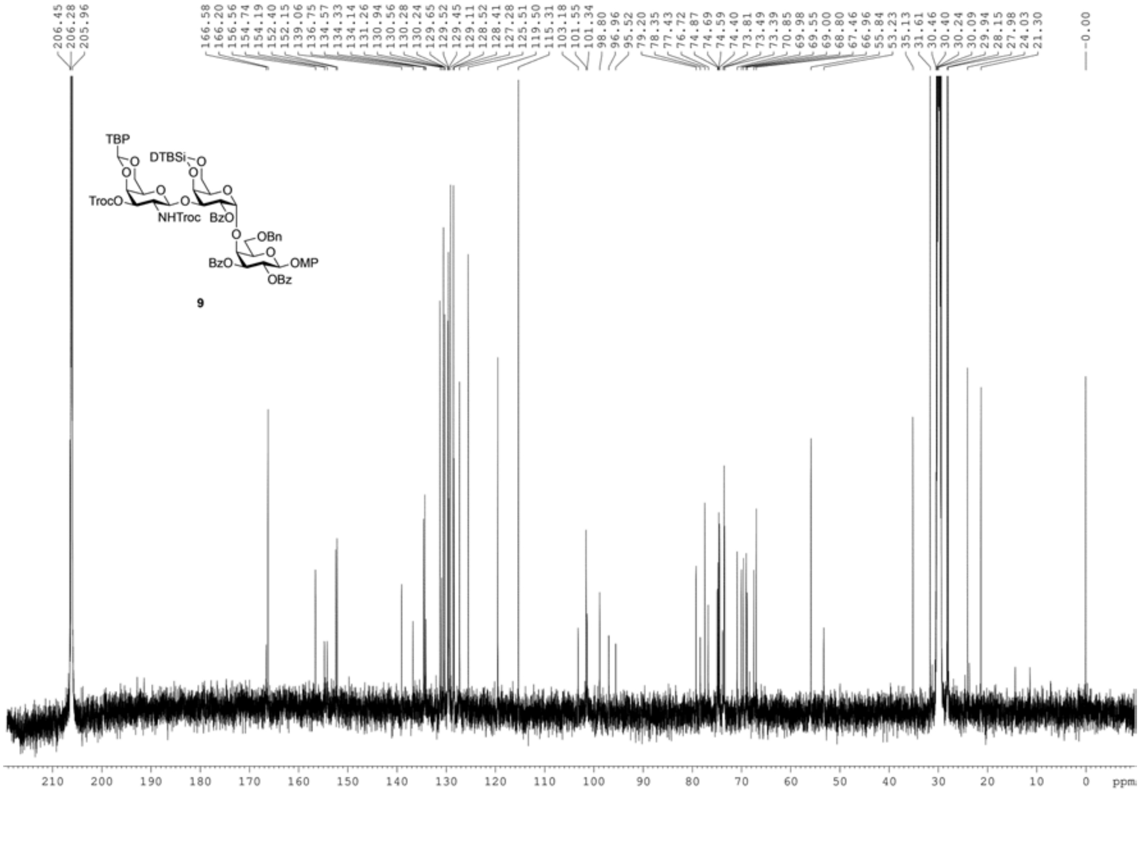
206.45
206.28
205.96
166.58
166.20
156.56
154.74
154.19
152.40
152.15
139.06
136.75
134.57
134.33
134.14
131.26
130.94
130.56
130.28
130.24
129.65
129.52
129.45
129.11
128.52
128.41
127.28
125.51
119.50
115.31
103.18
101.55
101.34
98.80
96.96
95.52
79.20
78.35
77.43
76.72
74.87
74.69
74.59
74.40
73.81
73.49
70.85
69.98
69.55
69.00
68.80
67.46
66.96
55.84
53.23
35.13
31.61
30.46
30.40
30.24
30.09
29.94
28.15
27.98
24.03
21.30
-0.00
TBP
DTBSi
TrocO
NHTroc
BzO
OBn
BzO
OMP
OBz
9
ppm

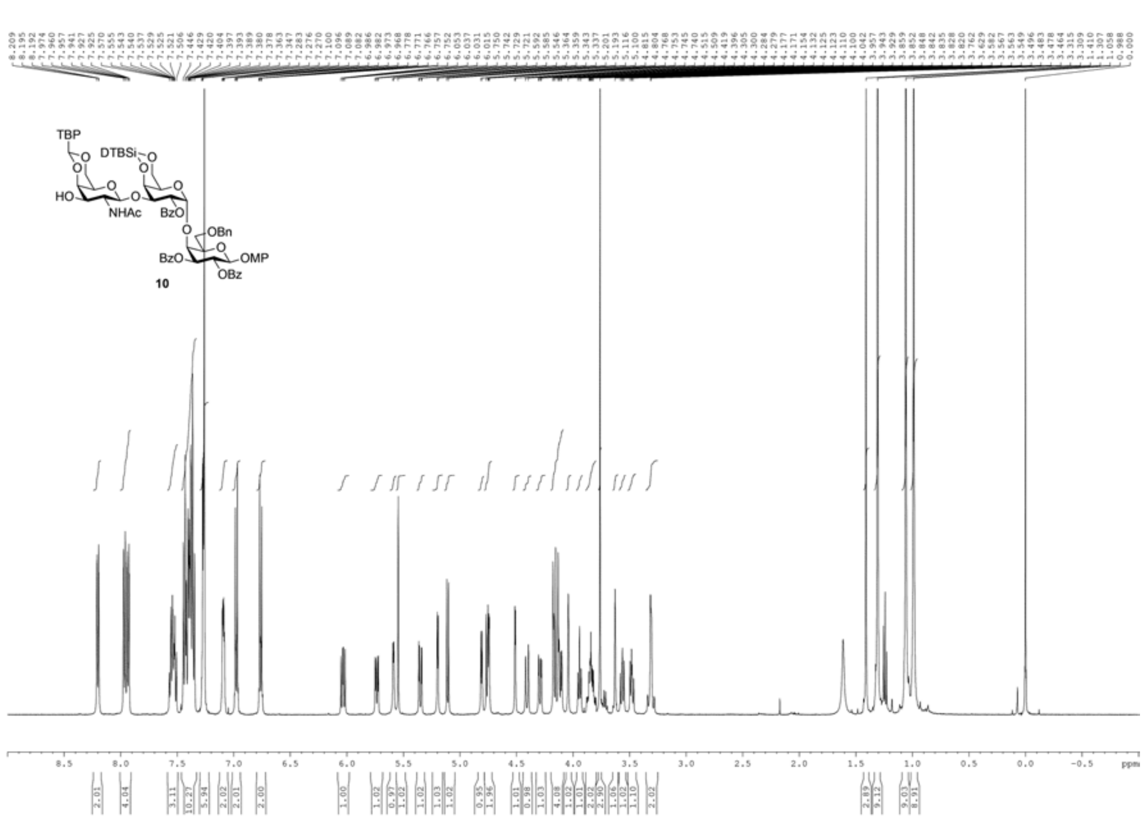

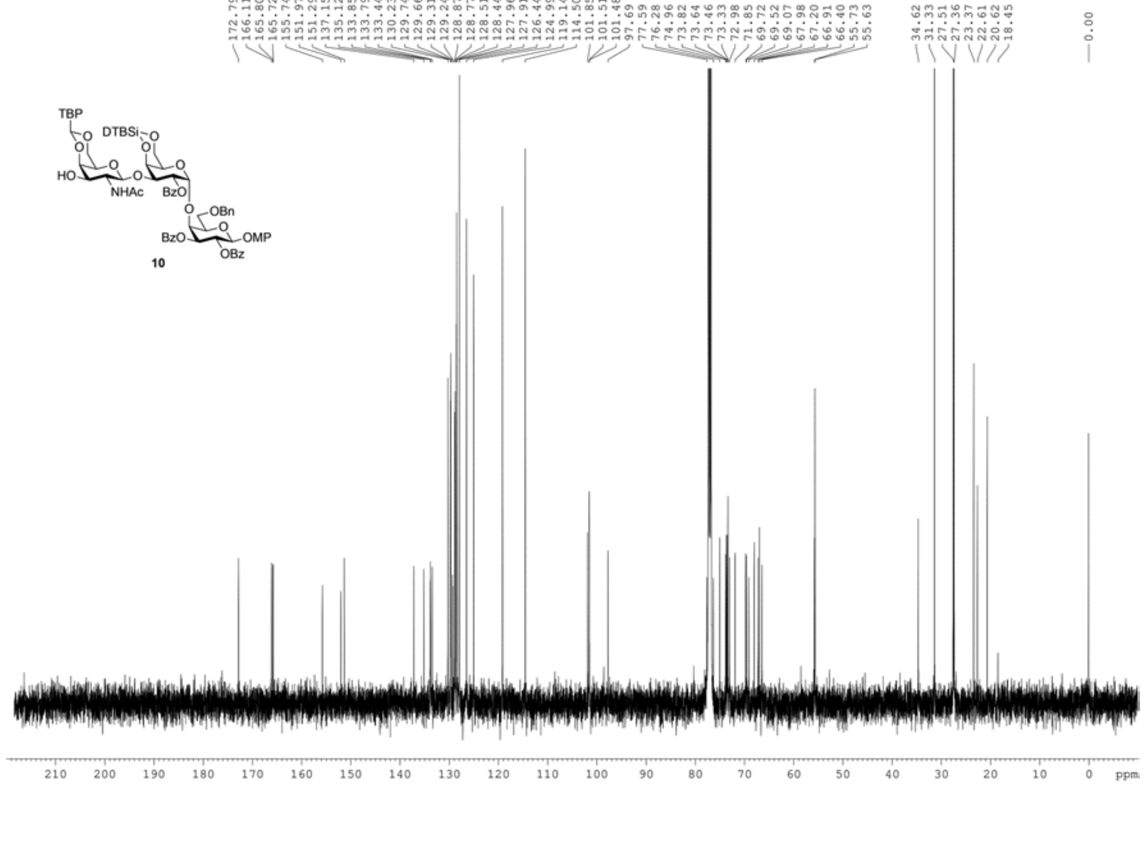

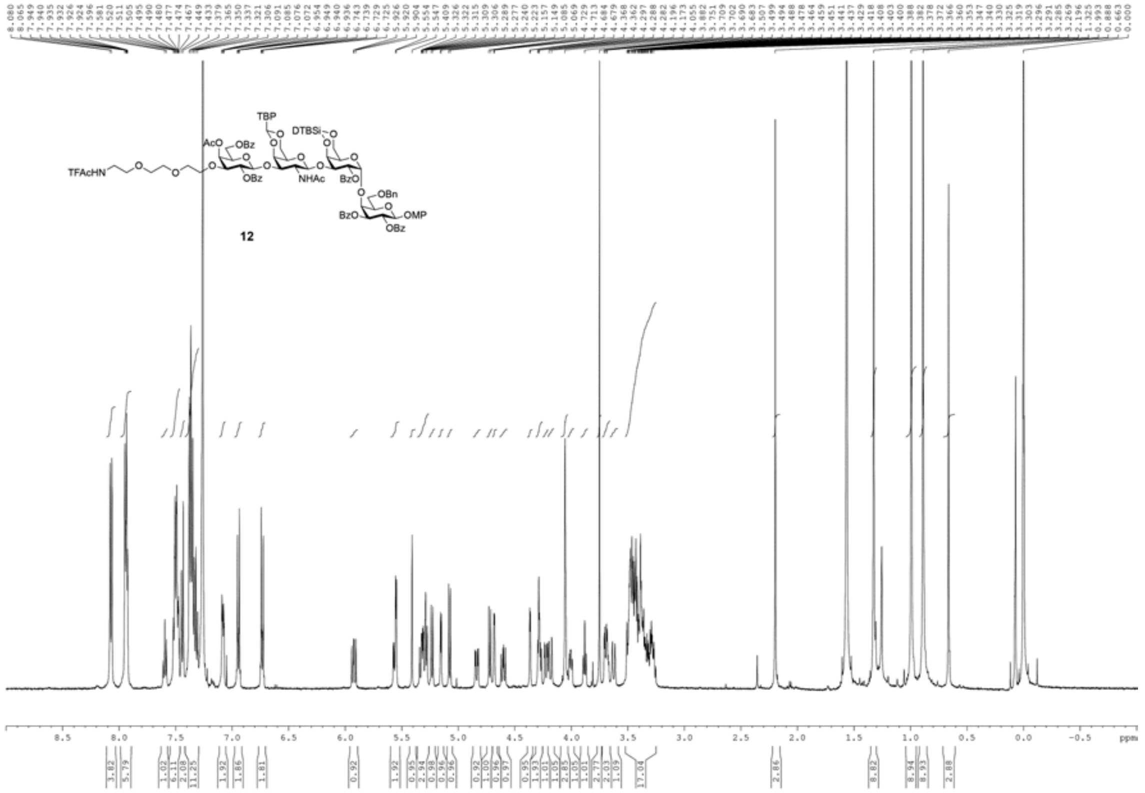

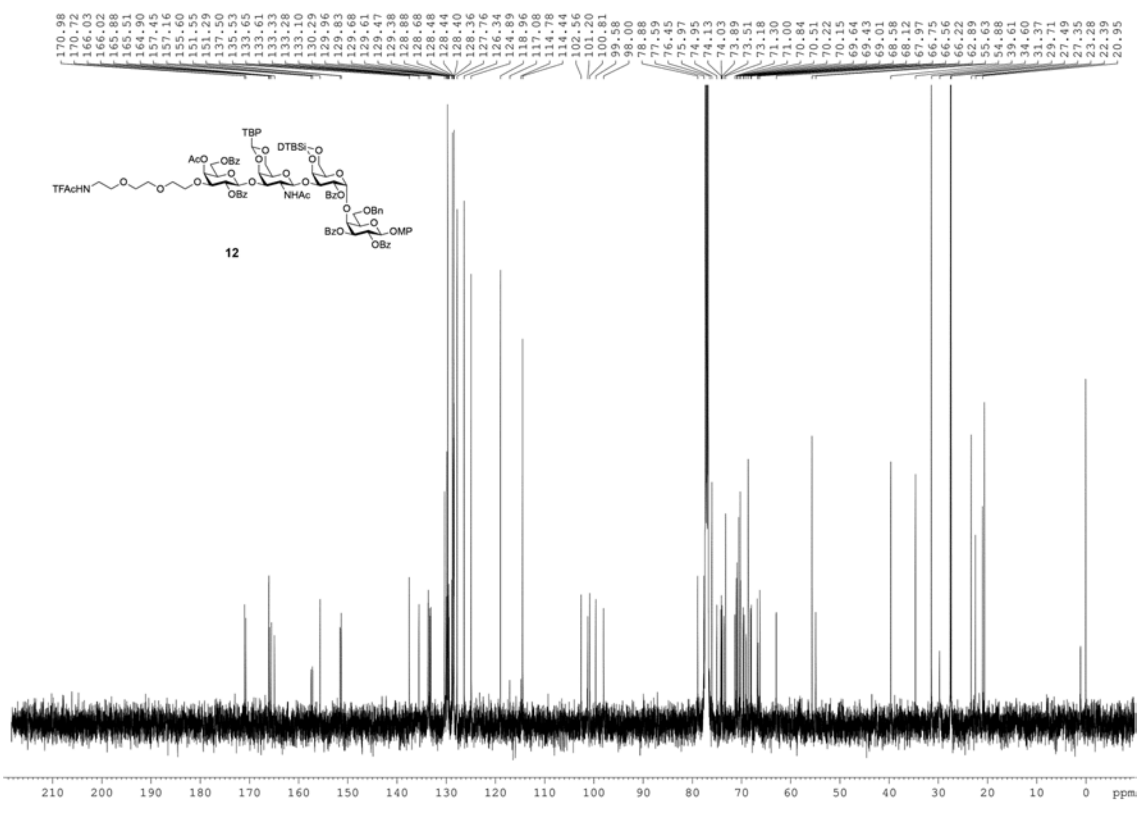

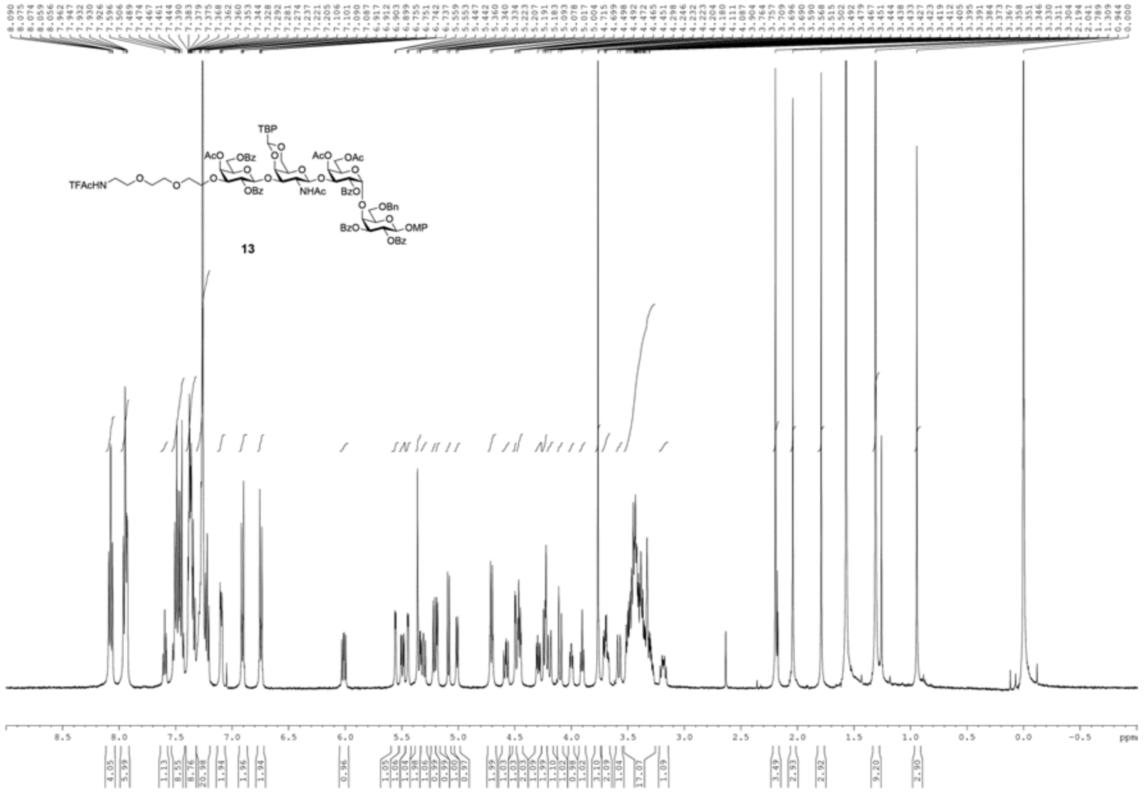
TFAcHN
13
ppm

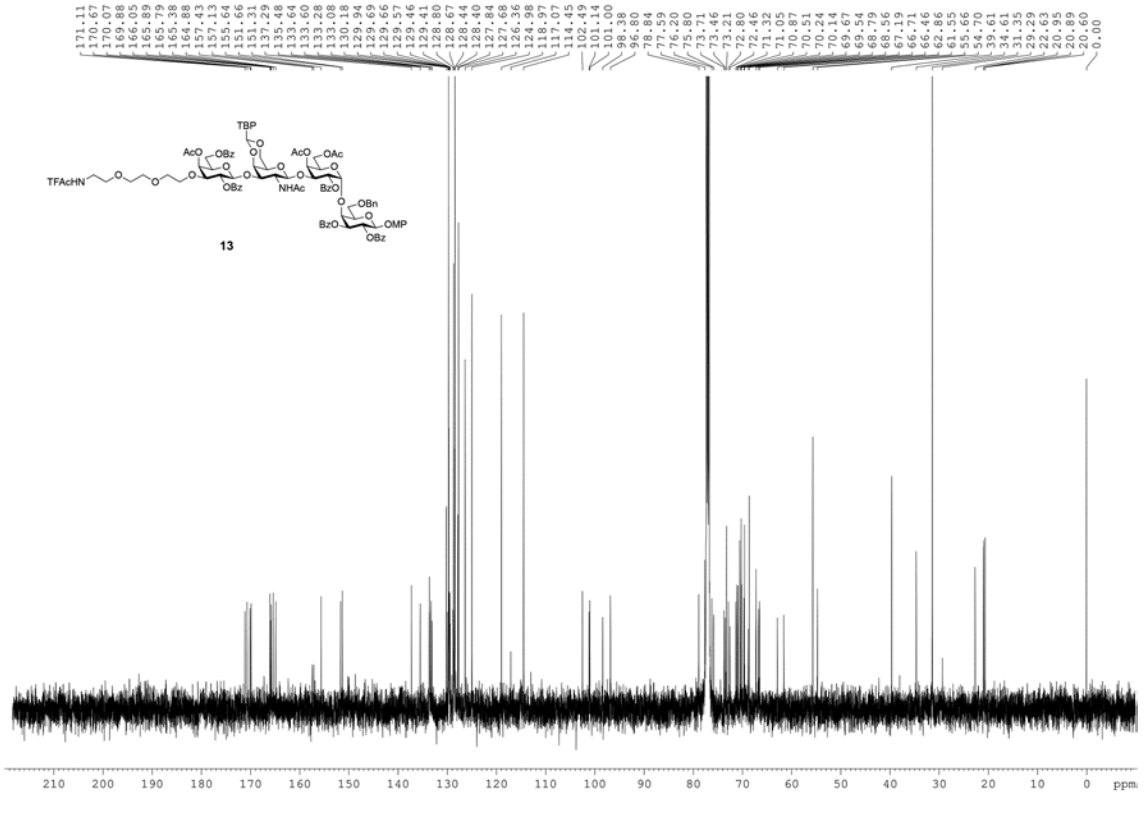
TFAcHN
13
ppm

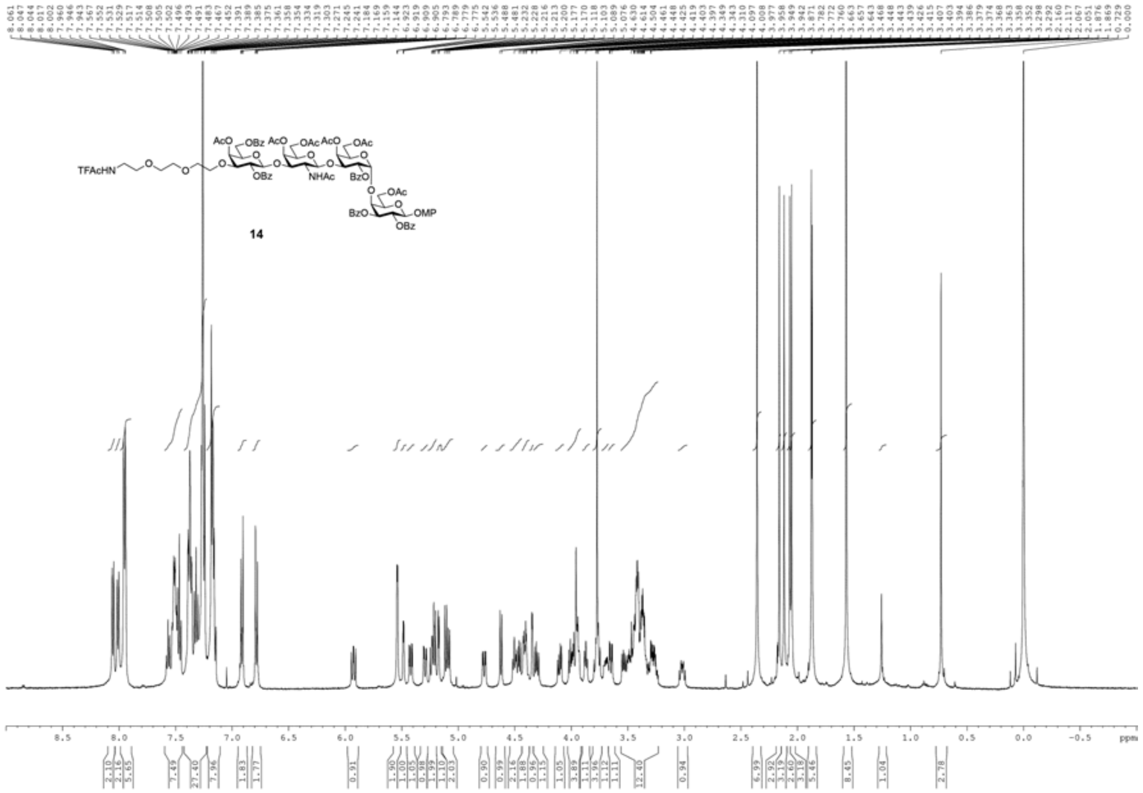

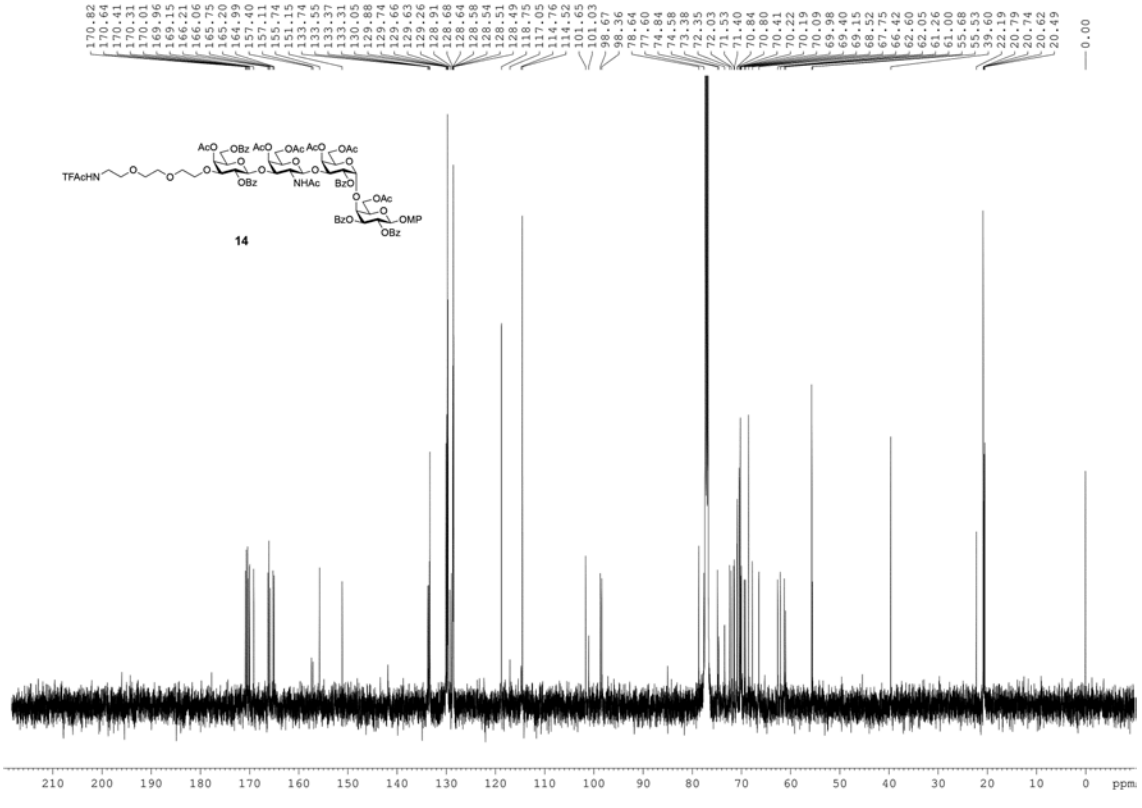

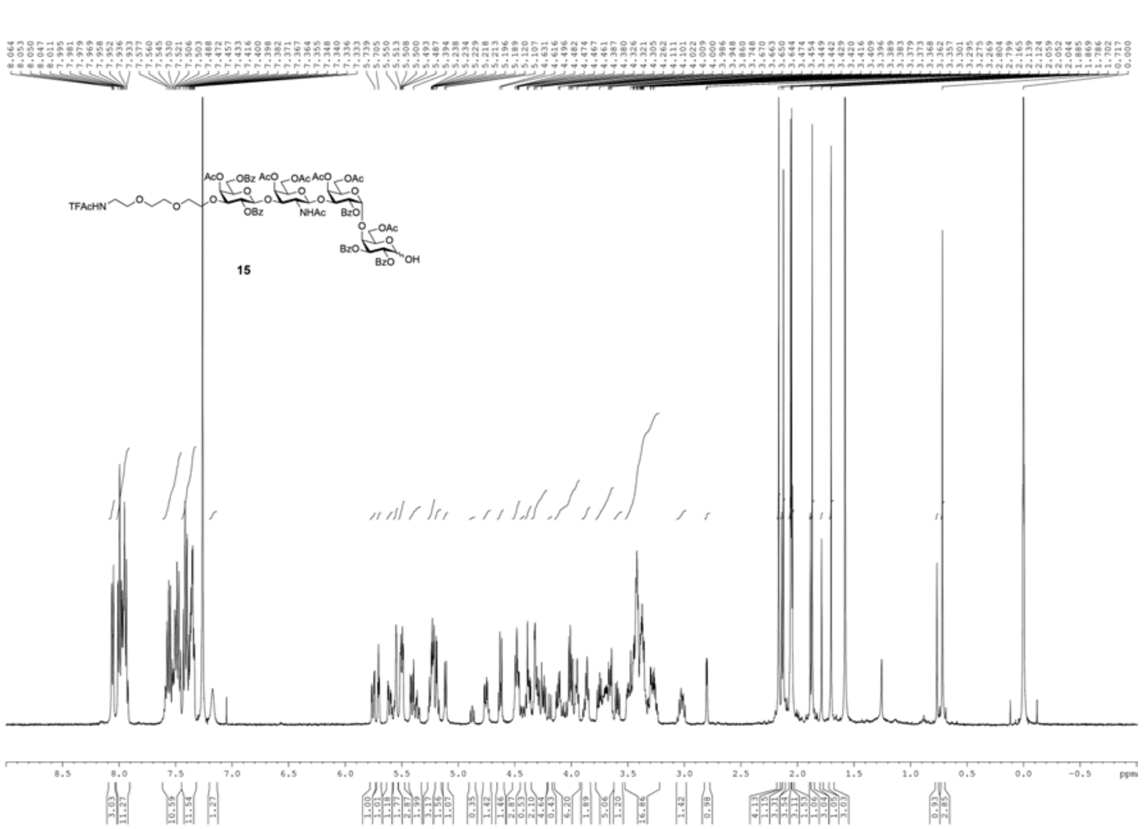

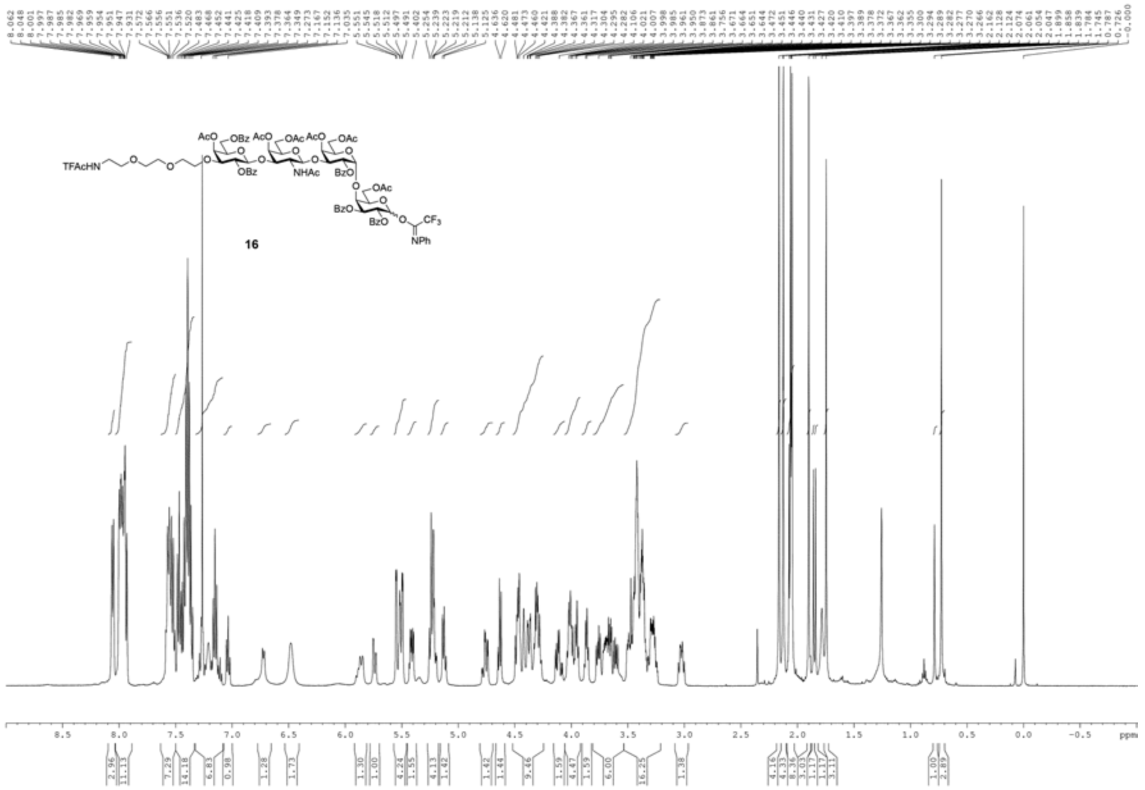

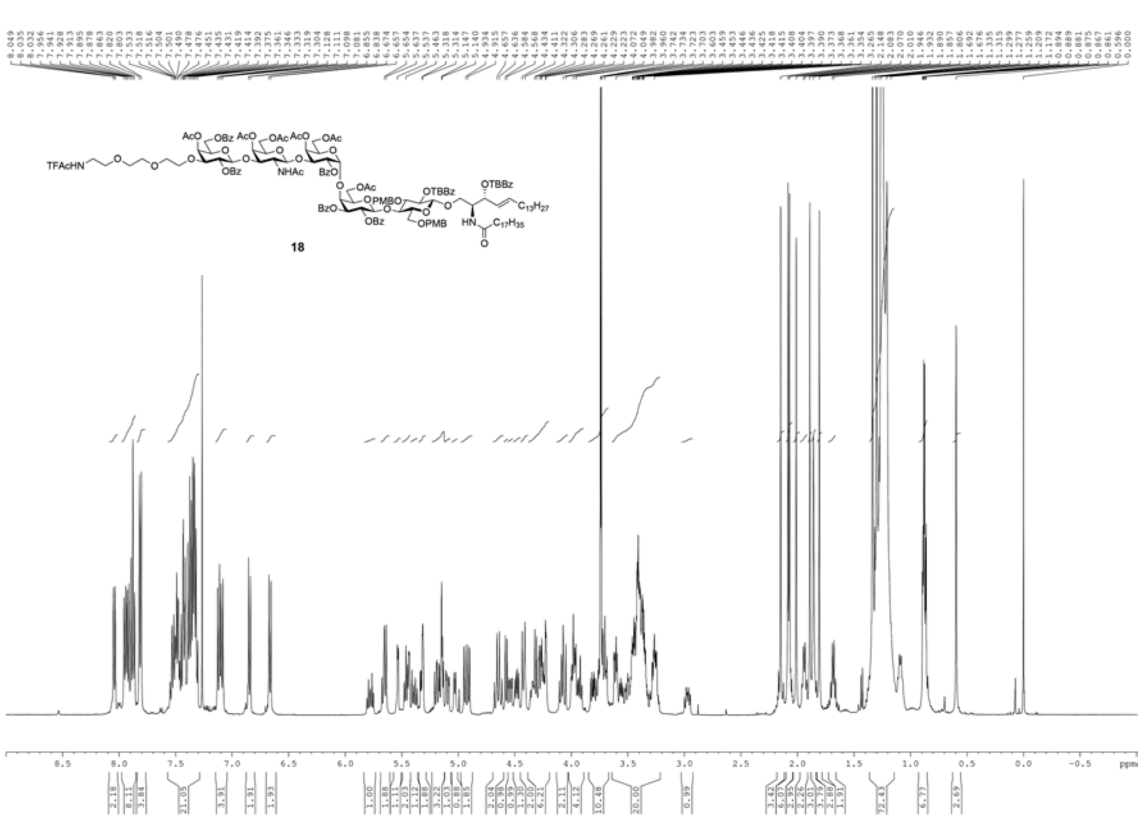

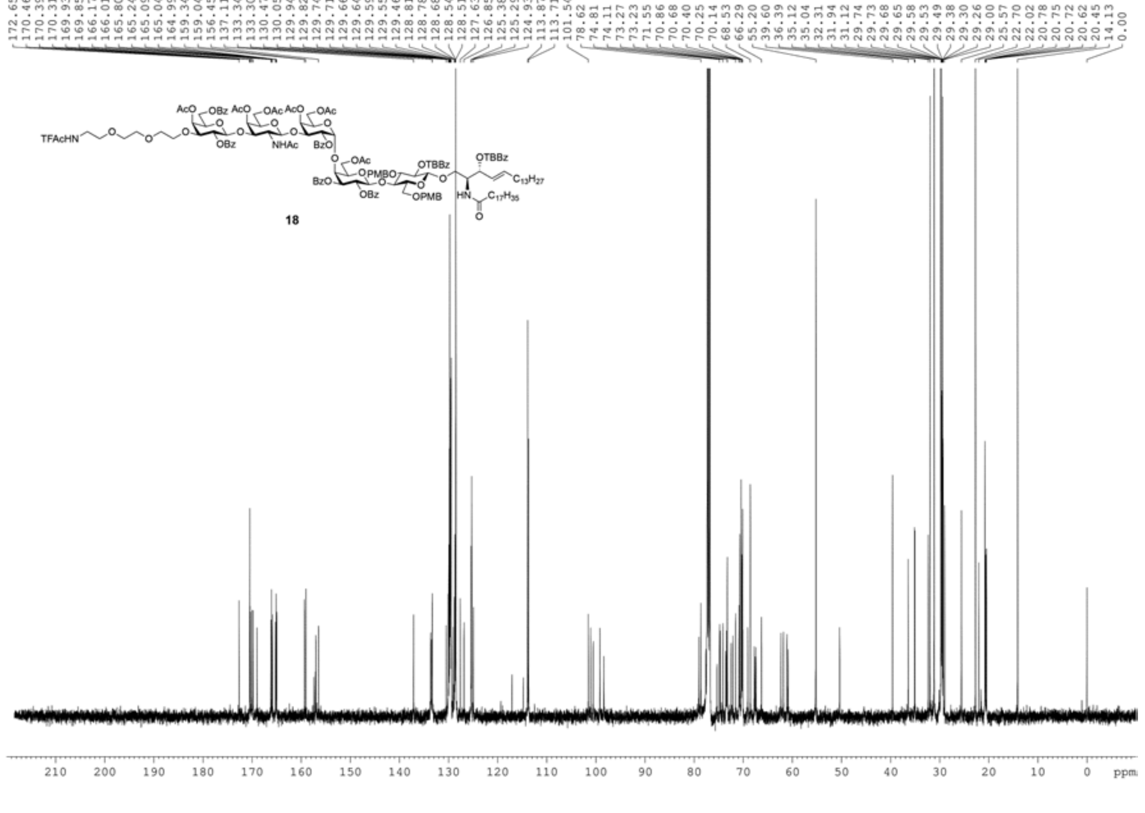

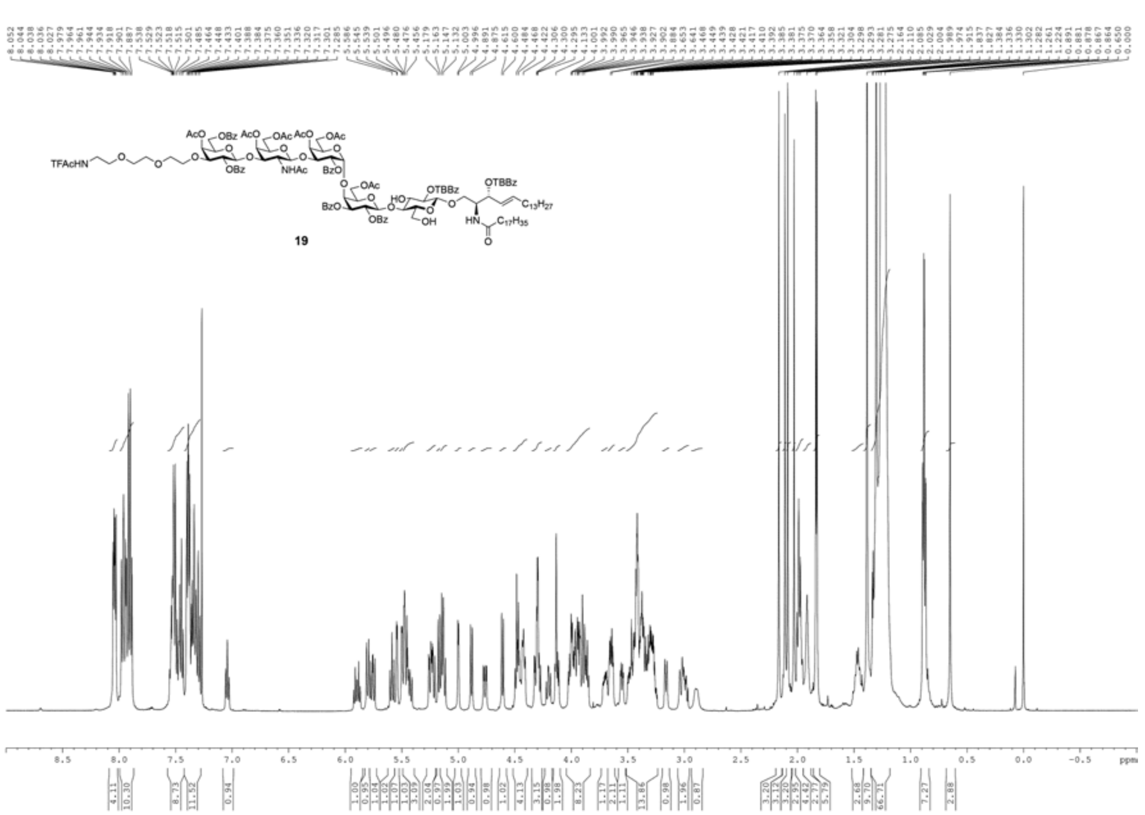
TFAcHN
AcO
OBz
NHAc
BzO
OAc
HO
OTBBz
OH
HN
19
ppm

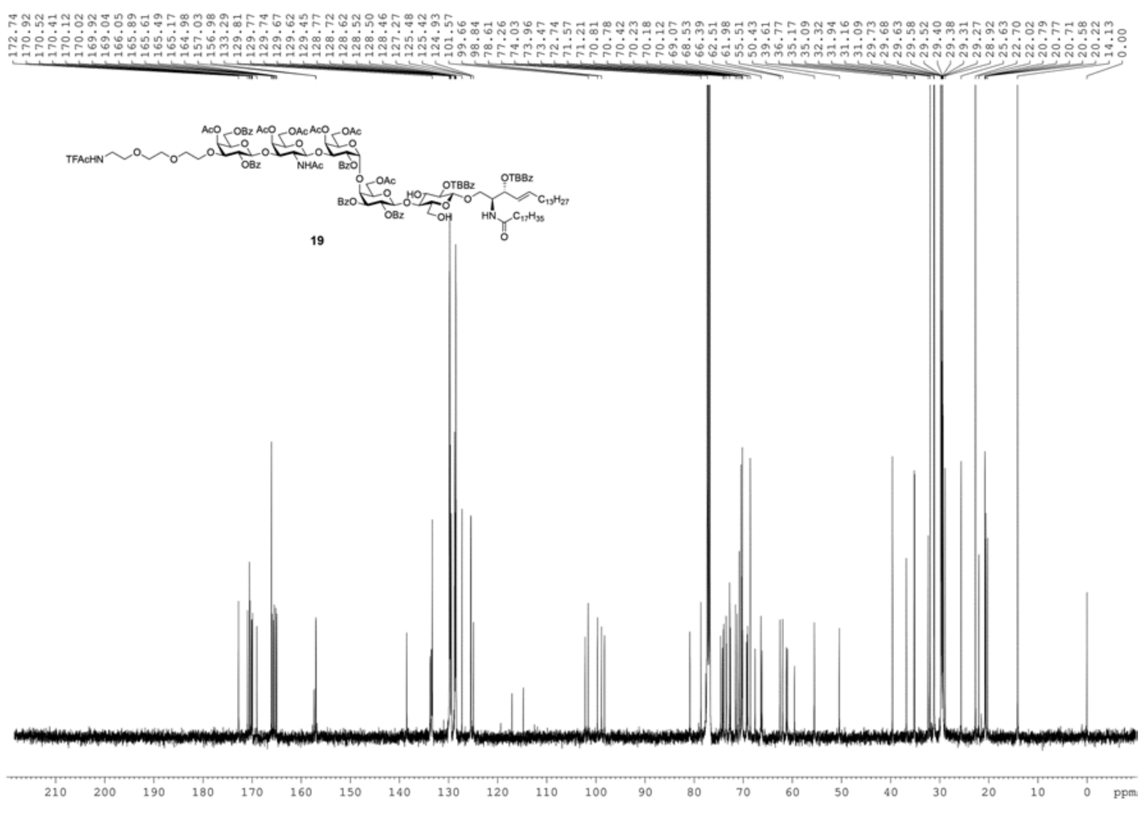
TFAcHN
AcO
OBz
NHAc
BzO
OAc
HO
OTBBz
OH
HN
19
ppm

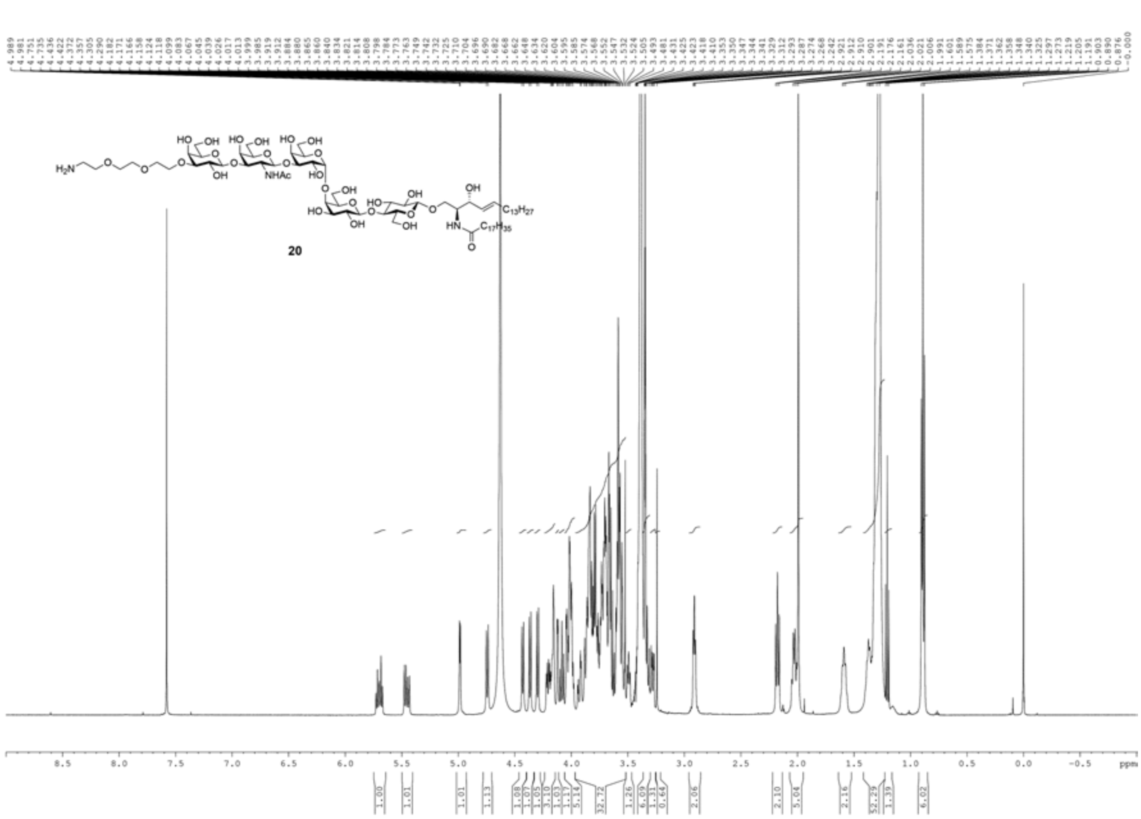
20
ppm

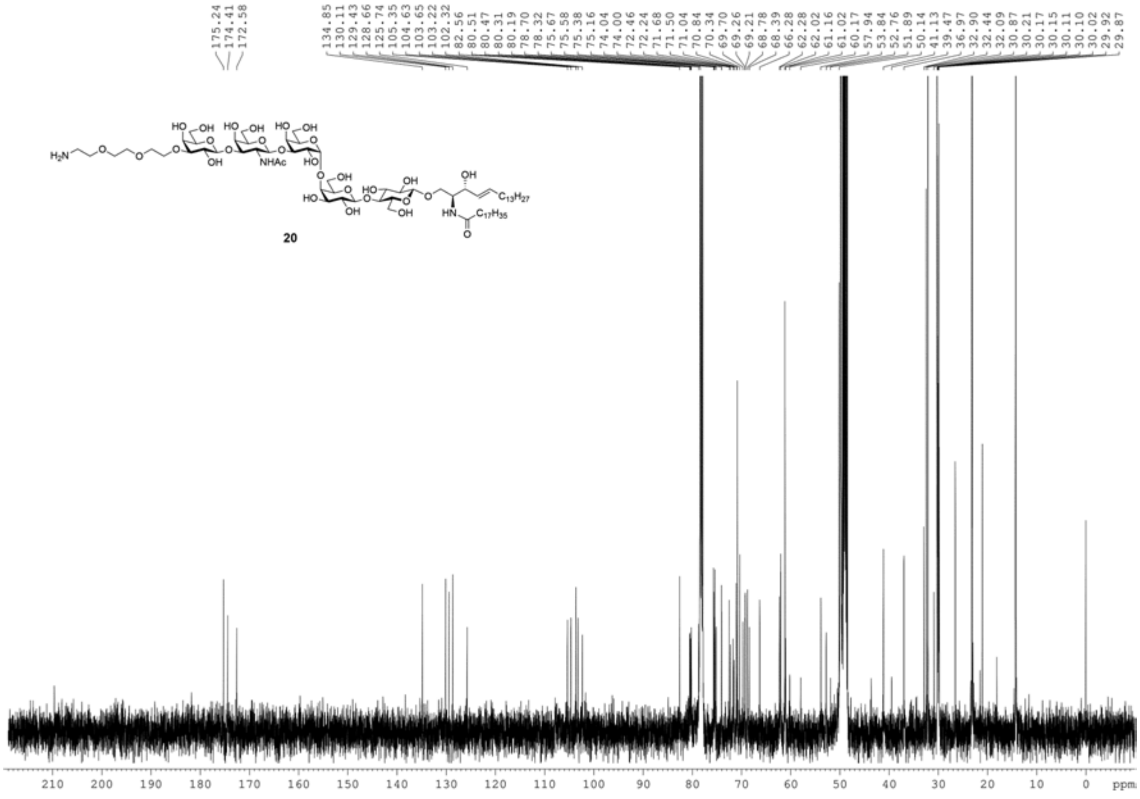
175.24
174.41
172.58
134.85
130.11
129.43
128.66
125.74
105.35
104.63
103.22
102.32
82.56
80.51
80.47
80.31
80.19
78.70
78.32
75.67
75.58
75.38
75.16
74.04
74.00
72.46
72.24
71.68
71.50
71.04
70.84
70.34
69.70
69.26
69.21
68.78
68.39
66.28
62.28
62.02
61.16
61.02
60.17
57.94
53.84
52.76
51.89
50.14
41.13
39.47
36.97
32.90
32.44
32.09
30.87
30.21
30.17
30.15
30.11
30.10
30.02
29.92
29.87
20
ppm

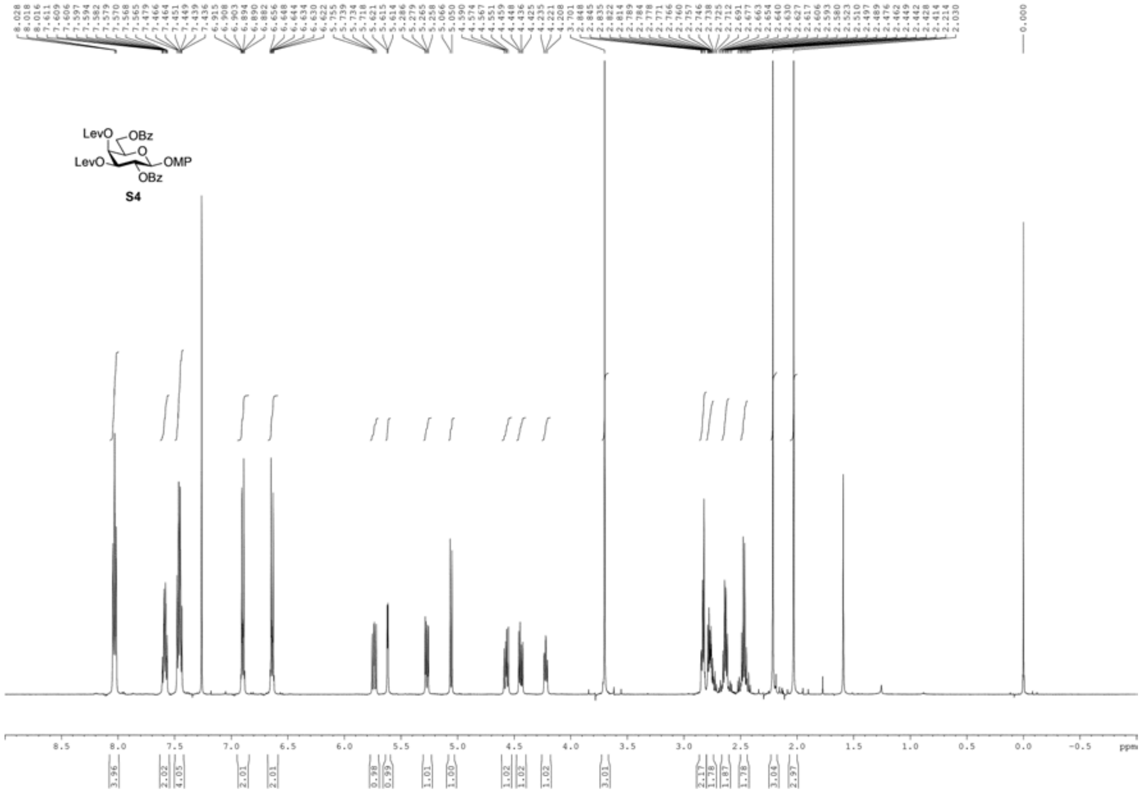

LevO
OBz
LevO
O
OMP
OBz
S4
ppm

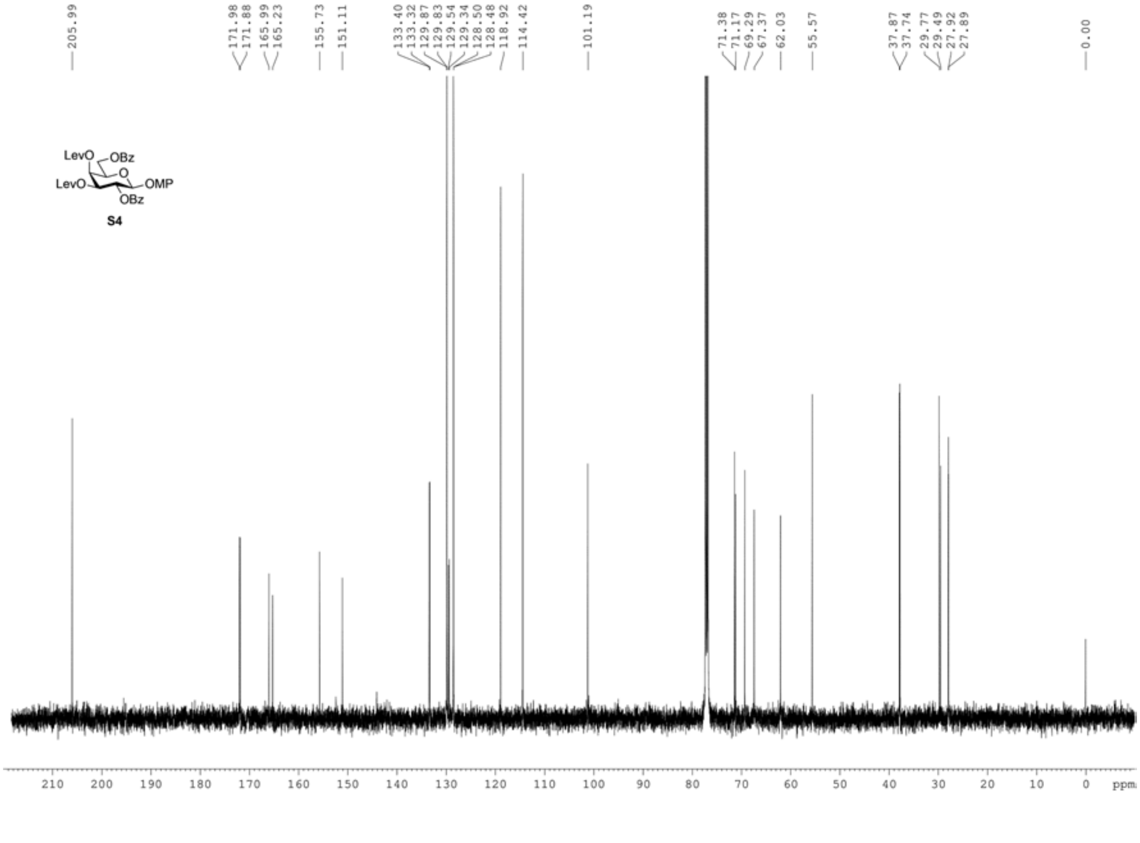

205.99
171.98
171.88
165.99
165.23
155.73
151.11
133.40
133.32
129.87
129.83
129.54
129.34
128.50
128.48
118.92
114.42
101.19
71.38
71.17
69.29
67.37
62.03
55.57
37.87
37.74
29.77
29.49
27.92
27.89
0.00
LevO
OBz
LevO
O
OMP
OBz
S4
ppm

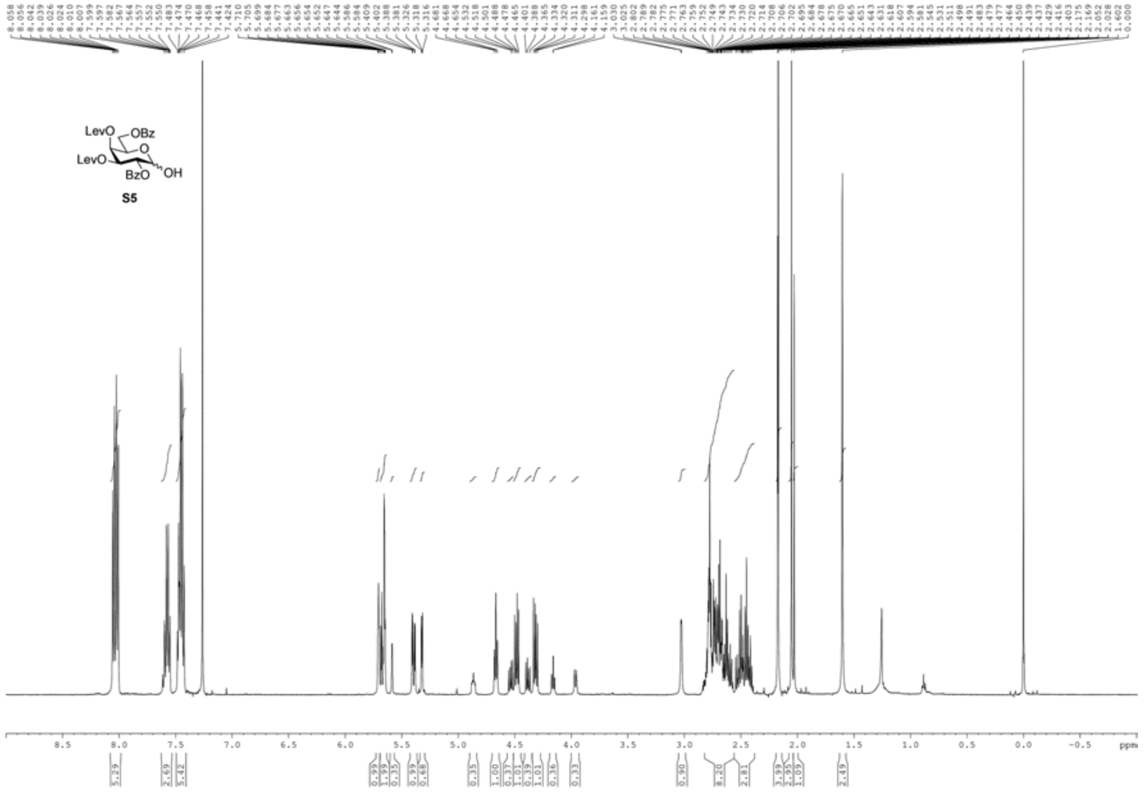
LevO
OBz
LevO
BzO
OH
S5
ppm

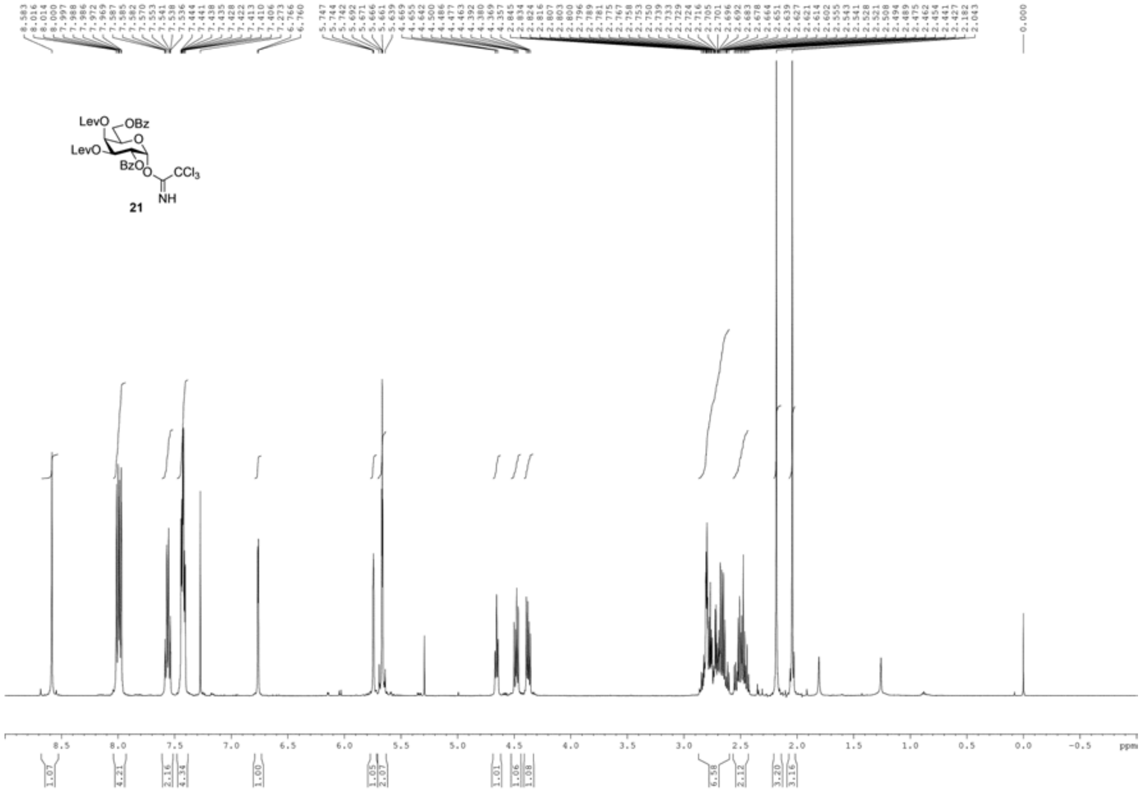
8.583 8.016 8.014 8.000 7.997 7.988 7.986 7.972 7.969 7.587 7.585 7.582 7.570 7.553 7.541 7.538 7.536 7.444 7.441 7.438 7.435 7.428 7.422 7.413 7.410 7.406 7.273 6.766 6.760
5.747 5.744 5.742 5.692 5.671 5.666 5.661 5.639 4.669 4.655 4.642 4.500 4.486 4.477 4.463 4.392 4.380 4.369 4.357 2.845 2.834 2.824 2.816 2.807 2.803 2.800 2.796 2.789 2.781 2.775 2.767 2.758 2.753 2.750 2.739 2.733 2.729 2.724 2.716 2.705 2.701 2.696 2.692 2.683 2.678 2.664 2.651 2.639 2.627 2.621 2.614 2.602 2.555 2.543 2.541 2.528 2.521 2.508 2.494 2.489 2.475 2.462 2.454 2.441 2.427 2.182 2.043
0.000
LevO
OBz
O
LevO
BzO
O
CCl3
NH
21
8.5 8.0 7.5 7.0 6.5 6.0 5.5 5.0 4.5 4.0 3.5 3.0 2.5 2.0 1.5 1.0 0.5 0.0 -0.5 ppm
1.07 4.21 2.16 4.34 1.00 1.05 2.07 1.01 1.06 1.08 6.58 2.12 3.20 3.16

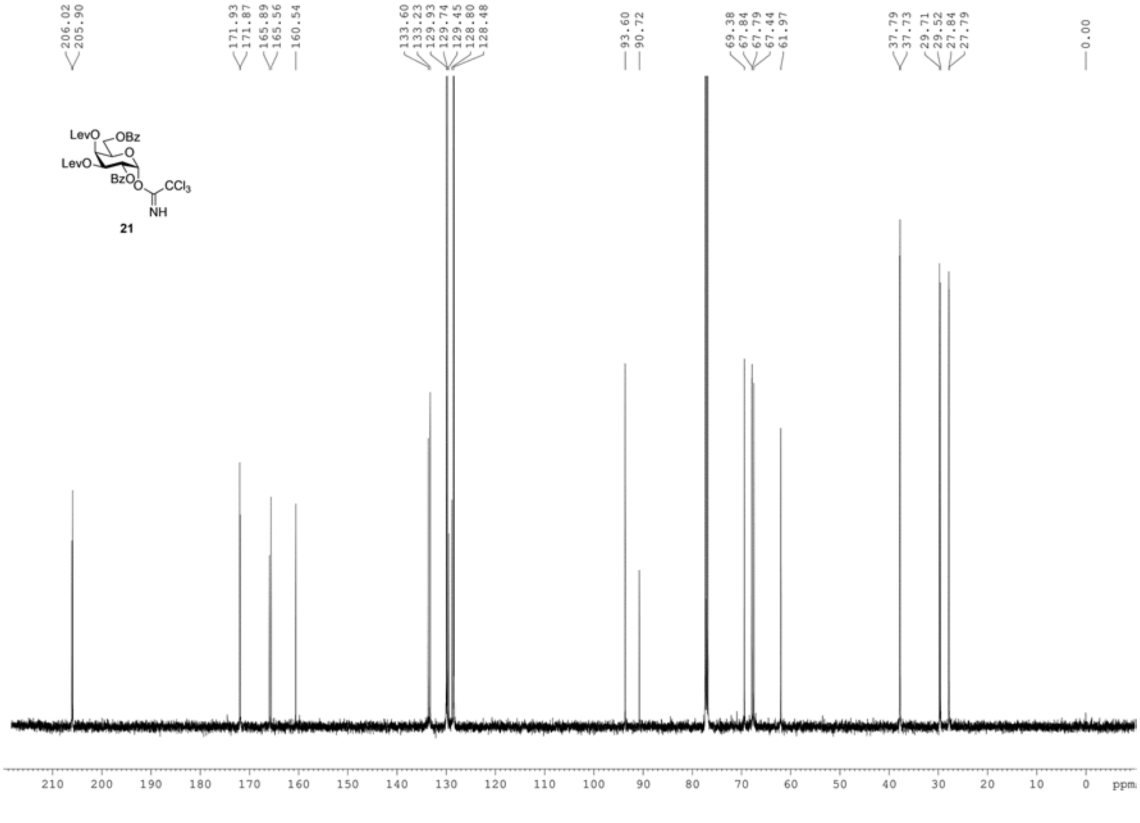
206.02 205.90
171.93 171.87 165.89 165.56 160.54
133.60 133.23 129.93 129.74 129.45 128.80 128.48
93.60 90.72
69.38 67.84 67.79 67.44 61.97
37.79 37.73 29.71 29.52 27.84 27.79
0.00
LevO
OBz
O
LevO
BzO
O
CCl3
NH
21
210 200 190 180 170 160 150 140 130 120 110 100 90 80 70 60 50 40 30 20 10 0 ppm

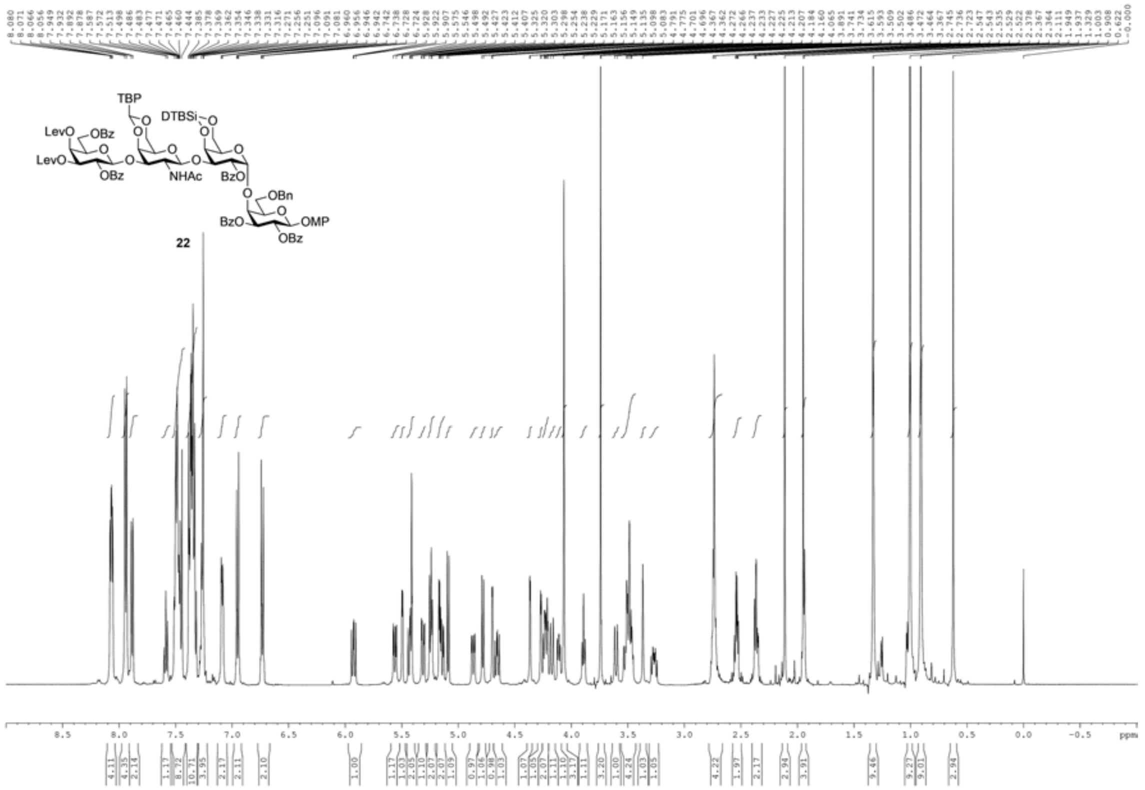

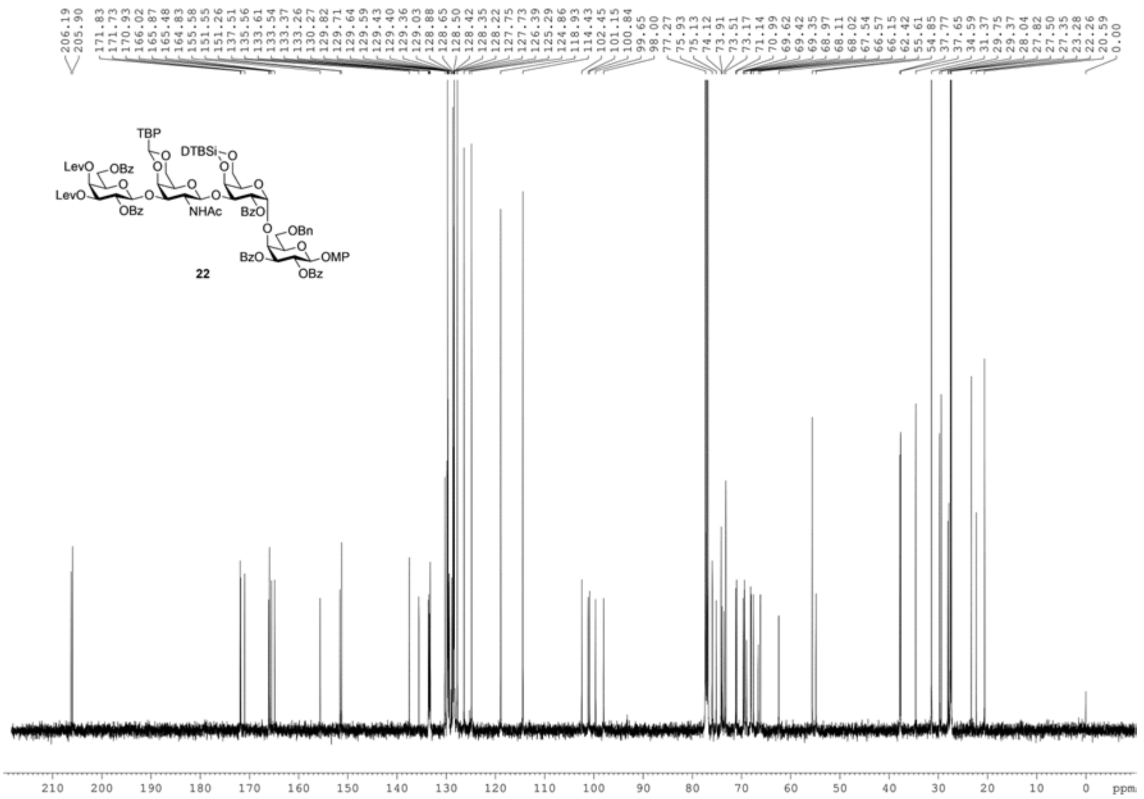

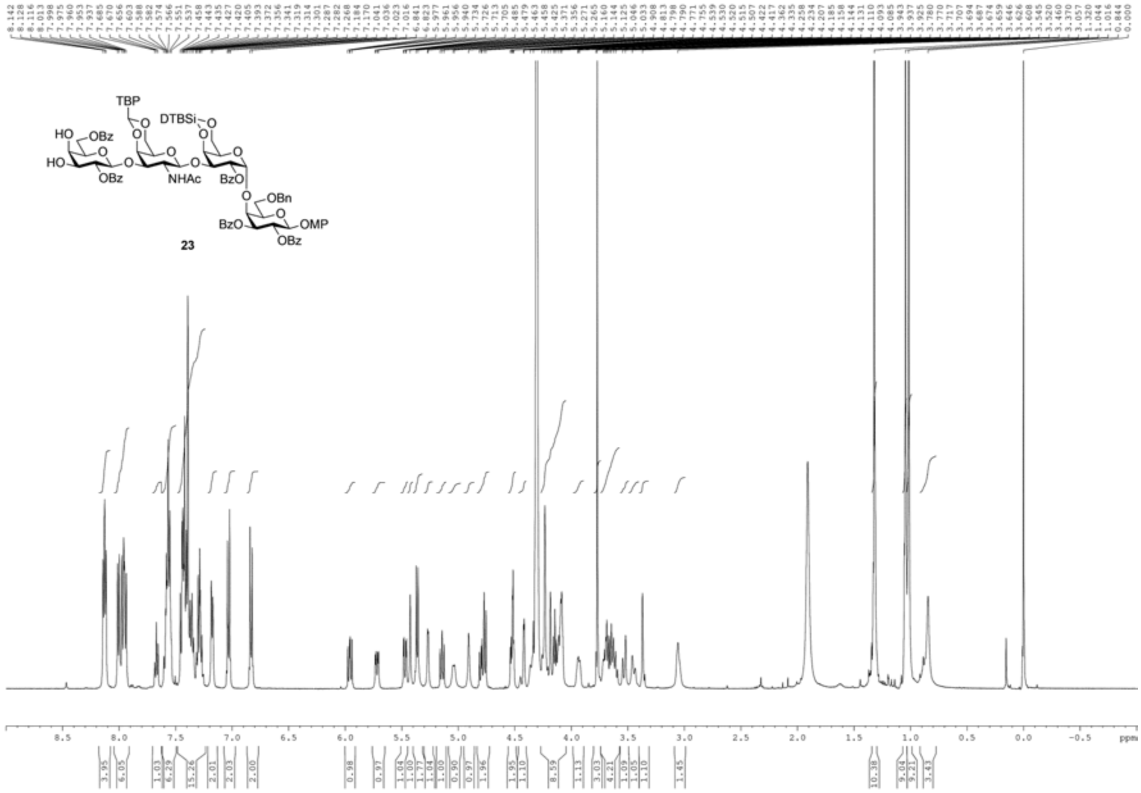
TBP
DTBSi
HO
OBz
NHAc
BzO
OBn
OMP
23
ppm

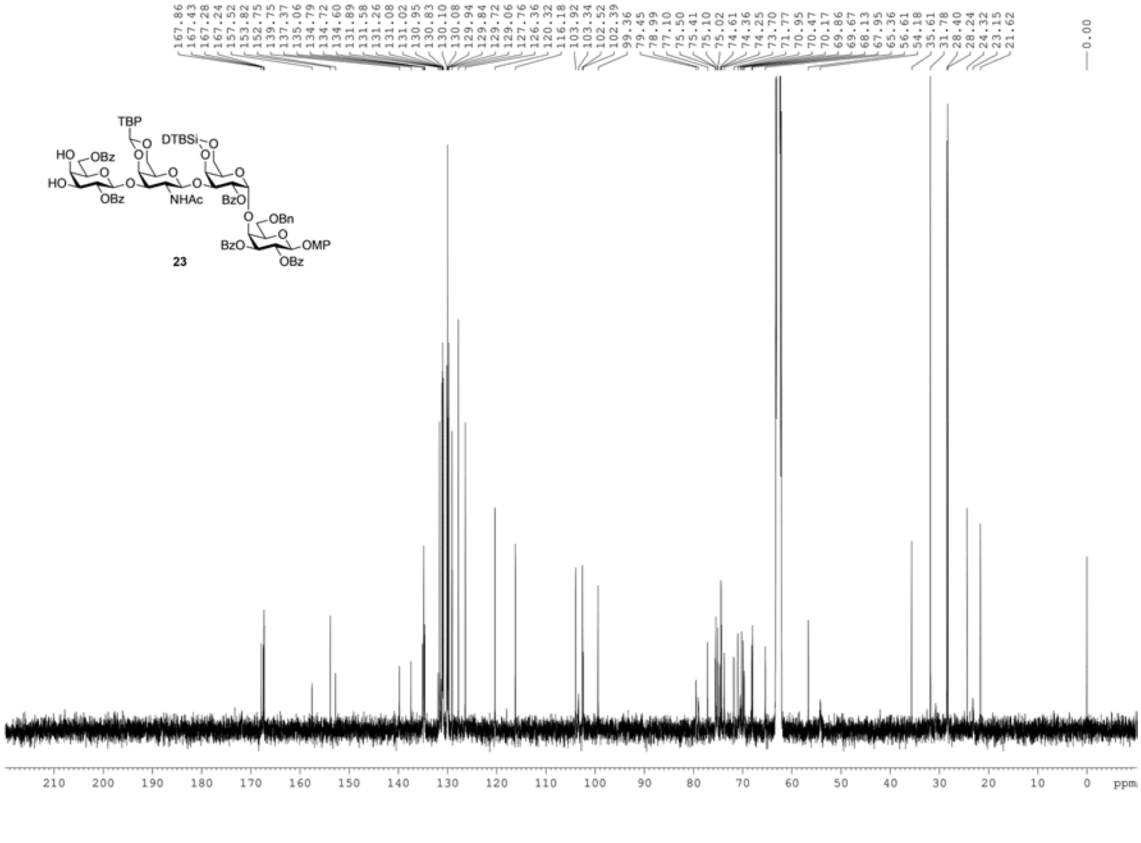
TBP
DTBSi
HO
OBz
NHAc
BzO
OBn
OMP
23
ppm

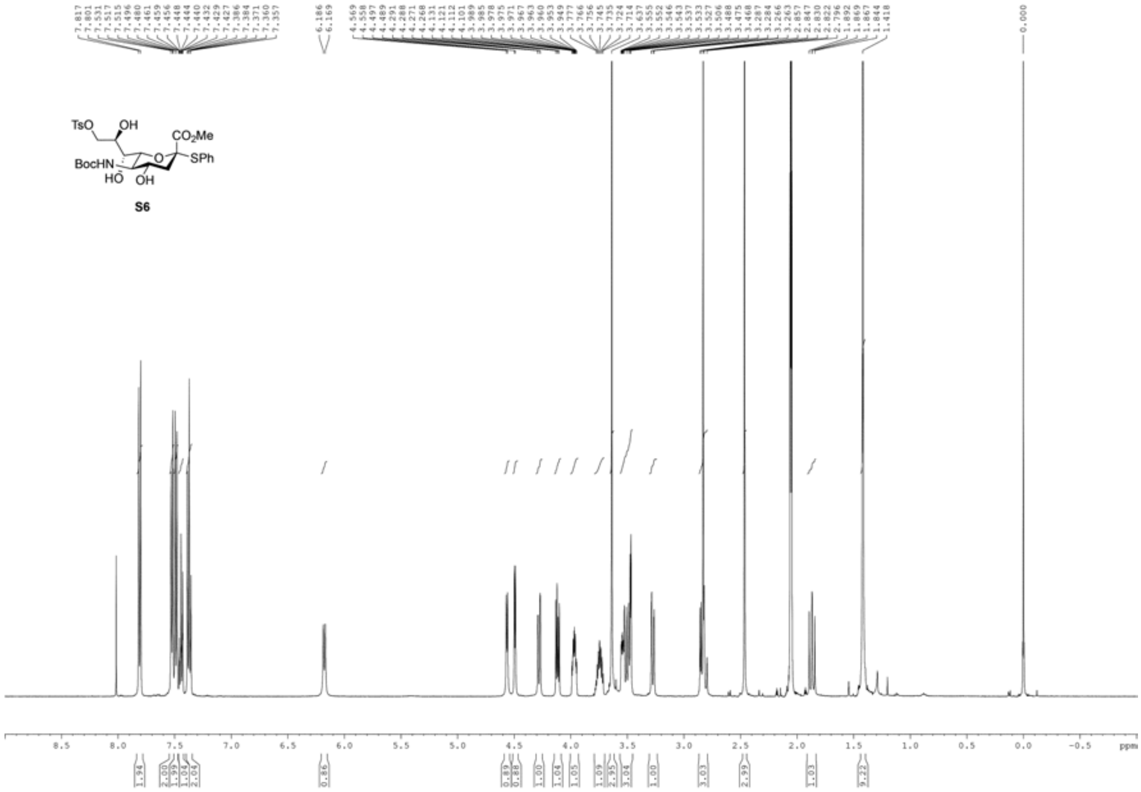

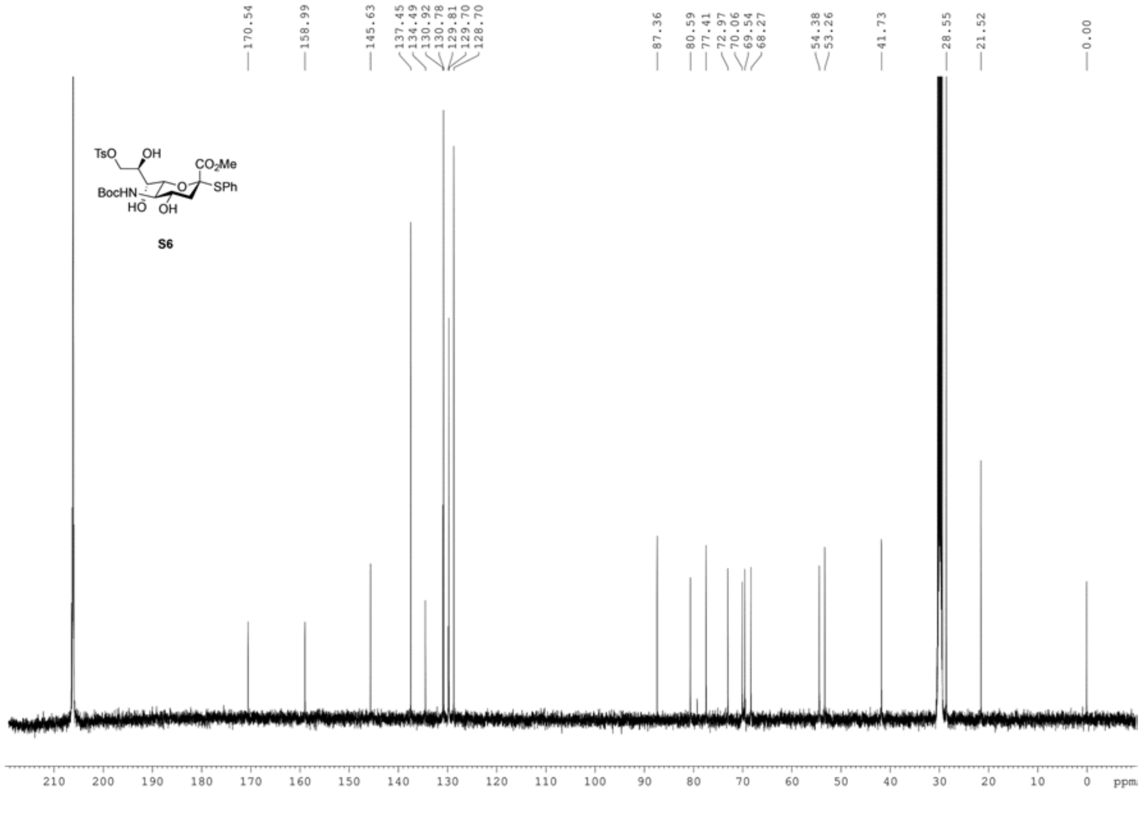

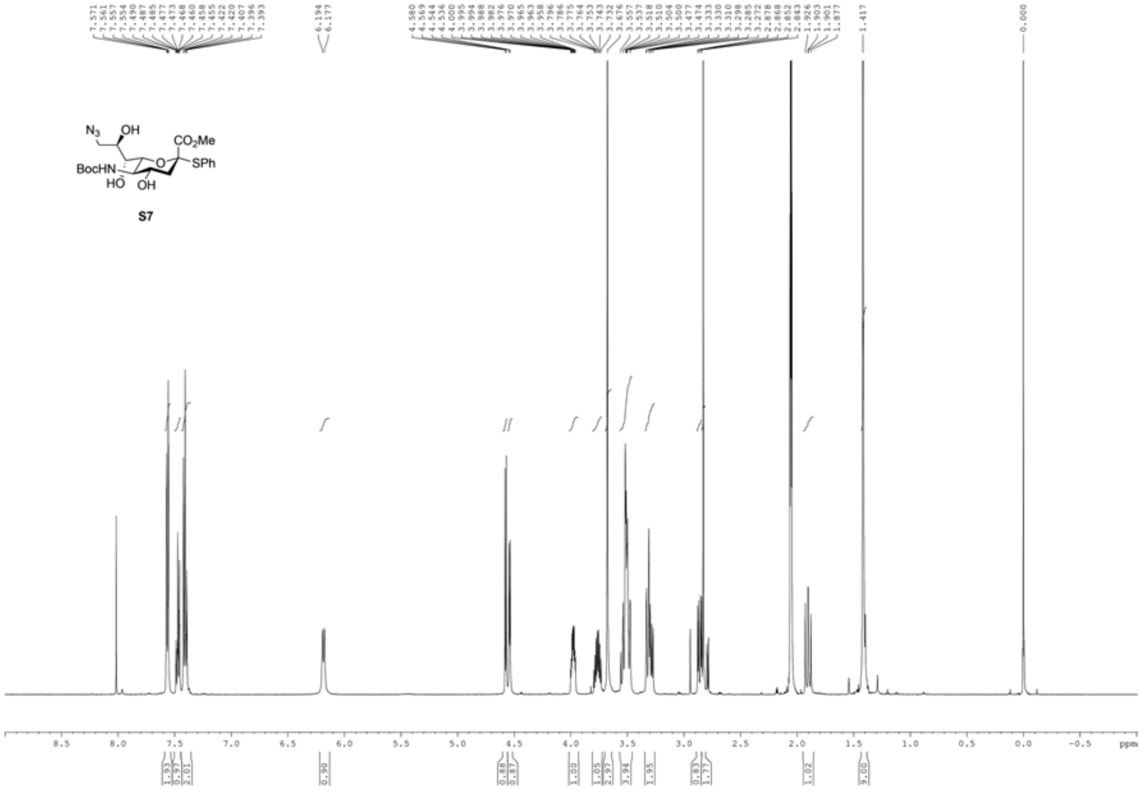

S7

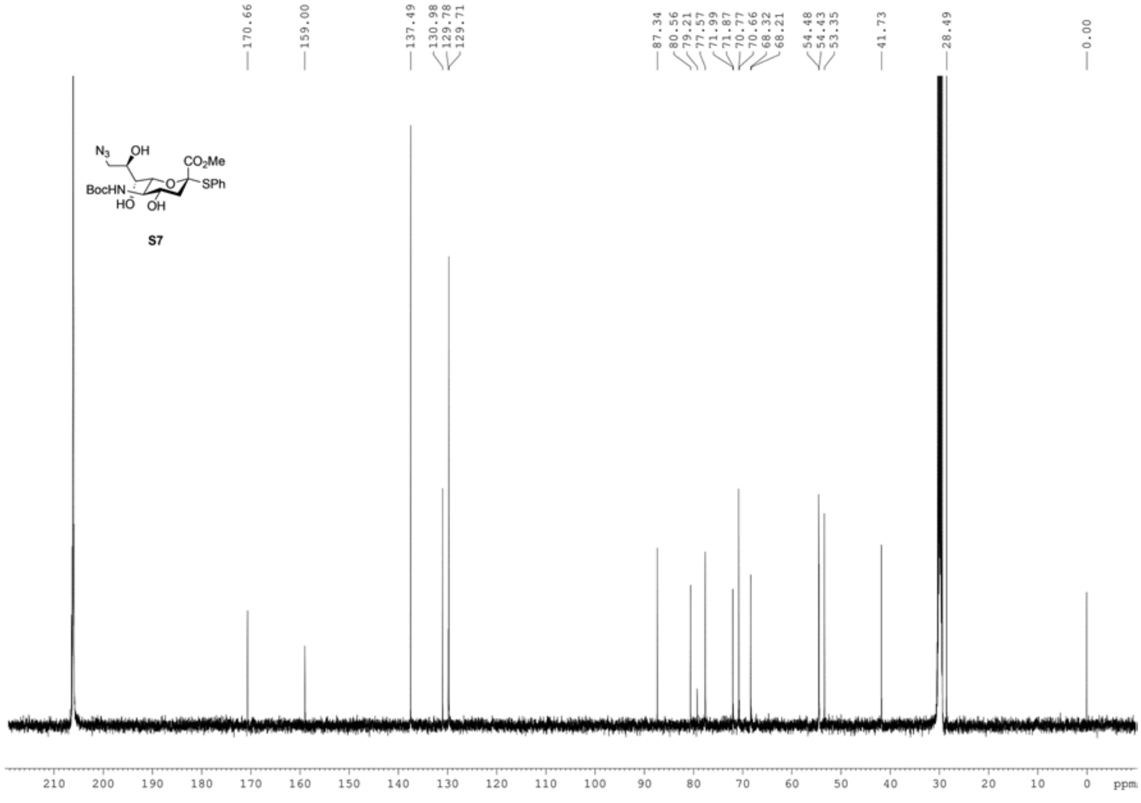

170.66
159.00
137.49
130.98
129.78
129.71
87.34
80.56
79.21
77.57
71.99
71.87
70.77
70.66
68.32
68.21
54.48
54.43
53.35
41.73
28.49
0.00
S7

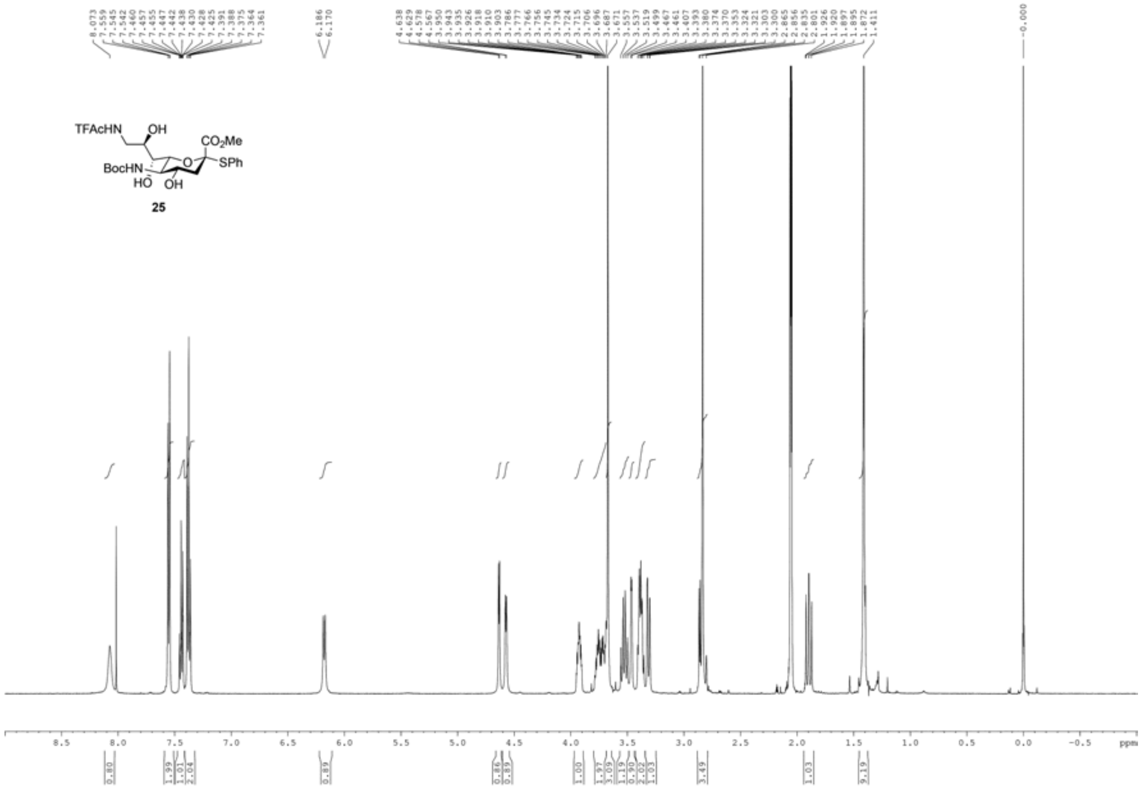

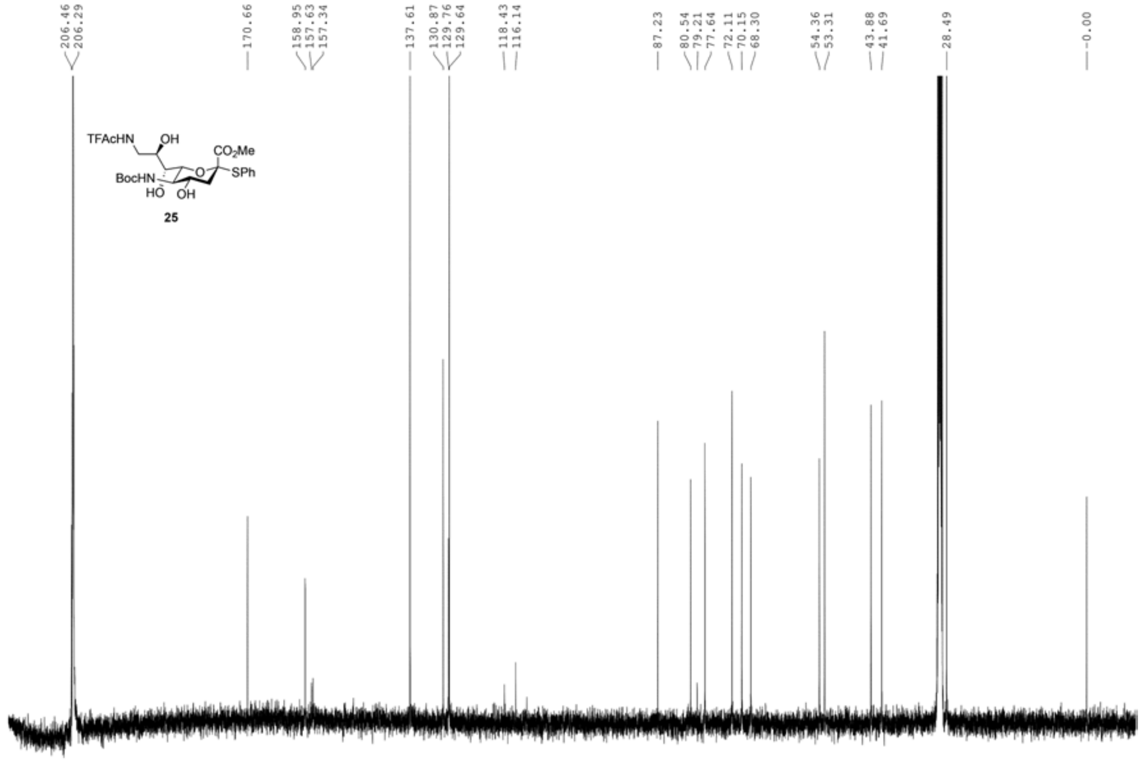

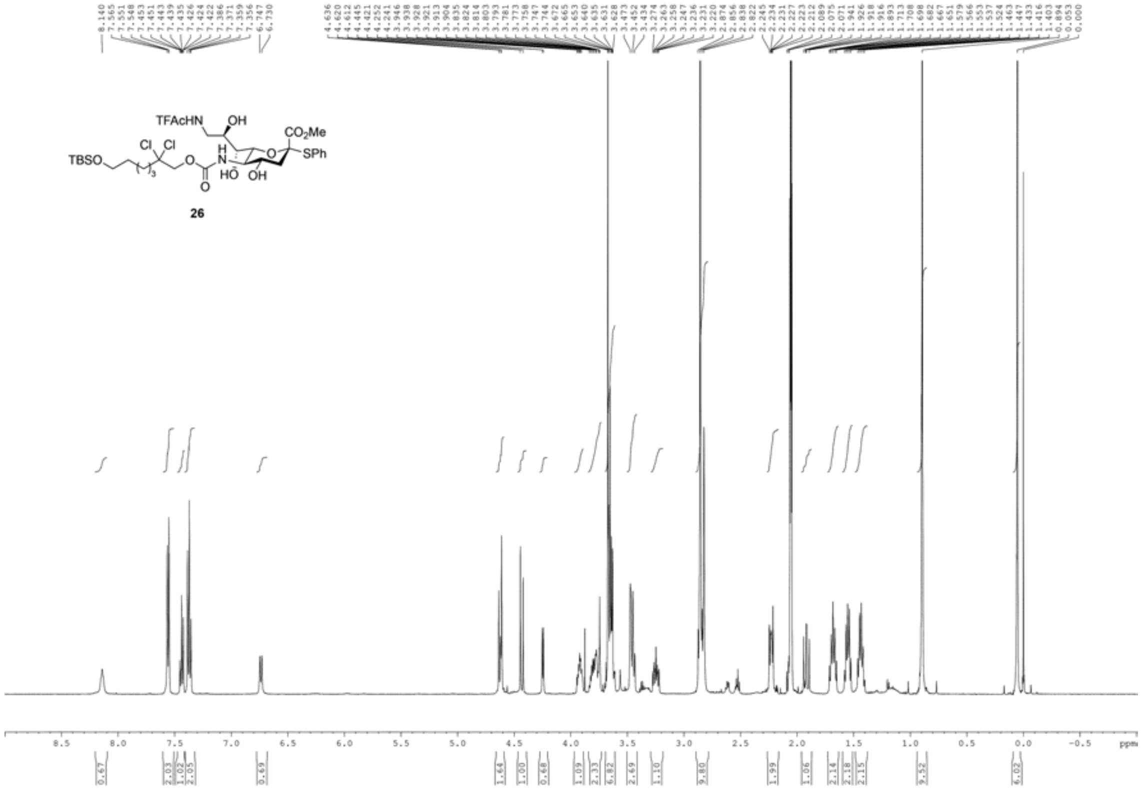
TFAcHN
OH
CO2Me
Cl
Cl
TBSO
H
N
O
O
SPh
HO
OH
3
O
26
ppm

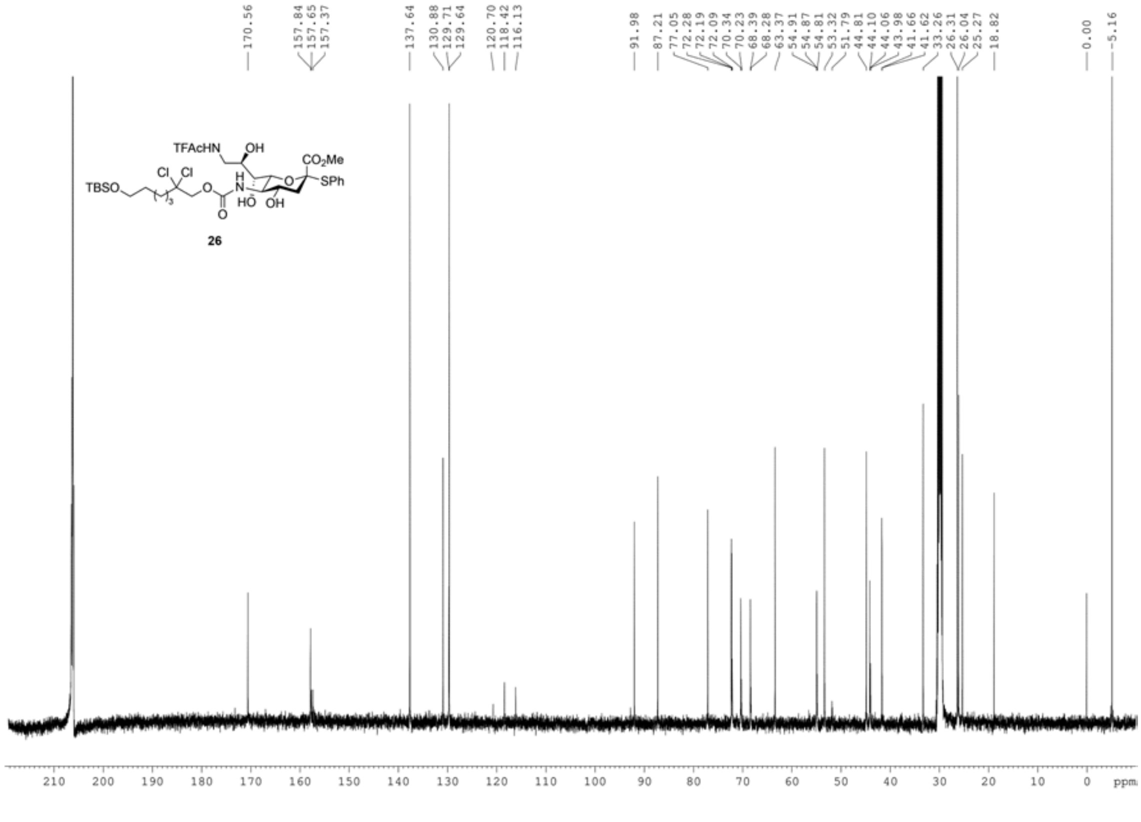
170.56
157.84
157.65
157.37
137.64
130.88
129.71
129.64
120.70
118.42
116.13
91.98
87.21
77.05
72.28
72.19
72.09
70.34
70.23
68.39
68.28
63.37
54.91
54.87
54.81
53.32
51.79
44.81
44.10
44.06
43.98
41.66
41.62
33.26
26.31
26.04
25.27
18.82
0.00
-5.16
TFAcHN
OH
CO2Me
Cl
Cl
TBSO
H
N
O
O
SPh
HO
OH
3
O
26
ppm

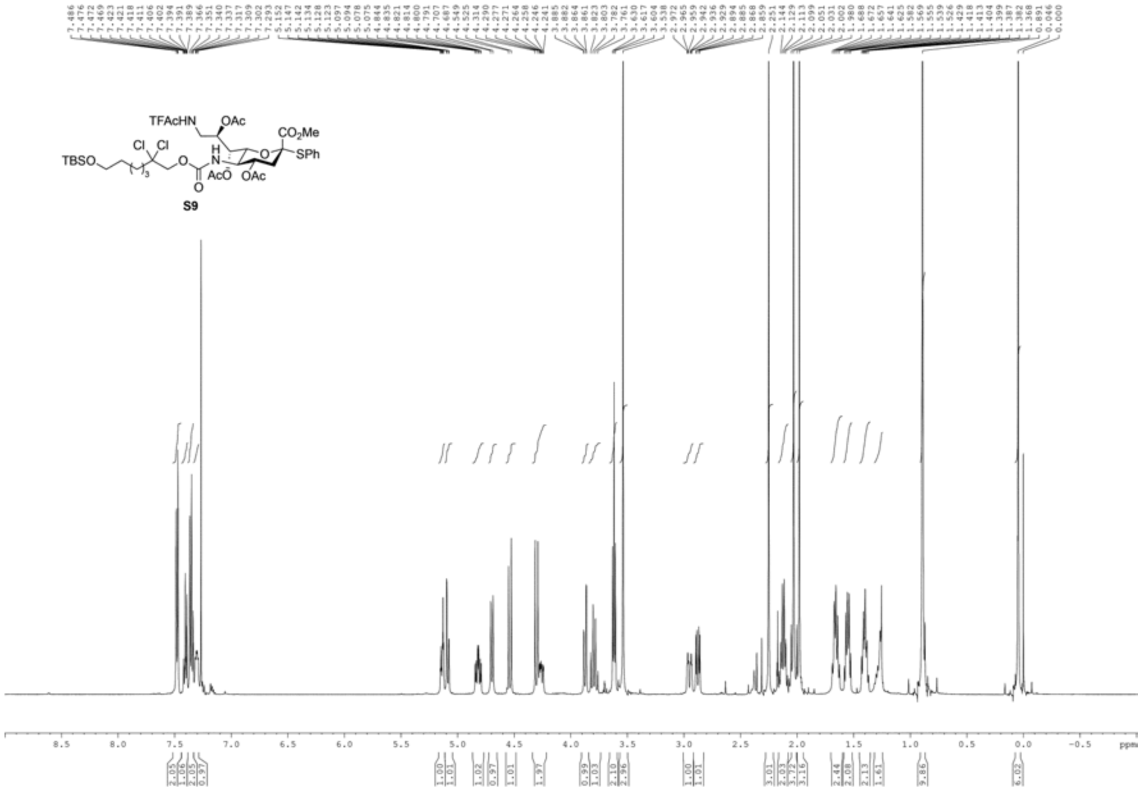

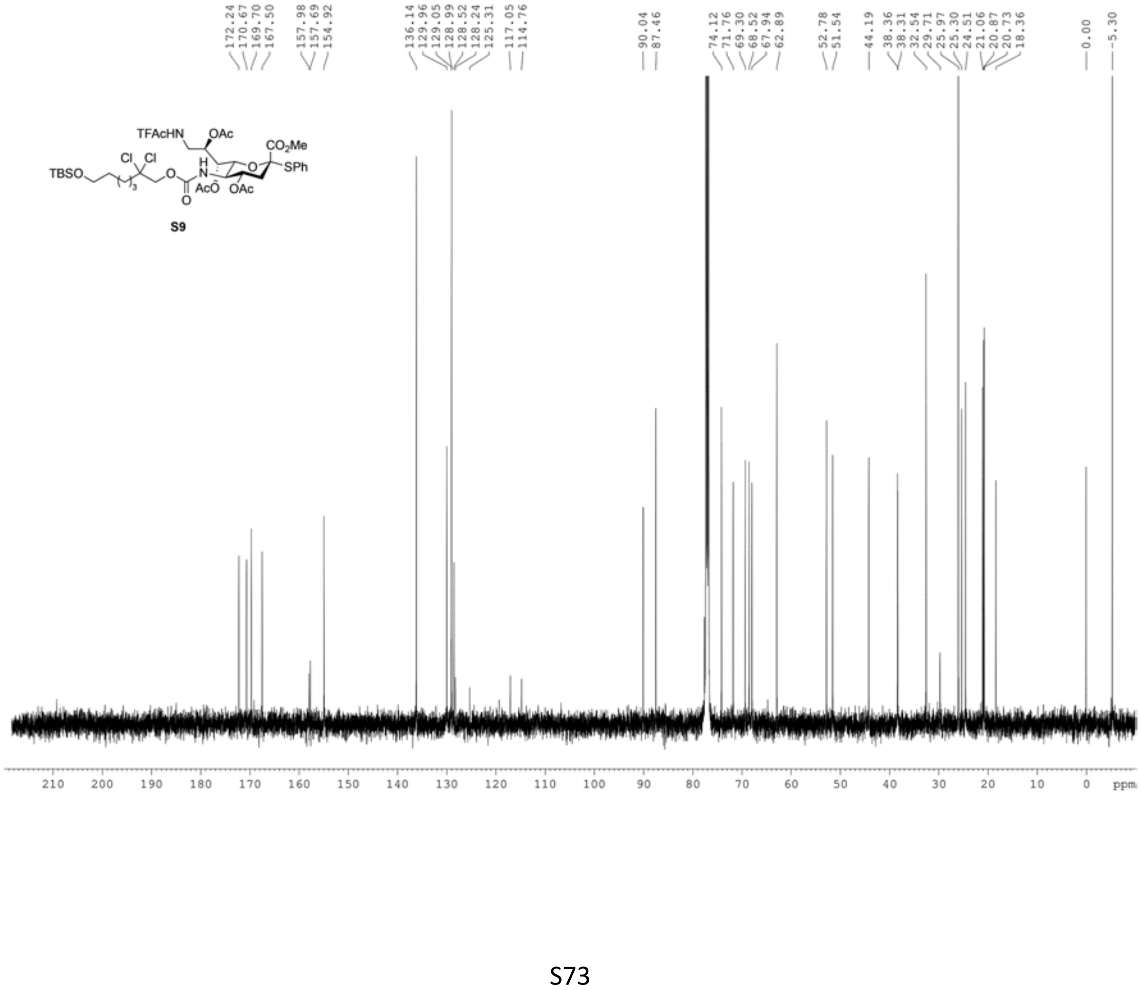

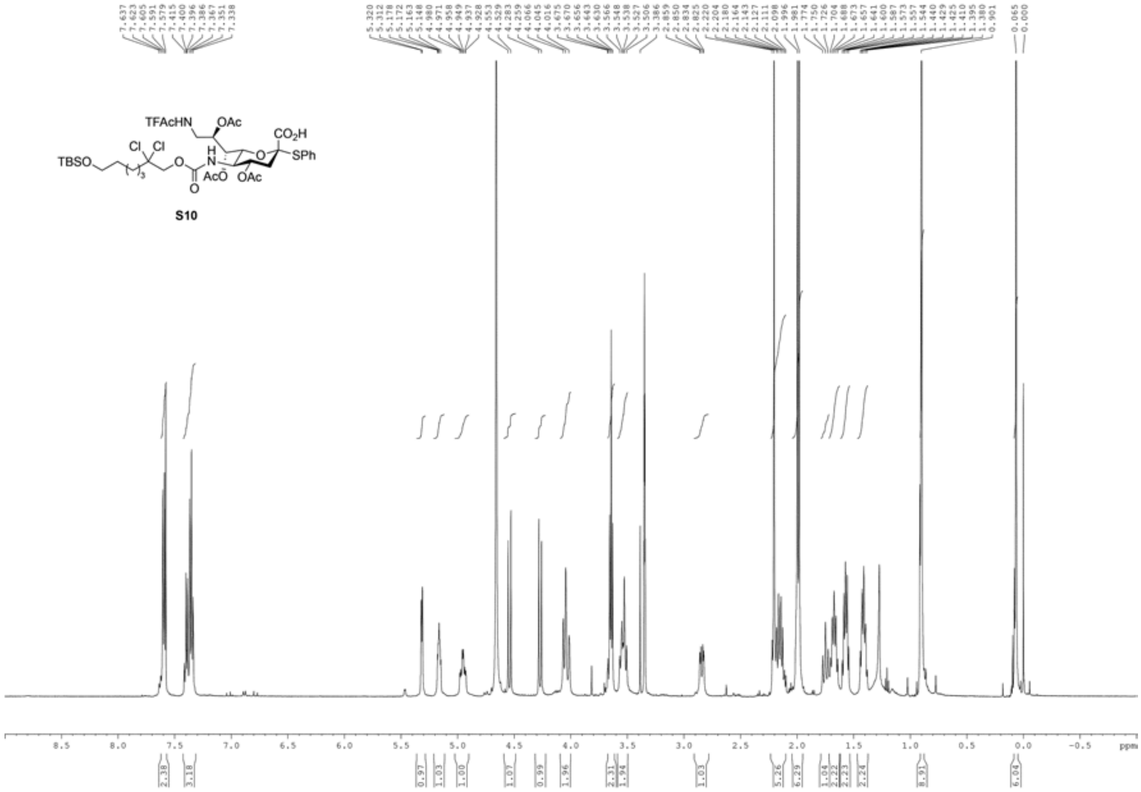
TFAcHN
OAc
CO2H
SPh
TBSO
Cl
Cl
3
O
H
N
O
AcO
OAc
S10
ppm

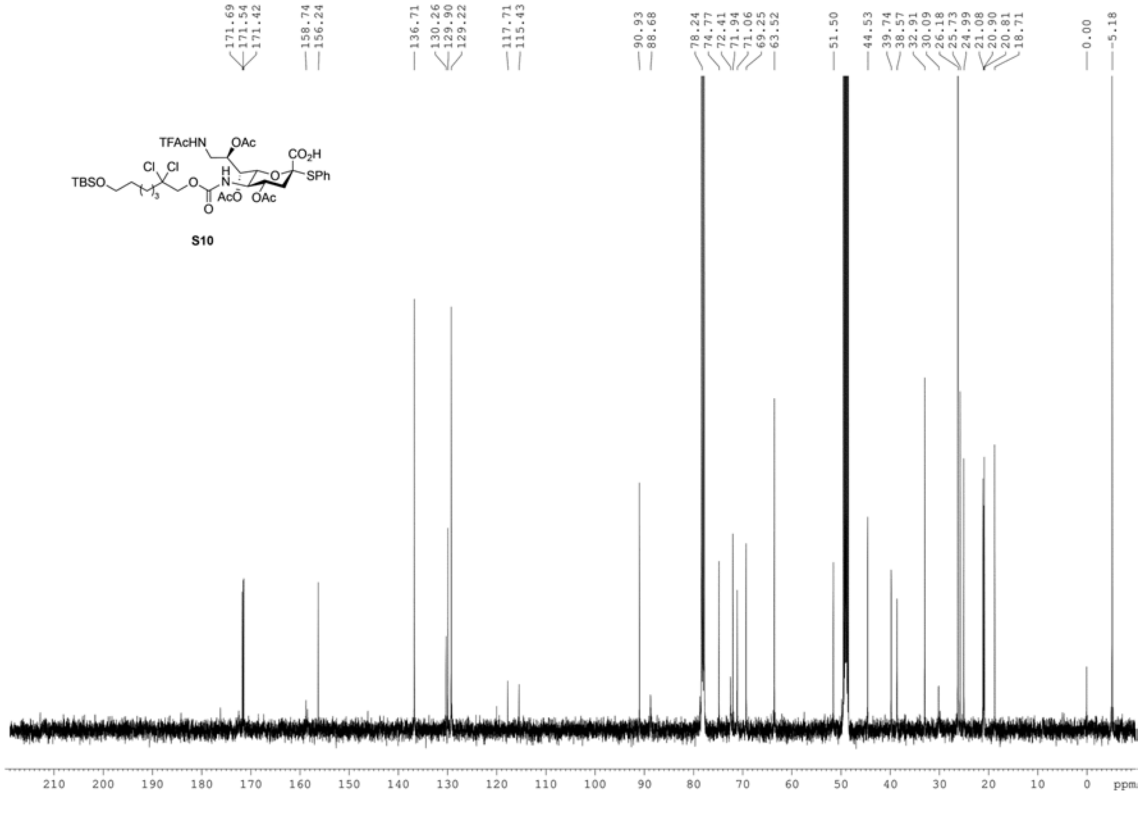
171.69
171.54
171.42
158.74
156.24
136.71
130.26
129.90
129.22
117.71
115.43
90.93
88.68
78.24
74.77
72.41
71.94
71.06
69.25
63.52
51.50
44.53
39.74
38.57
32.91
30.09
26.18
25.73
24.99
21.08
20.90
20.81
18.71
0.00
-5.18
TFAcHN
OAc
CO2H
SPh
TBSO
Cl
Cl
3
O
H
N
O
AcO
OAc
S10
ppm

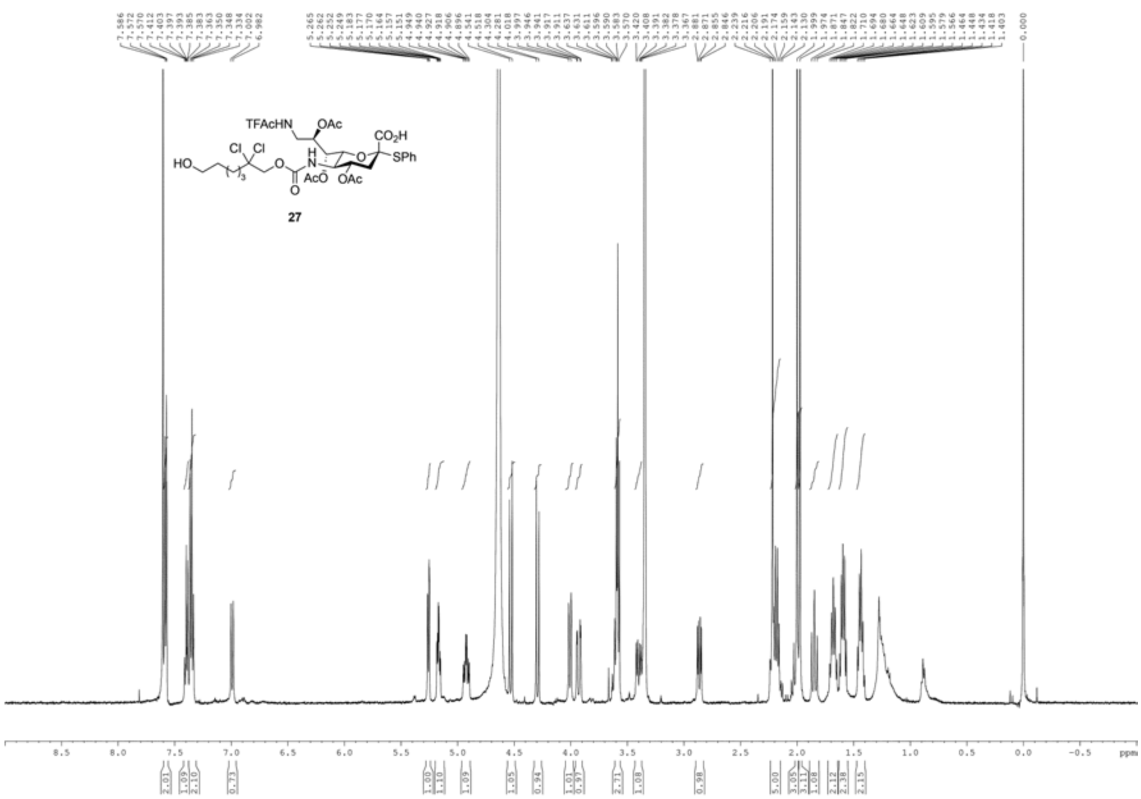
TFAcHN
OAc
CO2H
Cl
Cl
HO
SPh
AcO
OAc
27
ppm

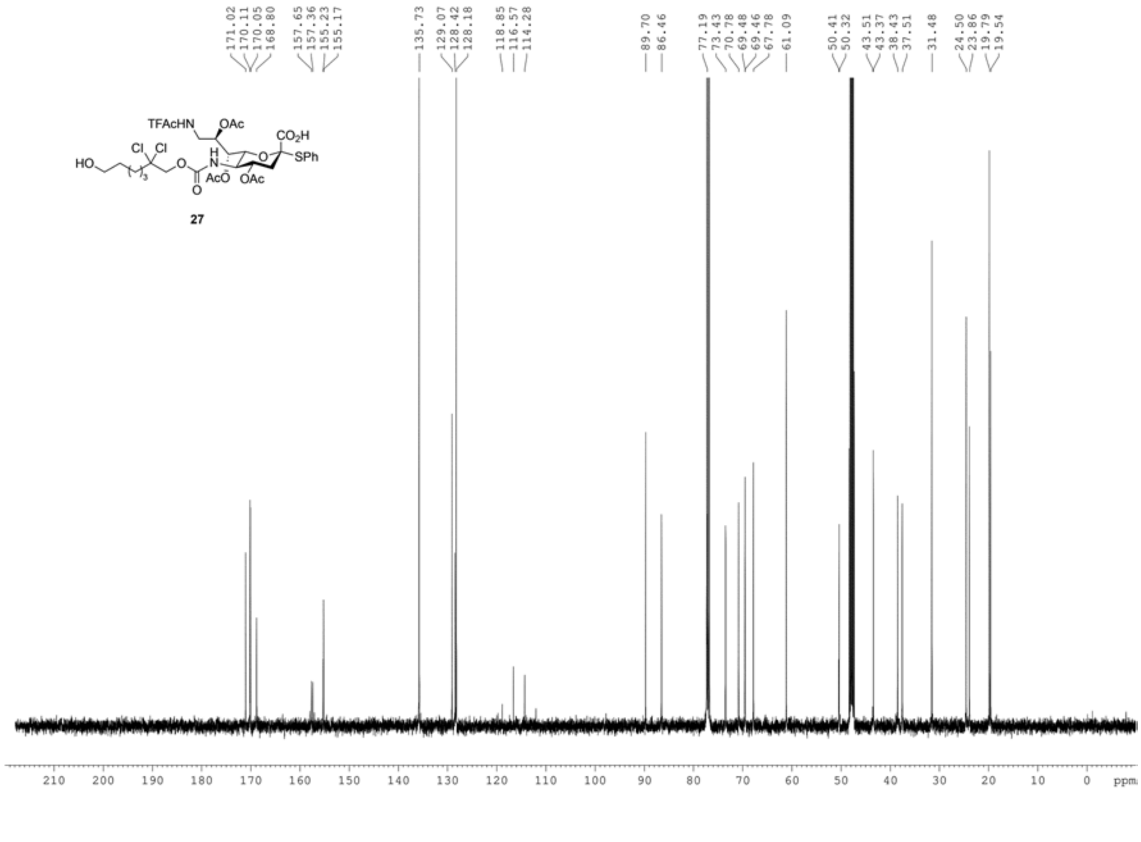
171.02
170.11
170.05
168.80
157.65
157.36
155.23
155.17
135.73
129.07
128.42
128.18
118.85
116.57
114.28
89.70
86.46
77.19
73.43
70.78
69.48
69.46
67.78
61.09
50.41
50.32
43.51
43.37
38.43
37.51
31.48
24.50
23.86
19.79
19.54
TFAcHN
OAc
CO2H
Cl
Cl
HO
SPh
AcO
OAc
27
ppm

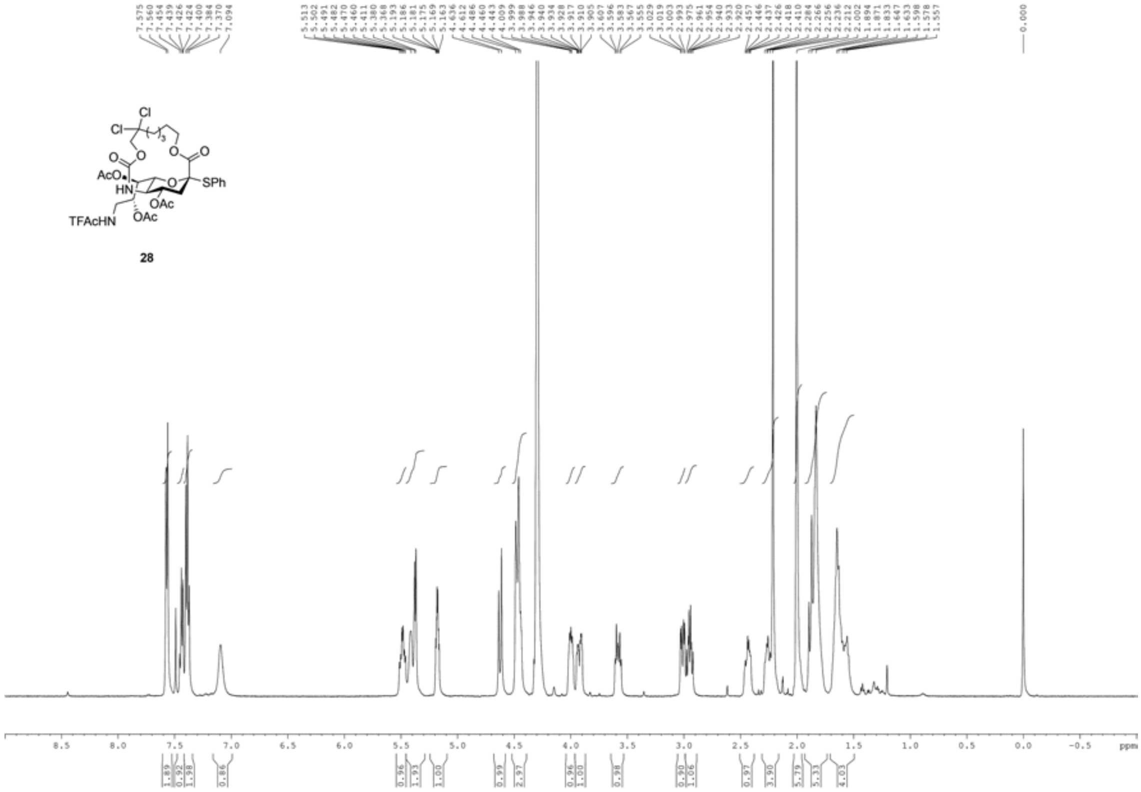

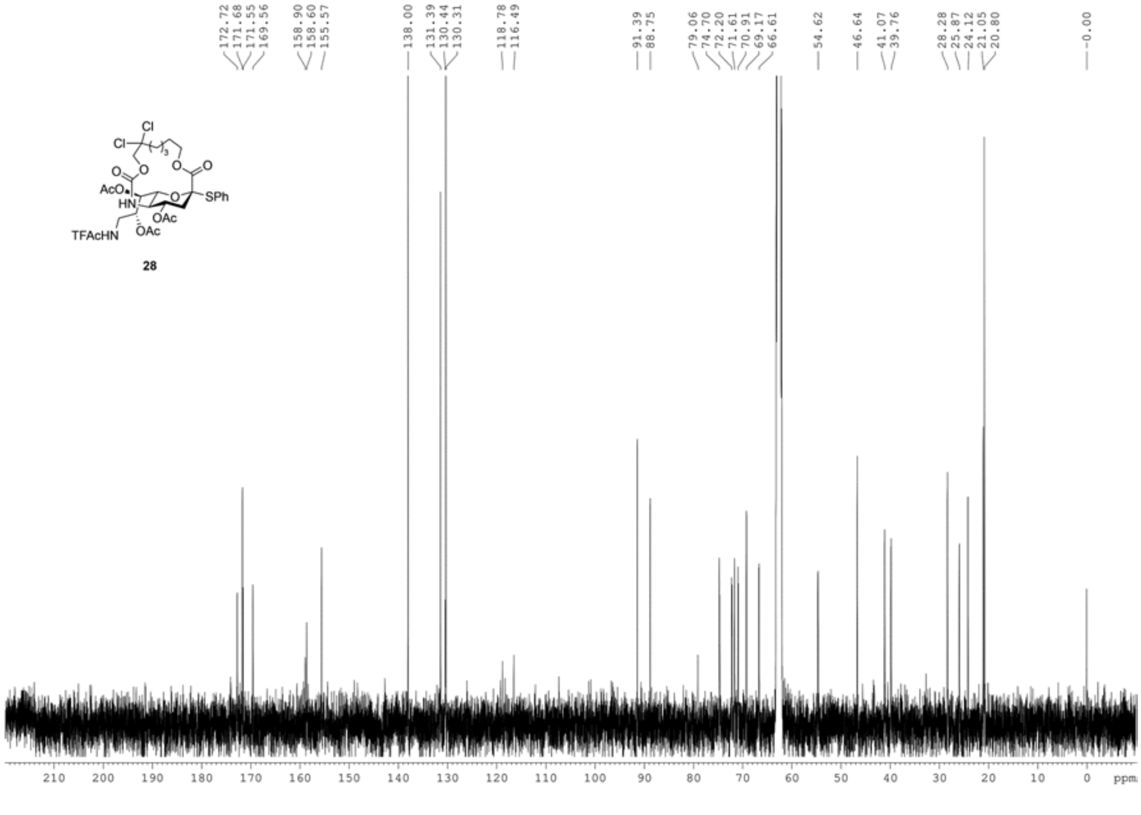

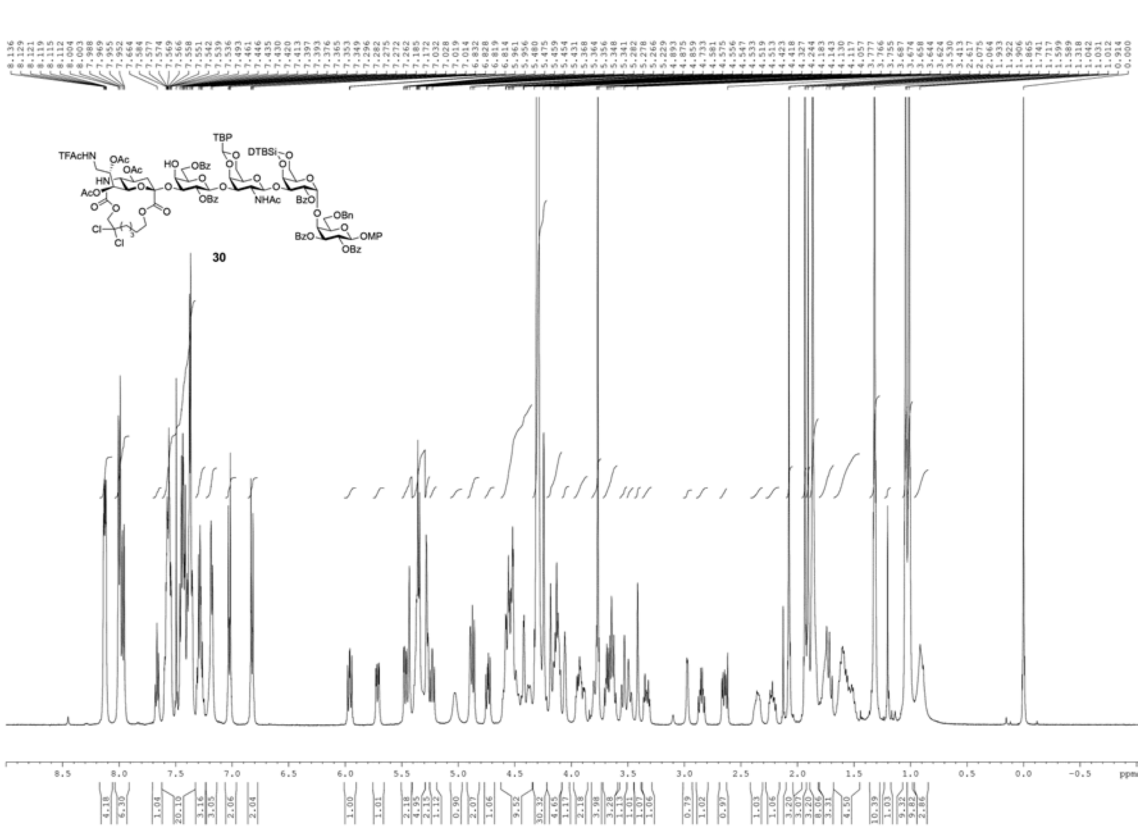
30
ppm

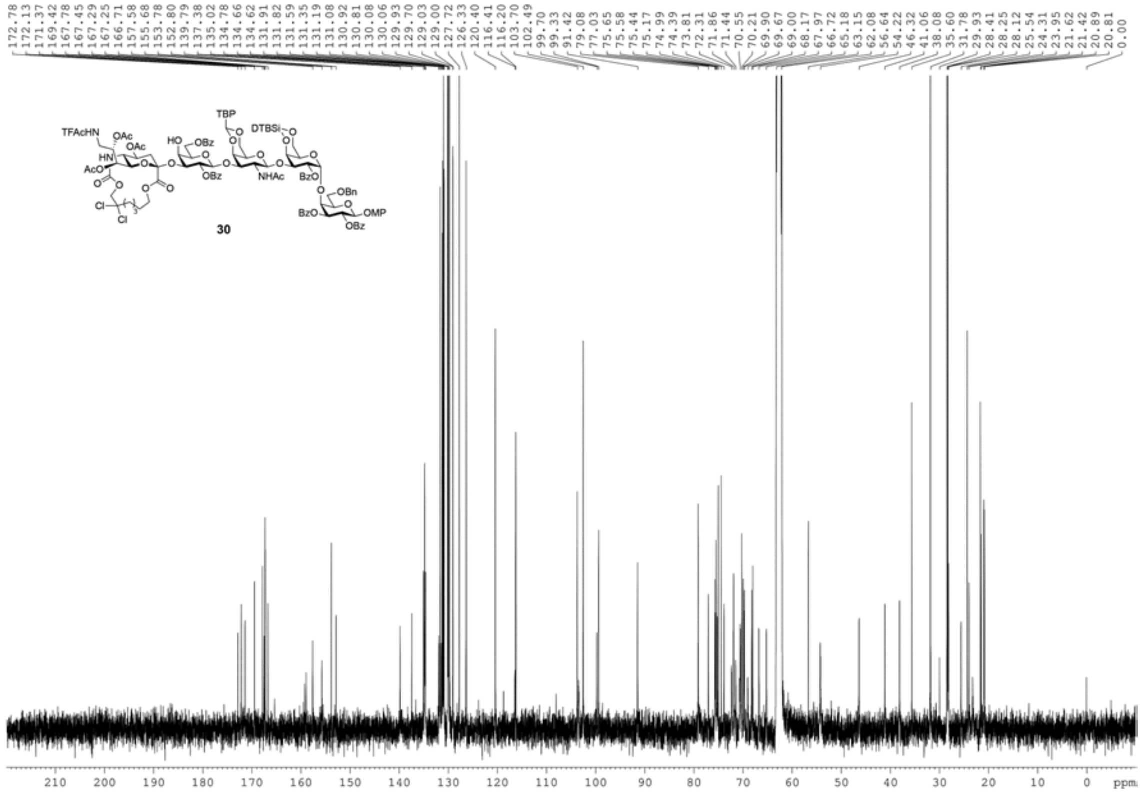
30
ppm

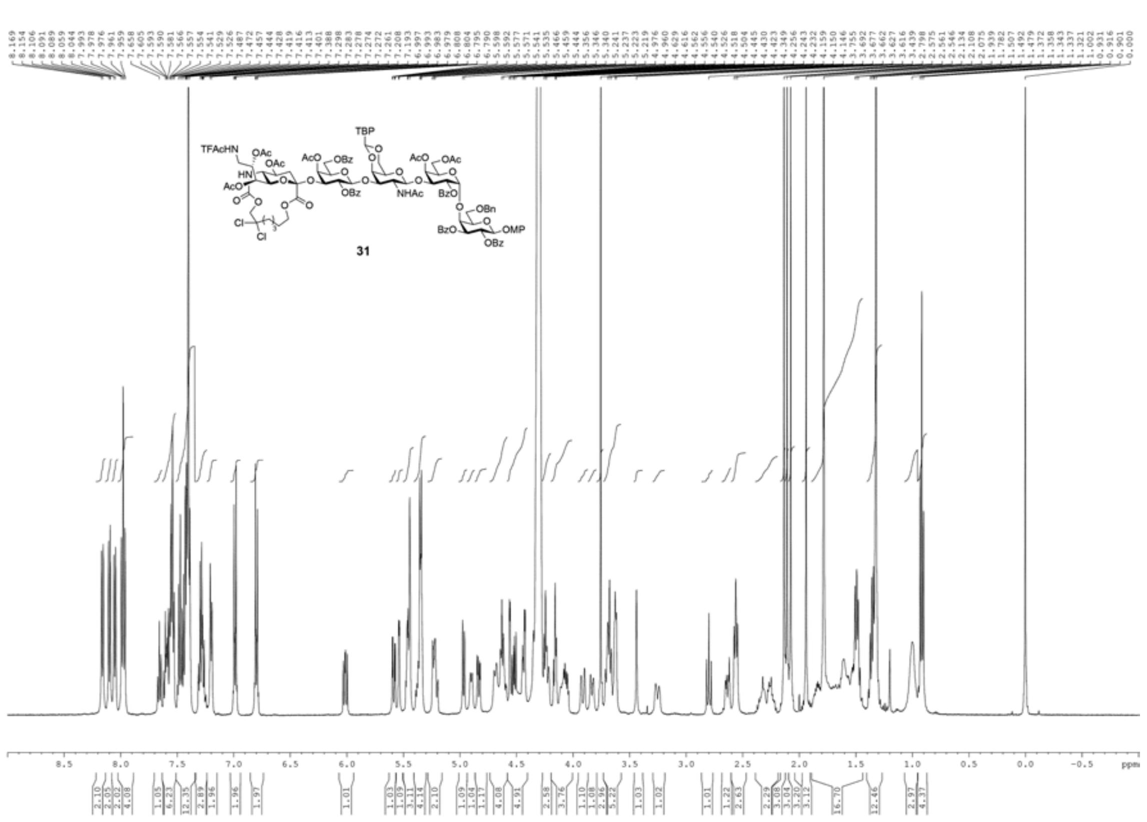

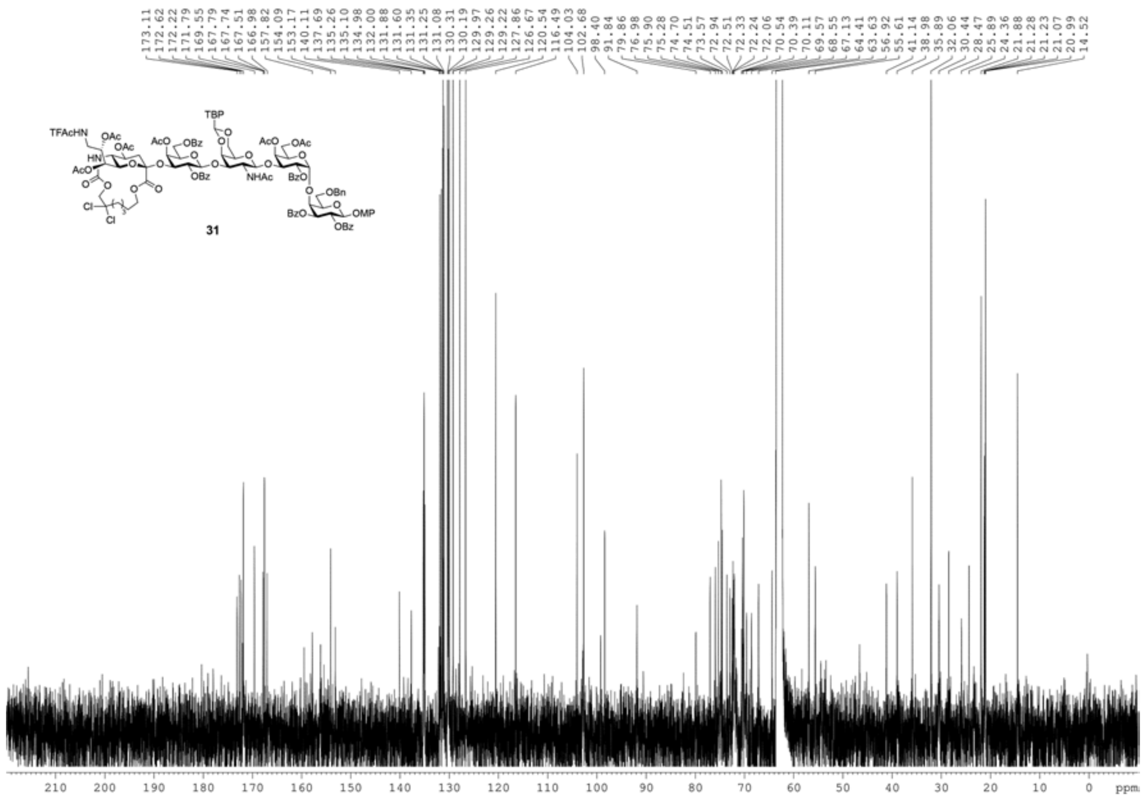

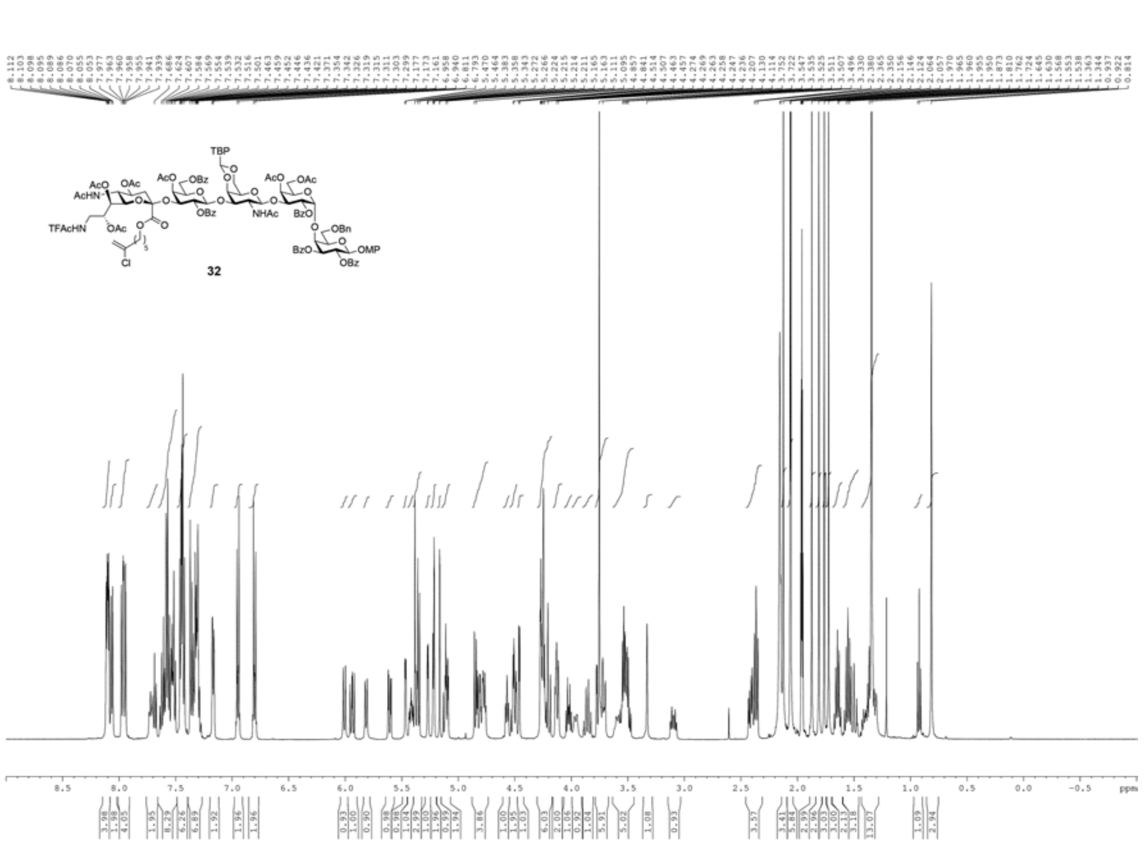
32
ppm

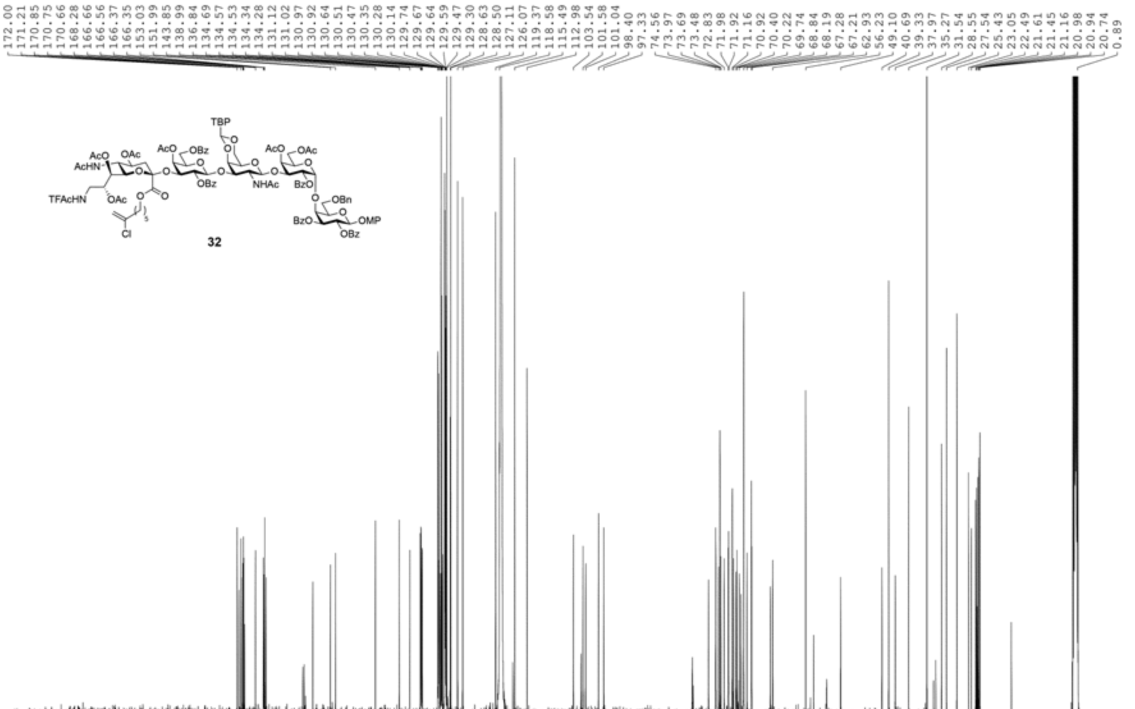
32
ppm

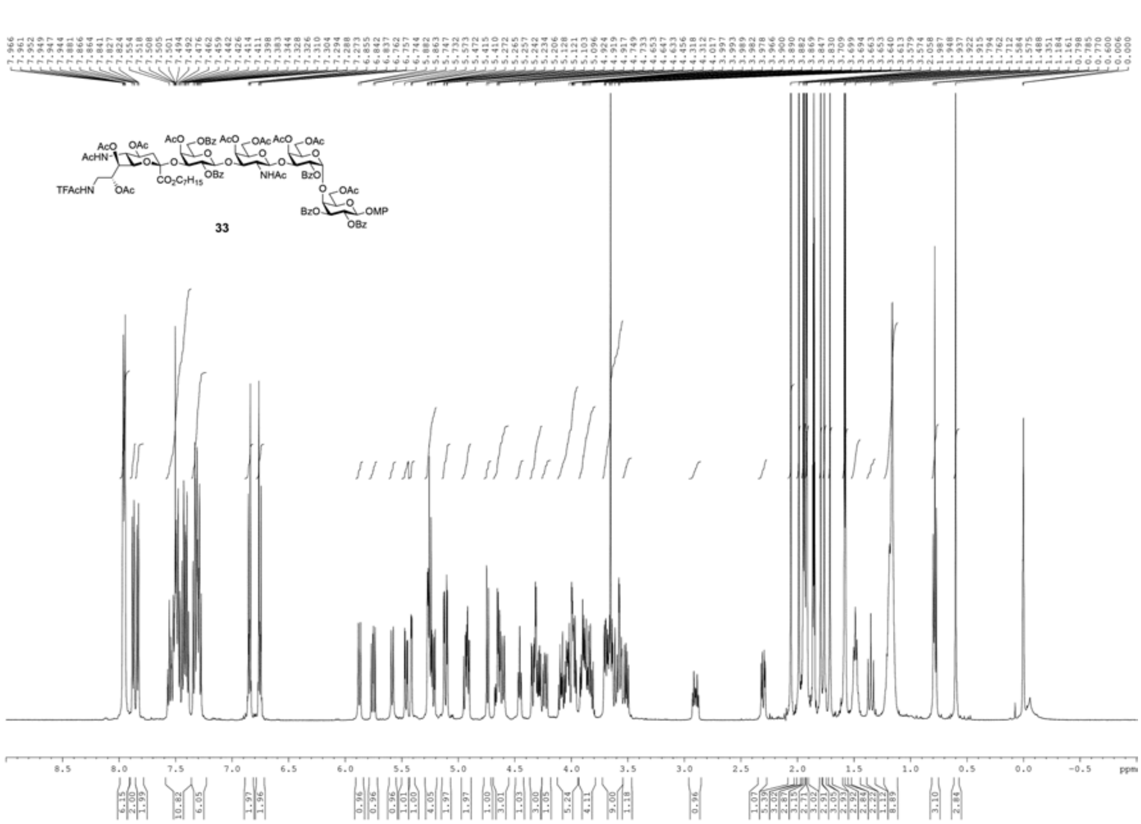
33
ppm

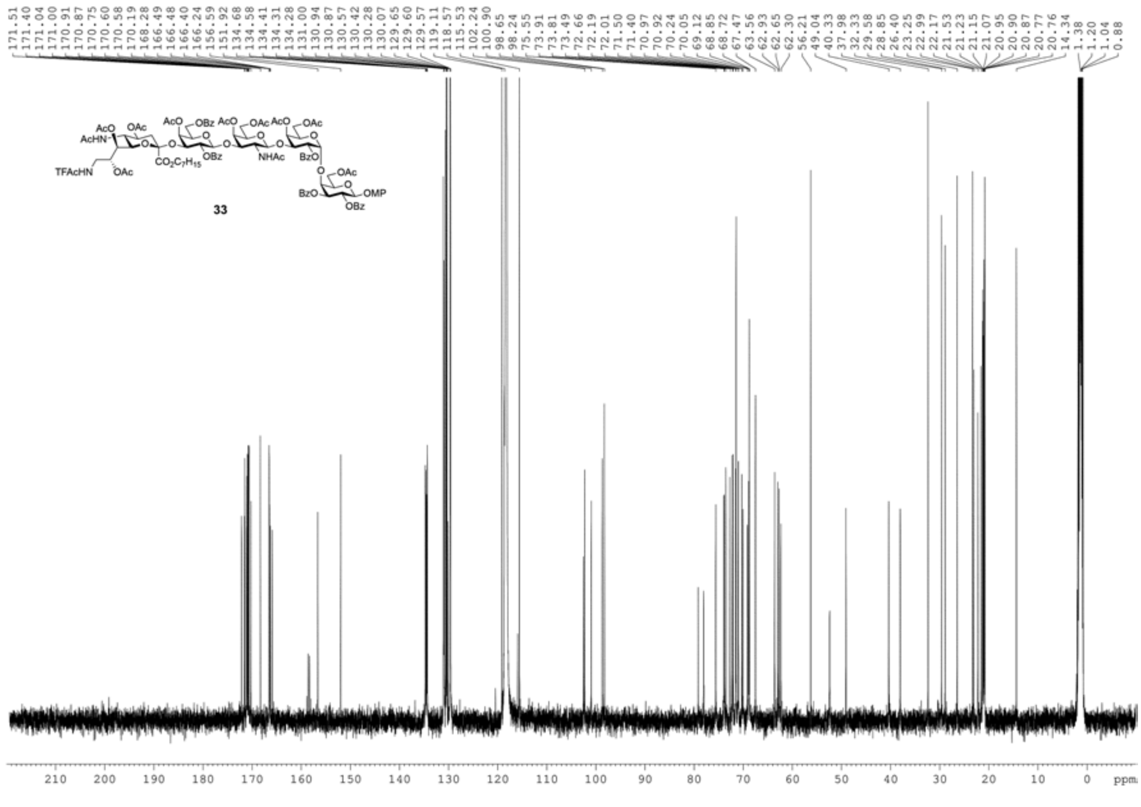
33
ppm

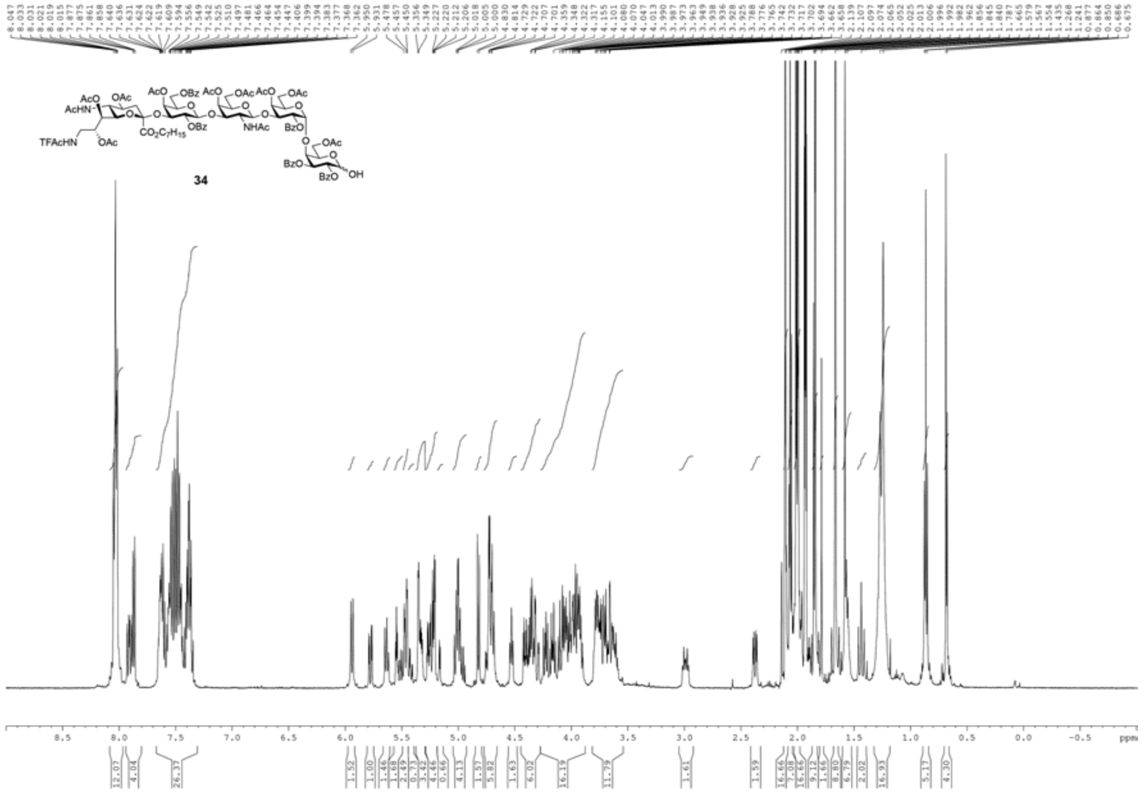

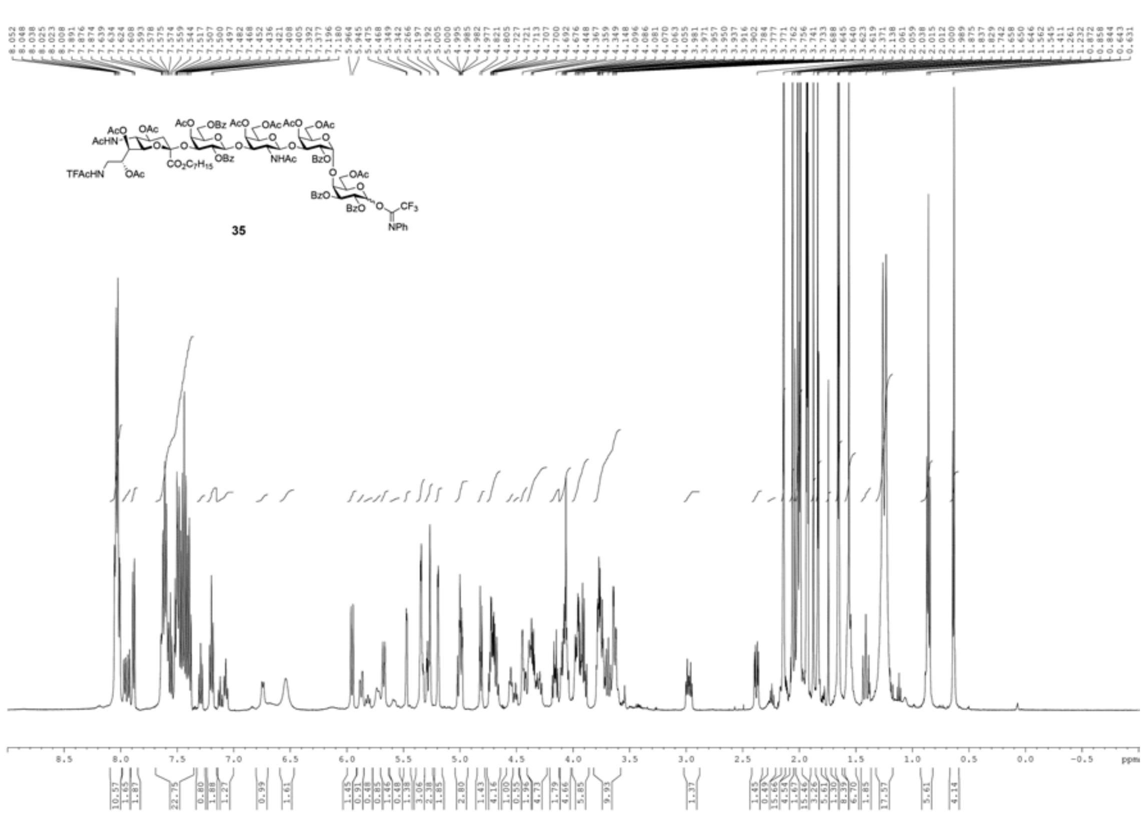

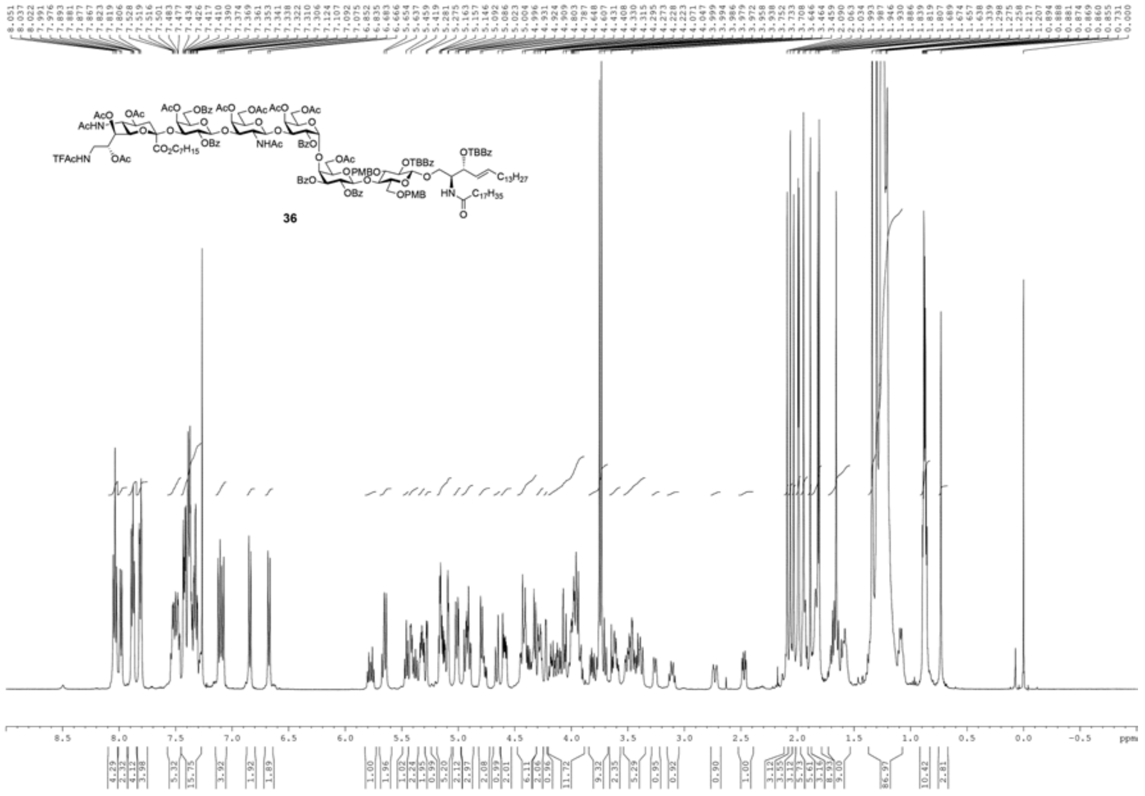

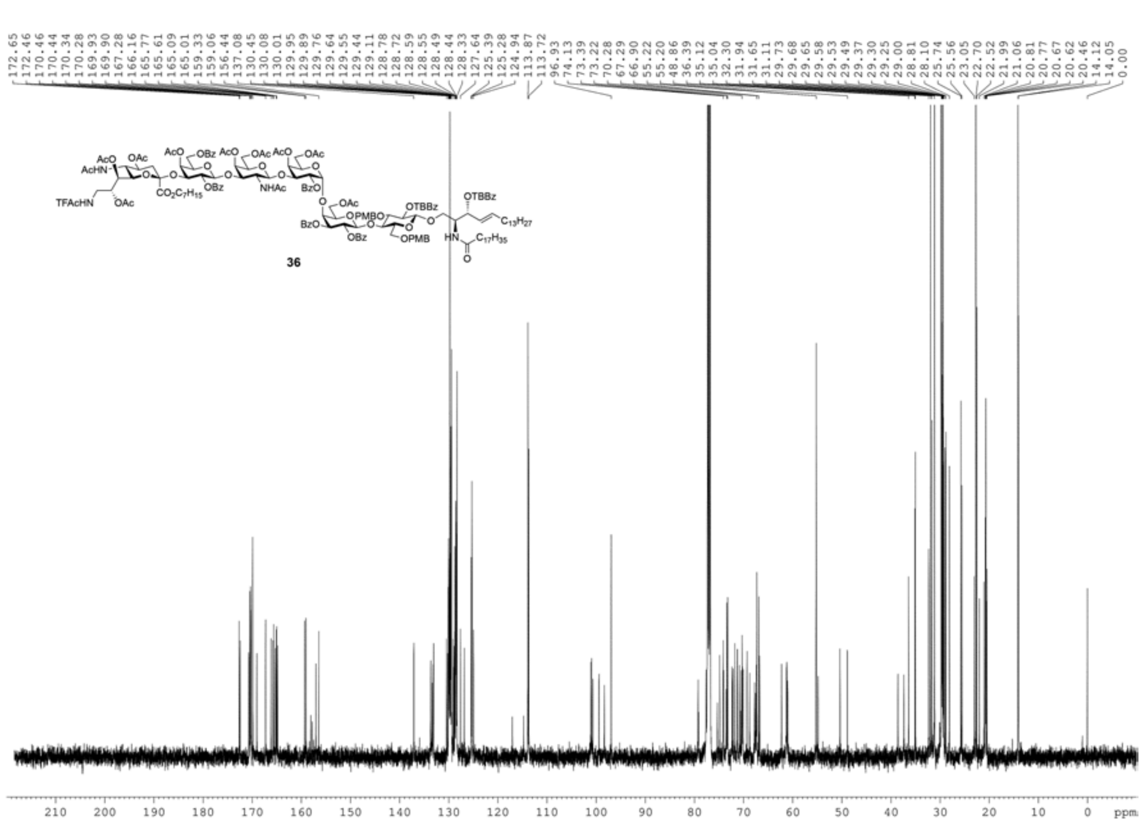

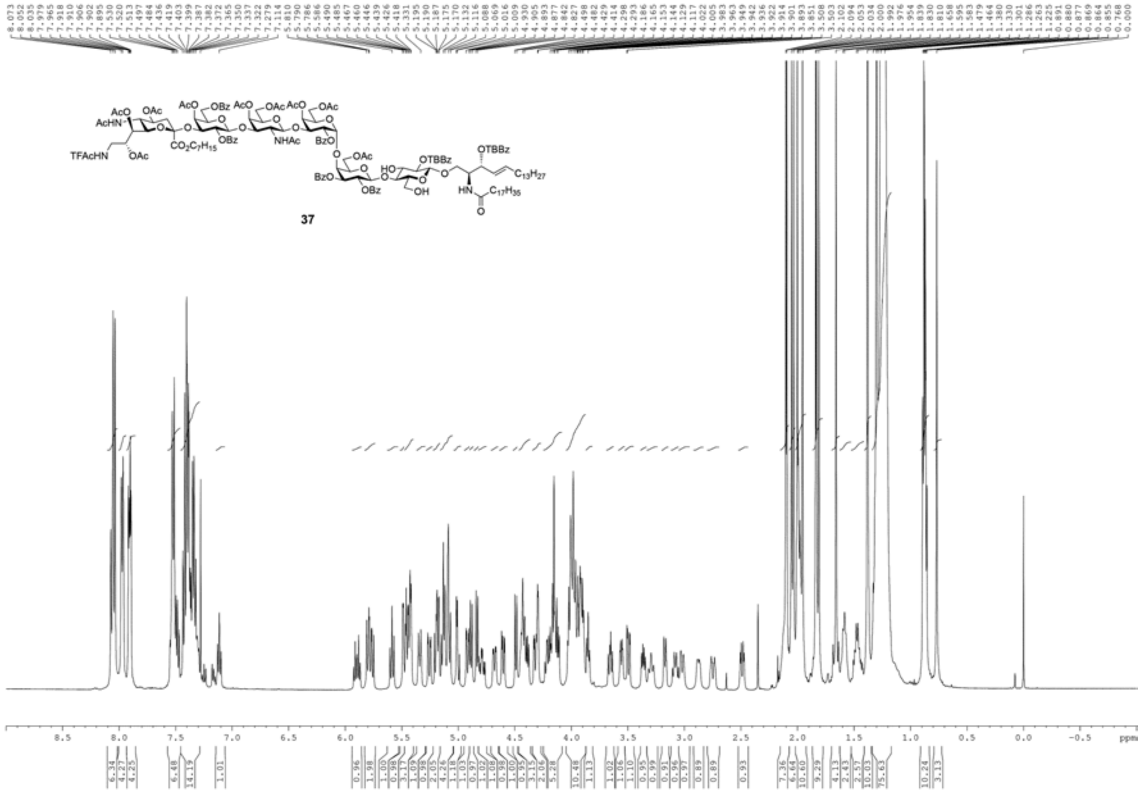

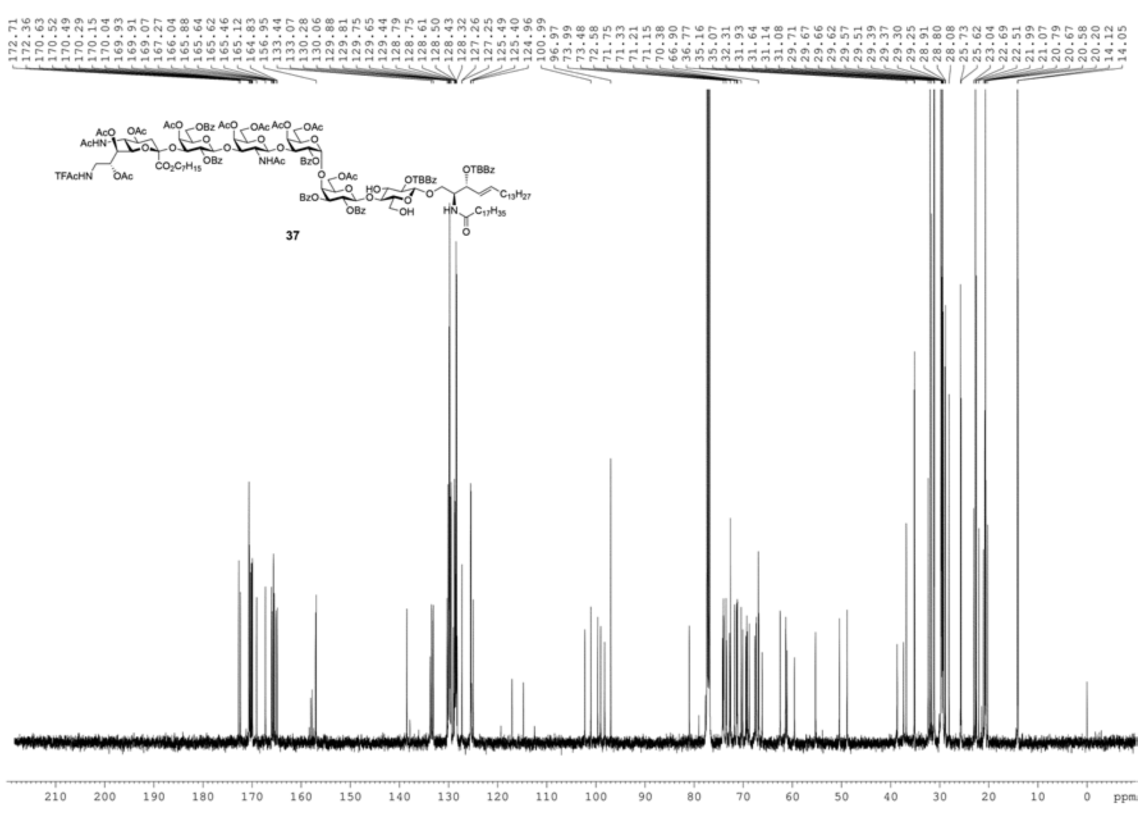

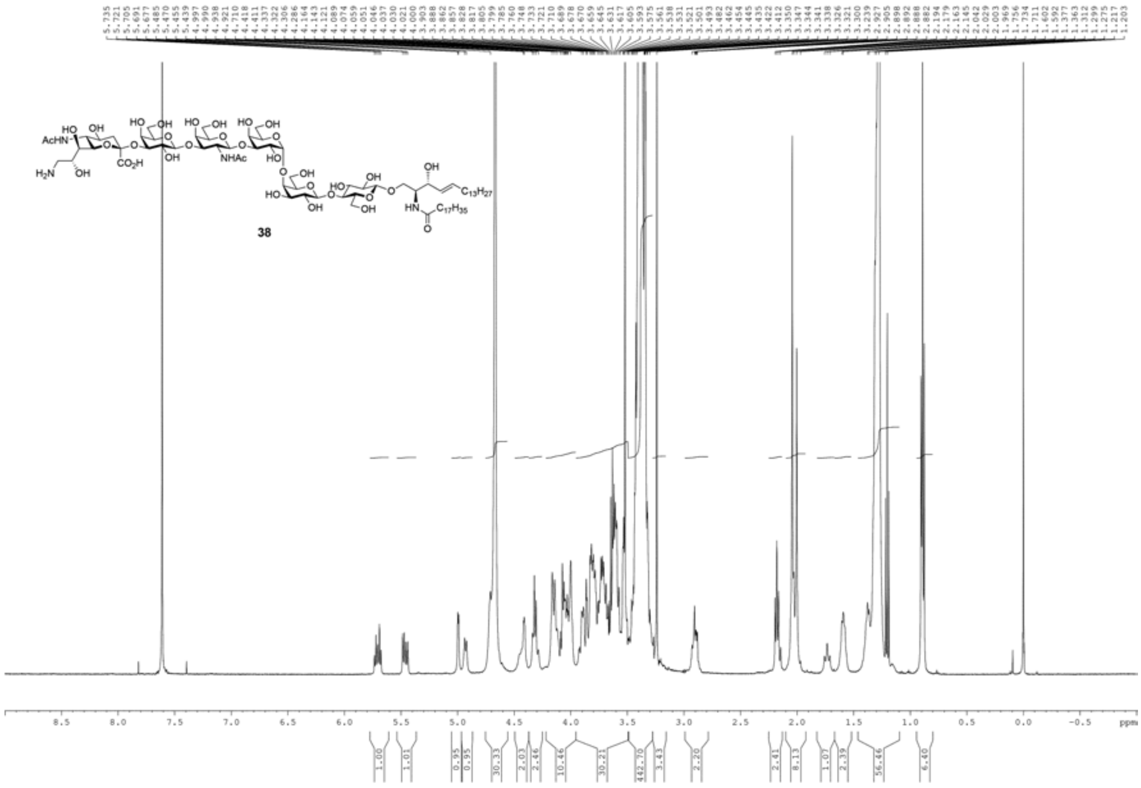
38
ppm

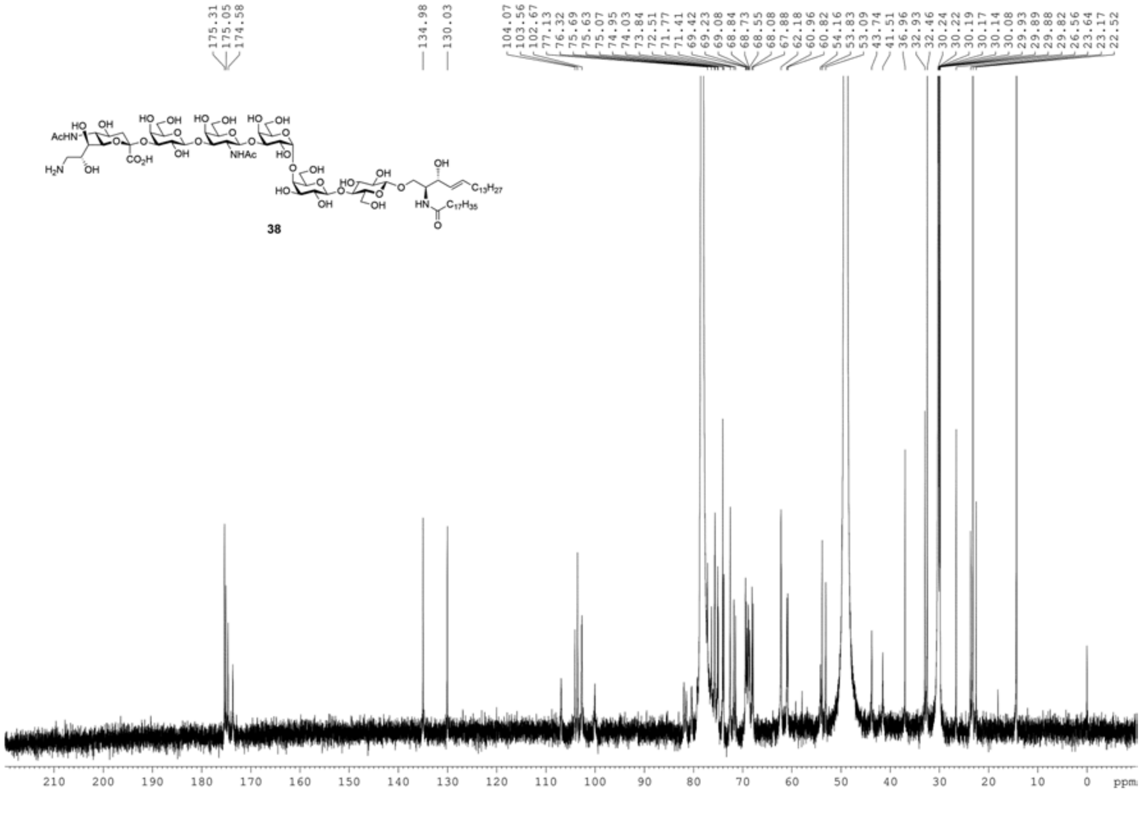
175.31
175.05
174.58
134.98
130.03
104.07
103.56
102.67
77.13
76.32
75.69
75.63
75.07
74.95
74.03
73.84
72.51
71.77
71.41
69.42
69.23
69.08
68.84
68.73
68.55
68.08
67.88
62.18
60.96
60.82
54.16
53.83
53.09
43.74
41.51
36.96
32.93
32.46
30.24
30.22
30.17
30.14
30.08
29.93
29.88
29.82
26.56
23.64
23.17
22.52
38
ppm

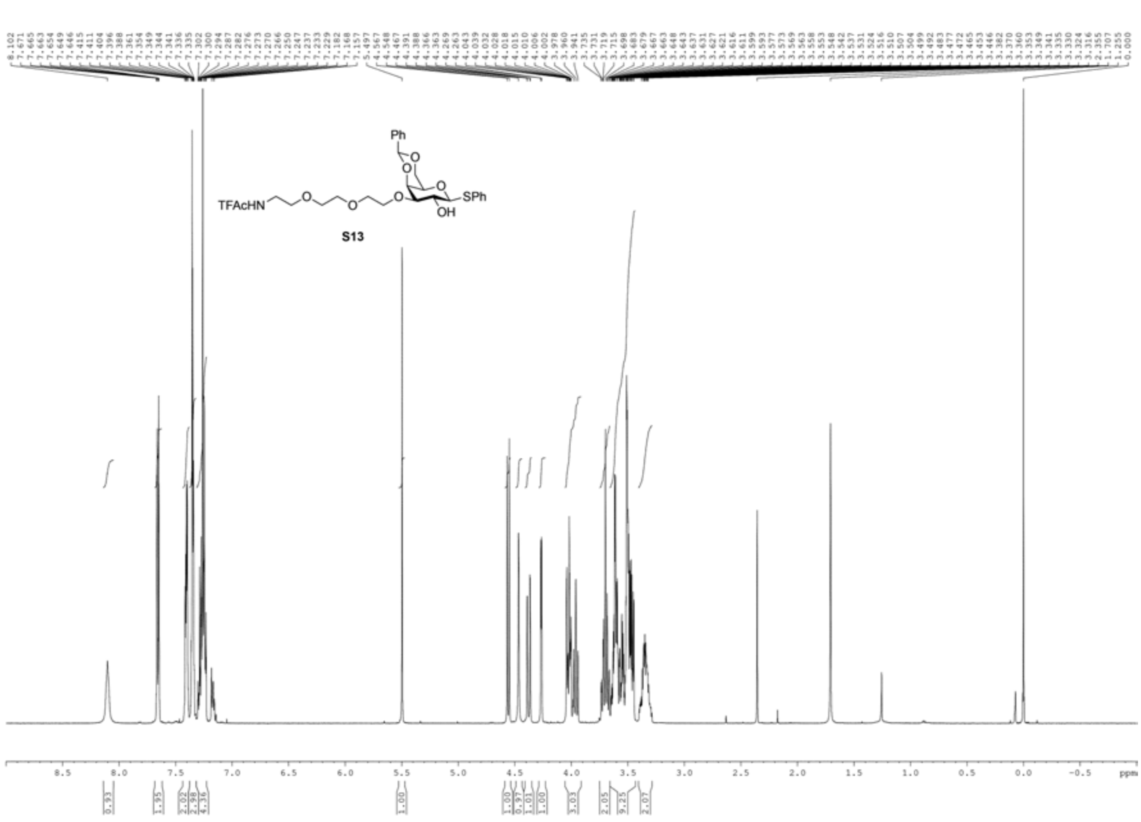

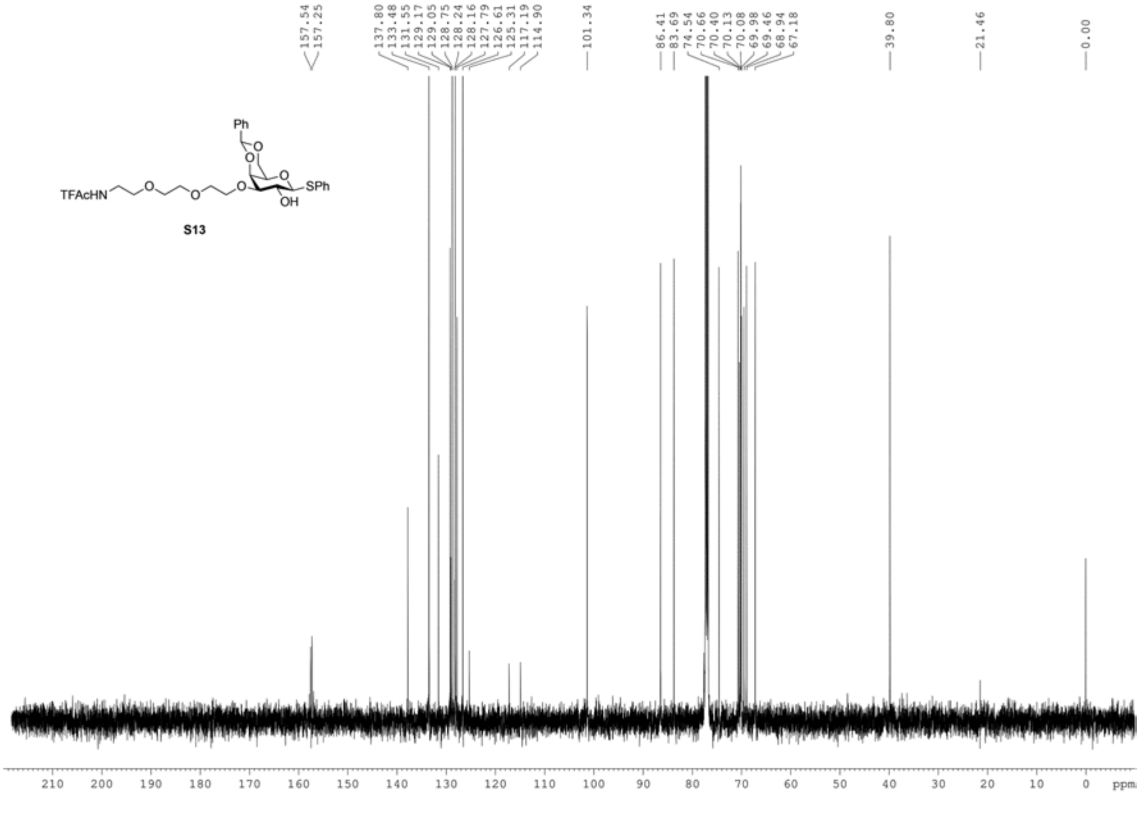

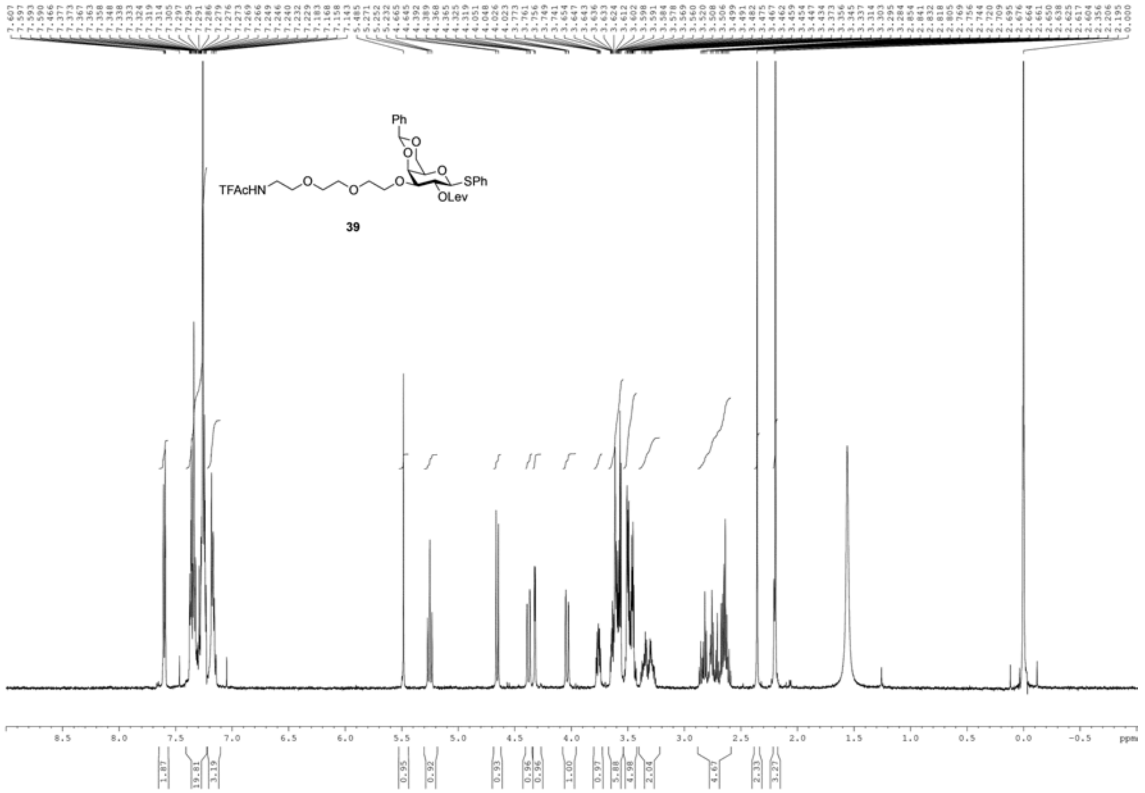

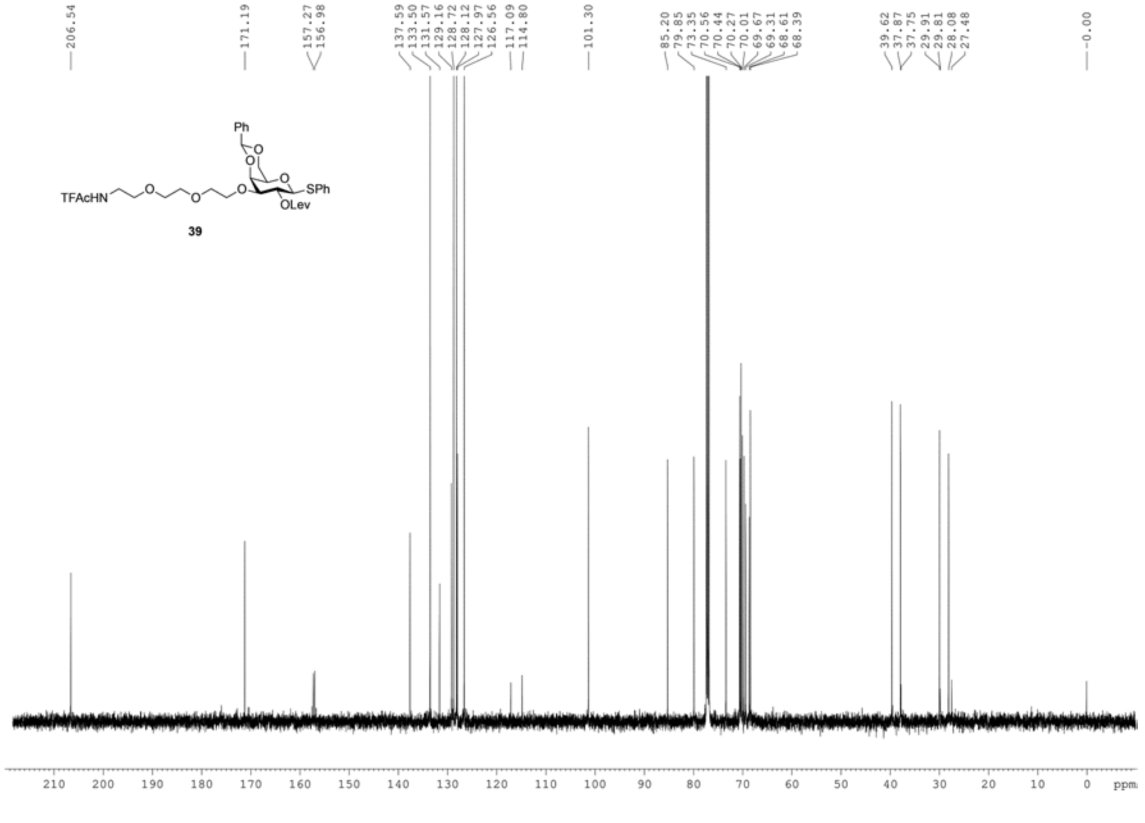

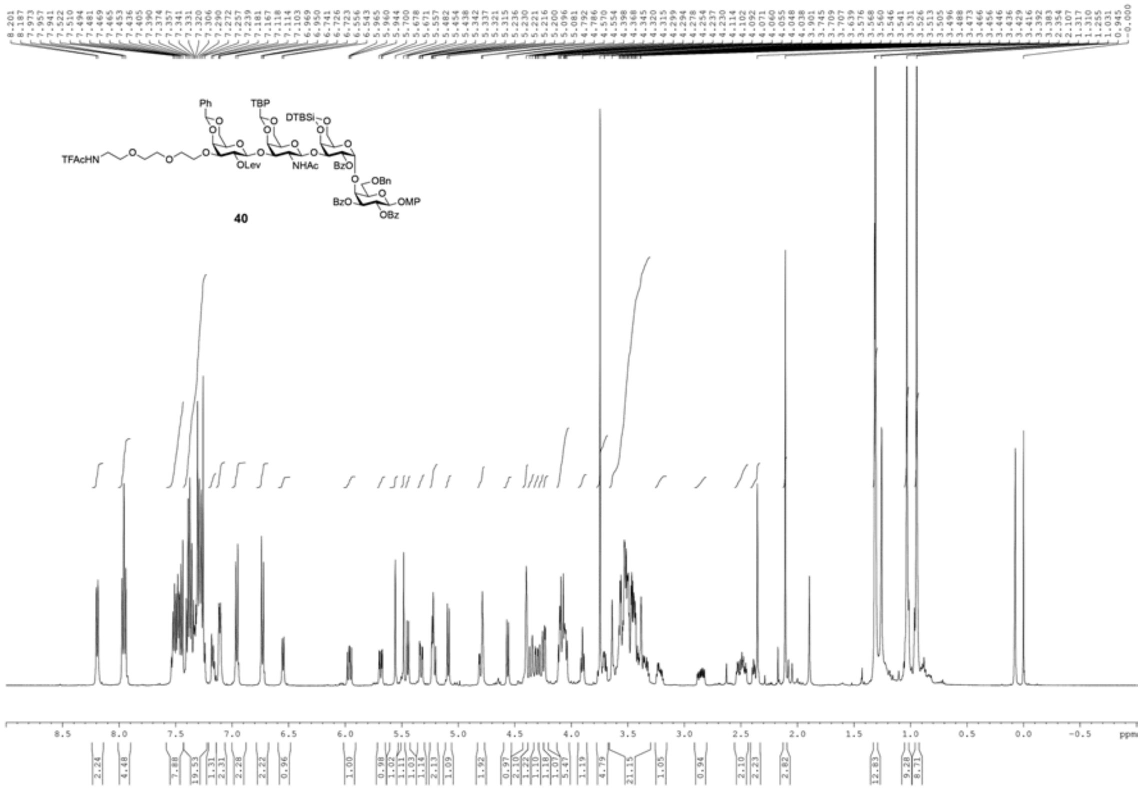

Ph
TBP
DTBSi
TFAcHN
OLev
NHAc
BzO
OBn
OMP
OBz
40
ppm

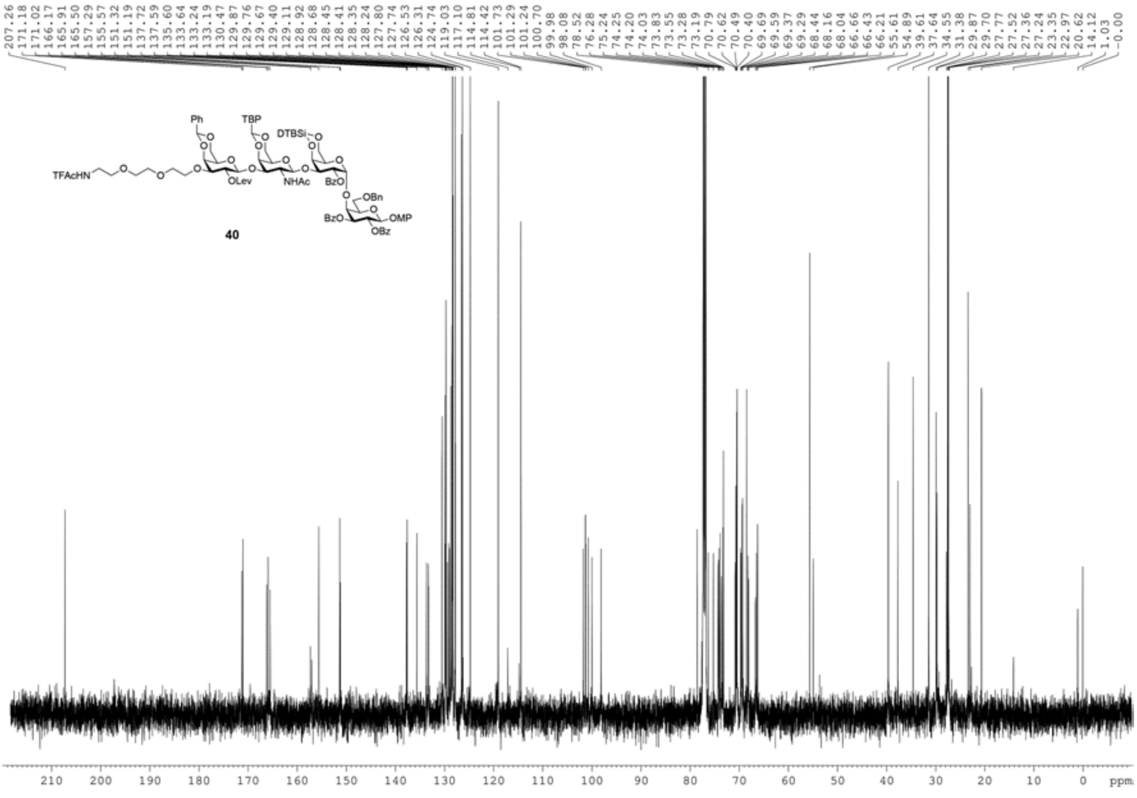

Ph
TBP
DTBSi
TFAcHN
OLev
NHAc
BzO
OBn
OMP
OBz
40
ppm

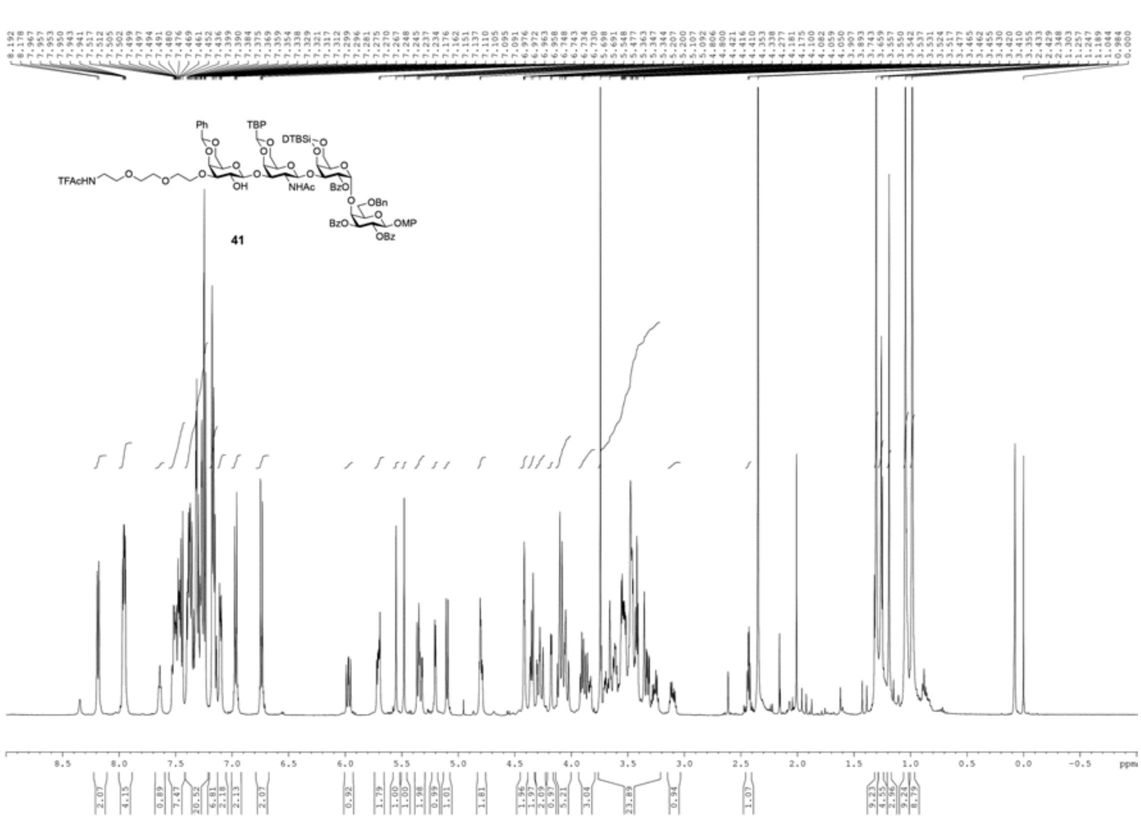
Ph
TBP
DTBSi
TFAcHN
OH
NHAc
BzO
OBn
BzO
OMP
OBz
41
ppm

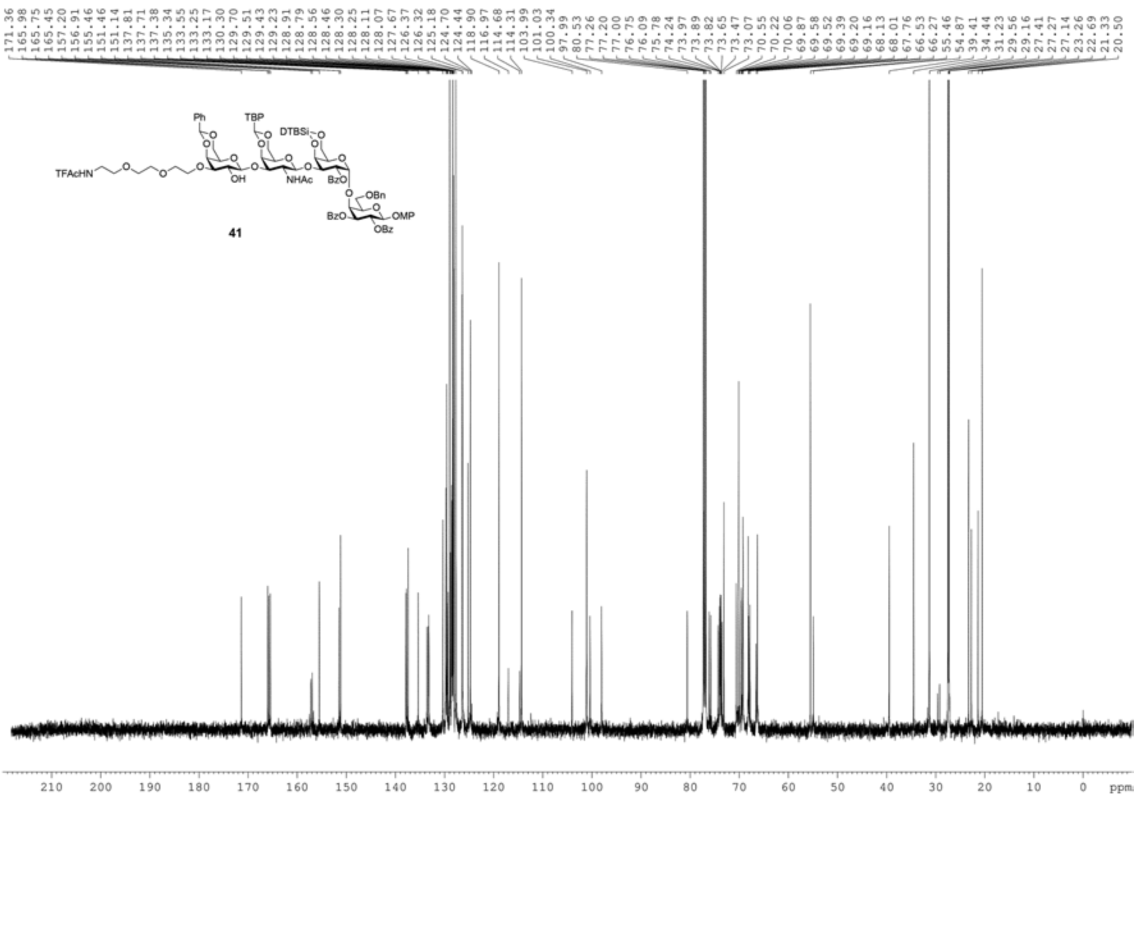
171.36
165.98
165.75
165.45
157.20
156.91
155.46
151.14
137.81
137.71
137.38
135.34
133.55
133.25
133.17
130.30
129.70
129.51
129.43
129.23
128.91
128.79
128.56
128.46
128.30
128.25
128.11
128.07
127.67
126.32
125.18
124.70
124.44
118.90
116.97
114.68
114.31
103.99
101.03
100.34
97.99
80.53
77.26
77.20
77.00
76.75
76.09
75.78
74.24
73.97
73.89
73.82
73.65
73.47
73.07
70.55
70.22
70.06
69.87
69.58
69.52
69.39
69.20
69.16
68.13
68.01
67.76
66.53
66.27
55.46
54.87
39.41
34.44
31.23
29.56
29.16
27.41
27.27
27.14
23.26
22.69
21.33
20.50
Ph
TBP
DTBSi
TFAcHN
OH
NHAc
BzO
OBn
BzO
OMP
OBz
41
ppm

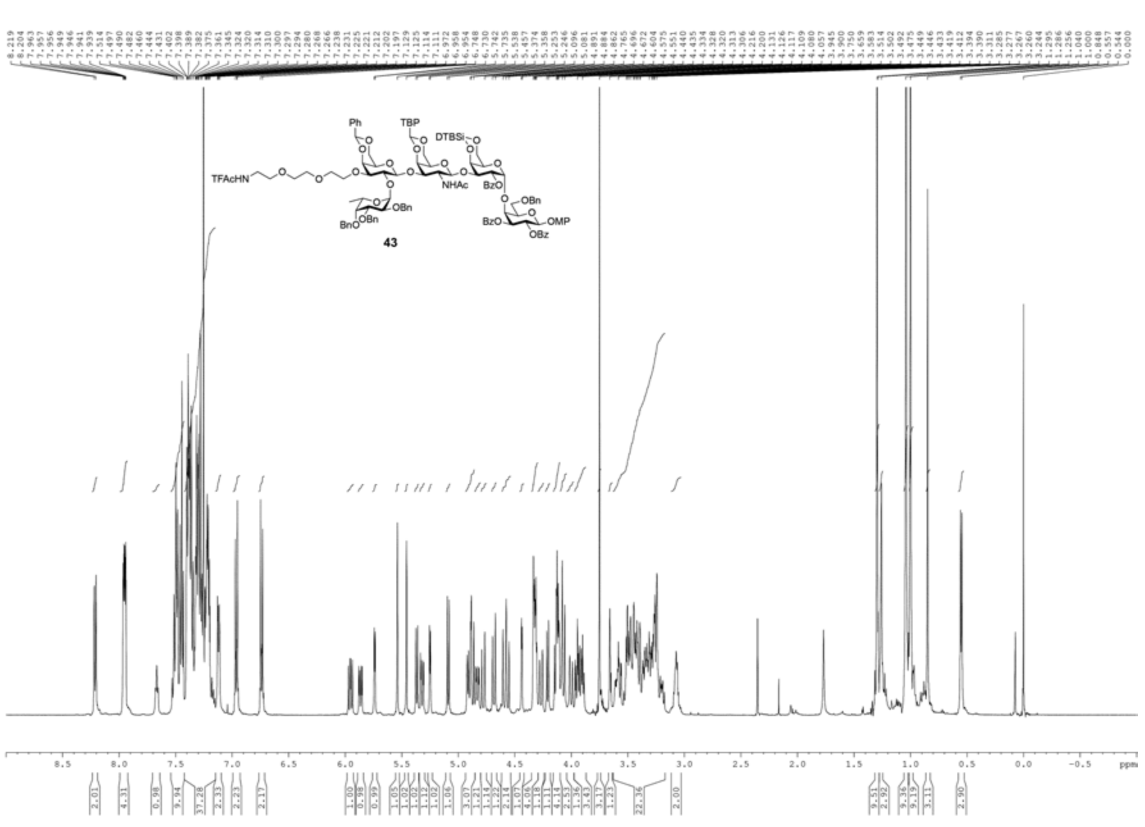

43

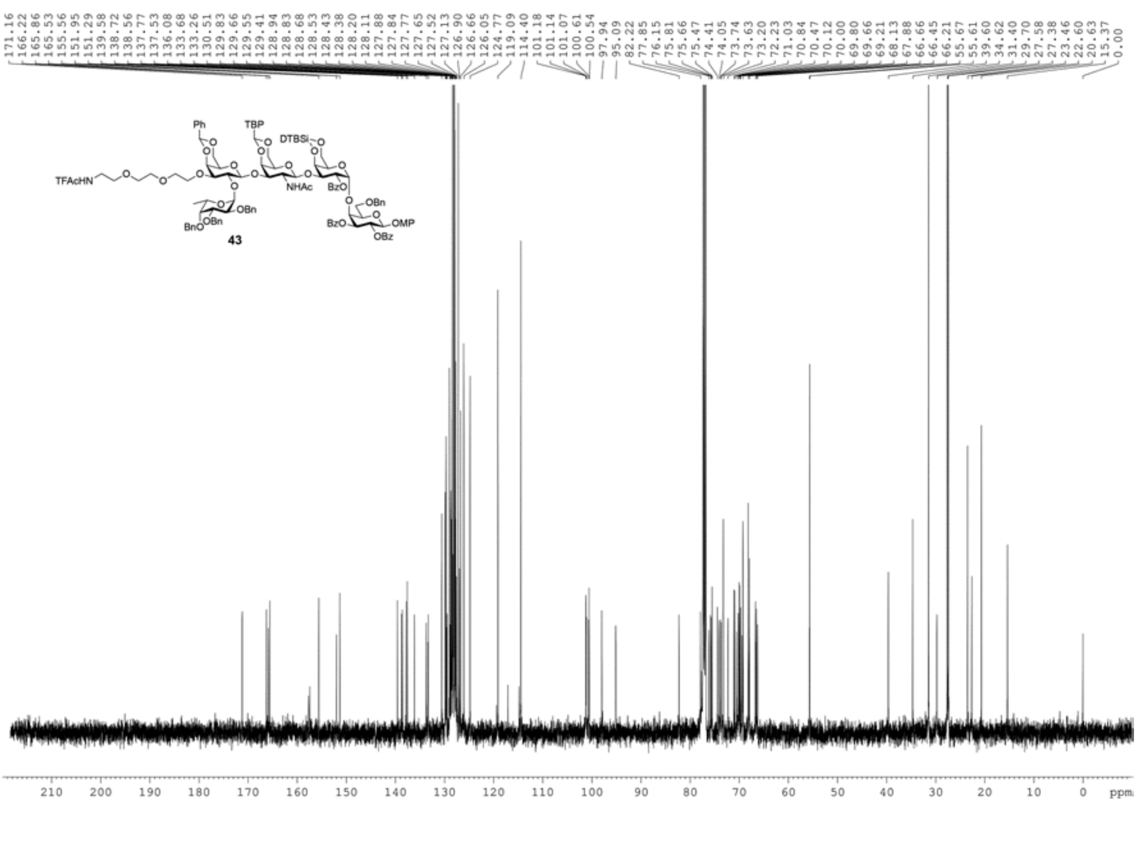

43

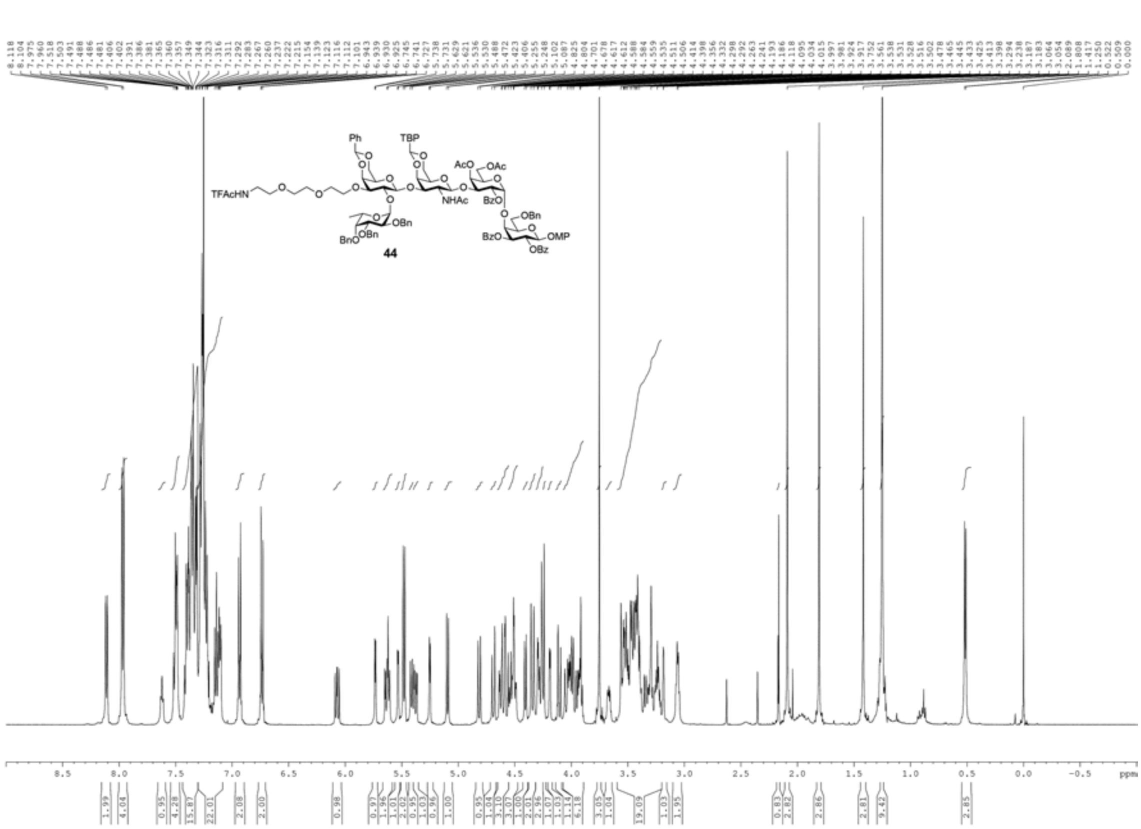
TFAcHN
Ph
TBP
AcO
OAc
NHAc
BzO
OBn
BzO
OMP
OBz
OBn
BnO
OBn
44
ppm

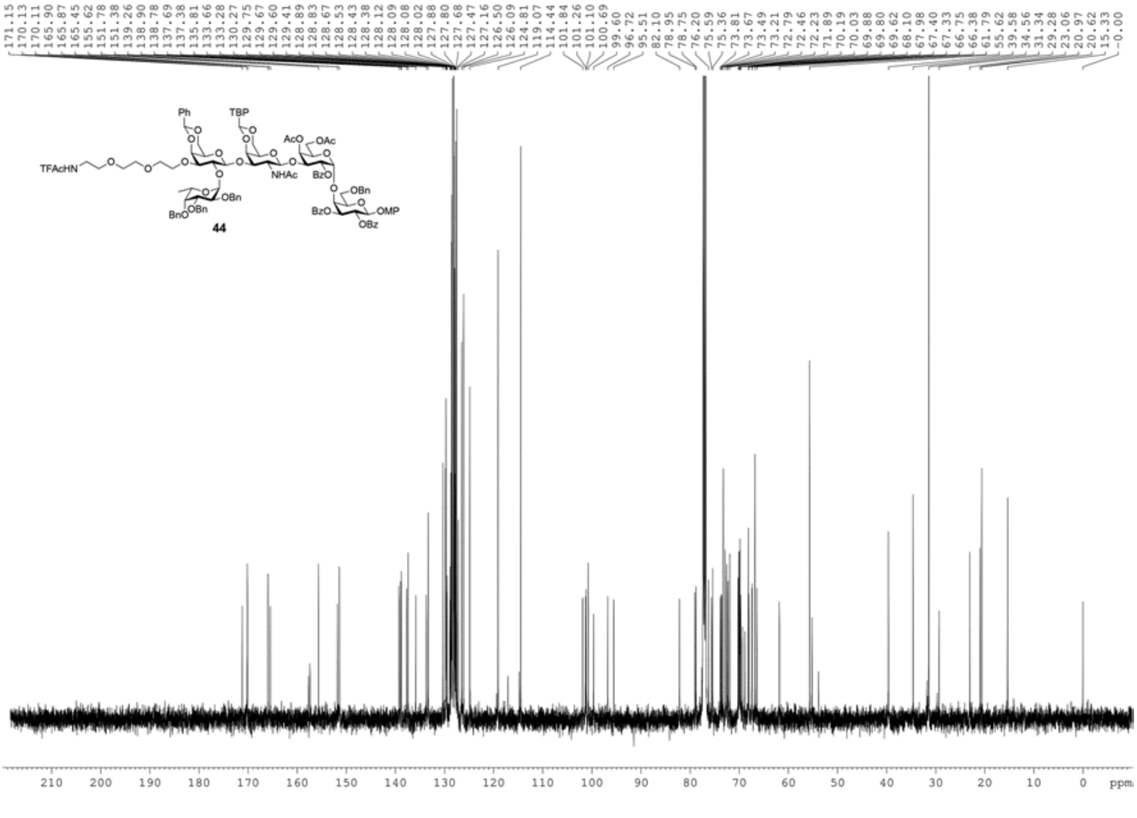
171.15
170.13
170.11
165.90
165.87
165.45
155.62
151.78
151.38
139.26
138.90
138.78
137.69
137.38
135.81
133.66
133.28
130.27
129.75
129.67
129.60
129.41
128.89
128.83
128.67
128.53
128.43
128.38
128.12
128.09
128.08
128.02
127.88
127.80
127.68
127.47
127.16
126.50
126.59
124.81
119.07
114.44
101.84
101.26
101.10
100.69
99.60
96.72
95.51
82.10
78.95
78.75
76.20
75.59
75.36
73.81
73.67
73.49
73.21
72.79
72.46
72.23
71.89
70.19
70.03
69.88
69.80
69.62
68.10
67.98
67.40
67.33
66.75
66.38
61.79
55.62
39.58
34.56
31.34
29.28
23.06
20.97
20.62
15.33
-0.00
TFAcHN
Ph
TBP
AcO
OAc
NHAc
BzO
OBn
BzO
OMP
OBz
OBn
BnO
OBn
44
ppm

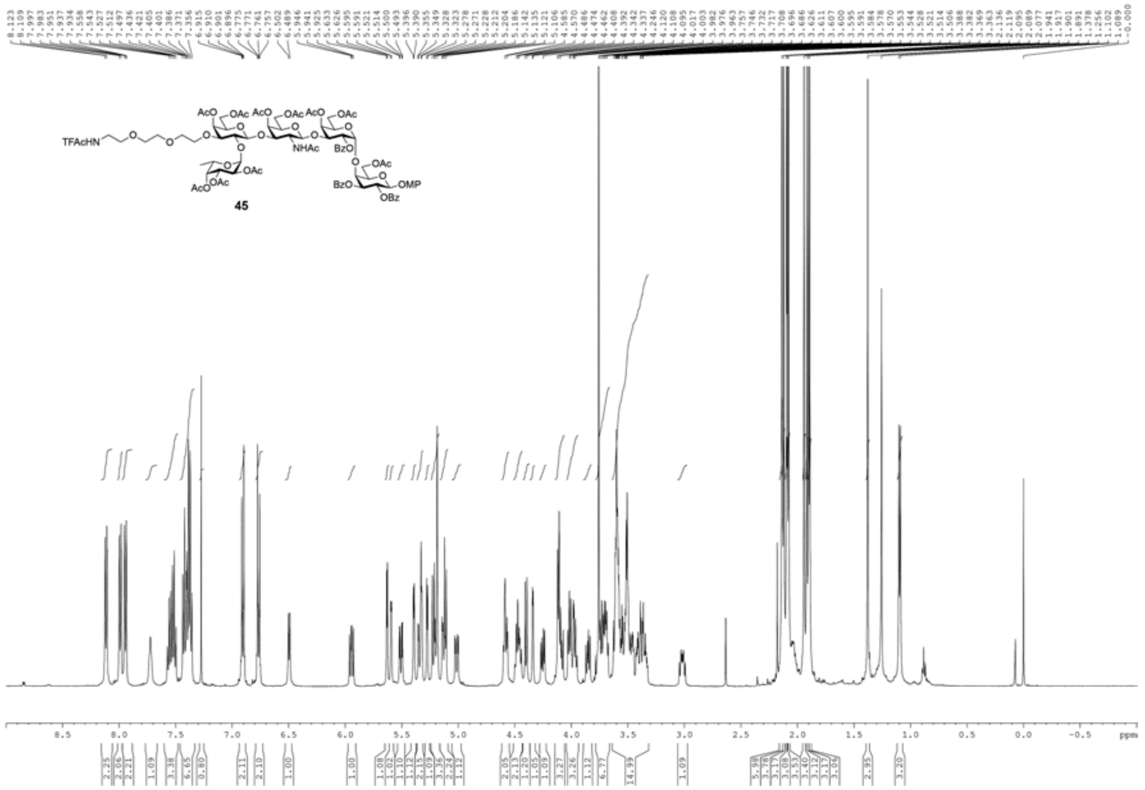
TFAcHN
AcO
OAc
NHAc
BzO
OMP
OBz
45
ppm

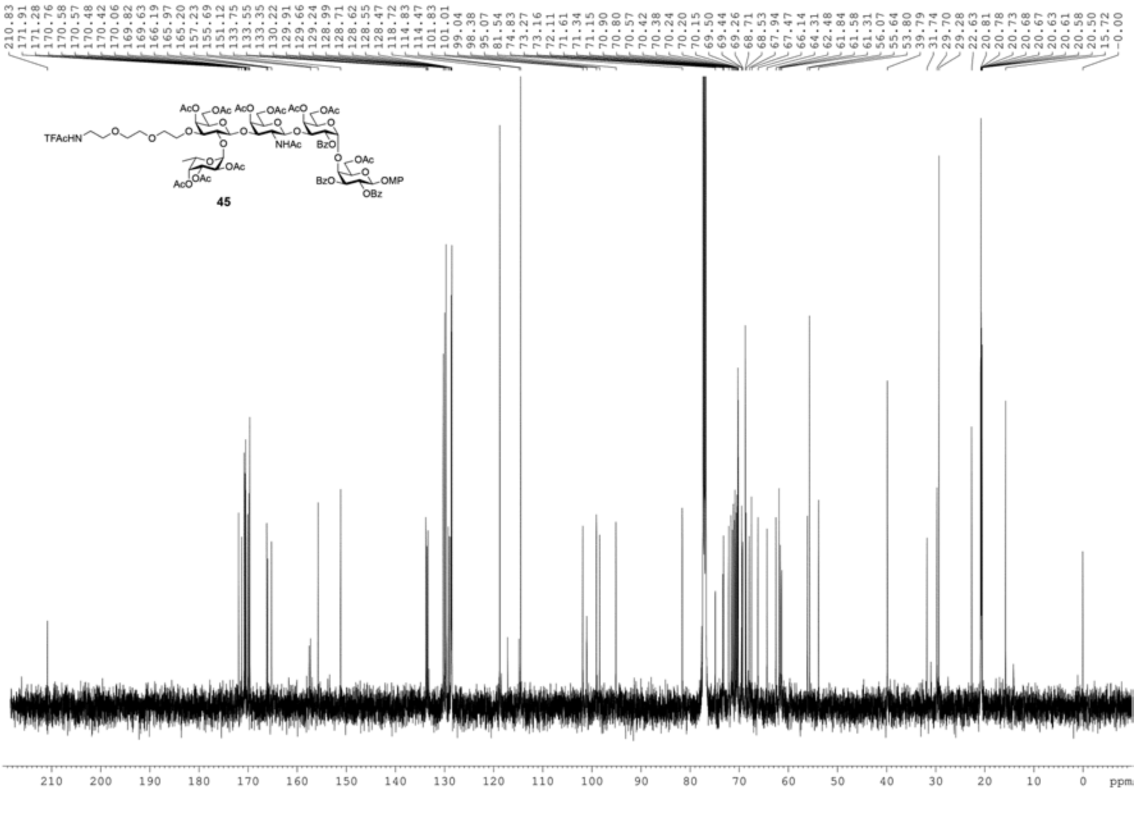
TFAcHN
AcO
OAc
NHAc
BzO
OMP
OBz
45
ppm

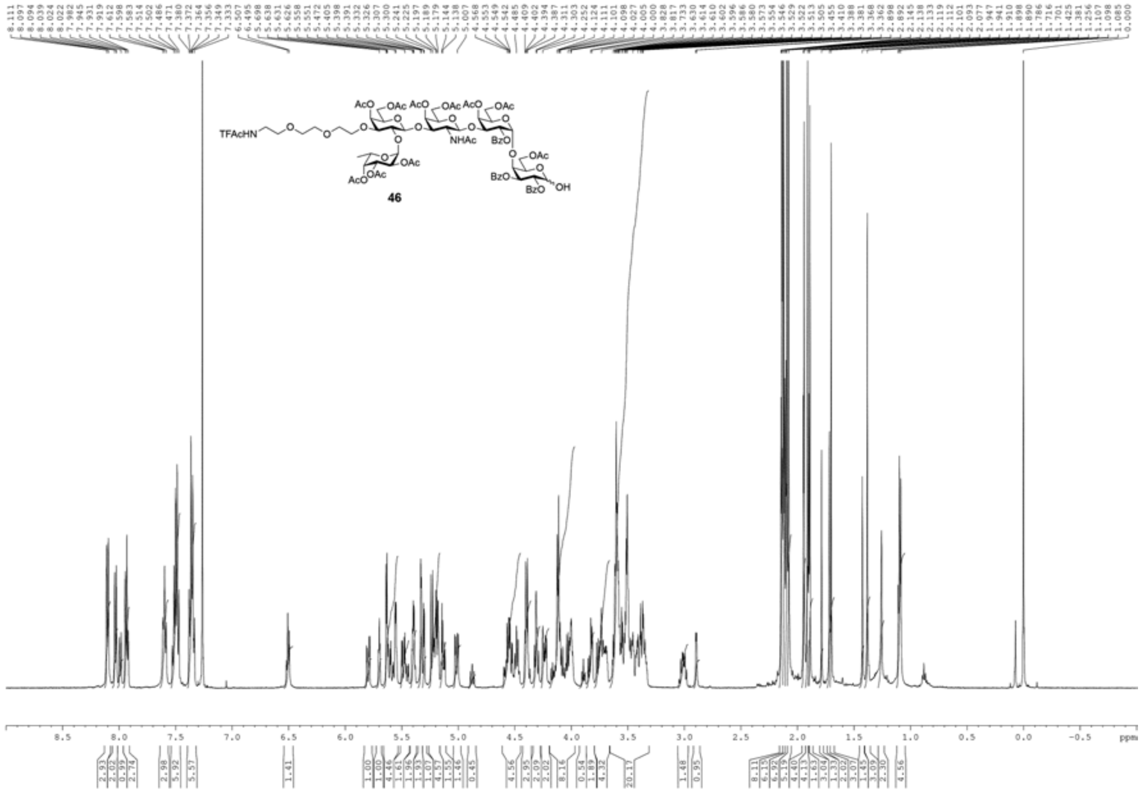

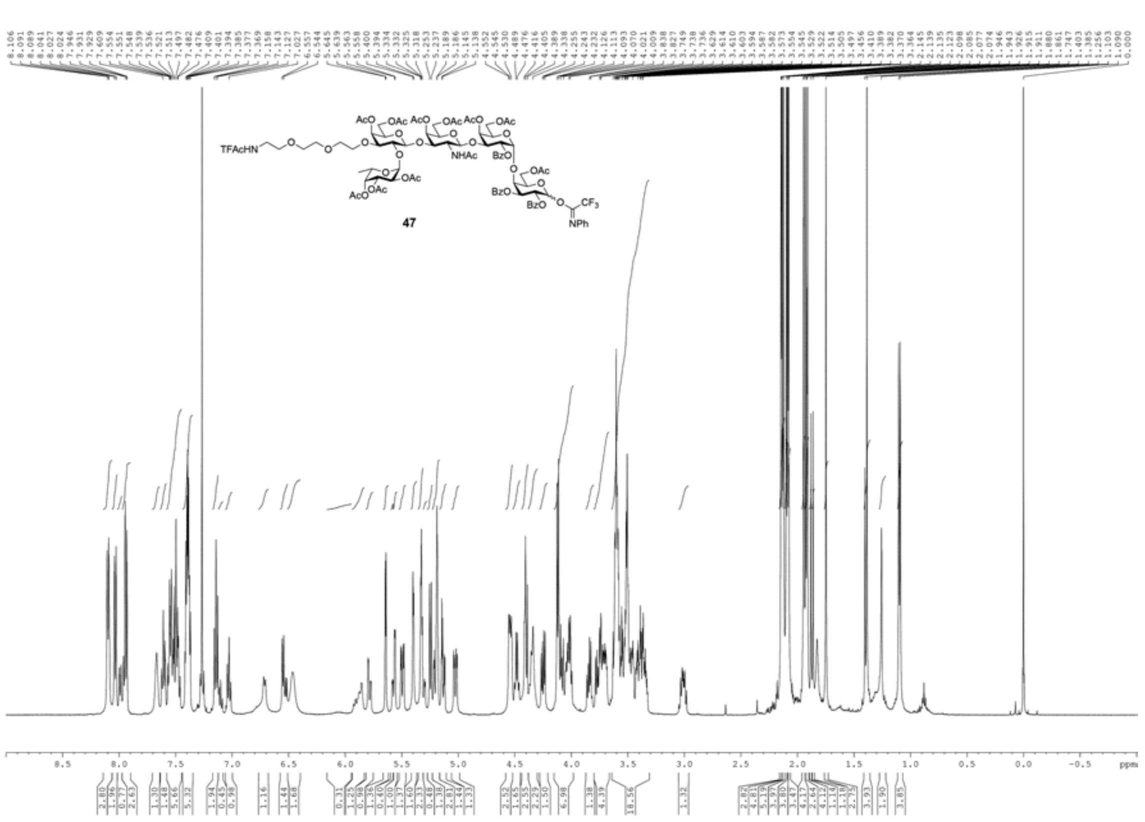
TFAcHN
AcO
OAc
NHAc
BzO
CF3
NPh
47
ppm

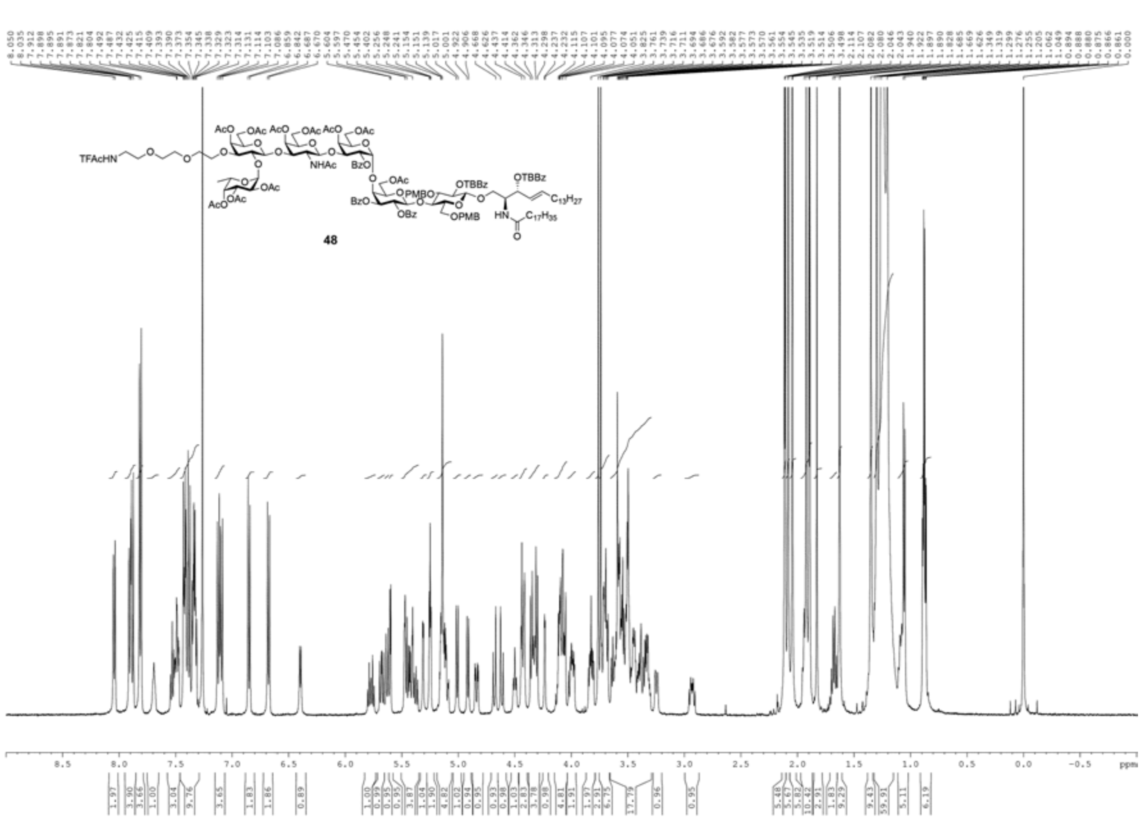

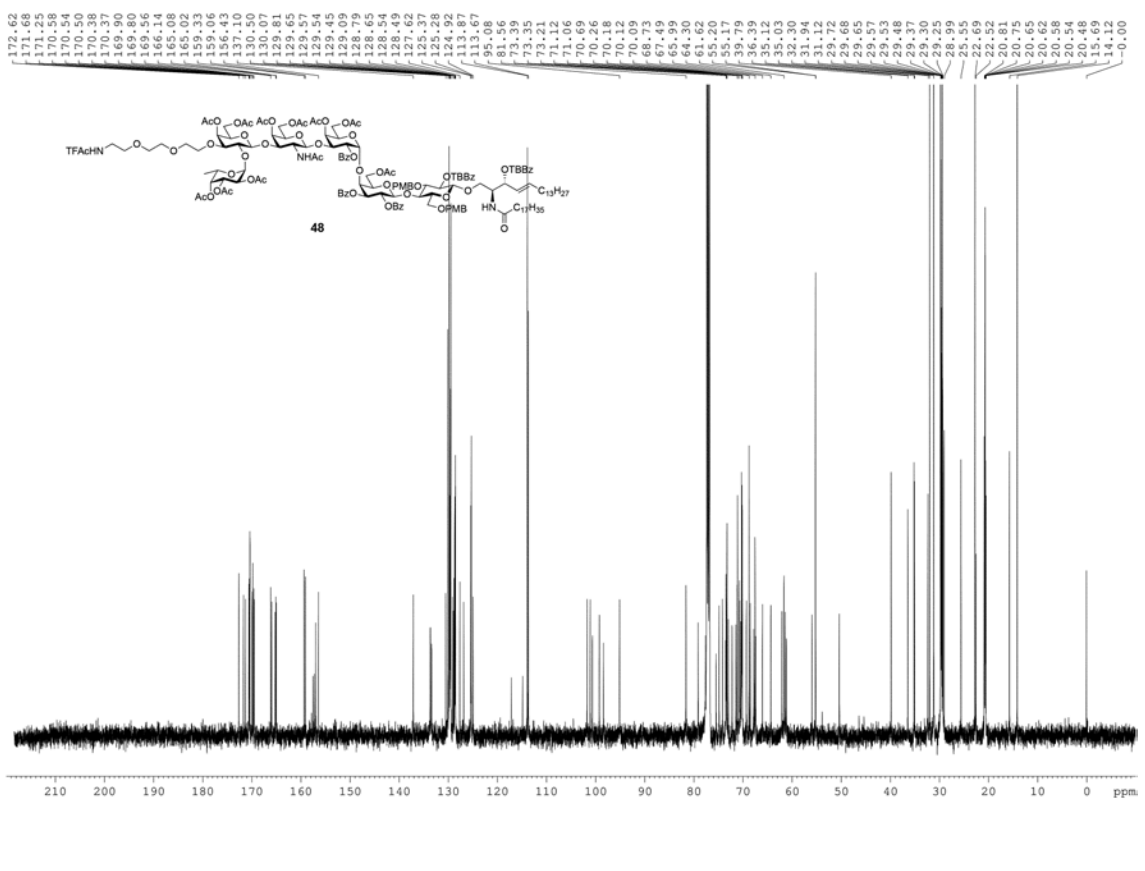

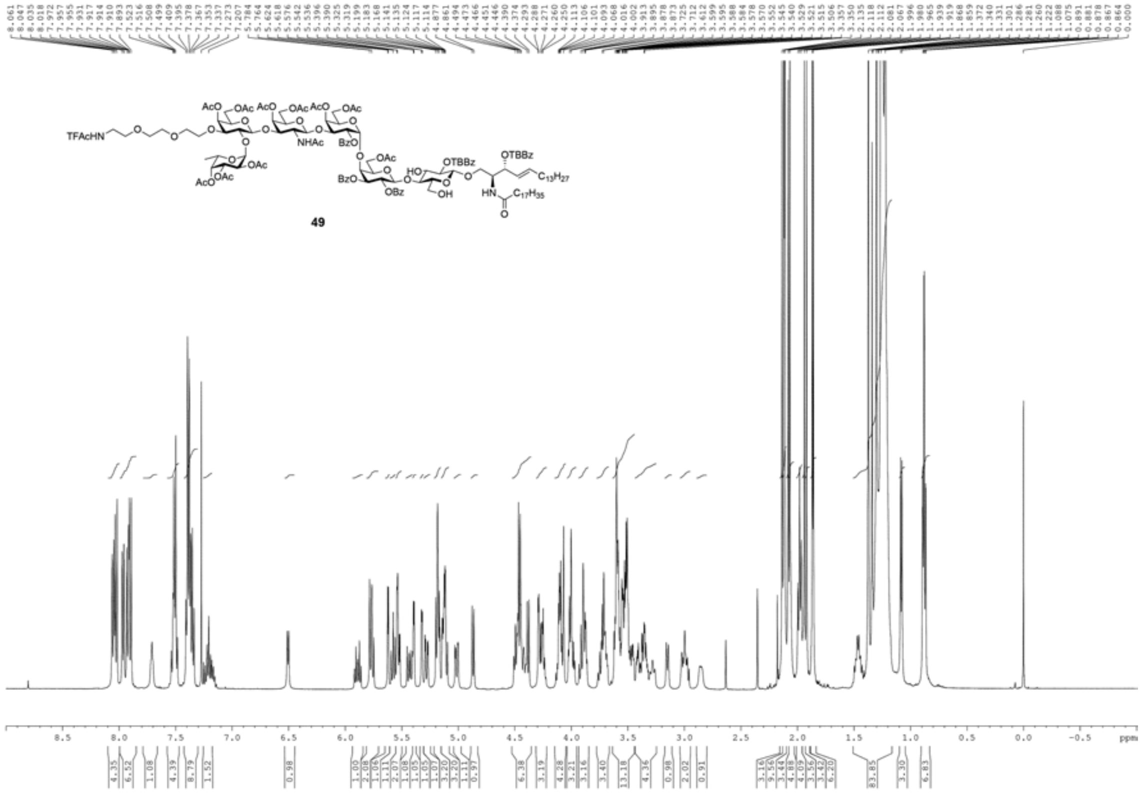
TFAcHN
49
ppm

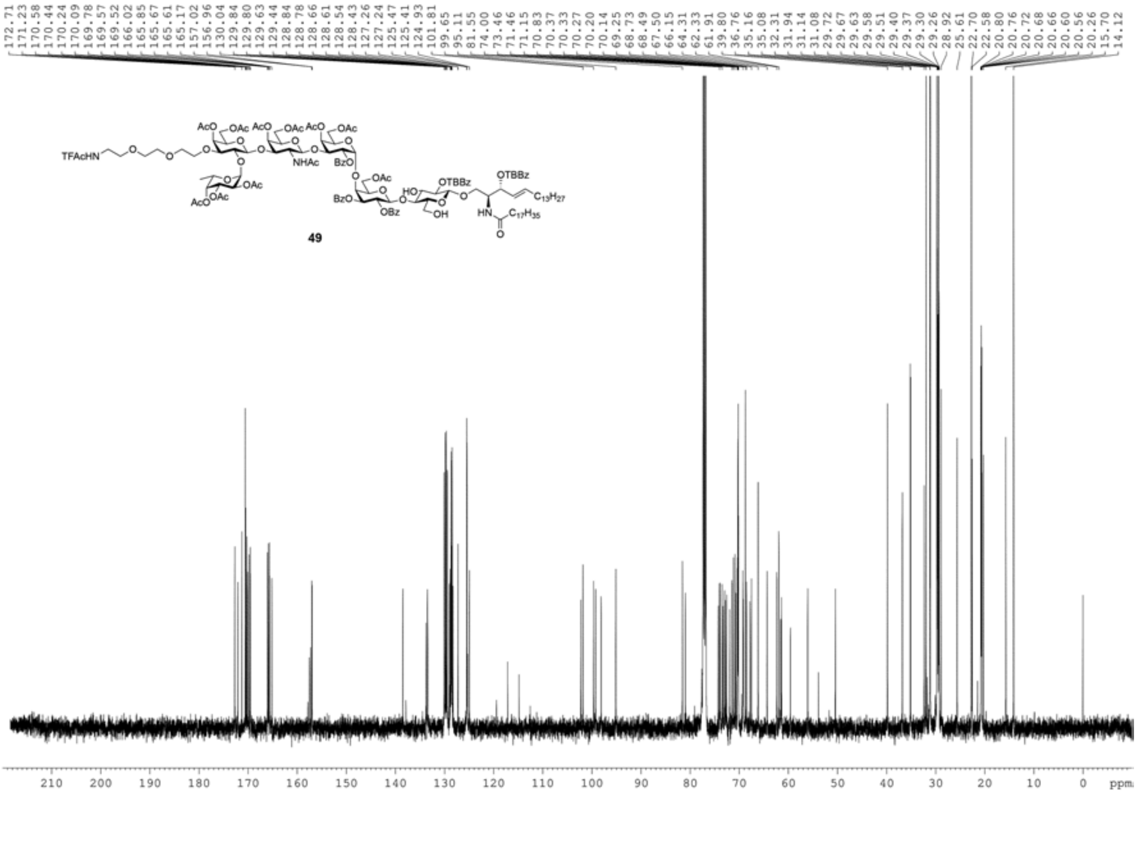
TFAcHN
49
ppm

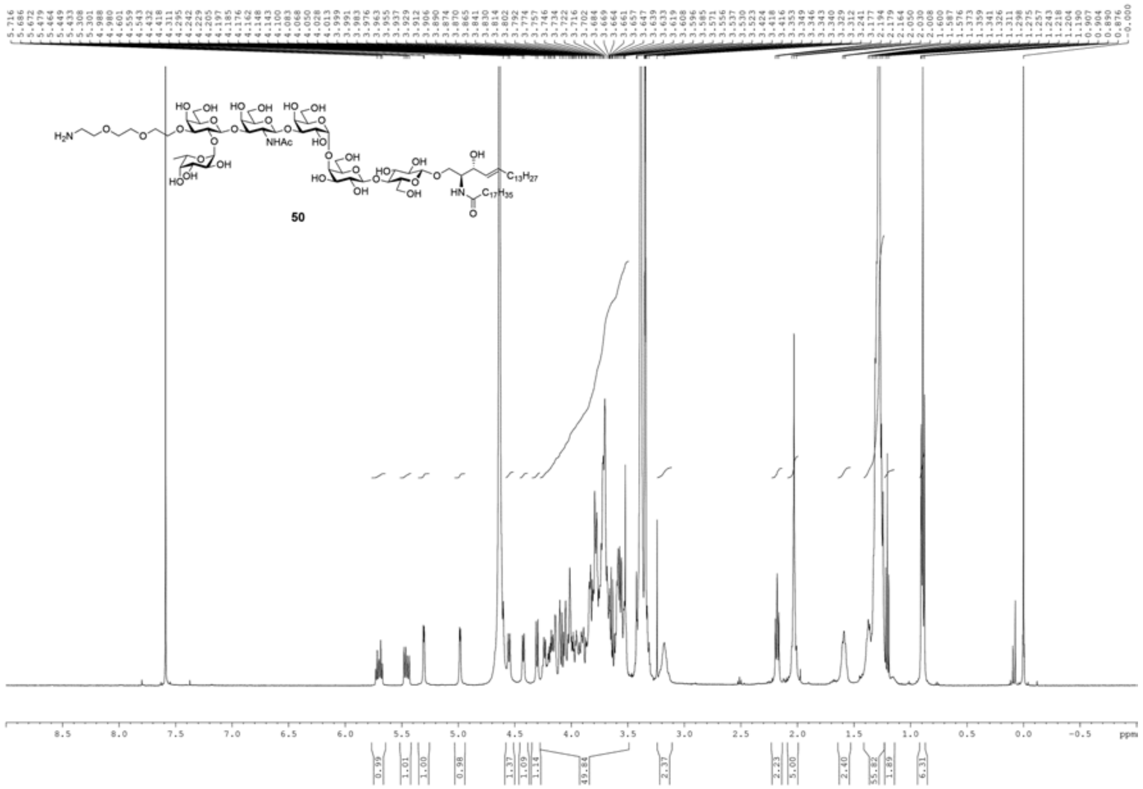

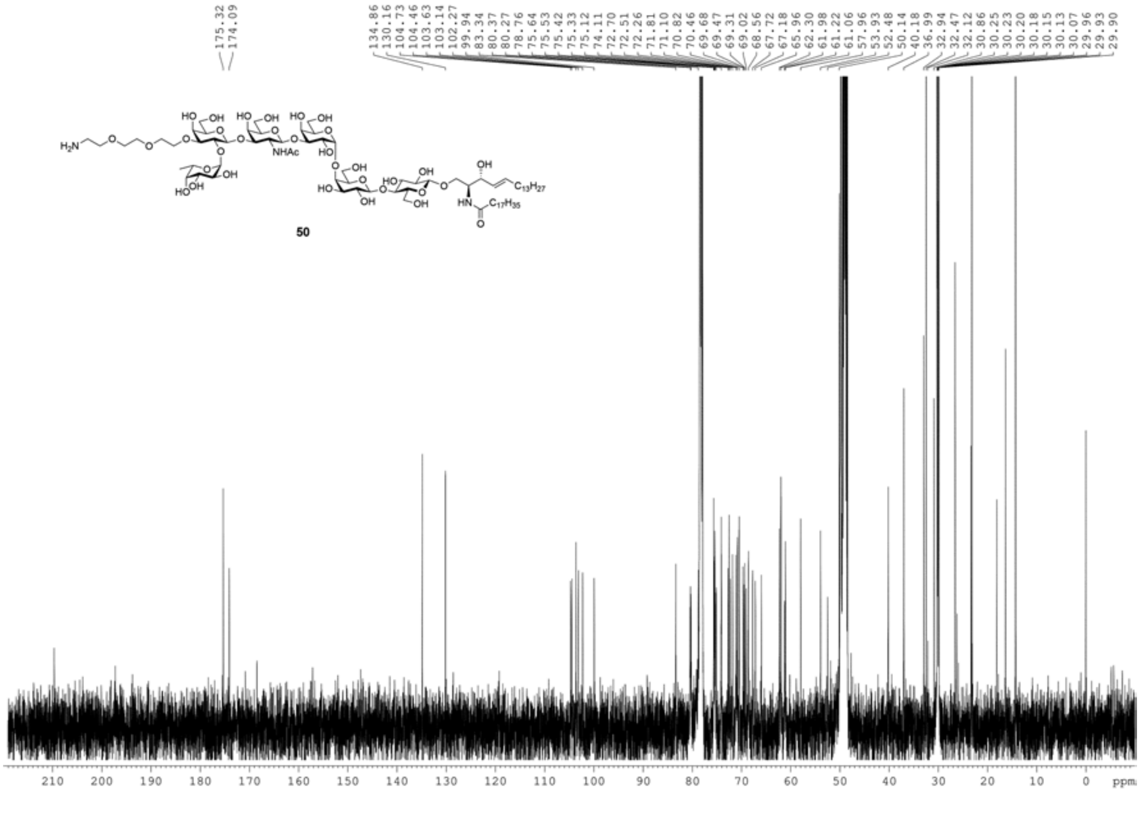

3. References

(S01) Gold, H.; Boot, R. G.; Aerts, J. M. F. G.; Overkleeft, H. S.; Codée, J. D. C.; van der Marel, G. A. A concise synthesis of globotriaosylsphingosine. *Eur*. *J*. *Org*. *Chem*. **2011**, 1652–1663.

(S02) Panchadhayee, R.; Misra, A. K. Regioselective reductive ring opening of benzylidene acetals using triethylsilane and iodine. *Synlett* **2010**, 1430–1430.

(S03) Asano, S.; Tanaka, H.-N.; Imamura, A.; Ishida, H.; Ando, H. *p*-*tert*-Butyl groups improve the utility of aromatic protective groups in carbohydrate synthesis. *Org*. *Lett*. **2019**, *21*, 4197–4200.

(S04) Gu, G.; Du, Y.; Pan, J. Concise synthesis of a buffalo milk pentasaccharide derivative. *Carbohydr*. *Res*. **2002**, *337*, 1313–1317.

(S05) Tanaka, H.; Ando, H.; Ishihara, H.; Koketsu, M. Sialylation reactions with 5-*N*,7-*O*-carbonyl-protected sialyl donors: unusual stereoselectivity with nitrile solvent assistance. *Carbohydr*. *Res*. **2008**, *343*, 1585–1593.

(S06) Hossain, M. I.; Hanashima, S.; Nomura, T.; Lethu, S.; Tsuchikawa, H.; Murata, M.; Kusaka, H.; Kita, S.; Maenaka, K. Synthesis and Th1-immunostimulatory activity of α-galactosylceramide analogues bearing a halogen-containing or selenium-containing acyl chain. *Bioorg*. *Med*. *Chem*. **2016**, *24*, 3687–3695.

(S07) Lu, G.; Burgess, K. A diversity oriented synthesis of 3’-O-modified nucleoside triphosphates for DNA ‘sequencing by synthesis’. *Bioorg*. *Med*. *Chem*. *Lett*. **2006**, *16*, 3902–3905.

(S08) Mandal, S. S.; Liao, G.; Guo, Z. Chemical synthesis of the tumor-associated Globo-H antigen. *RSC Adv*. **2015**, *5*, 23311–23319.
